# Shared heritability and functional enrichment across six solid cancers

**DOI:** 10.1038/s41467-018-08054-4

**Published:** 2019-01-25

**Authors:** Xia Jiang, Hilary K. Finucane, Fredrick R. Schumacher, Stephanie L. Schmit, Jonathan P. Tyrer, Younghun Han, Kyriaki Michailidou, Corina Lesseur, Karoline B. Kuchenbaecker, Joe Dennis, David V. Conti, Graham Casey, Mia M. Gaudet, Jeroen R. Huyghe, Demetrius Albanes, Melinda C. Aldrich, Angeline S. Andrew, Irene L. Andrulis, Hoda Anton-Culver, Antonis C. Antoniou, Natalia N. Antonenkova, Susanne M. Arnold, Kristan J. Aronson, Banu K. Arun, Elisa V. Bandera, Rosa B. Barkardottir, Daniel R. Barnes, Jyotsna Batra, Matthias W. Beckmann, Javier Benitez, Sara Benlloch, Andrew Berchuck, Sonja I. Berndt, Heike Bickeböller, Stephanie A. Bien, Carl Blomqvist, Stefania Boccia, Natalia V. Bogdanova, Stig E. Bojesen, Manjeet K. Bolla, Hiltrud Brauch, Hermann Brenner, James D. Brenton, Mark N. Brook, Joan Brunet, Hans Brunnström, Daniel D. Buchanan, Barbara Burwinkel, Ralf Butzow, Gabriella Cadoni, Trinidad Caldés, Maria A. Caligo, Ian Campbell, Peter T. Campbell, Géraldine Cancel-Tassin, Lisa Cannon-Albright, Daniele Campa, Neil Caporaso, André L. Carvalho, Andrew T. Chan, Jenny Chang-Claude, Stephen J. Chanock, Chu Chen, David C. Christiani, Kathleen B. M. Claes, Frank Claessens, Judith Clements, J. Margriet Collée, Marcia Cruz Correa, Fergus J. Couch, Angela Cox, Julie M. Cunningham, Cezary Cybulski, Kamila Czene, Mary B. Daly, Anna deFazio, Peter Devilee, Orland Diez, Manuela Gago-Dominguez, Jenny L. Donovan, Thilo Dörk, Eric J. Duell, Alison M. Dunning, Miriam Dwek, Diana M. Eccles, Christopher K. Edlund, Digna R Velez Edwards, Carolina Ellberg, D. Gareth Evans, Peter A. Fasching, Robert L. Ferris, Triantafillos Liloglou, Jane C. Figueiredo, Olivia Fletcher, Renée T. Fortner, Florentia Fostira, Silvia Franceschi, Eitan Friedman, Steven J. Gallinger, Patricia A. Ganz, Judy Garber, José A. García-Sáenz, Simon A. Gayther, Graham G. Giles, Andrew K. Godwin, Mark S. Goldberg, David E. Goldgar, Ellen L. Goode, Marc T. Goodman, Gary Goodman, Kjell Grankvist, Mark H. Greene, Henrik Gronberg, Jacek Gronwald, Pascal Guénel, Niclas Håkansson, Per Hall, Ute Hamann, Freddie C. Hamdy, Robert J. Hamilton, Jochen Hampe, Aage Haugen, Florian Heitz, Rolando Herrero, Peter Hillemanns, Michael Hoffmeister, Estrid Høgdall, Yun-Chul Hong, John L. Hopper, Richard Houlston, Peter J. Hulick, David J. Hunter, David G. Huntsman, Gregory Idos, Evgeny N. Imyanitov, Sue Ann Ingles, Claudine Isaacs, Anna Jakubowska, Paul James, Mark A. Jenkins, Mattias Johansson, Mikael Johansson, Esther M. John, Amit D. Joshi, Radka Kaneva, Beth Y. Karlan, Linda E. Kelemen, Tabea Kühl, Kay-Tee Khaw, Elza Khusnutdinova, Adam S. Kibel, Lambertus A. Kiemeney, Jeri Kim, Susanne K. Kjaer, Julia A. Knight, Manolis Kogevinas, Zsofia Kote-Jarai, Stella Koutros, Vessela N. Kristensen, Jolanta Kupryjanczyk, Martin Lacko, Stephan Lam, Diether Lambrechts, Maria Teresa Landi, Philip Lazarus, Nhu D. Le, Eunjung Lee, Flavio Lejbkowicz, Heinz-Josef Lenz, Goska Leslie, Davor Lessel, Jenny Lester, Douglas A. Levine, Li Li, Christopher I. Li, Annika Lindblom, Noralane M. Lindor, Geoffrey Liu, Fotios Loupakis, Jan Lubiński, Lovise Maehle, Christiane Maier, Arto Mannermaa, Loic Le Marchand, Sara Margolin, Taymaa May, Lesley McGuffog, Alfons Meindl, Pooja Middha, Austin Miller, Roger L. Milne, Robert J. MacInnis, Francesmary Modugno, Marco Montagna, Victor Moreno, Kirsten B. Moysich, Lorelei Mucci, Kenneth Muir, Anna Marie Mulligan, Katherine L. Nathanson, David E. Neal, Andrew R. Ness, Susan L. Neuhausen, Heli Nevanlinna, Polly A. Newcomb, Lisa F. Newcomb, Finn Cilius Nielsen, Liene Nikitina-Zake, Børge G. Nordestgaard, Robert L. Nussbaum, Kenneth Offit, Edith Olah, Ali Amin Al Olama, Olufunmilayo I. Olopade, Andrew F. Olshan, Håkan Olsson, Ana Osorio, Hardev Pandha, Jong Y. Park, Nora Pashayan, Michael T. Parsons, Tanja Pejovic, Kathryn L. Penney, Wilbert H M. Peters, Catherine M. Phelan, Amanda I. Phipps, Dijana Plaseska-Karanfilska, Miranda Pring, Darya Prokofyeva, Paolo Radice, Kari Stefansson, Susan J. Ramus, Leon Raskin, Gad Rennert, Hedy S. Rennert, Elizabeth J. van Rensburg, Marjorie J. Riggan, Harvey A. Risch, Angela Risch, Monique J. Roobol, Barry S. Rosenstein, Mary Anne Rossing, Kim De Ruyck, Emmanouil Saloustros, Dale P. Sandler, Elinor J. Sawyer, Matthew B. Schabath, Johanna Schleutker, Marjanka K. Schmidt, V. Wendy Setiawan, Hongbing Shen, Erin M. Siegel, Weiva Sieh, Christian F. Singer, Martha L. Slattery, Karina Dalsgaard Sorensen, Melissa C. Southey, Amanda B. Spurdle, Janet L. Stanford, Victoria L. Stevens, Sebastian Stintzing, Jennifer Stone, Karin Sundfeldt, Rebecca Sutphen, Anthony J. Swerdlow, Eloiza H. Tajara, Catherine M. Tangen, Adonina Tardon, Jack A. Taylor, M. Dawn Teare, Manuel R. Teixeira, Mary Beth Terry, Kathryn L. Terry, Stephen N. Thibodeau, Mads Thomassen, Line Bjørge, Marc Tischkowitz, Amanda E. Toland, Diana Torres, Paul A. Townsend, Ruth C. Travis, Nadine Tung, Shelley S. Tworoger, Cornelia M. Ulrich, Nawaid Usmani, Celine M. Vachon, Els Van Nieuwenhuysen, Ana Vega, Miguel Elías Aguado-Barrera, Qin Wang, Penelope M. Webb, Clarice R. Weinberg, Stephanie Weinstein, Mark C. Weissler, Jeffrey N. Weitzel, Catharine M. L. West, Emily White, Alice S. Whittemore, H-Erich Wichmann, Fredrik Wiklund, Robert Winqvist, Alicja Wolk, Penella Woll, Michael Woods, Anna H. Wu, Xifeng Wu, Drakoulis Yannoukakos, Wei Zheng, Shanbeh Zienolddiny, Argyrios Ziogas, Kristin K. Zorn, Jacqueline M. Lane, Richa Saxena, Duncan Thomas, Rayjean J. Hung, Brenda Diergaarde, James McKay, Ulrike Peters, Li Hsu, Montserrat García-Closas, Rosalind A. Eeles, Georgia Chenevix-Trench, Paul J. Brennan, Christopher A. Haiman, Jacques Simard, Douglas F. Easton, Stephen B. Gruber, Paul D. P. Pharoah, Alkes L. Price, Bogdan Pasaniuc, Christopher I. Amos, Peter Kraft, Sara Lindström

**Affiliations:** 1 Program in Genetic Epidemiology and Statistical Genetics, Harvard T.H. Chan School of Public Health, 677 Huntington Ave, Boston, MA 02115 USA; 2 Unit of Cardiovascular Epidemiology, Institute of Environmental Medicine, Karolinska Institutet, Nobels vagen 13, 17177 Stockholm, Sweden; 3 Department of Epidemiology, Harvard T.H. Chan School of Public Health, 677 Huntington Ave, Boston, MA 02115 USA; 4 Program in Medical and Population Genetics, Broad Institute of MIT and Harvard, 75 Ames St, Cambridge, MA 02142 USA; 5 Department of Population and Quantitative Health Sciences, Case Western Reserve University, 10900 Eucid Avenue, Cleveland, OH 44106 USA; 6 Seidman Cancer Center, University Hospitals, Cleveland, OH 44106 USA; 7 Department of Cancer Epidemiology, H. Lee Moffitt Cancer Center and Research Institute, 12902 Magnolia Dr. MRC-CANCONT, Tampa, FL 33612 USA; 8 Department of Gastrointestinal Oncology, H. Lee Moffitt Cancer Center and Research Institute, 12902 Magnolia Dr. MRC-CANCONT, Tampa, FL 33612 USA; 9 Centre for Cancer Genetic Epidemiology, Department of Oncology, University of Cambridge, 2 Worts’ Causeway, Cambridge, CB1 8RN UK; 10 Department of Biomedical Data Science, The Geisel School of Medicine at Dartmouth, 1 Medical Center Drive, Lebanon, NH 03756 USA; 11 Centre for Cancer Genetic Epidemiology, Department of Public Health and Primary Care, University of Cambridge, 2 Worts’ Causeway, Cambridge, CB1 8RN UK; 12 Department of Electron Microscopy/Molecular Pathology, The Cyprus Institute of Neurology and Genetics, 1683 Nicosia, Cyprus; 13 Genetic Epidemiology Group, International Agency for Research on Cancer, 150 Cours Albert Thomas, 69008 Lyon, France; 14 Section of Genetics, International Agency for Research on Cancer, 150 cours Albert Thomas, 69008 Lyon, France; 15 Division of Psychiatry, University College London, Maple House, 149 Tottenham Court Road, London, W1T 7NF UK; 16 UCL Genetics Institute, University College London, Gower Street, London, WC1E 6BT UK; 17 Department of Preventive Medicine, Keck School of Medicine, University of Southern California Norris Comprehensive Cancer Center, Los Angeles, CA 48109 USA; 18 Public Health Sciences, University of Virginia, P.O. Box 800717, Charlottesville, VI 22908 USA; 19 Center for Public Health Genomics, University of Virginia, P.O. Box 800717, Charlottesville, VI 22908 USA; 20 Epidemiology Research Program, American Cancer Society, 250 Williams Street NW, Atlanta, GA 30303 USA; 21 Public Health Sciences Division, Fred Hutchinson Cancer Research Center, 1100 Fairview Ave. N., Seattle, WA 98109-1024 USA; 22 Division of Cancer Epidemiology and Genetics, National Cancer Institute, 9609 Medical Center Dr, Rockville, MD 20850 USA; 23 Department of Thoracic Surgery, Division of Epidemiology, Vanderbilt University Medical Center, 609 Oxford House, Nashville, TN 37232 USA; 24 Department of Neurology, Dartmouth-Hitchcock Medical Center, 7927 Rubin Building, Room 860, One Medical Center Drive, Lebanon, NH 3756 USA; 25 Fred ALitwin Center for Cancer Genetics, Lunenfeld-Tanenbaum Research Institute of Mount Sinai Hospital, 600 University Avenue, Toronto, ON M5G1X5 Canada; 26 Department of Molecular Genetics, University of Toronto, 1 King’s College Circle, Toronto, ON M5S1A8 Canada; 27 Department of Epidemiology, Genetic Epidemiology Research Institute, University of California Irvine, 224 Irvine Hall, Irvine, CA 92617 USA; 28 NNAlexandrov Research Institute of Oncology and Medical Radiology, Settlement of Lesnoy-2, 223040 Minsk, Belarus; 29 Markey Cancer Center, University of Kentucky, 800 Rose Street, cc445, Lexington, KY 40508 USA; 30 Department of Public Health Sciences, and Cancer Research Institute, Queen’s University, 10 Stuart Street, Kingston, ON K7L 3N6 Canada; 31 Department of Breast Medical Oncology, University of Texas MD Anderson Cancer Center, 1155 Pressler St, Houston, TX 77030 USA; 32 Cancer Prevention and Control Program, Rutgers Cancer Institute of New Jersey, 195 Little Albany Street, Room 5568, New Brunswick, NJ 08903 USA; 33 Department of Pathology, Landspitali University Hospital, Hringbraut, Reykjavik, 101 Iceland; 34 BMC (Biomedical Centre), Faculty of Medicine, University of Iceland, Vatnsmyrarvegi 16, Reykjavik, 101 Iceland; 35 Australian Prostate Cancer Research Centre-Qld, Translational Research Institute, 37 Kent St, Woolloongabba, QLD 4102 Australia; 36 Institute of Health and Biomedical Innovation and School of Biomedical Science, Queensland University of Technology, 60 Musk Ave, Kelvin Grove, QLD 4059 Australia; 37 Department of Gynecology and Obstetrics, Comprehensive Cancer Center Erlangen Nuremberg, University Hospital Erlangen, Friedrich-Alexander-University Erlangen-Nuremberg, Universitaetsstrasse 21-23, 91054 Erlangen, Germany; 38 Human Cancer Genetics Programme, Spanish National Cancer Research Centre (CNIO), Calle de Melchor Fernández Almagro, 3, 28029 Madrid, Spain; 39 Biomedical Network on Rare Diseases (CIBERER), AvMonforte de Lemos, 3-5Pabellón 11Planta 0, 28029 Madrid, Spain; 40 Division of Genetics and Epidemiology, The Institute of Cancer Research, 15 Cotswold Road, London, SM2 5NG UK; 41 Department of Obstetrics and Gynecology, Duke University Medical Center, 25171 Morris Bldg, Durham, NC 27710 USA; 42 Department of Genetic Epidemiology, University Medical Center Goettingen, Humboldtallee 32, 37073 Goettingen, Germany; 43 School of Public Health, University of Washington, 1959 NE Pacific Street, Health Science Buidling, F-350, Seattle, WA 98195 USA; 44 Department of Oncology, Helsinki University Hospital, University of Helsinki, Haartmaninkatu 4, 00290 Helsinki, Finland; 45 Department of Oncology, Örebro University Hospital, 70185 Örebro, Sweden; 46 Fondazione Policlinico Universitario A. Gemelli IRCCS, 00168 Roma, Italy; 47 Università Cattolica del Sacro Cuore, 00168 Roma, Italy; 48 Department of Radiation Oncology, Hannover Medical School, Carl-Neuberg-Straße 1, 30625 Hannover, Germany; 49 Gynaecology Research Unit, Hannover Medical School, Carl-Neuberg-Straße 1, 30625 Hannover, Germany; 50 Copenhagen General Population Study, Herlev and Gentofte Hospital, Copenhagen University Hospital, Herlev Ringvej 75, 2730 Herlev, Denmark; 51 Department of Clinical Biochemistry, Herlev and Gentofte Hospital, Copenhagen University Hospital, Herlev Ringvej 75, 2730 Herlev, Denmark; 52 Faculty of Health and Medical Sciences, University of Copenhagen, Blegdamsvej 3B, 2200 Copenhagen, Denmark; 53 DrMargarete Fischer-Bosch-Institute of Clinical Pharmacology, Auerbachstr112, 70376 Stuttgart, Germany; 54 University of Tübingen, Geschwister-Scholl-Platz, 72074 Tübingen, Germany; 55 German Cancer Consortium (DKTK), German Cancer Research Center (DKFZ), Im Neuenheimer Feld 280, 69120 Heidelberg, Germany; 56 Division of Clinical Epidemiology and Aging Research, German Cancer Research Center (DKFZ), Im Neuenheimer Feld 280, 69120 Heidelberg, Germany; 57 Division of Preventive Oncology, German Cancer Research Center (DKFZ) and National Center for Tumor Diseases (NCT), Im Neuenheimer Feld 280, 69120 Heidelberg, Germany; 58 Cancer Research UK Cambridge Institute, University of Cambridge, Li Ka Shing Centre, Robinson Way, CB2 0RE Cambridge, UK; 59 Genetic Counseling Unit, Hereditary Cancer Program, IDIBGI (Institut d’Investigació Biomèdica de Girona), Catalan Institute of Oncology, CIBERONC, AvFrança s/n, 17007 Girona, Spain; 60 Clinical Sciences, Lund University, Box 117, 221 00 Lund, Sweden; 61 Department of Genetics and Pathology, Division of Laboratory Medicine, 221 85 Lund, Sweden; 62 University of Melbourne Centre for Cancer Research, Victorian Comprehensive Cancer Centre, Parkville, VIC 3010 Australia; 63 Colorectal Oncogenomics Group, Department of Clinical Pathology, The University of Melbourne, Parkville, VIC 3010 Australia; 64 Genomic Medicine and Family Cancer Clinic, Royal Melbourne Hospital, Parkville, VIC 3010 Australia; 65 Department of Obstetrics and Gynecology, University of Heidelberg, Im Neuenheimer Feld 440, 69120 Heidelberg, Germany; 66 Molecular Epidemiology Group, C080, German Cancer Research Center (DKFZ), Im Neuenheimer Feld 280, 69120 Heidelberg, Germany; 67 Department of Pathology, University of Helsinki and Helsinki University Hospital, Biomedicum Helsinki 4th floor, Haartmaninkatu 8, 00029 Helsinki, Finland; 68 Medical Oncology Department, Hospital Clínico San Carlos, Instituto de Investigación Sanitaria San Carlos (IdISSC), Centro Investigación Biomédica en Red de Cáncer (CIBERONC), Calle del Prof Martín Lagos, 28040 Madrid, Spain; 69 Section of Genetic Oncology, Department of Laboratory Medicine, University and University Hospital of Pisa, via Roma 67, 56126 Pisa, Italy; 70 Peter MacCallum Cancer Center, 305 Grattan Street, Melbourne, VIC 3000 Australia; 71 Sir Peter MacCallum Department of Oncology, The University of Melbourne, 305 Grattan Street, Melbourne, VIC 3000 Australia; 72 Sorbonne Université, GRC N°5 ONCOTYPE-URO, Tenon Hospital, 75020 Paris, France; 73 CeRePP, Tenon Hospital, 75020 Paris, France; 74 Division of Genetic Epidemiology, Department of Medicine, University of Utah School of Medicine, Salt Lake City, UT 84112 USA; 75 George EWahlen Department of Veterans Affairs Medical Center, Salt Lake City, UT 84112 USA; 76 Division of Cancer Epidemiology, German Cancer Research Center (DKFZ), Im Neuenheimer Feld 280, 69120 Heidelberg, Germany; 77 Department of Biology, University of Pisa, 56126 Pisa, Italy; 78 Molecular Oncology Research Center, Barretos Cancer Hospital, Rua Antenor Duarte Villela, 1331, Barretos, SP 784-400 Brazil; 79 Head and Neck Surgery Department, Barretos Cancer Hospital, Pio XII, 1331, Antenor Duarte Villela St, Barretos, SP 14784-400 Brazil; 80 Division of Gastroenterology, Massachusetts General Hospital, 55 Fruit Street, Boston, MA 02114 USA; 81 Channing Division of Network Medicine, Department of Medicine, Brigham and Women’s Hospital, Harvard Medical School, 181 Longwood Avenue, Boston, MA 02115 USA; 82 Cancer Epidemiology Group, University Cancer Center Hamburg (UCCH), University Medical Center Hamburg-Eppendorf, Martinistraße 52, 20246 Hamburg, Germany; 83 Program in Epidemiology, Division of Public Health Sciences, Fred Hutchinson Cancer Research Center, 1100 Fairview Ave N, Seattle, WA 98109 USA; 84 Centre for Medical Genetics, Ghent University, De Pintelaan 185, 9000 Gent, Belgium; 85 Molecular Endocrinology Laboratory, Department of Cellular and Molecular Medicine, KU Leuven, Leuven, 3000 Belgium; 86 Department of Clinical Genetics, Erasmus University Medical Center, Wytemaweg 80, 3015 Rotterdam, CN The Netherlands; 87 University of Puerto Rico Medical Sciences Campus and Comprehensive Cancer Center, San Juan, PR 00936 USA; 88 Department of Laboratory Medicine and Pathology, Mayo Clinic, 200 First StSW, Rochester, MN 55905 USA; 89 Sheffield Institute for Nucleic Acids (SInFoNiA), Department of Oncology and Metabolism, University of Sheffield, Western Bank, Sheffield, S10 2TN UK; 90 International Hereditary Cancer Center, Department of Genetics and Pathology, Pomeranian Medical University, ulUnii Lubelskiej 1, 71-252 Szczecin, Poland; 91 Department of Medical Epidemiology and Biostatistics, Karolinska Institutet, Karolinska Univ Hospital, 171 76 Stockholm, Sweden; 92 Department of Clinical Genetics, Fox Chase Cancer Center, 333 Cottman Ave, Philadelphia, PA 19111 USA; 93 Centre for Cancer Research, The Westmead Institute for Medical Research, The University of Sydney, 176 Hawkesbury Rd, Sydney, NSW 2145 Australia; 94 Department of Gynaecological Oncology, Westmead Hospital, Hawkesbury Rd & Darcy Rd, Sydney, NSW 2145 Australia; 95 Department of Pathology, Leiden University Medical Center, Albinusdreef 2, 2333 ZA Leiden, The Netherlands; 96 Department of Human Genetics, Leiden University Medical Center, Albinusdreef 2, 2333 ZA Leiden, The Netherlands; 97 Oncogenetics Group, Clinical and Molecular Genetics Area, Vall d’Hebron Institute of Oncology (VHIO), University Hospital, Vall d’Hebron, Passeig de la Vall d’Hebron 119-129, 08035 Barcelona, Spain; 98 Genomic Medicine Group, Galician Foundation of Genomic Medicine, Instituto de Investigación Sanitaria de Santiago de Compostela (IDIS), Complejo Hospitalario Universitario de Santiago, SERGAS, Travesía da Choupana S/N, 15706 Santiago de Compostela, Spain; 99 Moores Cancer Center, University of California, San Diego, 3855 Health Sciences Drive, La Jolla, CA 92037 USA; 100 School of Social and Community Medicine, University of Bristol, Bristol, BS8 1TH UK; 101 Unit of Nutrition and Cancer, Cancer Epidemiology Research Program, Catalan Institute of Oncology (ICO-IDIBELL), AvGran Via 199-203, L’Hospitalet de Llobregat, 08908 Barcelona, Spain; 102 Department of Biomedical Sciences, Faculty of Science and Technology, University of Westminster, 309 Regent Street, London, W1B 2HW UK; 103 Cancer Sciences Academic Unit, Faculty of Medicine, University of Southampton, Tremona Road, Southampton, SO16 6YD UK; 104 Department of Medicine, Keck School of Medicine, University of Southern California, Los Angeles, CA 90033 USA; 105 Vanderbilt Epidemiology Center, Vanderbilt Genetics Institute, Department of Obstetrics and Gynecology, Vanderbilt University Medical Center, 2525 West End Avenue, Suite 600, Nashville, TN 37203 USA; 106 Department of Cancer Epidemiology, Clinical Sciences, Lund University, Barngatan 4, Skånes universitetssjukhus, 222 42 Lund, Sweden; 107 Manchester Centre for Genomic Medicine, Division of Evolution and Genomic Sciences, University of Manchester, St Mary’s Hospital, Central Manchester University Hospitals NHS Foundation Trust, Oxford Road, Manchester, M13 9WL UK; 108 David Geffen School of Medicine, Department of Medicine Division of Hematology and Oncology, University of California at Los Angeles, 10833 Le Conte Ave, Los Angeles, CA 90095 USA; 109 Department of Otolaryngology, UPMC Hillman Cancer Center, Cancer Pavilion, University of Pittsburgh, Suite 500, 5150 Centre Avenue, Pittsburgh, PA 15232 USA; 110 Molecular and Clinical Cancer Medicine, Roy Castle Lung Cancer Research Programme, The University of Liverpool Institute of Translational Medicine, The Wiliam Duncan Building, 6 West Derby Street, Liverpool, L69 3BX UK; 111 Samuel Oschin Comprehensive Cancer Institute, Cedars-Sinai Medical Center, 8700 Beverly Boulevard, Los Angeles, CA 90048 USA; 112 Keck School of Medicine, University of Southern California, 1450 Biggy Street, Los Angeles, CA 90033 USA; 113 The Breast Cancer Now Toby Robins Research Centre, The Institute of Cancer Research, 123 Old Brompton Road, London, SW7 3RP UK; 114 Molecular Diagnostics Laboratory, INRASTES, National Centre for Scientific Research ‘Demokritos’, Neapoleos 10, AgParaskevi, Athens, 15310 Greece; 115 Section of Infections, International Agency for Research on Cancer, 150 cours Albert Thomas, 69008 Lyon, France; 116 The Susanne Levy Gertner Oncogenetics Unit, Chaim Sheba Medical Center, Emek HaEla St 1, 52621 Ramat Gan, Israel; 117 Sackler Faculty of Medicine, Tel Aviv University, Haim Levanon 30, 69978 Ramat Aviv, Israel; 118 Department of Surgery, Mount Sinai Hospital, 600 University Avenue, Toronto, ON M5G 1X5 Canada; 119 Samuel Lunenfeld Research Institute, 600 University Avenue, Toronto, ON M5G 1X5 Canada; 120 University Health Network Toronto General Hospital, 200 Elizabeth St, Toronto, ON M5G 2C4 Canada; 121 Schools of Medicine and Public Health, Division of Cancer Prevention & Control Research, Jonsson Comprehensive Cancer Centre, UCLA, 650 Charles Young Drive South, Los Angeles, CA 90095-6900 USA; 122 Cancer Risk and Prevention Clinic, Dana-Farber Cancer Institute, 450 Brookline Avenue, Boston, MA 02215 USA; 123 Department of Preventive Medicine, Keck School of Medicine, University of Southern California, 1975 Zonal Ave, Los Angeles, CA 90033 USA; 124 Center for Cancer Prevention and Translational Genomics, Samuel Oschin Comprehensive Cancer Institute, Cedars-Sinai Medical Center, Spielberg Building, 8725 Alden Dr, Los Angeles, CA 90048 USA; 125 Department of Biomedical Sciences, Cedars-Sinai Medical Center, Spielberg Building, 8725 Alden Dr, Los Angeles, CA 90048 USA; 126 Cancer Epidemiology & Intelligence Division, Cancer Council Victoria, 615 St Kilda Road, Melbourne, VIC 3004 Australia; 127 Centre for Epidemiology and Biostatistics, Melbourne School of Population and Global Health, The University of Melbourne, Level 1, 723 Swanston Street, Melbourne, VIC 3010 Australia; 128 Department of Epidemiology and Preventive Medicine, Monash University, Melbourne, VIC Australia; 129 Department of Pathology and Laboratory Medicine, University of Kansas Medical Center, 3901 Rainbow Blvd, Kansas City, KS 66160 USA; 130 Department of Medicine, McGill University, 1001 Decarie Boulevard, Montréal, QC H4A3J1 Canada; 131 Division of Clinical Epidemiology, Royal Victoria Hospital, McGill University, 1001 Decarie Boulevard, Montréal, QC H4A3J1 Canada; 132 Department of Dermatology, Huntsman Cancer Institute, University of Utah School of Medicine, 2000 Circle of Hope, Salt Lake City, UT 84112 USA; 133 Department of Health Sciences Research, Mayo Clinic, 200 First StSW, Rochester, MN 55905 USA; 134 Cancer Prevention and Control, Samuel Oschin Comprehensive Cancer Institute, Cedars-Sinai Medical Center, 8700 Beverly Blvd, Room 1S37, Los Angeles, CA 90048 USA; 135 Community and Population Health Research Institute, Department of Biomedical Sciences, Cedars-Sinai Medical Center, 8700 Beverly Blvd, Room 1S37, Los Angeles, CA 90048 USA; 136 Public Health Sciences Division, Swedish Cancer Institute, 1221 Madison StSte 300, Seattle, WA 98109 USA; 137 Unit of Clinical Chemistry, Department of Medical Biosciences, Umeå University, By 6M van 2, Sjukhusomradet, Umea universitet, 901 85 Umea, Sweden; 138 Clinical Genetics Branch, National Cancer Institute, DCEG, 9609 Medical Center Dr, Bethesda, MD 20850-9772 USA; 139 Cancer & Environment Group, Center for Research in Epidemiology and Population Health (CESP), INSERM, University Paris-Sud, University Paris-Saclay, 94805 Villejuif, France; 140 Department of Environmental Medicine, Division of Nutritional Epidemiology, Karolinska Institutet, Nobels väg 13, SE-171 77, SE-171 Stockholm, Sweden; 141 Department of Oncology, Södersjukhuset, Sjukhusbacken 10, 118 83 Stockholm, Sweden; 142 Molecular Genetics of Breast Cancer, German Cancer Research Center (DKFZ), Im Neuenheimer Feld 580, 69120 Heidelberg, Germany; 143 Nuffield Department of Surgical Sciences, Faculty of Medical Science, John Radcliffe Hospital, University of Oxford, Oxford, OX1 2JD UK; 144 Department of Surgical Oncology, Princess Margaret Cancer Centre, 610 University Avenue, Toronto, Ontario M5G2M9 Canada; 145 Department of Internal Medicine 1, University Hospital Dresden, Technische Universität Dresden (TU Dresden), 01307 Dresden, Germany; 146 National Institute of Occupational Health (STAMI), Gydas vei 8, 0033 Oslo, Norway; 147 Department of Gynecology and Gynecologic Oncology, DrHorst Schmidt Kliniken Wiesbaden, Ludwig-Erhard-Straße 100, 65199 Wiesbaden, Germany; 148 Department of Gynecology and Gynecologic Oncology, Kliniken Essen-Mitte/ EvangHuyssens-Stiftung/ Knappschaft GmbH, Henricistrasse 92, 45136 Essen, Germany; 149 Early Detection and Prevention Section, International Agency for Research on Cancer, 150 cours Albert Thomas, 69008 Lyon, France; 150 Department of Virus, Lifestyle and Genes, Danish Cancer Society Research Center, Strandboulevarden 49, DK-2100 Copenhagen, Denmark; 151 Molecular Unit, Department of Pathology, Herlev Hospital, University of Copenhagen, Herlev Ringvej 75, DK-2730 Herlev, Denmark; 152 Preventive Medicine, Seoul National University College of Medicine, 1 Gwanak-ro, Gwanak-gu, Seoul, 151 742 Korea; 153 German Research Center for Environmental Health, Institute for Cancer Research, Ingolstadter Landstr1, London, SM2 5NG UK; 154 Center for Medical Genetics, NorthShore University HealthSystem, 1000 Central St, Evanston, IL 60201 USA; 155 The University of Chicago Pritzker School of Medicine, 924 E 57th St, Chicago, IL 60637 USA; 156 British Columbia’s Ovarian Cancer Research (OVCARE) Program, Vancouver General Hospital, BC Cancer Agency and University of British Columbia, #3427-600 West 10th Avenue, Vancouver, BC V5Z 4E6 Canada; 157 Department of Molecular Oncology, BC Cancer Agency Research Centre, #3427-600 West 10th Avenue, Vancouver, BC V5Z 4E6 Canada; 158 Department of Pathology and Laboratory Medicine, University of British Columbia, #3427-600 West 10th Avenue, Vancouver, BC V5Z 4E6 Canada; 159 NNPetrov Institute of Oncology, Leningradskaya ul, 68, StPetersburg, Russia 197758; 160 Lombardi Comprehensive Cancer Center, Georgetown University, 3800 Reservoir Road, Washington, DC 20007 USA; 161 Independent Laboratory of Molecular Biology and Genetic Diagnostics, Pomeranian Medical University, Rybacka 1, 70-204 Szczecin, Poland; 162 Parkville Familial Cancer Centre, Peter MacCallum Cancer Center, 305 Grattan Street, Melbourne, VIC 3000 Australia; 163 Department of Radiation Sciences, Umeå University, By 6M van 2, Sjukhusomradet, Umea universitet, 901 85 Umea, Sweden; 164 Department of Medicine, Division of Oncology and Stanford Cancer Institute, Stanford University School of Medicine, 780 Welch Rd, Stanford, CA 94304 USA; 165 Clinical and Translational Epidemiology Unit, Massachusetts General Hospital, 02114 Boston, MA USA; 166 Molecular Medicine Center, Department of Medical Chemistry and Biochemistry, Medical Faculty, Medical University of Sofia, Sofia, 1504 Bulgaria; 167 Women’s Cancer Program at the Samuel Oschin Comprehensive Cancer Institute, Cedars-Sinai Medical Center, 8700 Beverly Boulevard, Los Angeles, CA 90048 USA; 168 Hollings Cancer Center and Department of Public Health Sciences, Medical University of South Carolina, 68 President Street Bioengineering Building, MSC955, Charleston, SC 29425 USA; 169 Cancer Epidemiology, University Cancer Center Hamburg (UCCH), University Medical Center Hamburg-Eppendorf, Martinistraße 52, 20246 Hamburg, Germany; 170 Clinical Gerontology, Department of Public Health and Primary Care, University of Cambridge, 2 Worts’ Causeway, Cambridge, CB1 8RN UK; 171 Department of Genetics and Fundamental Medicine, Bashkir State University, ulZaki Validi 32, Ufa, Russia 450076; 172 Institute of Biochemistry and Genetics, Ufa Scientific Center of Russian Academy of Sciences, 71 prosp Oktyabrya, Ufa, Russia 450054; 173 Division of Urologic Surgery, Brigham and Womens Hospital, Boston, Massachusettes 02115 USA; 174 Radboud Institute for Health Sciences, Radboud University Medical Center, Geert Grooteplein 21, 6525 EZ Nijmegen, The Netherlands; 175 Department of Genitourinary Medical Oncology, University of Texas MD Anderson Cancer Center, 1155 Pressler St, Houston, TX 77030 USA; 176 Department of Gynaecology, Rigshospitalet, University of Copenhagen, Blegdamsvej 9, DK-2100 Copenhagen, Denmark; 177 Prosserman Centre for Population Health Research, Lunenfeld-Tanenbaum Research Institute, Sinai Health System, 60 Murray Street, Toronto, Ontario M5T 3L9 Canada; 178 Division of Epidemiology, Dalla Lana School of Public Health, University of Toronto, 155 College Street, Toronto, ON M5T3M7 Canada; 179 Centre for Research in Environmental Epidemiology (CREAL), ISGlobal, 08036 Barcelona, Spain; 180 IMIM (Hospital del Mar Research Institute), Barcelona, 08003 Spain; 181 Universitat Pompeu Fabra (UPF), Barcelona, 08002 Spain; 182 Division of Cancer Epidemiology and Genetics, National Cancer Institute, Department of Health and Human Services, National Institutes of Health, 9609 Medical Center Dr, Bethesda, MD 20892 USA; 183 Department of Cancer Genetics, Institute for Cancer Research, Oslo University Hospital Radiumhospitalet, Ullernchausseen 70, 0379 Oslo, Norway; 184 Faculty of Medicine, Institute of Clinical Medicine, University of Oslo, Kirkeveien 166, 0450 Oslo, Norway; 185 Department of Clinical Molecular Biology, Oslo University Hospital, University of Oslo, Kirkeveien 166, 0450 Oslo, Norway; 186 Department of Pathology and Laboratory Diagnostics, the Maria Sklodowska-Curie Institute - Oncology Center, Roentgena 5, 02-781 Warsaw, Poland; 187 Head and Neck Surgery, Department of Otorhinolaryngology, Maastricht University Medical Center, PDebyelaan 25, POBox 5800, 6202 AZ Maastricht, The Netherlands; 188 Department of Integrative Oncology, British Columbia Cancer Agency, Room 10-111 675 West 10th Avenue, Vancouver, BC V5Z1L3 Canada; 189 VIB Center for Cancer Biology, VIB, Herestraat 49, 3001 Leuven, Belgium; 190 Laboratory for Translational Genetics, Department of Human Genetics, University of Leuven, Oude Markt 13, 3000 Leuven, Belgium; 191 Integrative Tumor Epidemiology Branch, DCEG, National Cancer Institute, 9609 Medical Center Drive, Room SG/7E106, Rockville, MD 20850 USA; 192 College of Pharmacy, Washington State University, PBS 431 PO Box 1495, Spokane, WA 99210-1495 USA; 193 Cancer Control Research, BC Cancer Agency, 675 West 10th Avenue, Vancouver, BC V5Z 1L3 Canada; 194 Clalit Health Services, Clalit National Israeli Cancer Control Center, Carmel Medical Center, 2 Horev Street, 3436212 Haifa, Israel; 195 Institute of Human Genetics, University Medical Center Hamburg-Eppendorf, Martinistraße 52, 20246 Hamburg, Germany; 196 Gynecology Service, Department of Surgery, Memorial Sloan Kettering Cancer Center, 1275 York Avenue, New York, NY 10065 USA; 197 Gynecologic Oncology, Laura and Isaac Pearlmutter Cancer Center, NYU Langone Medical Center, 240 East 38th Street 19th Floor, New York, NY 10016 USA; 198 Department of Family Medicine and Community Health, Mary Ann Swetland Center for Environmental Health, Case Western Reserve University, Cleveland, OH 44106 USA; 199 Servicio Galego de Saude (SERGAS), Instituto de Investigación Sanitaria de Santiago de Compostela (IDIS), 15706 Santiago De Compostela, Spain; 200 Translational Research Program, Fred Hutchinson Cancer Research Center, Seattle, WA 98109 USA; 201 Department of Molecular Medicine and Surgery, Karolinska Institutet, Karolinska Univ Hospital, 171 76 Stockholm, Sweden; 202 Health Sciences Research, Mayo Clinic Arizona, 13400 EShea Blvd, Scottsdale, AZ 85259 USA; 203 Epidemiology Division, Princess Margaret Cancer Centre, 610 University Avenue, Toronto, ON M5G2M9 Canada; 204 Unit of Oncology 1, Department of Clinical and Experimental Oncology, Istituto Oncologico Veneto IRCCS, 35122 Padua, Italy; 205 Department of Medical Genetics, Oslo University Hospital, Kirkeveien 166, 0450 Oslo, Norway; 206 Institute of Human Genetics, University Hospital Ulm, Prittwitzstrasse 43, 89075 Ulm, Germany; 207 Translational Cancer Research Area, University of Eastern Finland, Yliopistonranta 1, 70210 Kuopio, Finland; 208 Institute of Clinical Medicine, Pathology and Forensic Medicine, University of Eastern Finland, Kuopio, Yliopistonranta 1, 70210 Finland; 209 Imaging Center, Department of Clinical Pathology, Kuopio University Hospital, Puijonlaaksontie 2, 70210 Kuopio, Finland; 210 Epidemiology Program, University of Hawaii Cancer Center, 701 Ilalo St, Honolulu, HI 96813 USA; 211 Department of Clinical Science and Education, Södersjukhuset, Karolinska Institutet, Stockholm, 17177 Sweden; 212 Division of Gynecologic Oncology, University Health Network, Princess Margaret Hospital, 610 University Avenue, OPG Wing, 6-811, Toronto, ON M5G 2M9 Canada; 213 Division of Gynaecology and Obstetrics, , Technische Universität München, Arcisstraße 21, 80333 Munich, Germany; 214 Faculty of Medicine, University of Heidelberg, In Neuenheimer Feld 672, 69120 Heidelberg, Germany; 215 NRG Oncology, Statistics and Data Management Center, Roswell Park Cancer Institute, Elm & Carlton Streets, Buffalo, NY 14263 USA; 216 Womens Cancer Research Center, Magee-Womens Research Institute and Hillman Cancer Center, Pittsburgh, PA 15213 USA; 217 Division of Gynecologic Oncology, Department of Obstetrics, Gynecology and Reproductive Sciences, University of Pittsburgh School of Medicine, 300 Halket Street, Pittsburgh, PA 15213 USA; 218 Immunology and Molecular Oncology Unit, Veneto Institute of Oncology IOV - IRCCS, Via Gattamelata 64, Padua, 35128 Italy; 219 Catalan Institute of Oncology, Bellvitge Biomedical Research Institute (IDIBELL), Consortium for Biomedical Research in Epidemiology and Public Health (CIBERESP) and University of Barcelona, Barcelona, 08908 Spain; 220 Division of Cancer Prevention and Control, Roswell Park Cancer Institute, Elm & Carlton Streets, Buffalo, NY 14263 USA; 221 Division of Population Health, Health Services Research and Primary Care, University of Manchester, Oxford Road, Manchester, M13 9PL UK; 222 Division of Health Sciences, Warwick Medical School, University of Warwick, Coventry, CV4 7AL UK; 223 Department of Laboratory Medicine and Pathobiology, University of Toronto, 1 King’s College Circle, Toronto, ON M5S1A8 Canada; 224 Laboratory Medicine Program, University Health Network, 200 Elizabeth Street, Toronto, ON M5G2C4 Canada; 225 Department of Medicine, Abramson Cancer Center, Perelman School of Medicine at the University of Pennsylvania, 3400 Civic Center Boulevard, Philadelphia, PA 19104 USA; 226 Department of Oncology, Addenbrooke’s Hospital, University of Cambridge, Cambridge, CB1 8RN UK; 227 NIHR Bristol Biomedical Research Centre Nutrition Theme, University of Bristol, Upper Maudlin Street, Bristol, BS2 8AE UK; 228 Department of Population Sciences, Beckman Research Institute of City of Hope, 1500 E Duarte, Duarte, CA 91010 USA; 229 Department of Obstetrics and Gynecology, Helsinki University Hospital, University of Helsinki, Haartmaninkatu 8, 00290 Helsinki, Finland; 230 Department of Urology, University of Washington, Seattle, Washington 98195 USA; 231 Center for Genomic Medicine, Rigshospitalet, Copenhagen University Hospital, Blegdamsvej 9, DK-2100 Copenhagen, Denmark; 232 Latvian Biomedical Research and Study Centre, Ratsupites str 1, Riga, LV-1067 Latvia; 233 Cancer Genetics and Prevention Program, University of California San Francisco, 1600 Divisadero St, San Francisco, CA 94143-1714 USA; 234 Clinical Genetics Research Lab, Department of Cancer Biology and Genetics, Memorial Sloan-Kettering Cancer Center, 1275 York Avenue, New York, NY 10065 USA; 235 Clinical Genetics Service, Department of Medicine, Memorial Sloan-Kettering Cancer Center, 1275 York Avenue, New York, NY 10065 USA; 236 Department of Molecular Genetics, National Institute of Oncology, Ráth György u7-9, 1122 Budapest, Hungary; 237 Department of Clinical Neurosciences, University of Cambridge, Cambridge, CB2 0QQ UK; 238 Center for Clinical Cancer Genetics, The University of Chicago, 5841S Maryland Ave, Chicago, IL 60637 USA; 239 Department of Epidemiology, Gillings School of Global Public Health, University of North Carolina, 135 Dauer Dr, Chapel Hill, NC 27599-7435 USA; 240 UNC Lineberger Comprehensive Cancer Center, 450 West Dr, Chapell Hill, NC 27599 USA; 241 The University of Surrey, Guildford, Surrey GU2 7XH UK; 242 Department of Cancer Epidemiology, HLee Moffitt Cancer Center and Research Institute, 12902 Magnolia Drive, Tampa, FL 33612 USA; 243 Department of Applied Health Research, University College London, 1-19 Torrington Place, London, WC1E 6BT UK; 244 Centre for Cancer Genetic Epidemiology, Department of Oncology, Strangeways Laboratory, University of Cambridge, Cambridge, CB1 8RN UK; 245 Department of Genetics and Computational Biology, QIMR Berghofer Medical Research Institute, 300 Herston Road, Brisbane, QLD 4006 Australia; 246 Department of Obstetrics and Gynecology, Oregon Health & Science University, 3181 SW Sam Jackson Park Road, L-466, Portland, OR 97239 USA; 247 Knight Cancer Institute, Oregon Health & Science University, 3181 SW Sam Jackson Park Road, L-466, Portland, OR 97239 USA; 248 Department of Gastroenterology, Radboud University Nijmegen Medical Center, Geert Grooteplein Zuid 10, Internal BOBox 433, 6525 GA Nijmegen, The Netherlands; 249 Department of Epidemiology, University of Washington School of Public Health, 1959 NE Pacific St, Seattle, WA 98195 USA; 250 Research Centre for Genetic Engineering and Biotechnology ‘Georgi DEfremov’, Macedonian Academy of Sciences and Arts, Boulevard Krste Petkov Misirkov, 1000 Skopje, Republic of Macedonia; 251 Bristol Dental School, University of Bristol, Lower Maudlin Street, Bristol, BS1 2LY UK; 252 Unit of Molecular Bases of Genetic Risk and Genetic Testing, Department of Research, Fondazione IRCCS (Istituto Di Ricovero e Cura a Carattere Scientifico) Istituto Nazionale dei Tumori (INT), Via Giacomo Venezian 1, 20133 Milan, Italy; 253 Decode genetics, Sturlugata 8, IS-101 Reykjavik, Reykjavik Iceland, Iceland; 254 School of Women’s and Children’s Health, Faculty of Medicine, University of NSW Sydney, 18 High St, Sydney, NSW 2052 Australia; 255 The Kinghorn Cancer Centre, Garvan Institute of Medical Research, 384 Victoria Street, Sydney, NSW 2010 Australia; 256 Division of Epidemiology, Department of Medicine, Vanderbilt Epidemiology Center, Vanderbilt-Ingram Cancer Center, Vanderbilt University School of Medicine, 1161 21st Ave S # D3300, Nashville, TN 37232 USA; 257 Clalit National Cancer Control Center, Carmel Medical Center and Technion Faculty of Medicine, 7 Michal Street, 34362 Haifa, Israel; 258 Department of Genetics, University of Pretoria, Private Bag X323, Arcadia, 0007 South Africa; 259 Department of Chronic Disease Epidemiology, Yale School of Public Health, 60 College St, New Haven, CT 06510 USA; 260 Cancer Center Cluster Salzburg at PLUS, Department of Molecular Biology, University of Salzburg, Billrothstr11, 5020 Salzburg, Austria; 261 Division of Epigenomics and Cancer Risk Factors, DKFZ – German Cancer Research Center, Im Neuenheimer Feld 280, 69120 Heidelberg, Germany; 262 Member of the German Center for Lung Research (DZL), Translational Lung Research Center Heidelberg (TLRC-H), 69120 Heidelberg, Germany; 263 Department of Urology, Erasmus University Medical Center, Wytemaweg 80, 3015 CN Rotterdam, The Netherlands; 264 Department of Radiation Oncology, Icahn School of Medicine at Mount Sinai, 1425 Madison Avenue, New York, NY 10029 USA; 265 Department of Genetics and Genomic Sciences, Icahn School of Medicine at Mount Sinai, 1425 Madison Avenue, New York, NY 10029 USA; 266 Department of Epidemiology, University of Washington, M4 C308, 1100 Fairview Ave N, Seattle, WA 98109 USA; 267 Faculty of Medicine and Health Sciences, Basic Medical Sciences, Ghent University, De Pintelaan 185, 9000 Gent, Belgium; 268 Hereditary Cancer Clinic, University Hospital of Heraklion, Voutes, 711 10 Heraklion, Greece; 269 Epidemiology Branch, National Institute of Environmental Health Sciences, NIH, 111TWAlexander Drive, Research Triangle Park, NC 27709 USA; 270 Research Oncology, Guy’s Hospital, King’s College London, Guy’s Hospital Great Maze Pond, London, SE1 9RT UK; 271 Institute of Biomedicine, University of Turku, 20014 Turku, Finland; 272 Division of Laboratory, Department of Medical Genetics, Turku University Hospital, 20014 Turku, Finland; 273 Prostate Cancer Research Center, Faculty of Medicine and Life Sciences and BioMediTech Institute, University of Tampere, 33014 Tampere, Finland; 274 Division of Molecular Pathology, The Netherlands Cancer Institute - Antoni van Leeuwenhoek Hospital, Plesmanlaan 121, 1066 CX Amsterdam, The Netherlands; 275 Division of Psychosocial Research and Epidemiology, The Netherlands Cancer Institute - Antoni van Leeuwenhoek hospital, Plesmanlaan 121, 1066 CX Amsterdam, The Netherlands; 276 Department of Preventive Medicine, Keck School of Medicine, University of Southern California, 1450 Biggy Street, Los Angeles, CA 90033 USA; 277 Department of Epidemiology and Biostatistics, Jiangsu Key Lab of Cancer Biomarkers, Prevention and Treatment, Collaborative Innovation Center for Cancer Personalized Medicine, School of Public Health, Nanjing Medical University, 101 Longmian Ave, Jiangning District, 211166 Nanjing, People’s Republic of China; 278 Department of Genetics and Genomic Sciences, Department of Population Health Science and Policy, Icahn School of Medicine at Mount Sinai, 1425 Madison Avenue, 2nd floor, New York, NY 10029 USA; 279 Dept of OB/GYN and Comprehensive Cancer Center, Medical University of Vienna, Waehringer Guertel 18-20, 1090 Vienna, Austria; 280 Department of Internal Medicine, University of Utah Health Sciences Center, 295 Chipeta Way, Salt Lake City, UT 84132 USA; 281 Department of Molecular Medicine, Aarhus University Hospital, DK-8200 Aarhus, Denmark; 282 Department of Clinical Medicine, Aarhus University, DK-8200 Aarhus, Denmark; 283 Precision Medicine, School of Clinical Sciences at Monash Health, Monash University, 246 Clayton Road, Clayton, VIC 3168 Australia; 284 Department of Clinical Pathology, The University of Melbourne, Cnr Grattan Street and Royal Parade, Melbourne, VIC 3010 Australia; 285 Department of Medicine III, University Hospital, LMU Munich, Marchioninistr15, 81377 Munich, Germany; 286 The Curtin UWA Centre for Genetic Origins of Health and Disease, Curtin University and University of Western Australia, 35 Stirling Hwy, Perth, WA 6000 Australia; 287 Department of Obstetrics and Gynecology, Sahlgrenska Cancer Center, Inst Clinical Scienses, University of Gothenburg, Blå stråket 6, 41345 Gothenburg, Sweden; 288 Epidemiology Center, College of Medicine, University of South Florida, 3650 Spectrum Blvd, Suite 100, Tampa, FL 33612 USA; 289 Division of Breast Cancer Research, The Institute of Cancer Research, London, SW7 3RP UK; 290 Department of Molecular Biology, School of Medicine of São José do Rio Preto, Av Brig Faria Lima 5416 Vila São Pedro, São José do Rio Preto, SP 15090-000 Brazil; 291 Department of Genetics and Evolutive Biology, Institute of Biosciences, University of São Paulo, Rua do Matão, 321, São Paulo, SP 05508-090 Brazil; 292 SWOG Statistical Center, Fred Hutchinson Cancer Research Center, Seattle, Washington 98109 USA; 293 Faculty of Medicine, University of Oviedo and CIBERESP, Campus del Cristo s/n, 33006 Oviedo, Spain; 294 Epigenetic and Stem Cell Biology Laboratory, National Institute of Environmental Health Sciences, NIH, 111TWAlexander Drive, Research Triangle Park, NC 27709 USA; 295 Medical Statistics Group, School of Health and Related Research (ScHARR), University of Sheffield, Regent Court, 30 Regent Street, Sheffield, S1 4DA UK; 296 Department of Genetics, Portuguese Oncology Institute, Rua DrAntónio Bernardino de Almeida 62, 4220-072 Porto, Portugal; 297 Biomedical Sciences Institute (ICBAS), University of Porto, RJorge de Viterbo Ferreira 228, 4050-013 Porto, Portugal; 298 Department of Epidemiology, Mailman School of Public Health, Columbia University, 722 West 168th Street, New York, NY 10032 USA; 299 Obstetrics and Gynecology Epidemiology Center, Brigham and Women’s Hospital, 221 Longwood Avenue RFB 368, Boston, MA 02115 USA; 300 Harvard THChan School of Public Health, 221 Longwood Avenue RFB 368, Boston, MA 02115 USA; 301 Department of Clinical Genetics, Odense University Hospital, Sonder Boulevard 29, 5000 Odence C, Denmark; 302 Department of Gynecology and Obstetrics, Haukeland University Hospital, 5021 Bergen, Norway; 303 Centre for Cancer Biomarkers CCBIO, Department of Clinical Science, University of Bergen, 5021 Bergen, Norway; 304 Program in Cancer Genetics, Departments of Human Genetics and Oncology, McGill University, 1001 Decarie Boulevard, Montréal, QC H4A3J1 Canada; 305 Department of Medical Genetics, Cambridge University, Hills Road, Cambridge, CB2 0QQ UK; 306 Department of Cancer Biology and Genetics, The Ohio State University, 460W12th Avenue, Columbus, OH 43210 USA; 307 Institute of Human Genetics, Pontificia Universidad Javeriana, Carrera 7 No40-90, Bogota, Colombia; 308 Division of Cancer Sciences, Manchester Cancer Research Centre, Faculty of Biology, Medicine and Health, Manchester Academic Health Science Centre, NIHR Manchester Biomedical Research Centre, Health Innovation Manchester, University of Manchester, Manchester, M20 4GJ UK; 309 Cancer Epidemiology Unit, Nuffield Department of Population Health, University of Oxford, Oxford, OX3 7LF UK; 310 Department of Medical Oncology, Beth Israel Deaconess Medical Center, 330 Brookline Avenue, Boston, MA 02215 USA; 311 Huntsman Cancer Institute and Department of Population Health Sciences, University of Utah, 2000 Circle of Hope, Rm 4125, Salt Lake City, UT 84112 USA; 312 Department of Oncology, Cross Cancer Institute, University of Alberta, 116 St & 85 Ave, Edmonton, AB T6G 2R3 Canada; 313 Division of Radiation Oncology, Cross Cancer Institute, University of Alberta, 116 St & 85 Ave, Edmonton, AB T6G 2R3 Canada; 314 Division of Gynecologic Oncology, Department of Obstetrics and Gynaecology and Leuven Cancer Institute, University Hospitals Leuven, Herestraat 49, 3000 Leuven, Belgium; 315 Fundación Pública Galega Medicina Xenómica & Instituto de Investigación Sanitaria de Santiago de Compostela, calle Choupana s/n, 15706 Santiago De Compostela, Spain; 316 Population Health Department, QIMR Berghofer Medical Research Institute, 300 Herston Road, Brisbane, QLD 4006 Australia; 317 Biostatistics and Computational Biology Branch, National Institute of Environmental Health Sciences, NIH, 111TWAlexander Drive, Research Triangle Park, NC 27709 USA; 318 Department of Otolaryngology/Head and Neck Surgery, University of North Carolina at Chapel Hill, Chapel Hill, 27514 NC USA; 319 City of Hope Clinical Cancer Genomics Community Research Network, 1500 East Duarte Road, Duarte, CA 91010 USA; 320 Division of Cancer Sciences, University of Manchester, Manchester Cancer Research Centre, Manchester Academic Health Science Centre,, The Christie Hospital NHS Foundation Trust, Manchester, M13 9PL UK; 321 Fred Hutchinson Cancer Research Center, 1100 Fairview Ave N, Seattle, WA 98109 USA; 322 Department of Epidemiology, University of Washington, 1100 Fairview Ave N, Seattle, WA 98109 USA; 323 Department of Health Research and Policy - Epidemiology, Stanford University School of Medicine, 259 Campus Drive, Stanford, CA 94305 USA; 324 Department of Biomedical Data Science, Stanford University School of Medicine, 259 Campus Drive, Stanford, CA 94305 USA; 325 Institute of Medical Informatics, Biometry and Epidemiology, Chair of Epidemiology, Ludwig Maximilians University, Neuherberg D-85764, Munich, 803539 Bavaria Germany; 326 Helmholtz Zentrum Munchen, German Research Center for Environmental Health (GmbH), Institute of Epidemiology, Ingolstadter Landstr1, 85764 Neuherberg, Germany; 327 Institute of Medical Statistics and Epidemiology, Technical University Munich, Munich, 80333 Germany; 328 Laboratory of Cancer Genetics and Tumor Biology, Cancer and Translational Medicine Research Unit, Biocenter Oulu, University of Oulu, Aapistie 5A, 90220 Oulu, Finland; 329 Laboratory of Cancer Genetics and Tumor Biology, Northern Finland Laboratory Centre Oulu, Aapistie 5A, 90220 Oulu, Finland; 330 Department of Surgical Sciences, Uppsala University, 751 85 Uppsala, Sweden; 331 Academic Unit of Clinical Oncology, University of Sheffield, Weston Park Hospital, Whitham Road, Sheffield, S10 2SJ UK; 332 Discipline of Genetics, Memorial University of Newfoundland, StJohn’s, NL A1C 5S7 Canada; 333 Department of Epidemiology, Division of Cancer Prevention and Population Science, The University of Texas MD Anderson Cancer Center, 1515 Holcombe Blvd, Houston, TX 77030 USA; 334 Magee-Womens Hospital, University of Pittsburgh School of Medicine, 300 Halket St, Pittsburgh, PA 15213 USA; 335 Center for Genomic Medicine and Department of Anasthesia, Massachusetts General Hospital, Boston, MA 02114 USA; 336 Human Genetics, Graduate School of Public Health, University of Pittsburgh, UPMC Cancer Pavilion, Suite 4C, Office # 467, 5150 Centre Avenue, Pittsburgh, PA 15232 USA; 337 UPMC Hillman Cancer Center, Pittsburgh, 15232 PA USA; 338 Genetic Cancer Susceptibility Group, International Agency for Research on Cancer, 150 cours Albert Thomas, 69008 Lyon, France; 339 Oncogenetics Team, The Institute of Cancer Research and Royal Marsden NHS Foundation Trust, Downs Road, Sutton, SM2 5NG UK; 340 Genomics Center, Centre Hospitalier Universitaire de Québec - Université Laval Research Center, 2705 Laurier Boulevard, Québec City, QC G1V4G2 Canada; 341 UCLA Path and Lab Med, University of California, 10833 Le Conte Ave, Los Angeles, CA 190095 USA; 342 Department of Medicine, Epidemiology Section, Institute for Clinical and Translational Research, Baylor Medical College, One Baylor Plaza, MS: BCM451, Suite 100D, Houston, TX 77030-3411 USA

**Keywords:** Cancer genetics, Genomics, Cancer, Epidemiology

## Abstract

Quantifying the genetic correlation between cancers can provide important insights into the mechanisms driving cancer etiology. Using genome-wide association study summary statistics across six cancer types based on a total of 296,215 cases and 301,319 controls of European ancestry, here we estimate the pair-wise genetic correlations between breast, colorectal, head/neck, lung, ovary and prostate cancer, and between cancers and 38 other diseases. We observed statistically significant genetic correlations between lung and head/neck cancer (*r*_*g*_ = 0.57, *p* = 4.6 × 10^−8^), breast and ovarian cancer (*r*_*g*_ = 0.24, *p* = 7 × 10^−5^), breast and lung cancer (*r*_*g*_ = 0.18, *p *=1.5 × 10^−6^) and breast and colorectal cancer (*r*_*g*_ = 0.15, *p* = 1.1 × 10^−4^). We also found that multiple cancers are genetically correlated with non-cancer traits including smoking, psychiatric diseases and metabolic characteristics. Functional enrichment analysis revealed a significant excess contribution of conserved and regulatory regions to cancer heritability. Our comprehensive analysis of cross-cancer heritability suggests that solid tumors arising across tissues share in part a common germline genetic basis.

## Abstract (web summary)

Similarities in cancers can be studied to interrogate their etiology. Here, the authors use genome-wide association study summary statistics from six cancer types based on 296,215 cases and 301,319 controls of European ancestry, showing that solid tumours arising from different tissues share a degree of common germline genetic basis.

## Introduction

Inherited genetic variation plays an important role in cancer etiology. Large twin studies have demonstrated an excess familial risk for cancer sites including, but not limited to, breast, colorectal, head/neck, lung, ovary, and prostate with heritability estimates ranging between 9% (head/neck) to 57% (prostate)^1–3^. Data from nation-wide and multi-generation registries further show that elevated cancer risks go beyond nuclear families and isolated types, as family history of a specific cancer can increase risk for other cancers^4–6^. Additional evidence for a shared genetic component have been demonstrated by cross-cancer genome-wide association study (GWAS) meta-analyses, which set out to identify genetic variants associated with more than one cancer type. Fehringer et al. studied breast, colorectal, lung, ovarian, and prostate cancer, and identified a novel locus at 1q22 associated with both breast and lung cancer^7^. Kar et al. focused on three hormone-related cancers (breast, ovarian, and prostate), and identified seven novel susceptibility loci shared by at least two cancers^8^.

Previous attempts to estimate the genetic correlation across cancers using GWAS data^9–12^ have mostly relied on restricted maximum likelihood (REML) implemented in GCTA (genome-wide complex trait analysis)^13^ and individual-level genotype data. However, these studies have had limited sample sizes, yielding inconclusive results. Sampson et al. quantified genetic correlations across 13 cancers in European ancestry populations and identified four cancer pairs with nominally significant genetic correlations (bladder–lung, testis–kidney, lymphoma–osteosarcoma, and lymphoma–leukemia)^9^. They did not observe any significant genetic correlations across common solid tumors including cancers of the breast, lung and prostate^9^. REML becomes computationally challenging for large sample sizes and is sensitive to technical artifacts. LD score regression (LDSC)^14,15^ overcomes these issues by leveraging the relationship between association statistics and LD patterns across the genome. We recently used cross-trait LDSC to quantify genetic correlations across six cancers based on a subset of the data included here and found moderate correlations between colorectal and pancreatic cancer, as well as between lung and colorectal cancer^16^. However, the average sample size was only 11,210 cases and 13,961 controls per cancer, resulting in imprecise estimates with wide confidence intervals.

In addition to the development of novel analytical methods tailored to genomic data, several high-quality functional annotations have recently been released into the public domain through large-scale efforts. For example, the ENCODE consortium has built a comprehensive and informative parts list of functional elements in the human genome (http://www.nature.com/encode/#/threads), which allows for the analysis of components of SNP-heritability to unravel the functional architecture of complex traits.

Here, we use summary statistics from the largest-to-date European ancestry GWAS of breast, colorectal, head/neck, lung, ovary, and prostate cancer with an average sample size of 49,369 cases and 50,219 controls per cancer, to quantify genetic correlations between cancers and their subtypes. We also use GWAS summary statistics for 38 non-cancer traits (average N = 113,808 per trait), to quantify the genetic correlations between the six cancers and other diseases. Furthermore, we assessed the proportion of cancer heritability attributable to specific functional categories, with the goal of identifying functional elements that are enriched for SNP-heritability.

Our comprehensive analysis identifies statistically significant genetic correlations between lung and head/neck cancer, breast and ovarian cancer, breast and lung cancer, and breast and colorectal cancer. We also find multiple cancers to be genetically correlated with non-cancer traits including smoking, psychiatric diseases, and metabolic traits. Functional enrichment analysis reveals a significant contribution of conserved and regulatory regions to cancer heritability. Our results suggest that solid tumors arising across tissues share in part a common germline genetic basis.

## Results

### Heritability estimates across cancers

**Fig. 1:**
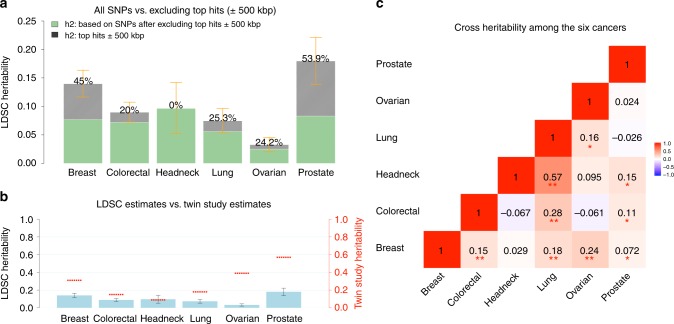
Estimates of SNP-heritability ($h_g^2$) and cross-cancer heritability (*r*_*g*_) for the six cancer types. SNP-heritability and cross-cancer heritability are calculated based on HapMap3 SNPs using LD score regression (LDSC). **a** The solid bar represents overall SNP $h_g^2$ on the liability scale, calculated based on all HapMap3 SNPs. The dark green bar represents $h_g^2$ calculated based on non-significant SNPs—the remaining SNPs after excluding genome-wide significant hits (*p* < 5 × 10^−8^) ± 500 kb. The black bar with density texture indicates proportion of $h_g^2$ (as reflected by the percentages displayed on top of each bar) that could be explained by top hits ±500 kb surrounded areas. The orange error bars represent 95% confidence intervals. **b** The solid blue bar represents overall SNP $h_g^2$ in liability scale (no SNP exclusion), with black error bars indicating 95% confidence intervals. The red short lines correspond to classical estimates of *h*^2^ measured in a twin study of Scandinavian countries (Mucci et al.^2^). **c** Genetic correlations between cancers. Estimates withstood Bonferroni corrections (*p* < 0.05/15) are marked with double asterisk (**), and nominal significant results (*p* < 0.05) are marked with single asterisk (*)

We first estimated cancer-specific heritability causally explained by common SNPs ($h_g^2$) using LDSC (note that this quantity is slightly different from the $h_g^2$ as defined in Yang et al.^17^ which estimates the heritability due to genotyped and imputed SNPs) (see Methods). Estimates of $h_g^2$ on the liability scale ranged from 0.03 (ovarian) to 0.25 (prostate) (Supplementary Table 1). After removing genome-wide significant (*p* < 5 × 10^−8^) loci, defined as all SNPs within 500 kb of the most significant SNP in a given region (Supplementary Data 1), we observed an ~50% decrease in SNP-heritability for prostate and breast cancer, and ~20% decrease for lung, ovarian, and colorectal cancer, despite the fact that we were only excluding 1% (colorectal cancer) to 5% (breast cancer) of the genome. In contrast, the SNP-heritability for head/neck cancer was not affected by removing genome-wide significant loci (Fig. 1a). For most of the cancers, the GWAS significant loci for that particular cancer explained most of the heritability. For some cancers, however, significant GWAS loci of other cancers also explained a non-trivial part of its heritability. For example, the significant breast cancer GWAS loci explained 10%, 15%, and 22% heritability of colorectal, ovarian and prostate cancer, respectively; the significant colorectal cancer GWAS loci explained 11% heritability of prostate cancer; the significant lung cancer GWAS loci explained 10% heritability of head/neck cancer; and the significant prostate cancer GWAS loci explained 11 and 15% heritability of breast and ovarian cancer, respectively (Supplementary Table 2). Comparing the liability-scale SNP-heritability to corresponding estimates from twin studies suggests that common SNPs can almost entirely explain the classical heritability of head/neck cancer, whereas for other cancers, only 30–40% of heritability can be explained (Fig. 1b).

### Genetic correlations between cancers

We then estimated the genetic correlation between cancers using cross-trait LDSC (see Methods). After adjusting for the number of tests (*p* < 0.05/15 = 0.003), we found multiple significant genetic correlations Fig. 1c and Supplementary Table 1), with the strongest result observed for lung and head/neck cancer (*r*_*g*_ = 0.57, se = 0.10). In addition, colorectal and lung cancer (*r*_*g*_ = 0.28, se = 0.06), breast and ovarian cancer (*r*_*g*_ = 0.24, se=0.06), breast and lung cancer (*r*_*g*_ = 0.18, se = 0.04), and breast and colorectal cancer (*r*_*g*_ = 0.15, se = 0.04) showed statistically significant genetic correlations. We also observed nominally significant genetic correlations (*p* < 0.05) between lung and ovarian cancer (*r*_*g*_ = 0.16, se = 0.08), prostate cancer and head/neck (*r*_*g*_ = 0.15, se = 0.08), colorectal (*r*_*g*_ = 0.11, se = 0.05), and breast cancer (*r*_*g*_ = 0.07, se = 0.03) (Fig. 1c). Some cancer pairs showed minimal correlations with estimates close to 0 (ovarian and prostate: *r*_*g*_ = 0.02, se = 0.07; lung and prostate: *r*_*g*_ = −0.03, se = 0.04; breast and head/neck: *r*_*g*_ = 0.03, se = 0.06). We further calculated the cross-cancer genetic correlation based on data after excluding the GWAS significant regions of each cancer. The estimates were mostly consistent with the results calculated based on all SNPs.

We conducted subtype-specific analysis for breast, lung, ovarian, and prostate cancer (Supplementary Table 1). Estrogen receptor positive (ER+) and negative (ER−) breast cancer showed a genetic correlation of 0.60 (se = 0.03), indicating that the genetic contributions to these two subtypes are in part distinct. The genetic correlation between the two common lung cancer subtypes adenocarcinoma and squamous cell carcinoma was similarly 0.58 (se = 0.10). Further, we observed a significantly larger genetic correlation of lung cancer with ER− (*r*_*g*_ = 0.29, se = 0.06) than with ER + breast cancer (*r*_*g*_ = 0.13, se = 0.04) (*p*_difference_ = 0.002). This also held true for lung squamous cell carcinoma, which showed statistically stronger genetic correlation with ER− (*r*_*g*_ = 0.33, se = 0.08) than with ER + breast cancer (*r*_*g*_ = 0.11, se = 0.05) (*p*_difference_ = 0.0019). We observed no other statistically significant differential genetic correlations across subtypes (all *p*_difference_ > 0.1).

**Fig. 2:**
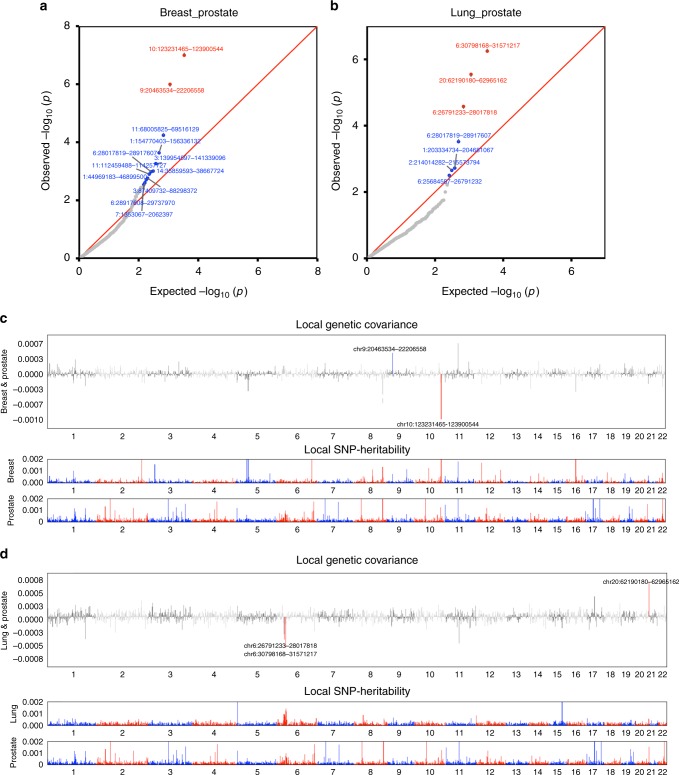
Local genetic correlation between breast, lung and prostate cancer. The region-specific *p*-values for the local genetic covariance for breast and prostate cancer are shown in **a**, and for lung and prostate cancer in **b**. Each dot presents a specific genomic region. In the QQ plots, red color indicates significance after multiple corrections (*p* < 0.05/1703 regions compared), and blue color indicates nominal significance (*p* < 0.05/15 pairs of cancers compared). Manhattan-style plots showing the estimates of local genetic covariance for breast and prostate cancer (**c**), and for lung and prostate cancer (**d**). Although breast and prostate cancer only show modest genome-wide genetic correlation, two loci exhibit significant local genetic covariance. Similarly, albeit the negligible overall genetic correlation for lung and prostate cancer, three loci present significant local genetic covariance. In the Manhattan plots, red color indicates even number chromosomes and blue color indicates odd number chromosomes

We then estimated local genetic correlations between cancers using ρ-HESS, dividing the genome into 1703 regions (see Methods) (Fig. 2 and Supplementary Fig. 1). We found that although the genome-wide genetic correlation between breast and prostate cancer was modest (*r*_*g*_ = 0.07), chr10:123M (10q26.13, *p* = 1.0 × 10^−7^) and chr9:20–22 M (9p21, *p* = 1.0 × 10^−6^), two previously known pleiotropic regions^18^, showed significant genetic correlations (*r*_*g*_ = −0.00098 and *r*_*g*_ = 0.00046). Similarly, although the genome-wide genetic correlation between lung and prostate cancer was negligible (*r*_*g*_ = −0.03), two previously identified pleiotropic regions (chr6:30–31 M or 6p21.33, *p* = 5.7 × 10^−7^ and chr20:62M or 20q13.33, *p* = 2.8 × 10^−6^) exhibited significant local genetic correlations (*r*_*g*_ = −0.00060 and *r*_*g*_ = 0.00067). Overall, local genetic correlation analysis reinforced shared effects for 44% (31/71) of previously reported pleiotropic cancer regions (Supplementary Data 2). It also identified novel pleiotropic signals. For example, the breast and prostate cancer pleiotropic region at 2q33.1 showed significant local genetic correlation between breast and ovarian cancer (*p* = 2.3 × 10^−6^). Additionally, 6p21.32, a region indicated for head/neck and prostate cancer, showed highly significant local genetic correlation for head/neck and lung cancer (*p* = 8.6 × 10^−8^).

### Genetic correlations between cancer and other traits

**Fig. 3:**
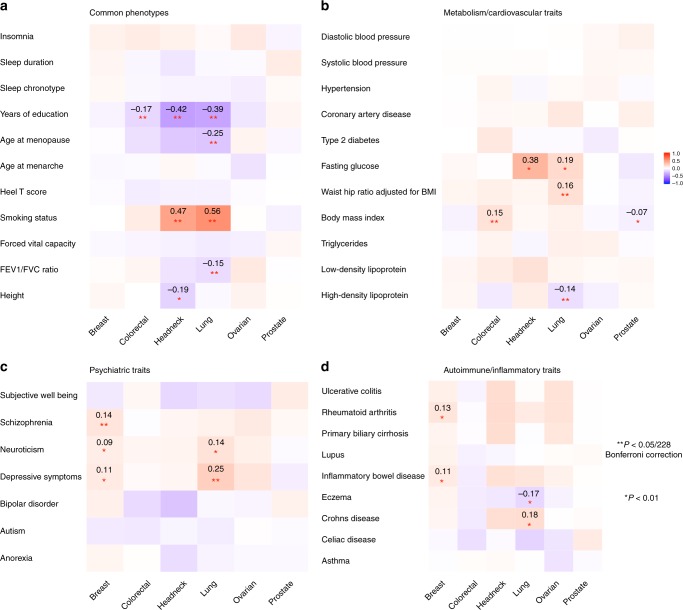
Cross-trait genetic correlation (*r*_*g*_) analysis between cancers and non-cancer traits. The traits were divided into four categories: **a** Common phenotypes, **b** Metabolic or cardiovascular related traits, **c** Psychiatric traits, **d** Autoimmune inflammatory diseases. Pair-wise genetic correlations withstood Bonferroni corrections (228 tests) are marked with double asterisk (**), with estimates of correlation shown in the cells. Pair-wise genetic correlations with significance at *p* < 0.01 are marked with a single asterisk (*). The color of cells represents the magnitude of correlation

Significant genetic correlations (*p* < 0.05/228 = 0.0002) between the six cancers and 38 non-cancer traits reflected several known associations (Fig. 3 and Supplementary Data 3). We observed a strong genetic correlation between smoking and lung cancer (*r*_*g*_ = 0.56, se = 0.06), and similarly for head/neck cancer (*r*_*g*_ = 0.47, se = 0.08), both cancers having smoking as its primary risk factor^19,20^. Educational attainment was negatively genetically correlated with colorectal (*r*_*g*_ = −0.17, se = 0.04), head/neck (*r*_*g*_ = −0.42, se = 0.07), and lung cancer (*r*_*g*_ = −0.39, se=0.04) (all *p* < 5 × 10^−6^). Body mass index (BMI) showed a positive genetic correlation with colorectal cancer (*r*_*g*_ = 0.15, se = 0.03) and also suggestive but weak negative correlations with prostate (*r*_*g*_ = −0.07, se = 0.03) and breast cancer (*r*_*g*_ = −0.06, se = 0.03). Lung cancer showed a negative genetic correlation with lung function (*r*_*g*_ = −0.15, se = 0.04) and age at natural menopause (*r*_*g*_ = −0.25, se = 0.05), and moderate positive genetic correlations with depressive symptoms (*r*_*g*_ = 0.25, se=0.06) and waist-to-hip ratio (*r*_*g*_ = 0.16, se = 0.04). Breast cancer showed a positive genetic correlation with schizophrenia (*r*_*g*_ = 0.14, se = 0.03).

We did not find evidence of genetic correlations between cancer and several previously suggested risk factors^21–23^ including cardiovascular traits (coronary artery disease, hypertension, and blood pressure) or sleep characteristics (chronotype, duration, and insomnia). Further, we did not observe genetic correlations between cancer and circulating lipids (HDL, LDL, and triglycerides) or type 2 diabetes-related traits except a significant negative correlation between HDL and lung cancer (*r*_*g*_ = −0.14, se = 0.04). We observed no significant genetic correlation between breast cancer and age at menarche (*r*_*g*_ = −0.03, se = 0.03) or age at natural menopause (*r*_*g*_ = −0.01, se = 0.03). We also did not observe notable genetic correlations between cancer and autoimmune inflammatory diseases or height.

Subtype analysis revealed that smoking and educational attainment showed genetic correlations with all lung cancer subtypes (Supplementary Data 3). Educational attainment, forced vital capacity and depressive symptoms showed genetic correlations with ER− but not ER + breast cancer, whilst the observed genetic correlation between schizophrenia and breast cancer was limited to ER + disease, and the genetic correlation between depressive symptoms and lung cancer was observed only for lung squamous cell carcinoma.

**Fig. 4:**
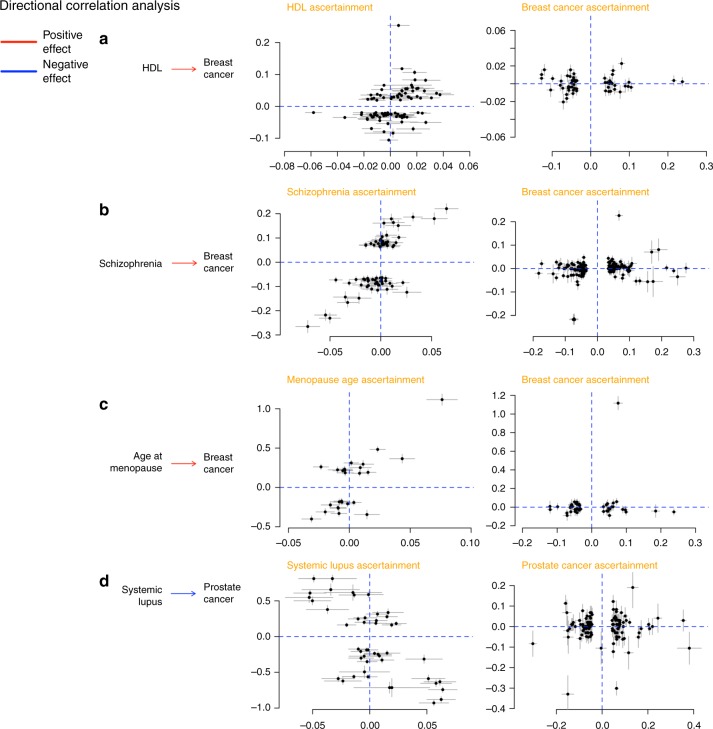
Putative directional relationships between cancers and traits. For each cancer–trait pair identified as candidates to be related in a causal manner, the plots show trait-specific effect sizes (beta coefficients) of the included genetic variants. Gray lines represent the relevant standard errors. **a** HDL and breast cancer. Trait-specific effect sizes for HDL and breast cancer are shown for SNPs associated with HDL levels (left) and breast cancer (right). **b** Schizophrenia and breast cancer. Trait-specific effect sizes for schizophrenia and breast cancer are shown for SNPs associated with schizophrenia (left) and breast cancer (right). **c** Age at natural menopause and breast cancer. Trait-specific effect sizes for age at natural menopause and breast cancer are shown for SNPs associated with age at natural menopause (left) and breast cancer (right). **d** Lupus and prostate cancer. Trait-specific effect sizes for lupus and prostate cancer are shown for SNPs associated with lupus (left) and prostate cancer (right)

We further assessed the support for mediated or pleiotropic causal models for non-cancer traits and cancer using the correlation between trait-specific effect sizes of genome-wide significant SNPs for pairs of phenotypes. We detected four putative directional genetic correlations (defined as *p* < 0.05 from a likelihood ratio (LR) comparing the best non-causal model to the best causal model) (Fig. 4), where SNPs associated with the non-cancer trait showed correlated effect estimates with cancer but the reverse was not true (circulating HDL concentrations and breast cancer, LR_non-causal vs. causal_ = 0.04, schizophrenia and breast cancer, LR_non-causal vs. causal_ = 0.003, age at natural menopause and breast cancer, LR_non-causal vs. causal_ = 0.04, and lupus and prostate cancer, LR_non-causal vs. causal_ = 0.0006).

### Functional enrichment analysis of cancer heritability

**Fig. 5:**
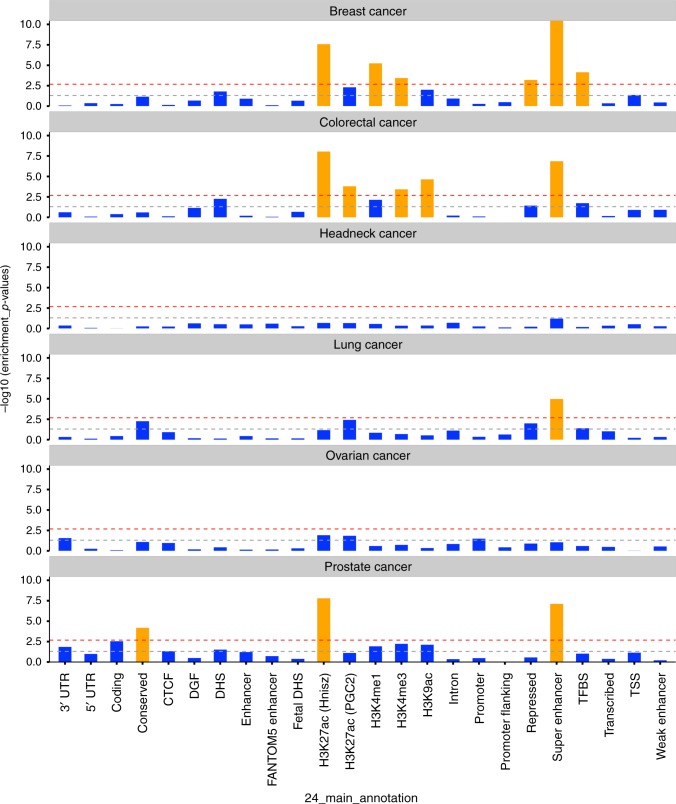
Enrichment *p*-values of 24 non-cell-type-specific functional categories over six cancer types. The *x*-axis represents each of the 24 functional categories, *y*-axis represents log-transformed *p*-values of enrichment. Annotations with statistical significance after Bonferroni corrections (*p* < 0.05/24) were plotted in orange, otherwise blue. The horizontal gray dash line indicates *p*-threshold of 0.05; horizontal red dash line indicates *p*-threshold of 0.05/24. From top to bottom are six panels representing six cancers: breast cancer, colorectal cancer, head/neck cancer, lung cancer, ovarian cancer, and prostate cancer. TSS transcription start site, UTR untranslated region, TFBS transcription factor binding sites, DHS DNase I hypersensitive sites, DGF digital genomic foot printing, CTCF CCCTC-binding factor

Finally, we partitioned SNP-heritability of each cancer by using 24 genomic functional annotations (the baseline-LD model described in Gazal et al.^24^) and 220 cell-type-specific histone mark annotations (the cell-type-specific model described in Finucane et al.^14^). Meta-analysis across the six cancers revealed statistically significant enrichments for multiple functional categories. We observed the highest enrichment for conserved regions (Table 1, Supplementary Table 3) which overlapped with only 2.6% of SNPs but explained 25% of cancer SNP-heritability (9.8-fold enrichment, *p* = 2.3 × 10^−5^). Transcription factor binding sites showed the second highest enrichment (4.0-fold, 13% of SNPs explaining 40% of SNP-heritability, *p* = 1.4 × 10^−7^). Further, super-enhancers (groups of putative enhancers in close genomic proximity with unusually high levels of mediator binding) showed a significant 2.6-fold enrichment (*p* = 2.0 × 10^−20^). Additional enhancers, including regular enhancers (3.2-fold), weak enhancers (3.1-fold) and FANTOM5 enhancers (3.1-fold), presented similar enrichments but were not statistically significant. In addition, multiple histone modifications of epigenetic markers H3K9ac, H3K4me3, and H3K27ac, were all significantly enriched for cancer heritability. Repressed regions exhibited depletion (0.34-fold, *p* = 1.2 × 10^−6^). Enrichment analysis of functional categories for each cancer and cancer subtype are shown in Fig. 5 and Supplementary Table 4.

**Table 1:** Significant enrichment estimates of genomic functional categories, meta-analyzed across six cancer sites

Category	Enrichment (95% CI)	*P*-value
Conserved region	9.78 (5.72–13.84)	2.28 × 10^−5^
TFBS	4.04 (2.91–5.17)	1.43 × 10^−7^
H3K9ac	3.41 (2.14–4.69)	2.04 × 10^−4^
H3K4me3	3.23 (2.47–4.00)	8.91 × 10^−9^
Super Enhancer	2.56 (2.23–2.89)	1.99 × 10^−20^
H3K27ac (PGC)	2.36 (1.91–2.80)	2.12 × 10^−9^
H3K27ac (Hnisz)	1.90 (1.65–2.15)	1.86 × 10^−12^
H3K4me1	1.84 (1.56–2.12)	2.57 × 10^−9^
Repressed region	0.34 (0.07–0.61)	1.15 × 10^−6^

The meta-analysis was performed based on the enrichment estimates and standard errors calculated using LD score regression in each individual cancer type. *P*-values were significant after Bonferroni correction (*P* < 0.05/24)

*TFBS* transcription factor binding sites

Overall, cell-type-specific analysis of histone marks identified significant enrichments specific to individual cancers (Supplementary Fig. 2). For breast cancer, 3 out of 8 statistically significant tissues were adipose nuclei (H3K4me1, H3K9ac) and breast myoepithelial (H3K4me1) cells. For colorectal cancer, 15 out of the 18 statistically significant enrichments were observed in either colon or rectal tissues (colon/rectal mucosa, duodenum mucosa, small/large intestine, and colon smooth muscle). We observed no significant enrichments for head/neck, lung, and ovarian cancer, but we noted that for both lung (9 out of 10) and ovarian cancer (6 out of 10), the most enriched cell types were immune cells; while in head/neck cancer, 6 out of 10 most highly enriched cell types belonged to CNS (Supplementary Fig. 3, Supplementary Data 4). Cell-type-specific analysis for cancer subtypes are shown in Supplementary Data 5. Comparing cell-type-specific enrichment for cancers to the additional 38 non-cancer traits revealed notably differential clustering patterns (Supplementary Fig. 4). Breast, colorectal, and prostate cancer showed enrichment mostly for adipose and epithelial tissues, in contrast to autoimmune diseases (enriched for immune/hematopoietic cells) or psychiatric disorders (enriched for brain tissues).

## Discussion

We performed a comprehensive analysis quantifying the heritability and genetic correlation of six cancers, leveraging summary statistics from the largest cancer GWAS conducted to date. Our study demonstrates shared genetic components across multiple cancer types. These results contrast with a prior study conducted by Sampson et al. which reported an overall negligible genetic correlation among common solid tumors^9^. Our results are, however, in line with a recent study,^16^ which analyzed a subset of the data included here, and identified a significant genetic correlation between lung and colorectal cancer.

Our data support, and for the first time quantify, the strong genetic correlation (*r*_*g*_ = 0.57) between lung and head/neck cancer, two cancers linked to tobacco use^20,25^. We also for the first time observed a significant genetic correlation between breast and ovarian cancer (*r*_*g*_ = 0.24), two cancers that are known to share rare genetic factors including *BRCA1*/*2* mutations, and environmental exposures associated with endogenous and exogenous hormone exposures^26^. Prostate cancer is also considered as hormone-dependent and associated with *BRCA1/2* mutations, but interestingly, we only observed a nominally significant and modest (*r*_*g*_ = 0.07) genetic correlation between breast and prostate cancer, while ovarian and prostate cancer showed no genetic correlation (*r*_*g*_ = 0.02, se = 0.07).

Our large sample sizes allowed us to conduct well-powered analyses for cancer subtypes. While head/neck cancer showed negligible genetic correlation with overall (*r*_*g*_ = 0.03, se = 0.06) and ER + breast cancer (*r*_*g*_ = −0.02, se = 0.07), it showed a stronger genetic correlation with ER− breast cancer (*r*_*g*_ = 0.21, se = 0.09). Similarly, lung cancer showed a statistically more pronounced genetic correlation with ER− (*r*_*g*_ = 0.29, se = 0.06) than ER + breast cancer (*r*_*g*_ = 0.13, se = 0.04). A recent pooled analysis of smoking and breast cancer risk demonstrated a smoking-related increased risk for ER + but not for ER− breast cancer^27^, and thus it is unlikely that the stronger genetic correlation between ER− subtype and lung and head/neck cancer is due to smoking behavior. Perhaps surprisingly, despite literature suggesting substantial similarities between ER− breast cancer and serous ovarian cancer in particular^28^, we did not observe statistically significant different genetic correlations between ER− or ER + breast cancer and serous ovarian cancer (*r*_*g*_ = 0.17, se = 0.08 *vs. r*_*g*_ = 0.11, se = 0.06). This suggests that rare high penetrance variants may play a more important role in driving the similarities behind ER− breast cancer and serous ovarian cancer than common genetic variation.

Heritability analysis confirms that common cancers have a polygenic component that involves a large number of variants. Although susceptibility variants identified at genome-wide significance explain an appreciable fraction of the heritability for some cancers, we estimate that the majority of the polygenic effect is attributable to other, yet undiscovered variants, presumably with effects that are too weak to have been identified with current sample sizes. We found the genetic component that could be attributed to genome-wide significant loci varied greatly from ~0% for head/neck cancer to ~50% for breast and prostate cancer. These results reflect in part the strong correlation between number of GWAS-identified loci and sample size, as we had more than twice as many breast and prostate cancer samples compared to the other cancers. One corollary is that larger GWAS are likely to identify new susceptibility loci that could help our understanding of disease development, improve prediction power of genetic risk scores and hence contribute to screening and personalized risk prediction^29^.

Among the genetic correlations between cancer and non-cancer traits, we observed positive correlations for psychiatric disorders (depressive symptoms, schizophrenia) with lung and breast cancer, where findings from epidemiological studies have been suggestive but inconclusive. It has been proposed that the linkage between psychiatric traits and cancers are more likely to be mediated through cancer-associated risk phenotypes such as smoking, excessive alcohol consumption in depressed populations^30^, and reduced fertility patterns (e.g., nulliparous) in psychiatric populations^31^. Detailed analyses considering confounding traits like reproductive history and smoking are needed to make inference about the mechanisms involved. GWAS have identified pleiotropic regions influencing both lung cancer and nicotine dependence, such as 15q25.1^32,33^. In line with those results, we identified a strong genetic correlation between smoking and both lung (*r*_*g*_ = 0.56) and head/neck cancer (*r*_*g*_ = 0.47). It remains unclear whether this genetic correlation is completely explained by the direct influence of smoking or if the shared genetic component affects the traits through separate pathways. Interestingly, a genetic correlation (*r*_*g*_ = 0.35, se = 0.14) between lung and bladder cancer, another smoking-associated cancer, has been identified previously^9^. Due to the small numbers of GWAS-identified smoking-associated SNPs, we were unable to assess a directional correlation between smoking and cancer, but we expect such analyses to become feasible as additional smoking-related SNPs are identified. We found modest positive, yet significant genetic correlations between adiposity-related measures (as reflected by waist-to-hip ratio, circulating HDL levels and BMI) and both colorectal and lung cancer, but negative genetic correlations between BMI and prostate and breast cancer, consistent with previous reported findings^34^ and reinforce the complex dynamics between obesity and cancer where multiple factors including age, smoking, endogenous hormones and reproductive status play a role.

We did not observe genetic correlations between breast cancer and age at menarche or age at natural menopause. These null observations were largely driven by ER + breast cancer (ER + : *r*_*g*_ = 0.006, se = 0.03 vs. ER−: *r*_*g*_ = −0.09, se = 0.04 for age at menarche. ER + : *r*_*g*_ = 0.0005, se = 0.04 vs. ER−: *r*_*g*_ = −0.10, se = 0.05 for age at natural menopause), and were unexpected given that both factors play pivotal roles in breast cancer etiology^35^ and previous Mendelian randomization (MR) analyses have identified a link^36,37^. An important difference between genetic correlation and MR analyses is that the latter only considers genome-wide significant SNPs while the former incorporates the entire genome. It is possible that a relatively small overlap in strongly associated SNPs can result in significant MR results despite low evidence of an overall genetic correlation. Indeed, the directional genetic correlations we observed for age at natural menopause, schizophrenia, and HDL with breast cancer, and for lupus with prostate cancer, highlight again that although an overall genetic correlation may be negligible, there can still be genetic links between traits. It is important to note that we cannot rule out unmeasured confounding, including the possibility that these genetic variants affect an intermediate phenotype that is pleiotropic for both target traits. Given the observational nature of our data, these putative causal directions should be interpreted with caution.

Pan-cancer tumor-based studies have demonstrated that different cancers are sometimes driven by similar somatic functional events such as specific copy number abnormalities and mutations^38,39^. Our enrichment results of germline genetic across functional annotation data shed new light on the biological mechanisms leading to cancer development. The more pronounced enrichment identified for conserved regions compared with coding regions provides evidence for the biological importance of the former, which has been shown to be true for multiple traits^14,40^. Even though the biochemical function of many conserved regions remains uncharacterized, transcribed ultra-conserved regions have been found to be frequently located at fragile sites. Compared to normal cells, cancer cells have a unique spectrum of transcribed ultra-conservative regions, suggesting that variation in expression of these regions are involved in the malignant process^41,42^. These results bridge the link between germline and somatic genetics in cancer development, which was also observed in a recent breast cancer GWAS that has demonstrated a strong overlap between target genes for GWAS hits and somatic driver genes in breast tumors^43^. We also found a four-fold enrichment for transcription factor binding sites and a three-fold enrichment for super-enhancers, consistent with prior observations that breast cancer GWAS loci fall in enhancer regions involved in distal regulation of target genes^43^. Cell-type-specific analysis of histone marks demonstrated the importance of tissue specificity, primarily for colorectal and breast cancer. Further, our results suggest that immune cells are important for ovarian and lung cancer whilst CNS is important to head/neck cancer. Unfortunately, we did not have data on prostate-specific tissues, but we note that tissue-specific enrichment of prostate cancer heritability for epigenetic markers has been observed previously^10^. We note that generation of rich functional annotation is ongoing and we expect to include additional tissue-specific functional elements in our future work.

Our study has several strengths. We were able to robustly quantify pair-wise genetic correlations between multiple cancers using the largest available cancer GWAS, comprising almost 600,000 samples across six major cancers and subtypes. We were also able to systematically assess the genetic correlations between cancer and 38 non-cancer traits. Notwithstanding the large sample sizes, several limitations need to be acknowledged. We did not have the sample sizes required to assess relevant cancer subgroups including oropharyngeal cancer, clear cell, mucinous and endometrioid ovarian cancer, or lung cancer among never smokers (each with ~2000 cases). In addition, we did not have access to GWAS summary statistics for pre- vs. post-menopausal breast cancer. We were not able to consider all cancer risk factors when selecting non-cancer traits, since some of the well-established risk factors such as infection were either not available, showed no evidence of heritability or were not based on adequate sample sizes for robust analyses. SNP-heritability varies with minor allele frequency, linkage disequilibrium, and genotype certainty; we note that approaches to estimate heritability leveraging GWAS data are constantly evolving. We also note that estimate variability needs to be taken into account when comparing the SNP-heritability with the classical twin-heritability, in particular for cancers with small sample sizes such as head/neck cancer (SNP-heritability varied between 5–14% and twin-heritability varied between 0–60%, although both point estimates were 9%). Further, our data were based on GWAS meta-analysis from multiple individual GWAS across European ancestry populations from Europe, Australia and the US. Intra-European ancestry differences are likely to be a source of bias. However, since we limited our analysis to SNPs with MAF > 1% and HapMap3 SNPs (which have proven to be well imputed across European ancestry populations), we believe that any population structure across cancers will have minimal effect on our results. Finally, as more non-European and multi-ethnic GWAS data become available, it is important to examine trans-ethnic genetic correlation in cancer.

In conclusion, results from our comprehensive analysis of heritability and genetic correlations across six cancer types indicate that solid tumors arising from different tissues share common germline genetic influences. Our results also demonstrate evidence for common genetic risk sharing between cancers and smoking, psychiatric, and metabolic traits. In addition, functional components of the genome, particularly conserved and regulatory regions, are significant contributors to cancer heritability across multiple cancer types. Our results provide a basis and direction for future cross-cancer studies aiming to further explore the biological mechanisms underlying cancer development.

## Methods

### Studies and quality control

We used summary statistics from six cancer GWASs based on a total of 597,534 participants of European ancestry. Cancer-specific sample sizes were: breast cancer: 122,977 cases/105,974 controls; colorectal cancer: 36,948/30,864; head/neck cancer (oral and oropharyngeal cancers): 5452/5984; lung cancer: 29,266/56,450; ovarian cancer: 22,406/40,941; prostate cancer: 79,166/61,106. These data were generated through the joint efforts of multiple consortia. Details on study characteristics and subjects contributed to each cancer-specific GWAS summary dataset have been described elsewhere^43–49^. SNPs were imputed to the 1000 Genomes Project reference panel (1KGP) using a standardized protocol for all cancer types^18^. We included autosomal SNPs with a minor allele frequency (MAF) larger than 1% and present in HapMap3 (N_SNPs_ = ~1 million) because those SNPs are usually well imputed in most studies (note that excluding sex chromosomes could reduce the overall heritability estimates). A brief overview of the quality control in each cancer dataset are presented in Supplementary Table 5. For some of the cancers, we further obtained summary statistics data on subtypes (ER + and ER− breast cancer; lung adenocarcinoma, and squamous cell carcinoma; serous invasive ovarian cancer and advanced stage prostate cancer, defined as metastatic disease or Gleason score ≥ 8 or PSA > 100 or prostate cancer death). Sample sizes and more details shown in Supplementary Table 1.

We additionally assembled European ancestry GWAS summary statistics from 38 traits, which spanned a wide range of phenotypes including anthropometric (e.g., height and body mass index (BMI)), psychiatric disorder (e.g., depressive symptoms and schizophrenia), and autoimmune disease (e.g., rheumatoid arthritis and celiac disease) (Supplementary Table 6). We calculated trait-specific SNP-heritability and restricted our analysis to traits with a heritable component (Supplementary Table 7)^14^. We removed the major histocompatibility complex (MHC) region from all analysis because of its unusual LD and genetic architecture.

### Estimation of SNP-heritability and genetic correlation

We estimated the SNP-heritability due to genotyped and imputed SNPs ($h_g^2$, the proportion of phenotypic variance causally explained by common SNPs) of each cancer using LDSC^15^. Briefly, this method is based on the relationship between LD score and *χ*^2^-statistics:

1 $$E\left[ {\chi _j^2} \right] \approx \frac{{N_jh_g^2}}{M}l_j + 1$$

where $E\left[ {\chi _j^2} \right]$ denotes the expected *χ*^2^-statistics for the association between the outcome and SNP *j*, *N*_*j*_ is the study sample size available for SNP *j*, *M* is the total numbers of variants and *l*_*j*_ denotes the LD score of SNP *j* defined as $l_j = \mathop {\sum }\limits_k r^2\left( {j,k} \right)$ (*k* denotes other variants within the LD region). Note that the quantity estimated by LDSC is the causal heritability of common SNPs, which is different from the SNP-heritability as defined in Yang et al.^17^. To estimate $h_g^2$ attributable to undiscovered loci, we identified SNPs that were associated with a given cancer at genome-wide significance (*p* < 5 × 10^−8^) and removed all SNPs within (+/−) 500,000 base-pairs of those loci prior to calculation (number of regions (+/− 500 kb) for each cancer that reach the 5 × 10^−8^ threshold and measures of effect size are shown in Supplementary Data 1). We also converted the SNP-heritability from observed scale to liability scale by incorporating sample prevalence (*P*) and population prevalence (*F*) of each cancer:

2 $$h_{{\rm{liability}}}^{2} = h_{{\rm{observed}}}^{2}\frac{{F\left( {1 - F} \right)}}{{\phi \left( {{\Phi}^{ - 1}\left( F \right)} \right)^{2}}}\frac{{F\left( {1 - F} \right)}}{{P\left( {1 - P} \right)}}$$

We subsequently calculated the genome-wide genetic correlations (*r*_*g*_) between different cancers, and between cancers and non-cancer traits, using an algorithm^14^:

3 $$E\left[ {\beta _j\gamma _j} \right] = \frac{{\sqrt {N_1N_2} r_g}}{M}l_j + \frac{{N_sr}}{{\sqrt {N_1N_2} }}$$

where *β*_*j*_ and *γ*_*j*_ are the effect sizes of SNP *j* on traits 1 and 2, *r*_*g*_ is the genetic covariance, *M* is number of SNPs, *N*_1_ and *N*_2_ are the sample sizes for trait 1 and 2, *N*_*s*_ is the number of overlapping samples, *r* is the phenotypic correlation in overlapping samples and *l*_*j*_ is the LD score defined as above. For genetic correlation between 6 cancers, the significance level is 0.05/15 = 0.003; for genetic correlation between 6 cancers and 38 traits, the significance level is 0.05/(6 × 38) = 0.0002.

Overall genetic correlations as estimated by LDSC are based on aggregated information across all variants in the genome. It is possible that even though two traits show negligible overall genetic correlation, there are specific regions in the genome that contribute to both traits. We therefore examined local genetic correlations between cancer pairs using ρ-HESS^50^, an algorithm which partitions the whole genome into 1703 regions based on LD-pattern of European populations and quantifies correlation between pairs of traits due to genetic variation restricted to these genomic regions. Local genetic correlation was considered statistically significant if *p* < 0.05/1,703 = 2.9 × 10^−5^. In particular, we assessed the local genetic correlations for previously reported pleiotropic regions^18,51^ known to harbor SNPs affecting multiple cancers.

### Directional genetic correlation analysis

In addition to the genetic correlation analysis, which reflects overall genetic overlaps, we also attempted to identify directions of potential genetic correlations using a subset of SNPs as proposed by Pickrell et al.^52^. The method adopts the following assumption: if a trait X influences trait Y, then SNPs influencing X should also influence Y, and the SNP-specific effect sizes for the two traits should be correlated. Further, since Y does not influence X, but could be influenced by mechanisms independent of X, genetic variants that influence Y do not necessarily influence X. Based on this assumption, the method proposes two causal models and two non-causal models; and calculates the relative likelihood ratio (LR) of the best non-causal model compared to the best causal model. We determined significant SNPs for each given cancer or trait in two independent ways, (1) LD pruned SNPs: we selected genome-wide significant (*p* < 5 × 10^−8^) SNPs and pruned on LD-pattern in the European populations in Phase1 of 1KGP; (2) posterior probability of association (PPA) SNPs: we used a method implemented in fgwas^53^, which splits the genome into independent blocks based on LD patterns in 1KGP and estimates the prior probability that any block contains an association. The model outputs posterior probability that the region contains a variant that influences the trait. We selected the lead SNP from each of the regions with a PPA of at least 0.9. We scanned through all pairs of cancers and traits to identify directional correlations. Only pairs of traits with evidence of directional correlations (LR comparing the best non-causal model over the best causal model < 0.05) and without evidence of heteroscedasticity (pleiotropic effects)^54^ were reported as relatively more likely to exhibit mediated causation.

### Functional partitioning of SNP-heritability

To assess the importance of specific functional annotations in SNP-heritability across cancers, we partitioned the cancer-specific heritability using stratified-LDSC^14^. This method partitions SNPs into functional categories and calculates category-specific enrichments based on the assumption that a category of SNPs is enriched for heritability if SNPs with high LD to that category have higher *χ*^2^ statistics than SNPs with low LD to that category. The analysis was performed using two models^14,24^.

1. A full baseline-LD model including 24 publicly available annotations that are not specific to any cell type. When performing this model, we adjusted for MAF via MAF-stratified quantile-normalized LD score, and other LD-related annotations such as predicted allele age and recombination rate, as implemented by Gazal et al.^24^. Briefly, the 24 annotations included coding, 3′UTR and 5′UTR, promoter and intronic regions, obtained from UCSC Genome Browser and post-processed by Gusev et al.^55^; the histone marks mono-methylation (H3K4me1) and tri-methylation of histone H3 at lysine 4 (H3K4me3), acetylation of histone H3 at lysine 9 (H3K9ac) processed by Trynka et al.^56–58^ and two versions of acetylation of histone H3 at lysine 27 (H3K27ac, one version processed by Hnisz et al.^59^, another used by the Psychiatric Genomics Consortium (PGC)^60^); open chromatin, as reflected by DNase I hypersensitivity sites (DHSs and fetal DHSs)^55^, obtained as a combination of ENCODE and Roadmap Epigenomics data, processed by Trynka et al.^58^; combined chromHMM and Segway predictions obtained from Hoffman et al.^61^, which make use of many annotations to produce a single partition of the genome into seven underlying chromatin states (The CCCTC-binding factor (CTCF), promoter-flanking, transcribed, transcription start site (TSS), strong enhancer, weak enhancer categories, and the repressed category); regions that are conserved in mammals, obtained from Lindblad-Toh et al.^40^ and post-processed by Ward and Kellis^62^; super-enhancers, which are large clusters of highly active enhancers, obtained from Hnisz et al.^59^; FANTOM5 enhancers with balanced bi-directional capped transcripts identified using cap analysis of gene expression in the FANTOM5 panel of samples, obtained from Andersson et al.^63^; digital genomic footprint (DGF) and transcription factor binding site (TFBS) annotations obtained from ENCODE and post-processed by Gusev et al.^55^
2. In addition to the baseline-LD model, we also performed analyses using 220 cell-type-specific annotations for the four histone marks H3K4me1, H3K4me3, H3K9ac, and H3K27ac. Each cell-type-specific annotation corresponds to a histone mark in a single cell type (for example, H3K27ac in CD19 immune cells), and there were 220 such annotations in total. We further divided these 220 cell-type-specific annotations into 10 groups (adrenal and pancreas, central nervous system (CNS), cardiovascular, connective and bone, gastrointestinal, immune and hematopoietic, kidney, liver, skeletal muscle, and other) by taking a union of the cell-type-specific annotations within each group (for example, SNPs with any of the four histone modifications in any hematopoietic and immune cells were considered as one big category). When generating the cell-type-specific models, we added annotations individually to the baseline model, creating 220 separate models.

We performed a random-effects meta-analysis of the proportion of heritability over six cancers for each functional category. We set significance thresholds for individual annotations at *p* < 0.05/24 for baseline model and at *p* < 0.05/220 for cell-type-specific annotation.

## Supplementary information

Supplementary Information

Description of Additional Supplementary Files

Supplementary Data 1

Supplementary Data 2

Supplementary Data 3

Supplementary Data 4

Supplementary Data 5

Reporting Summary

Peer Review Files

## Data Availability

The datasets generated during and/or analyzed during the current study are available from the authors on request. Breast cancer: summary results for all variants are available at http://bcac.ccge.medschl.cam.ac.uk/. Requests for further data should be made through the Data Access Coordination Committee (http://bcac.ccge.medschl.cam.ac.uk/). Ovarian cancer: summary results are available from the Ovarian Cancer Association Consortium (OCAC) (http://ocac.ccge.medschl.cam.ac.uk/). Requests for further data can be made to the Data Access Coordination Committee (http://cimba.ccge.medschl.cam.ac.uk/). Prostate cancer: summary results are publicly available at the PRACTICAL website (http://practical.icr.ac.uk/blog/). Lung cancer: genotype data for lung cancer are available at the database of Genotypes and Phenotypes (dbGaP) under accession phs001273.v1.p1. Readers interested in obtaining a copy of the original data can do so by completing the proposal request form at http://oncoarray.dartmouth.edu/. Head/neck cancer: genotype data for the oral and pharyngeal OncoArray study have been deposited at the database of Genotypes and Phenotypes (dbGaP) under accession phs001202.v1.p1. Colorectal cancer: genotype data have been deposited at the database of Genotypes and Phenotypes (dbGaP) under accession number phs001415.v1.p1 and phs001078.v1.p1.
