# Publisher Correction: Shared heritability and functional enrichment across six solid cancers

**DOI:** 10.1038/s41467-019-12095-8

**Published:** 2019-09-23

**Authors:** Xia Jiang, Hilary K. Finucane, Fredrick R. Schumacher, Stephanie L. Schmit, Jonathan P. Tyrer, Younghun Han, Kyriaki Michailidou, Corina Lesseur, Karoline B. Kuchenbaecker, Joe Dennis, David V. Conti, Graham Casey, Mia M. Gaudet, Jeroen R. Huyghe, Demetrius Albanes, Melinda C. Aldrich, Angeline S. Andrew, Irene L. Andrulis, Hoda Anton-Culver, Antonis C. Antoniou, Natalia N. Antonenkova, Susanne M. Arnold, Kristan J. Aronson, Banu K. Arun, Elisa V. Bandera, Rosa B. Barkardottir, Daniel R. Barnes, Jyotsna Batra, Matthias W. Beckmann, Javier Benitez, Sara Benlloch, Andrew Berchuck, Sonja I. Berndt, Heike Bickeböller, Stephanie A. Bien, Carl Blomqvist, Stefania Boccia, Natalia V. Bogdanova, Stig E. Bojesen, Manjeet K. Bolla, Hiltrud Brauch, Hermann Brenner, James D. Brenton, Mark N. Brook, Joan Brunet, Hans Brunnström, Daniel D. Buchanan, Barbara Burwinkel, Ralf Butzow, Gabriella Cadoni, Trinidad Caldés, Maria A. Caligo, Ian Campbell, Peter T. Campbell, Géraldine Cancel-Tassin, Lisa Cannon-Albright, Daniele Campa, Neil Caporaso, André L. Carvalho, Andrew T. Chan, Jenny Chang-Claude, Stephen J. Chanock, Chu Chen, David C. Christiani, Kathleen B. M. Claes, Frank Claessens, Judith Clements, J. Margriet Collée, Marcia Cruz Correa, Fergus J. Couch, Angela Cox, Julie M. Cunningham, Cezary Cybulski, Kamila Czene, Mary B. Daly, Anna deFazio, Peter Devilee, Orland Diez, Manuela Gago-Dominguez, Jenny L. Donovan, Thilo Dörk, Eric J. Duell, Alison M. Dunning, Miriam Dwek, Diana M. Eccles, Christopher K. Edlund, Digna R. Velez Edwards, Carolina Ellberg, D. Gareth Evans, Peter A. Fasching, Robert L. Ferris, Triantafillos Liloglou, Jane C. Figueiredo, Olivia Fletcher, Renée T. Fortner, Florentia Fostira, Silvia Franceschi, Eitan Friedman, Steven J. Gallinger, Patricia A. Ganz, Judy Garber, José A. García-Sáenz, Simon A. Gayther, Graham G. Giles, Andrew K. Godwin, Mark S. Goldberg, David E. Goldgar, Ellen L. Goode, Marc T. Goodman, Gary Goodman, Kjell Grankvist, Mark H. Greene, Henrik Gronberg, Jacek Gronwald, Pascal Guénel, Niclas Håkansson, Per Hall, Ute Hamann, Freddie C. Hamdy, Robert J. Hamilton, Jochen Hampe, Aage Haugen, Florian Heitz, Rolando Herrero, Peter Hillemanns, Michael Hoffmeister, Estrid Høgdall, Yun-Chul Hong, John L. Hopper, Richard Houlston, Peter J. Hulick, David J. Hunter, David G. Huntsman, Gregory Idos, Evgeny N. Imyanitov, Sue Ann Ingles, Claudine Isaacs, Anna Jakubowska, Paul James, Mark A. Jenkins, Mattias Johansson, Mikael Johansson, Esther M. John, Amit D. Joshi, Radka Kaneva, Beth Y. Karlan, Linda E. Kelemen, Tabea Kühl, Kay-Tee Khaw, Elza Khusnutdinova, Adam S. Kibel, Lambertus A. Kiemeney, Jeri Kim, Susanne K. Kjaer, Julia A. Knight, Manolis Kogevinas, Zsofia Kote-Jarai, Stella Koutros, Vessela N. Kristensen, Jolanta Kupryjanczyk, Martin Lacko, Stephan Lam, Diether Lambrechts, Maria Teresa Landi, Philip Lazarus, Nhu D. Le, Eunjung Lee, Flavio Lejbkowicz, Heinz-Josef Lenz, Goska Leslie, Davor Lessel, Jenny Lester, Douglas A. Levine, Li Li, Christopher I. Li, Annika Lindblom, Noralane M. Lindor, Geoffrey Liu, Fotios Loupakis, Jan Lubiński, Lovise Maehle, Christiane Maier, Arto Mannermaa, Loic Le Marchand, Sara Margolin, Taymaa May, Lesley McGuffog, Alfons Meindl, Pooja Middha, Austin Miller, Roger L. Milne, Robert J. MacInnis, Francesmary Modugno, Marco Montagna, Victor Moreno, Kirsten B. Moysich, Lorelei Mucci, Kenneth Muir, Anna Marie Mulligan, Katherine L. Nathanson, David E. Neal, Andrew R. Ness, Susan L. Neuhausen, Heli Nevanlinna, Polly A. Newcomb, Lisa F. Newcomb, Finn Cilius Nielsen, Liene Nikitina-Zake, Børge G. Nordestgaard, Robert L. Nussbaum, Kenneth Offit, Edith Olah, Ali Amin Al Olama, Olufunmilayo I. Olopade, Andrew F. Olshan, Håkan Olsson, Ana Osorio, Hardev Pandha, Jong Y. Park, Nora Pashayan, Michael T. Parsons, Tanja Pejovic, Kathryn L. Penney, Wilbert H. M. Peters, Catherine M. Phelan, Amanda I. Phipps, Dijana Plaseska-Karanfilska, Miranda Pring, Darya Prokofyeva, Paolo Radice, Kari Stefansson, Susan J. Ramus, Leon Raskin, Gad Rennert, Hedy S. Rennert, Elizabeth J. van Rensburg, Marjorie J. Riggan, Harvey A. Risch, Angela Risch, Monique J. Roobol, Barry S. Rosenstein, Mary Anne Rossing, Kim De Ruyck, Emmanouil Saloustros, Dale P. Sandler, Elinor J. Sawyer, Matthew B. Schabath, Johanna Schleutker, Marjanka K. Schmidt, V. Wendy Setiawan, Hongbing Shen, Erin M. Siegel, Weiva Sieh, Christian F. Singer, Martha L. Slattery, Karina Dalsgaard Sorensen, Melissa C. Southey, Amanda B. Spurdle, Janet L. Stanford, Victoria L. Stevens, Sebastian Stintzing, Jennifer Stone, Karin Sundfeldt, Rebecca Sutphen, Anthony J. Swerdlow, Eloiza H. Tajara, Catherine M. Tangen, Adonina Tardon, Jack A. Taylor, M. Dawn Teare, Manuel R. Teixeira, Mary Beth Terry, Kathryn L. Terry, Stephen N. Thibodeau, Mads Thomassen, Line Bjørge, Marc Tischkowitz, Amanda E. Toland, Diana Torres, Paul A. Townsend, Ruth C. Travis, Nadine Tung, Shelley S. Tworoger, Cornelia M. Ulrich, Nawaid Usmani, Celine M. Vachon, Els Van Nieuwenhuysen, Ana Vega, Miguel Elías Aguado-Barrera, Qin Wang, Penelope M. Webb, Clarice R. Weinberg, Stephanie Weinstein, Mark C. Weissler, Jeffrey N. Weitzel, Catharine M. L. West, Emily White, Alice S. Whittemore, H-Erich Wichmann, Fredrik Wiklund, Robert Winqvist, Alicja Wolk, Penella Woll, Michael Woods, Anna H. Wu, Xifeng Wu, Drakoulis Yannoukakos, Wei Zheng, Shanbeh Zienolddiny, Argyrios Ziogas, Kristin K. Zorn, Jacqueline M. Lane, Richa Saxena, Duncan Thomas, Rayjean J. Hung, Brenda Diergaarde, James McKay, Ulrike Peters, Li Hsu, Montserrat García-Closas, Rosalind A. Eeles, Georgia Chenevix-Trench, Paul J. Brennan, Christopher A. Haiman, Jacques Simard, Douglas F. Easton, Stephen B. Gruber, Paul D. P. Pharoah, Alkes L. Price, Bogdan Pasaniuc, Christopher I. Amos, Peter Kraft, Sara Lindström

**Affiliations:** 1 Program in Genetic Epidemiology and Statistical Genetics, Harvard T.H. Chan School of Public Health, 677 Huntington Ave, Boston, MA 02115 USA; 2 Unit of Cardiovascular Epidemiology, Institute of Environmental Medicine, Karolinska Institutet, Nobels vagen 13, 17177 Stockholm, Sweden; 3 Department of Epidemiology, Harvard T.H. Chan School of Public Health, 677 Huntington Ave, Boston, MA 02115 USA; 4 Program in Medical and Population Genetics, Broad Institute of MIT and Harvard, 75 Ames St, Cambridge, MA 02142 USA; 5 Department of Population and Quantitative Health Sciences, Case Western Reserve University, 10900 Eucid Avenue, Cleveland, OH 44106 USA; 6 Seidman Cancer Center, University Hospitals, Cleveland, OH 44106 USA; 7 Department of Cancer Epidemiology, H. Lee Moffitt Cancer Center and Research Institute, 12902 Magnolia Dr. MRC-CANCONT, Tampa, FL 33612 USA; 8 Department of Gastrointestinal Oncology, H. Lee Moffitt Cancer Center and Research Institute, 12902 Magnolia Dr. MRC-CANCONT, Tampa, FL 33612 USA; 9 Centre for Cancer Genetic Epidemiology, Department of Oncology, University of Cambridge, 2 Worts’ Causeway, Cambridge, CB1 8RN UK; 10 Department of Biomedical Data Science, The Geisel School of Medicine at Dartmouth, 1 Medical Center Drive, Lebanon, NH 03756 USA; 11 Centre for Cancer Genetic Epidemiology, Department of Public Health and Primary Care, University of Cambridge, 2 Worts’ Causeway, Cambridge, CB1 8RN UK; 12 Department of Electron Microscopy/Molecular Pathology, The Cyprus Institute of Neurology and Genetics, 1683 Nicosia, Cyprus; 13 Genetic Epidemiology Group, International Agency for Research on Cancer, 150 Cours Albert Thomas, 69008 Lyon, France; 14 Section of Genetics, International Agency for Research on Cancer, 150 cours Albert Thomas, 69008 Lyon, France; 15 Division of Psychiatry, University College London, Maple House, 149 Tottenham Court Road, London, W1T 7NF UK; 16 UCL Genetics Institute, University College London, Gower Street, London, WC1E 6BT UK; 17 Department of Preventive Medicine, Keck School of Medicine, University of Southern California Norris Comprehensive Cancer Center, Los Angeles, CA 48109 USA; 18 Public Health Sciences, University of Virginia, P.O. Box 800717, Charlottesville, VI 22908 USA; 19 Center for Public Health Genomics, University of Virginia, P.O. Box 800717, Charlottesville, VI 22908 USA; 20 Epidemiology Research Program, American Cancer Society, 250 Williams Street NW, Atlanta, GA 30303 USA; 21 Public Health Sciences Division, Fred Hutchinson Cancer Research Center, 1100 Fairview Ave. N., Seattle, WA 98109-1024 USA; 22 Division of Cancer Epidemiology and Genetics, National Cancer Institute, 9609 Medical Center Dr, Rockville, MD 20850 USA; 23 Department of Thoracic Surgery, Division of Epidemiology, Vanderbilt University Medical Center, 609 Oxford House, Nashville, TN 37232 USA; 24 Department of Neurology, Dartmouth-Hitchcock Medical Center, 7927 Rubin Building, Room 860, One Medical Center Drive, Lebanon, NH 3756 USA; 25 Fred ALitwin Center for Cancer Genetics, Lunenfeld-Tanenbaum Research Institute of Mount Sinai Hospital, 600 University Avenue, Toronto, ON M5G1X5 Canada; 26 Department of Molecular Genetics, University of Toronto, 1 King’s College Circle, Toronto, ON M5S1A8 Canada; 27 Department of Epidemiology, Genetic Epidemiology Research Institute, University of California Irvine, 224 Irvine Hall, Irvine, CA 92617 USA; 28 NNAlexandrov Research Institute of Oncology and Medical Radiology, Settlement of Lesnoy-2, 223040 Minsk, Belarus; 29 Markey Cancer Center, University of Kentucky, 800 Rose Street, cc445, Lexington, KY 40508 USA; 30 Department of Public Health Sciences, and Cancer Research Institute, Queen’s University, 10 Stuart Street, Kingston, ON K7L 3N6 Canada; 31 Department of Breast Medical Oncology, University of Texas MD Anderson Cancer Center, 1155 Pressler St, Houston, TX 77030 USA; 32 Cancer Prevention and Control Program, Rutgers Cancer Institute of New Jersey, 195 Little Albany Street, Room 5568, New Brunswick, NJ 08903 USA; 33 Department of Pathology, Landspitali University Hospital, Hringbraut, Reykjavik, 101 Iceland; 34 BMC (Biomedical Centre), Faculty of Medicine, University of Iceland, Vatnsmyrarvegi 16, Reykjavik, 101 Iceland; 35 Australian Prostate Cancer Research Centre-Qld, Translational Research Institute, 37 Kent St, Woolloongabba, QLD 4102 Australia; 36 Institute of Health and Biomedical Innovation and School of Biomedical Science, Queensland University of Technology, 60 Musk Ave, Kelvin Grove, QLD 4059 Australia; 37 Department of Gynecology and Obstetrics, Comprehensive Cancer Center Erlangen Nuremberg, University Hospital Erlangen, Friedrich-Alexander-University Erlangen-Nuremberg, Universitaetsstrasse 21-23, 91054 Erlangen, Germany; 38 Human Cancer Genetics Programme, Spanish National Cancer Research Centre (CNIO), Calle de Melchor Fernández Almagro, 3, 28029 Madrid, Spain; 39 Biomedical Network on Rare Diseases (CIBERER), AvMonforte de Lemos, 3-5Pabellón 11Planta 0, 28029 Madrid, Spain; 40 Division of Genetics and Epidemiology, The Institute of Cancer Research, 15 Cotswold Road, London, SM2 5NG UK; 41 Department of Obstetrics and Gynecology, Duke University Medical Center, 25171 Morris Bldg, Durham, NC 27710 USA; 42 Department of Genetic Epidemiology, University Medical Center Goettingen, Humboldtallee 32, 37073 Goettingen, Germany; 43 School of Public Health, University of Washington, 1959 NE Pacific Street, Health Science Buidling, F-350, Seattle, WA 98195 USA; 44 Department of Oncology, Helsinki University Hospital, University of Helsinki, Haartmaninkatu 4, 00290 Helsinki, Finland; 45 Department of Oncology, Örebro University Hospital, 70185 Örebro, Sweden; 46 Fondazione Policlinico Universitario A. Gemelli IRCCS, 00168 Roma, Italy; 47 Università Cattolica del Sacro Cuore, 00168 Roma, Italy; 48 Department of Radiation Oncology, Hannover Medical School, Carl-Neuberg-Straße 1, 30625 Hannover, Germany; 49 Gynaecology Research Unit, Hannover Medical School, Carl-Neuberg-Straße 1, 30625 Hannover, Germany; 50 Copenhagen General Population Study, Herlev and Gentofte Hospital, Copenhagen University Hospital, Herlev Ringvej 75, 2730 Herlev, Denmark; 51 Department of Clinical Biochemistry, Herlev and Gentofte Hospital, Copenhagen University Hospital, Herlev Ringvej 75, 2730 Herlev, Denmark; 52 Faculty of Health and Medical Sciences, University of Copenhagen, Blegdamsvej 3B, 2200 Copenhagen, Denmark; 53 DrMargarete Fischer-Bosch-Institute of Clinical Pharmacology, Auerbachstr112, 70376 Stuttgart, Germany; 54 University of Tübingen, Geschwister-Scholl-Platz, 72074 Tübingen, Germany; 55 German Cancer Consortium (DKTK), German Cancer Research Center (DKFZ), Im Neuenheimer Feld 280, 69120 Heidelberg, Germany; 56 Division of Clinical Epidemiology and Aging Research, German Cancer Research Center (DKFZ), Im Neuenheimer Feld 280, 69120 Heidelberg, Germany; 57 Division of Preventive Oncology, German Cancer Research Center (DKFZ) and National Center for Tumor Diseases (NCT), Im Neuenheimer Feld 280, 69120 Heidelberg, Germany; 58 Cancer Research UK Cambridge Institute, University of Cambridge, Li Ka Shing Centre, Robinson Way, CB2 0RE Cambridge, UK; 59 Genetic Counseling Unit, Hereditary Cancer Program, IDIBGI (Institut d’Investigació Biomèdica de Girona), Catalan Institute of Oncology, CIBERONC, AvFrança s/n, 17007 Girona, Spain; 60 Clinical Sciences, Lund University, Box 117, 221 00 Lund, Sweden; 61 Department of Genetics and Pathology, Division of Laboratory Medicine, 221 85 Lund, Sweden; 62 University of Melbourne Centre for Cancer Research, Victorian Comprehensive Cancer Centre, Parkville, VIC 3010 Australia; 63 Colorectal Oncogenomics Group, Department of Clinical Pathology, The University of Melbourne, Parkville, VIC 3010 Australia; 64 Genomic Medicine and Family Cancer Clinic, Royal Melbourne Hospital, Parkville, VIC 3010 Australia; 65 Department of Obstetrics and Gynecology, University of Heidelberg, Im Neuenheimer Feld 440, 69120 Heidelberg, Germany; 66 Molecular Epidemiology Group, C080, German Cancer Research Center (DKFZ), Im Neuenheimer Feld 280, 69120 Heidelberg, Germany; 67 Department of Pathology, University of Helsinki and Helsinki University Hospital, Biomedicum Helsinki 4th Floor, Haartmaninkatu 8, 00029 Helsinki, Finland; 68 Medical Oncology Department, Hospital Clínico San Carlos, Instituto de Investigación Sanitaria San Carlos (IdISSC), Centro Investigación Biomédica en Red de Cáncer (CIBERONC), Calle del Prof Martín Lagos, 28040 Madrid, Spain; 69 Section of Genetic Oncology, Department of Laboratory Medicine, University and University Hospital of Pisa, via Roma 67, 56126 Pisa, Italy; 70 Peter MacCallum Cancer Center, 305 Grattan Street, Melbourne, VIC 3000 Australia; 71 Sir Peter MacCallum Department of Oncology, The University of Melbourne, 305 Grattan Street, Melbourne, VIC 3000 Australia; 72 Sorbonne Université, GRC N°5 ONCOTYPE-URO, Tenon Hospital, 75020 Paris, France; 73 CeRePP, Tenon Hospital, 75020 Paris, France; 74 Division of Genetic Epidemiology, Department of Medicine, University of Utah School of Medicine, Salt Lake City, UT 84112 USA; 75 George EWahlen Department of Veterans Affairs Medical Center, Salt Lake City, UT 84112 USA; 76 Division of Cancer Epidemiology, German Cancer Research Center (DKFZ), Im Neuenheimer Feld 280, 69120 Heidelberg, Germany; 77 Department of Biology, University of Pisa, 56126 Pisa, Italy; 78 Molecular Oncology Research Center, Barretos Cancer Hospital, Rua Antenor Duarte Villela, 1331, Barretos, SP 784-400 Brazil; 79 Head and Neck Surgery Department, Barretos Cancer Hospital, Pio XII, 1331, Antenor Duarte Villela St, Barretos, SP 14784-400 Brazil; 80 Division of Gastroenterology, Massachusetts General Hospital, 55 Fruit Street, Boston, MA 02114 USA; 81 Channing Division of Network Medicine, Department of Medicine, Brigham and Women’s Hospital, Harvard Medical School, 181 Longwood Avenue, Boston, MA 02115 USA; 82 Cancer Epidemiology Group, University Cancer Center Hamburg (UCCH), University Medical Center Hamburg-Eppendorf, Martinistraße 52, 20246 Hamburg, Germany; 83 Program in Epidemiology, Division of Public Health Sciences, Fred Hutchinson Cancer Research Center, 1100 Fairview Ave N, Seattle, WA 98109 USA; 84 Centre for Medical Genetics, Ghent University, De Pintelaan 185, 9000 Gent, Belgium; 85 Molecular Endocrinology Laboratory, Department of Cellular and Molecular Medicine, KU Leuven, Leuven, 3000 Belgium; 86 Department of Clinical Genetics, Erasmus University Medical Center, Wytemaweg 80, 3015 Rotterdam, CN The Netherlands; 87 University of Puerto Rico Medical Sciences Campus and Comprehensive Cancer Center, San Juan, PR 00936 USA; 88 Department of Laboratory Medicine and Pathology, Mayo Clinic, 200 First StSW, Rochester, MN 55905 USA; 89 Sheffield Institute for Nucleic Acids (SInFoNiA), Department of Oncology and Metabolism, University of Sheffield, Western Bank, Sheffield, S10 2TN UK; 90 International Hereditary Cancer Center, Department of Genetics and Pathology, Pomeranian Medical University, ulUnii Lubelskiej 1, 71-252 Szczecin, Poland; 91 Department of Medical Epidemiology and Biostatistics, Karolinska Institutet, Karolinska Univ Hospital, 171 76 Stockholm, Sweden; 92 Department of Clinical Genetics, Fox Chase Cancer Center, 333 Cottman Ave, Philadelphia, PA 19111 USA; 93 Centre for Cancer Research, The Westmead Institute for Medical Research, The University of Sydney, 176 Hawkesbury Rd, Sydney, NSW 2145 Australia; 94 Department of Gynaecological Oncology, Westmead Hospital, Hawkesbury Rd & Darcy Rd, Sydney, NSW 2145 Australia; 95 Department of Pathology, Leiden University Medical Center, Albinusdreef 2, 2333 ZA Leiden, The Netherlands; 96 Department of Human Genetics, Leiden University Medical Center, Albinusdreef 2, 2333 ZA Leiden, The Netherlands; 97 Oncogenetics Group, Clinical and Molecular Genetics Area, Vall d’Hebron Institute of Oncology (VHIO), University Hospital, Vall d’Hebron, Passeig de la Vall d’Hebron 119-129, 08035 Barcelona, Spain; 98 Genomic Medicine Group, Galician Foundation of Genomic Medicine, Instituto de Investigación Sanitaria de Santiago de Compostela (IDIS), Complejo Hospitalario Universitario de Santiago, SERGAS, Travesía da Choupana S/N, 15706 Santiago de Compostela, Spain; 99 Moores Cancer Center, University of California, San Diego, 3855 Health Sciences Drive, La Jolla, CA 92037 USA; 100 School of Social and Community Medicine, University of Bristol, Bristol, BS8 1TH UK; 101 Unit of Nutrition and Cancer, Cancer Epidemiology Research Program, Catalan Institute of Oncology (ICO-IDIBELL), AvGran Via 199-203, L’Hospitalet de Llobregat, 08908 Barcelona, Spain; 102 Department of Biomedical Sciences, Faculty of Science and Technology, University of Westminster, 309 Regent Street, London, W1B 2HW UK; 103 Cancer Sciences Academic Unit, Faculty of Medicine, University of Southampton, Tremona Road, Southampton, SO16 6YD UK; 104 Department of Medicine, Keck School of Medicine, University of Southern California, Los Angeles, CA 90033 USA; 105 Vanderbilt Epidemiology Center, Vanderbilt Genetics Institute, Department of Obstetrics and Gynecology, Vanderbilt University Medical Center, 2525 West End Avenue, Suite 600, Nashville, TN 37203 USA; 106 Department of Cancer Epidemiology, Clinical Sciences, Lund University, Barngatan 4, Skånes universitetssjukhus, 222 42 Lund, Sweden; 107 Manchester Centre for Genomic Medicine, Division of Evolution and Genomic Sciences, University of Manchester, St Mary’s Hospital, Central Manchester University Hospitals NHS Foundation Trust, Oxford Road, Manchester, M13 9WL UK; 108 David Geffen School of Medicine, Department of Medicine Division of Hematology and Oncology, University of California at Los Angeles, 10833 Le Conte Ave, Los Angeles, CA 90095 USA; 109 Department of Otolaryngology, UPMC Hillman Cancer Center, Cancer Pavilion, University of Pittsburgh, Suite 500, 5150 Centre Avenue, Pittsburgh, PA 15232 USA; 110 Molecular and Clinical Cancer Medicine, Roy Castle Lung Cancer Research Programme, The University of Liverpool Institute of Translational Medicine, The Wiliam Duncan Building, 6 West Derby Street, Liverpool, L69 3BX UK; 111 Samuel Oschin Comprehensive Cancer Institute, Cedars-Sinai Medical Center, 8700 Beverly Boulevard, Los Angeles, CA 90048 USA; 112 Keck School of Medicine, University of Southern California, 1450 Biggy Street, Los Angeles, CA 90033 USA; 113 The Breast Cancer Now Toby Robins Research Centre, The Institute of Cancer Research, 123 Old Brompton Road, London, SW7 3RP UK; 114 Molecular Diagnostics Laboratory, INRASTES, National Centre for Scientific Research ‘Demokritos’, Neapoleos 10, AgParaskevi, Athens, 15310 Greece; 115 Section of Infections, International Agency for Research on Cancer, 150 cours Albert Thomas, 69008 Lyon, France; 116 The Susanne Levy Gertner Oncogenetics Unit, Chaim Sheba Medical Center, Emek HaEla St 1, 52621 Ramat Gan, Israel; 117 Sackler Faculty of Medicine, Tel Aviv University, Haim Levanon 30, 69978 Ramat Aviv, Israel; 118 Department of Surgery, Mount Sinai Hospital, 600 University Avenue, Toronto, ON M5G 1X5 Canada; 119 Samuel Lunenfeld Research Institute, 600 University Avenue, Toronto, ON M5G 1X5 Canada; 120 University Health Network Toronto General Hospital, 200 Elizabeth St, Toronto, ON M5G 2C4 Canada; 121 Schools of Medicine and Public Health, Division of Cancer Prevention & Control Research, Jonsson Comprehensive Cancer Centre, UCLA, 650 Charles Young Drive South, Los Angeles, CA 90095-6900 USA; 122 Cancer Risk and Prevention Clinic, Dana-Farber Cancer Institute, 450 Brookline Avenue, Boston, MA 02215 USA; 123 Department of Preventive Medicine, Keck School of Medicine, University of Southern California, 1975 Zonal Ave, Los Angeles, CA 90033 USA; 124 Center for Cancer Prevention and Translational Genomics, Samuel Oschin Comprehensive Cancer Institute, Cedars-Sinai Medical Center, Spielberg Building, 8725 Alden Dr, Los Angeles, CA 90048 USA; 125 Department of Biomedical Sciences, Cedars-Sinai Medical Center, Spielberg Building, 8725 Alden Dr, Los Angeles, CA 90048 USA; 126 Cancer Epidemiology & Intelligence Division, Cancer Council Victoria, 615 St Kilda Road, Melbourne, VIC 3004 Australia; 127 Centre for Epidemiology and Biostatistics, Melbourne School of Population and Global Health, The University of Melbourne, Level 1, 723 Swanston Street, Melbourne, VIC 3010 Australia; 128 Department of Epidemiology and Preventive Medicine, Monash University, Melbourne, VIC Australia; 129 Department of Pathology and Laboratory Medicine, University of Kansas Medical Center, 3901 Rainbow Blvd, Kansas City, KS 66160 USA; 130 Department of Medicine, McGill University, 1001 Decarie Boulevard, Montréal, QC H4A3J1 Canada; 131 Division of Clinical Epidemiology, Royal Victoria Hospital, McGill University, 1001 Decarie Boulevard, Montréal, QC H4A3J1 Canada; 132 Department of Dermatology, Huntsman Cancer Institute, University of Utah School of Medicine, 2000 Circle of Hope, Salt Lake City, UT 84112 USA; 133 Department of Health Sciences Research, Mayo Clinic, 200 First StSW, Rochester, MN 55905 USA; 134 Cancer Prevention and Control, Samuel Oschin Comprehensive Cancer Institute, Cedars-Sinai Medical Center, 8700 Beverly Blvd, Room 1S37, Los Angeles, CA 90048 USA; 135 Community and Population Health Research Institute, Department of Biomedical Sciences, Cedars-Sinai Medical Center, 8700 Beverly Blvd, Room 1S37, Los Angeles, CA 90048 USA; 136 Public Health Sciences Division, Swedish Cancer Institute, 1221 Madison StSte 300, Seattle, WA 98109 USA; 137 Unit of Clinical Chemistry, Department of Medical Biosciences, Umeå University, By 6M van 2, Sjukhusomradet, Umea universitet, 901 85 Umea, Sweden; 138 Clinical Genetics Branch, National Cancer Institute, DCEG, 9609 Medical Center Dr, Bethesda, MD 20850-9772 USA; 139 Cancer & Environment Group, Center for Research in Epidemiology and Population Health (CESP), INSERM, University Paris-Sud, University Paris-Saclay, 94805 Villejuif, France; 140 Department of Environmental Medicine, Division of Nutritional Epidemiology, Karolinska Institutet, Nobels väg 13, SE-171 77, SE-171 Stockholm, Sweden; 141 Department of Oncology, Södersjukhuset, Sjukhusbacken 10, 118 83 Stockholm, Sweden; 142 Molecular Genetics of Breast Cancer, German Cancer Research Center (DKFZ), Im Neuenheimer Feld 580, 69120 Heidelberg, Germany; 143 Nuffield Department of Surgical Sciences, Faculty of Medical Science, John Radcliffe Hospital, University of Oxford, Oxford, OX1 2JD UK; 144 Department of Surgical Oncology, Princess Margaret Cancer Centre, 610 University Avenue, Toronto, Ontario M5G2M9 Canada; 145 Department of Internal Medicine 1, University Hospital Dresden, Technische Universität Dresden (TU Dresden), 01307 Dresden, Germany; 146 National Institute of Occupational Health (STAMI), Gydas vei 8, 0033 Oslo, Norway; 147 Department of Gynecology and Gynecologic Oncology, DrHorst Schmidt Kliniken Wiesbaden, Ludwig-Erhard-Straße 100, 65199 Wiesbaden, Germany; 148 Department of Gynecology and Gynecologic Oncology, Kliniken Essen-Mitte/EvangHuyssens-Stiftung/Knappschaft GmbH, Henricistrasse 92, 45136 Essen, Germany; 149 Early Detection and Prevention Section, International Agency for Research on Cancer, 150 cours Albert Thomas, 69008 Lyon, France; 150 Department of Virus, Lifestyle and Genes, Danish Cancer Society Research Center, Strandboulevarden 49, DK-2100 Copenhagen, Denmark; 151 Molecular Unit, Department of Pathology, Herlev Hospital, University of Copenhagen, Herlev Ringvej 75, DK-2730 Herlev, Denmark; 152 Preventive Medicine, Seoul National University College of Medicine, 1 Gwanak-ro, Gwanak-gu, Seoul, 151 742 Korea; 153 German Research Center for Environmental Health, Institute for Cancer Research, Ingolstadter Landstr1, London, SM2 5NG UK; 154 Center for Medical Genetics, NorthShore University HealthSystem, 1000 Central St, Evanston, IL 60201 USA; 155 The University of Chicago Pritzker School of Medicine, 924 E 57th St, Chicago, IL 60637 USA; 156 British Columbia’s Ovarian Cancer Research (OVCARE) Program, Vancouver General Hospital, BC Cancer Agency and University of British Columbia, #3427-600 West 10th Avenue, Vancouver, BC V5Z 4E6 Canada; 157 Department of Molecular Oncology, BC Cancer Agency Research Centre, #3427-600 West 10th Avenue, Vancouver, BC V5Z 4E6 Canada; 158 Department of Pathology and Laboratory Medicine, University of British Columbia, #3427-600 West 10th Avenue, Vancouver, BC V5Z 4E6 Canada; 159 NNPetrov Institute of Oncology, Leningradskaya ul, 68, StPetersburg, Russia 197758; 160 Lombardi Comprehensive Cancer Center, Georgetown University, 3800 Reservoir Road, Washington, DC 20007 USA; 161 Independent Laboratory of Molecular Biology and Genetic Diagnostics, Pomeranian Medical University, Rybacka 1, 70-204 Szczecin, Poland; 162 Parkville Familial Cancer Centre, Peter MacCallum Cancer Center, 305 Grattan Street, Melbourne, VIC 3000 Australia; 163 Department of Radiation Sciences, Umeå University, By 6M van 2, Sjukhusomradet, Umea universitet, 901 85 Umea, Sweden; 164 Department of Medicine, Division of Oncology and Stanford Cancer Institute, Stanford University School of Medicine, 780 Welch Rd, Stanford, CA 94304 USA; 165 Clinical and Translational Epidemiology Unit, Massachusetts General Hospital, 02114 Boston, MA USA; 166 Molecular Medicine Center, Department of Medical Chemistry and Biochemistry, Medical Faculty, Medical University of Sofia, Sofia, 1504 Bulgaria; 167 Women’s Cancer Program at the Samuel Oschin Comprehensive Cancer Institute, Cedars-Sinai Medical Center, 8700 Beverly Boulevard, Los Angeles, CA 90048 USA; 168 Hollings Cancer Center and Department of Public Health Sciences, Medical University of South Carolina, 68 President Street Bioengineering Building, MSC955, Charleston, SC 29425 USA; 169 Cancer Epidemiology, University Cancer Center Hamburg (UCCH), University Medical Center Hamburg-Eppendorf, Martinistraße 52, 20246 Hamburg, Germany; 170 Clinical Gerontology, Department of Public Health and Primary Care, University of Cambridge, 2 Worts’ Causeway, Cambridge, CB1 8RN UK; 171 Department of Genetics and Fundamental Medicine, Bashkir State University, ulZaki Validi 32, Ufa, Russia 450076; 172 Institute of Biochemistry and Genetics, Ufa Scientific Center of Russian Academy of Sciences, 71 prosp Oktyabrya, Ufa, Russia 450054; 173 Division of Urologic Surgery, Brigham and Womens Hospital, Boston, Massachusettes 02115 USA; 174 Radboud Institute for Health Sciences, Radboud University Medical Center, Geert Grooteplein 21, 6525 EZ Nijmegen, The Netherlands; 175 Department of Genitourinary Medical Oncology, University of Texas MD Anderson Cancer Center, 1155 Pressler St, Houston, TX 77030 USA; 176 Department of Gynaecology, Rigshospitalet, University of Copenhagen, Blegdamsvej 9, DK-2100 Copenhagen, Denmark; 177 Prosserman Centre for Population Health Research, Lunenfeld-Tanenbaum Research Institute, Sinai Health System, 60 Murray Street, Toronto, Ontario M5T 3L9 Canada; 178 Division of Epidemiology, Dalla Lana School of Public Health, University of Toronto, 155 College Street, Toronto, ON M5T3M7 Canada; 179 Centre for Research in Environmental Epidemiology (CREAL), ISGlobal, 08036 Barcelona, Spain; 180 IMIM (Hospital del Mar Research Institute), Barcelona, 08003 Spain; 181 Universitat Pompeu Fabra (UPF), Barcelona, 08002 Spain; 182 Division of Cancer Epidemiology and Genetics, National Cancer Institute, Department of Health and Human Services, National Institutes of Health, 9609 Medical Center Dr, Bethesda, MD 20892 USA; 183 Department of Cancer Genetics, Institute for Cancer Research, Oslo University Hospital Radiumhospitalet, Ullernchausseen 70, 0379 Oslo, Norway; 184 Faculty of Medicine, Institute of Clinical Medicine, University of Oslo, Kirkeveien 166, 0450 Oslo, Norway; 185 Department of Clinical Molecular Biology, Oslo University Hospital, University of Oslo, Kirkeveien 166, 0450 Oslo, Norway; 186 Department of Pathology and Laboratory Diagnostics, the Maria Sklodowska-Curie Institute - Oncology Center, Roentgena 5, 02-781 Warsaw, Poland; 187 Head and Neck Surgery, Department of Otorhinolaryngology, Maastricht University Medical Center, PDebyelaan 25, POBox 5800, 6202 AZ Maastricht, The Netherlands; 188 Department of Integrative Oncology, British Columbia Cancer Agency, Room 10-111 675 West 10th Avenue, Vancouver, BC V5Z1L3 Canada; 189 VIB Center for Cancer Biology, VIB, Herestraat 49, 3001 Leuven, Belgium; 190 Laboratory for Translational Genetics, Department of Human Genetics, University of Leuven, Oude Markt 13, 3000 Leuven, Belgium; 191 Integrative Tumor Epidemiology Branch, DCEG, National Cancer Institute, 9609 Medical Center Drive, Room SG/7E106, Rockville, MD 20850 USA; 192 College of Pharmacy, Washington State University, PBS 431 PO Box 1495, Spokane, WA 99210-1495 USA; 193 Cancer Control Research, BC Cancer Agency, 675 West 10th Avenue, Vancouver, BC V5Z 1L3 Canada; 194 Clalit Health Services, Clalit National Israeli Cancer Control Center, Carmel Medical Center, 2 Horev Street, 3436212 Haifa, Israel; 195 Institute of Human Genetics, University Medical Center Hamburg-Eppendorf, Martinistraße 52, 20246 Hamburg, Germany; 196 Gynecology Service, Department of Surgery, Memorial Sloan Kettering Cancer Center, 1275 York Avenue, New York, NY 10065 USA; 197 Gynecologic Oncology, Laura and Isaac Pearlmutter Cancer Center, NYU Langone Medical Center, 240 East 38th Street 19th Floor, New York, NY 10016 USA; 198 Department of Family Medicine and Community Health, Mary Ann Swetland Center for Environmental Health, Case Western Reserve University, Cleveland, OH 44106 USA; 199 Servicio Galego de Saude (SERGAS), Instituto de Investigación Sanitaria de Santiago de Compostela (IDIS), 15706 Santiago De Compostela, Spain; 200 Translational Research Program, Fred Hutchinson Cancer Research Center, Seattle, WA 98109 USA; 201 Department of Molecular Medicine and Surgery, Karolinska Institutet, Karolinska Univ Hospital, 171 76 Stockholm, Sweden; 202 Health Sciences Research, Mayo Clinic Arizona, 13400 EShea Blvd, Scottsdale, AZ 85259 USA; 203 Epidemiology Division, Princess Margaret Cancer Centre, 610 University Avenue, Toronto, ON M5G2M9 Canada; 204 Unit of Oncology 1, Department of Clinical and Experimental Oncology, Istituto Oncologico Veneto IRCCS, 35122 Padua, Italy; 205 Department of Medical Genetics, Oslo University Hospital, Kirkeveien 166, 0450 Oslo, Norway; 206 Institute of Human Genetics, University Hospital Ulm, Prittwitzstrasse 43, 89075 Ulm, Germany; 207 Translational Cancer Research Area, University of Eastern Finland, Yliopistonranta 1, 70210 Kuopio, Finland; 208 Institute of Clinical Medicine, Pathology and Forensic Medicine, University of Eastern Finland, Kuopio, Yliopistonranta 1, 70210 Finland; 209 Imaging Center, Department of Clinical Pathology, Kuopio University Hospital, Puijonlaaksontie 2, 70210 Kuopio, Finland; 210 Epidemiology Program, University of Hawaii Cancer Center, 701 Ilalo St, Honolulu, HI 96813 USA; 211 Department of Clinical Science and Education, Södersjukhuset, Karolinska Institutet, Stockholm, 17177 Sweden; 212 Division of Gynecologic Oncology, University Health Network, Princess Margaret Hospital, 610 University Avenue, OPG Wing, 6-811, Toronto, ON M5G 2M9 Canada; 213 Division of Gynaecology and Obstetrics, , Technische Universität München, Arcisstraße 21, 80333 Munich, Germany; 214 Faculty of Medicine, University of Heidelberg, In Neuenheimer Feld 672, 69120 Heidelberg, Germany; 215 NRG Oncology, Statistics and Data Management Center, Roswell Park Cancer Institute, Elm & Carlton Streets, Buffalo, NY 14263 USA; 216 Womens Cancer Research Center, Magee-Womens Research Institute and Hillman Cancer Center, Pittsburgh, PA 15213 USA; 217 Division of Gynecologic Oncology, Department of Obstetrics, Gynecology and Reproductive Sciences, University of Pittsburgh School of Medicine, 300 Halket Street, Pittsburgh, PA 15213 USA; 218 Immunology and Molecular Oncology Unit, Veneto Institute of Oncology IOV - IRCCS, Via Gattamelata 64, Padua, 35128 Italy; 219 Catalan Institute of Oncology, Bellvitge Biomedical Research Institute (IDIBELL), Consortium for Biomedical Research in Epidemiology and Public Health (CIBERESP) and University of Barcelona, Barcelona, 08908 Spain; 220 Division of Cancer Prevention and Control, Roswell Park Cancer Institute, Elm & Carlton Streets, Buffalo, NY 14263 USA; 221 Division of Population Health, Health Services Research and Primary Care, University of Manchester, Oxford Road, Manchester, M13 9PL UK; 222 Division of Health Sciences, Warwick Medical School, University of Warwick, Coventry, CV4 7AL UK; 223 Department of Laboratory Medicine and Pathobiology, University of Toronto, 1 King’s College Circle, Toronto, ON M5S1A8 Canada; 224 Laboratory Medicine Program, University Health Network, 200 Elizabeth Street, Toronto, ON M5G2C4 Canada; 225 Department of Medicine, Abramson Cancer Center, Perelman School of Medicine at the University of Pennsylvania, 3400 Civic Center Boulevard, Philadelphia, PA 19104 USA; 226 Department of Oncology, Addenbrooke’s Hospital, University of Cambridge, Cambridge, CB1 8RN UK; 227 NIHR Bristol Biomedical Research Centre Nutrition Theme, University of Bristol, Upper Maudlin Street, Bristol, BS2 8AE UK; 228 Department of Population Sciences, Beckman Research Institute of City of Hope, 1500 E Duarte, Duarte, CA 91010 USA; 229 Department of Obstetrics and Gynecology, Helsinki University Hospital, University of Helsinki, Haartmaninkatu 8, 00290 Helsinki, Finland; 230 Department of Urology, University of Washington, Seattle, Washington 98195 USA; 231 Center for Genomic Medicine, Rigshospitalet, Copenhagen University Hospital, Blegdamsvej 9, DK-2100 Copenhagen, Denmark; 232 Latvian Biomedical Research and Study Centre, Ratsupites str 1, Riga, LV-1067 Latvia; 233 Cancer Genetics and Prevention Program, University of California San Francisco, 1600 Divisadero St, San Francisco, CA 94143-1714 USA; 234 Clinical Genetics Research Lab, Department of Cancer Biology and Genetics, Memorial Sloan-Kettering Cancer Center, 1275 York Avenue, New York, NY 10065 USA; 235 Clinical Genetics Service, Department of Medicine, Memorial Sloan-Kettering Cancer Center, 1275 York Avenue, New York, NY 10065 USA; 236 Department of Molecular Genetics, National Institute of Oncology, Ráth György u7-9, 1122 Budapest, Hungary; 237 Department of Clinical Neurosciences, University of Cambridge, Cambridge, CB2 0QQ UK; 238 Center for Clinical Cancer Genetics, The University of Chicago, 5841S Maryland Ave, Chicago, IL 60637 USA; 239 Department of Epidemiology, Gillings School of Global Public Health, University of North Carolina, 135 Dauer Dr, Chapel Hill, NC 27599-7435 USA; 240 UNC Lineberger Comprehensive Cancer Center, 450 West Dr, Chapell Hill, NC 27599 USA; 241 The University of Surrey, Guildford, Surrey GU2 7XH UK; 242 Department of Cancer Epidemiology, HLee Moffitt Cancer Center and Research Institute, 12902 Magnolia Drive, Tampa, FL 33612 USA; 243 Department of Applied Health Research, University College London, 1-19 Torrington Place, London, WC1E 6BT UK; 244 Centre for Cancer Genetic Epidemiology, Department of Oncology, Strangeways Laboratory, University of Cambridge, Cambridge, CB1 8RN UK; 245 Department of Genetics and Computational Biology, QIMR Berghofer Medical Research Institute, 300 Herston Road, Brisbane, QLD 4006 Australia; 246 Department of Obstetrics and Gynecology, Oregon Health & Science University, 3181 SW Sam Jackson Park Road, L-466, Portland, OR 97239 USA; 247 Knight Cancer Institute, Oregon Health & Science University, 3181 SW Sam Jackson Park Road, L-466, Portland, OR 97239 USA; 248 Department of Gastroenterology, Radboud University Nijmegen Medical Center, Geert Grooteplein Zuid 10, Internal BOBox 433, 6525 GA Nijmegen, The Netherlands; 249 Department of Epidemiology, University of Washington School of Public Health, 1959 NE Pacific St, Seattle, WA 98195 USA; 250 Research Centre for Genetic Engineering and Biotechnology ‘Georgi DEfremov’, Macedonian Academy of Sciences and Arts, Boulevard Krste Petkov Misirkov, 1000 Skopje, Republic of Macedonia; 251 Bristol Dental School, University of Bristol, Lower Maudlin Street, Bristol, BS1 2LY UK; 252 Unit of Molecular Bases of Genetic Risk and Genetic Testing, Department of Research, Fondazione IRCCS (Istituto Di Ricovero e Cura a Carattere Scientifico) Istituto Nazionale dei Tumori (INT), Via Giacomo Venezian 1, 20133 Milan, Italy; 253 Decode genetics, Sturlugata 8, IS-101 Reykjavik, Reykjavik Iceland, Iceland; 254 School of Women’s and Children’s Health, Faculty of Medicine, University of NSW Sydney, 18 High St, Sydney, NSW 2052 Australia; 255 The Kinghorn Cancer Centre, Garvan Institute of Medical Research, 384 Victoria Street, Sydney, NSW 2010 Australia; 256 Division of Epidemiology, Department of Medicine, Vanderbilt Epidemiology Center, Vanderbilt-Ingram Cancer Center, Vanderbilt University School of Medicine, 1161 21st Ave S # D3300, Nashville, TN 37232 USA; 257 Clalit National Cancer Control Center, Carmel Medical Center and Technion Faculty of Medicine, 7 Michal Street, 34362 Haifa, Israel; 258 Department of Genetics, University of Pretoria, Private Bag X323, Arcadia, 0007 South Africa; 259 Department of Chronic Disease Epidemiology, Yale School of Public Health, 60 College St, New Haven, CT 06510 USA; 260 Cancer Center Cluster Salzburg at PLUS, Department of Molecular Biology, University of Salzburg, Billrothstr11, 5020 Salzburg, Austria; 261 Division of Epigenomics and Cancer Risk Factors, DKFZ – German Cancer Research Center, Im Neuenheimer Feld 280, 69120 Heidelberg, Germany; 262 Member of the German Center for Lung Research (DZL), Translational Lung Research Center Heidelberg (TLRC-H), 69120 Heidelberg, Germany; 263 Department of Urology, Erasmus University Medical Center, Wytemaweg 80, 3015 CN Rotterdam, The Netherlands; 264 Department of Radiation Oncology, Icahn School of Medicine at Mount Sinai, 1425 Madison Avenue, New York, NY 10029 USA; 265 Department of Genetics and Genomic Sciences, Icahn School of Medicine at Mount Sinai, 1425 Madison Avenue, New York, NY 10029 USA; 266 Department of Epidemiology, University of Washington, M4 C308, 1100 Fairview Ave N, Seattle, WA 98109 USA; 267 Faculty of Medicine and Health Sciences, Basic Medical Sciences, Ghent University, De Pintelaan 185, 9000 Gent, Belgium; 268 Hereditary Cancer Clinic, University Hospital of Heraklion, Voutes, 711 10 Heraklion, Greece; 269 Epidemiology Branch, National Institute of Environmental Health Sciences, NIH, 111TWAlexander Drive, Research Triangle Park, NC 27709 USA; 270 Research Oncology, Guy’s Hospital, King’s College London, Guy’s Hospital Great Maze Pond, London, SE1 9RT UK; 271 Institute of Biomedicine, University of Turku, 20014 Turku, Finland; 272 Division of Laboratory, Department of Medical Genetics, Turku University Hospital, 20014 Turku, Finland; 273 Prostate Cancer Research Center, Faculty of Medicine and Life Sciences and BioMediTech Institute, University of Tampere, 33014 Tampere, Finland; 274 Division of Molecular Pathology, The Netherlands Cancer Institute - Antoni van Leeuwenhoek Hospital, Plesmanlaan 121, 1066 CX Amsterdam, The Netherlands; 275 Division of Psychosocial Research and Epidemiology, The Netherlands Cancer Institute - Antoni van Leeuwenhoek hospital, Plesmanlaan 121, 1066 CX Amsterdam, The Netherlands; 276 Department of Preventive Medicine, Keck School of Medicine, University of Southern California, 1450 Biggy Street, Los Angeles, CA 90033 USA; 277 Department of Epidemiology and Biostatistics, Jiangsu Key Lab of Cancer Biomarkers, Prevention and Treatment, Collaborative Innovation Center for Cancer Personalized Medicine, School of Public Health, Nanjing Medical University, 101 Longmian Ave, Jiangning District, 211166 Nanjing, People’s Republic of China; 278 Department of Genetics and Genomic Sciences, Department of Population Health Science and Policy, Icahn School of Medicine at Mount Sinai, 1425 Madison Avenue, 2nd floor, New York, NY 10029 USA; 279 Dept of OB/GYN and Comprehensive Cancer Center, Medical University of Vienna, Waehringer Guertel 18-20, 1090 Vienna, Austria; 280 Department of Internal Medicine, University of Utah Health Sciences Center, 295 Chipeta Way, Salt Lake City, UT 84132 USA; 281 Department of Molecular Medicine, Aarhus University Hospital, DK-8200 Aarhus, Denmark; 282 Department of Clinical Medicine, Aarhus University, DK-8200 Aarhus, Denmark; 283 Precision Medicine, School of Clinical Sciences at Monash Health, Monash University, 246 Clayton Road, Clayton, VIC 3168 Australia; 284 Department of Clinical Pathology, The University of Melbourne, Cnr Grattan Street and Royal Parade, Melbourne, VIC 3010 Australia; 285 Department of Medicine III, University Hospital, LMU Munich, Marchioninistr15, 81377 Munich, Germany; 286 The Curtin UWA Centre for Genetic Origins of Health and Disease, Curtin University and University of Western Australia, 35 Stirling Hwy, Perth, WA 6000 Australia; 287 Department of Obstetrics and Gynecology, Sahlgrenska Cancer Center, Inst Clinical Scienses, University of Gothenburg, Blå stråket 6, 41345 Gothenburg, Sweden; 288 Epidemiology Center, College of Medicine, University of South Florida, 3650 Spectrum Blvd, Suite 100, Tampa, FL 33612 USA; 289 Division of Breast Cancer Research, The Institute of Cancer Research, London, SW7 3RP UK; 290 Department of Molecular Biology, School of Medicine of São José do Rio Preto, Av Brig Faria Lima 5416 Vila São Pedro, São José do Rio Preto, SP 15090-000 Brazil; 291 Department of Genetics and Evolutive Biology, Institute of Biosciences, University of São Paulo, Rua do Matão, 321, São Paulo, SP 05508-090 Brazil; 292 SWOG Statistical Center, Fred Hutchinson Cancer Research Center, Seattle, Washington 98109 USA; 293 Faculty of Medicine, University of Oviedo and CIBERESP, Campus del Cristo s/n, 33006 Oviedo, Spain; 294 Epigenetic and Stem Cell Biology Laboratory, National Institute of Environmental Health Sciences, NIH, 111TWAlexander Drive, Research Triangle Park, NC 27709 USA; 295 Medical Statistics Group, School of Health and Related Research (ScHARR), University of Sheffield, Regent Court, 30 Regent Street, Sheffield, S1 4DA UK; 296 Department of Genetics, Portuguese Oncology Institute, Rua DrAntónio Bernardino de Almeida 62, 4220-072 Porto, Portugal; 297 Biomedical Sciences Institute (ICBAS), University of Porto, RJorge de Viterbo Ferreira 228, 4050-013 Porto, Portugal; 298 Department of Epidemiology, Mailman School of Public Health, Columbia University, 722 West 168th Street, New York, NY 10032 USA; 299 Obstetrics and Gynecology Epidemiology Center, Brigham and Women’s Hospital, 221 Longwood Avenue RFB 368, Boston, MA 02115 USA; 300 Harvard THChan School of Public Health, 221 Longwood Avenue RFB 368, Boston, MA 02115 USA; 301 Department of Clinical Genetics, Odense University Hospital, Sonder Boulevard 29, 5000 Odence C, Denmark; 302 Department of Gynecology and Obstetrics, Haukeland University Hospital, 5021 Bergen, Norway; 303 Centre for Cancer Biomarkers CCBIO, Department of Clinical Science, University of Bergen, 5021 Bergen, Norway; 304 Program in Cancer Genetics, Departments of Human Genetics and Oncology, McGill University, 1001 Decarie Boulevard, Montréal, QC H4A3J1 Canada; 305 Department of Medical Genetics, Cambridge University, Hills Road, Cambridge, CB2 0QQ UK; 306 Department of Cancer Biology and Genetics, The Ohio State University, 460W12th Avenue, Columbus, OH 43210 USA; 307 Institute of Human Genetics, Pontificia Universidad Javeriana, Carrera 7 No40-90, Bogota, Colombia; 308 Division of Cancer Sciences, Manchester Cancer Research Centre, Faculty of Biology, Medicine and Health, Manchester Academic Health Science Centre, NIHR Manchester Biomedical Research Centre, Health Innovation Manchester, University of Manchester, Manchester, M20 4GJ UK; 309 Cancer Epidemiology Unit, Nuffield Department of Population Health, University of Oxford, Oxford, OX3 7LF UK; 310 Department of Medical Oncology, Beth Israel Deaconess Medical Center, 330 Brookline Avenue, Boston, MA 02215 USA; 311 Huntsman Cancer Institute and Department of Population Health Sciences, University of Utah, 2000 Circle of Hope, Rm 4125, Salt Lake City, UT 84112 USA; 312 Department of Oncology, Cross Cancer Institute, University of Alberta, 116 St & 85 Ave, Edmonton, AB T6G 2R3 Canada; 313 Division of Radiation Oncology, Cross Cancer Institute, University of Alberta, 116 St & 85 Ave, Edmonton, AB T6G 2R3 Canada; 314 Division of Gynecologic Oncology, Department of Obstetrics and Gynaecology and Leuven Cancer Institute, University Hospitals Leuven, Herestraat 49, 3000 Leuven, Belgium; 315 Fundación Pública Galega Medicina Xenómica & Instituto de Investigación Sanitaria de Santiago de Compostela, calle Choupana s/n, 15706 Santiago De Compostela, Spain; 316 Population Health Department, QIMR Berghofer Medical Research Institute, 300 Herston Road, Brisbane, QLD 4006 Australia; 317 Biostatistics and Computational Biology Branch, National Institute of Environmental Health Sciences, NIH, 111TWAlexander Drive, Research Triangle Park, NC 27709 USA; 318 Department of Otolaryngology/Head and Neck Surgery, University of North Carolina at Chapel Hill, Chapel Hill, 27514 NC USA; 319 City of Hope Clinical Cancer Genomics Community Research Network, 1500 East Duarte Road, Duarte, CA 91010 USA; 320 Division of Cancer Sciences, University of Manchester, Manchester Cancer Research Centre, Manchester Academic Health Science Centre,, The Christie Hospital NHS Foundation Trust, Manchester, M13 9PL UK; 321 Fred Hutchinson Cancer Research Center, 1100 Fairview Ave N, Seattle, WA 98109 USA; 322 Department of Epidemiology, University of Washington, 1100 Fairview Ave N, Seattle, WA 98109 USA; 323 Department of Health Research and Policy - Epidemiology, Stanford University School of Medicine, 259 Campus Drive, Stanford, CA 94305 USA; 324 Department of Biomedical Data Science, Stanford University School of Medicine, 259 Campus Drive, Stanford, CA 94305 USA; 325 Institute of Medical Informatics, Biometry and Epidemiology, Chair of Epidemiology, Ludwig Maximilians University, Neuherberg D-85764, Munich, 803539 Bavaria Germany; 326 Helmholtz Zentrum Munchen, German Research Center for Environmental Health (GmbH), Institute of Epidemiology, Ingolstadter Landstr1, 85764 Neuherberg, Germany; 327 Institute of Medical Statistics and Epidemiology, Technical University Munich, Munich, 80333 Germany; 328 Laboratory of Cancer Genetics and Tumor Biology, Cancer and Translational Medicine Research Unit, Biocenter Oulu, University of Oulu, Aapistie 5A, 90220 Oulu, Finland; 329 Laboratory of Cancer Genetics and Tumor Biology, Northern Finland Laboratory Centre Oulu, Aapistie 5A, 90220 Oulu, Finland; 330 Department of Surgical Sciences, Uppsala University, 751 85 Uppsala, Sweden; 331 Academic Unit of Clinical Oncology, University of Sheffield, Weston Park Hospital, Whitham Road, Sheffield, S10 2SJ UK; 332 Discipline of Genetics, Memorial University of Newfoundland, StJohn’s, NL A1C 5S7 Canada; 333 Department of Epidemiology, Division of Cancer Prevention and Population Science, The University of Texas MD Anderson Cancer Center, 1515 Holcombe Blvd, Houston, TX 77030 USA; 334 Magee-Womens Hospital, University of Pittsburgh School of Medicine, 300 Halket St, Pittsburgh, PA 15213 USA; 335 Center for Genomic Medicine and Department of Anasthesia, Massachusetts General Hospital, Boston, MA 02114 USA; 336 Human Genetics, Graduate School of Public Health, University of Pittsburgh, UPMC Cancer Pavilion, Suite 4C, Office # 467, 5150 Centre Avenue, Pittsburgh, PA 15232 USA; 337 UPMC Hillman Cancer Center, Pittsburgh, 15232 PA USA; 338 Genetic Cancer Susceptibility Group, International Agency for Research on Cancer, 150 cours Albert Thomas, 69008 Lyon, France; 339 Oncogenetics Team, The Institute of Cancer Research and Royal Marsden NHS Foundation Trust, Downs Road, Sutton, SM2 5NG UK; 340 Genomics Center, Centre Hospitalier Universitaire de Québec - Université Laval Research Center, 2705 Laurier Boulevard, Québec City, QC G1V4G2 Canada; 341 UCLA Path and Lab Med, University of California, 10833 Le Conte Ave, Los Angeles, CA 190095 USA; 342 Department of Medicine, Epidemiology Section, Institute for Clinical and Translational Research, Baylor Medical College, One Baylor Plaza, MS: BCM451, Suite 100D, Houston, TX 77030-3411 USA

**Keywords:** Cancer genetics, Genomics, Cancer, Epidemiology

Correction to: *Nature Communications* 10.1038/s41467-018-08054-4, published online 25 January 2019.

The original version of this Article contained an error in the author affiliations.

Affiliation 46 incorrectly read ‘Fondazione Policlinico Universitario A. Gemelli IRCCS, 00168 Roma, Italy.’

This has now been corrected in both the PDF and HTML versions of the Article.

