# Supplementary material for: Shared heritability and functional enrichment across six solid cancers
Source: Nat Commun. 2019 Jan 25;10:431. doi: 10.1038/s41467-018-08054-4 (PMC6347624; doi:10.1038/s41467-018-08054-4)
Supplement: Supplementary file 1 — Supplementary Information [file 41467_2018_8054_MOESM1_ESM.pdf]

Shared heritability and functional enrichment across six solid cancers

Jiang et al.

Supplementary Table1. Estimates of SNP-heritability and cross-cancer heritability based on HapMap3 SNPs using LD score regression for each cancer and its subsets.

cancer type	sample size		All HapMap3 SNPs		Remove GWAS significant hits +/- 500 kb		Proportion explained by known GWAS hits		population prevalence (cumulative risk)
	cases	controls	h^2 observed	h^2 liability	h^2 observed	h^2 liability	observed	liability	
**Breast cancer**	122977	105974	0.13 (0.011)	0.14 (0.012)	0.074 (0.0041)	0.077 (0.0043)	0.43	0.45	9.4%
ER-positive	69501	95042	0.14 (0.014)	-	0.080 (0.0052)	-	0.43	-	-
ER-negative	21468	100564	0.075 (0.0065)	-	0.057 (0.0048)	-	0.24	-	-
**Colorectal cancer**	36948	30864	0.11 (0.011)	0.090 (0.0089)	0.085 (0.0088)	0.072 (0.0074)	0.23	0.20	4.8%
**head and neck cancer**	5452	5984	0.19 (0.043)	0.097 (0.023)	0.18 (0.043)	0.097 (0.023)	0.05	0.00	0.8%
**Lung cancer**	29266	56450	0.090 (0.013)	0.075 (0.011)	0.068 (0.007)	0.056 (0.0058)	0.24	0.25	3.2%
Adenocarcinoma	11273	55483	0.067 (0.0094)	-	0.055 (0.0077)	-	0.18	-	-
Ever smoking	23223	16964	0.11 (0.024)	-	0.086 (0.016)	-	0.22	-	-
Squamous carcinoma	7426	55627	0.062 (0.013)	-	0.045 (0.009)	-	0.27	-	-
**Ovarian cancer**	22406	40941	0.048 (0.0096)	0.033 (0.0065)	0.037 (0.0076)	0.025 (0.0051)	0.23	0.24	1.6%
Serous invasive	14055	40941	0.065 (0.012)	-	0.044 (0.009)	-	0.32	-	-
**Prostate cancer**	79166	61106	0.17 (0.020)	0.18 (0.021)	0.077 (0.0054)	0.083 (0.0058)	0.55	0.54	10.5%
Advanced stage	15167	58308	0.10 (0.012)	-	0.069 (0.0085)	-	0.31	-	-

cancer type	Breast cancer	ER-positive	ER-negative	Colorectal cancer	Head and neck cancer	Lung cancer	Adenocarcinoma	Ever smoking	Squamous cell	Ovarian cancer	Serous invasive	Prostate cancer	Advanced stage
**Breast cancer**	1	**0.98 (0.0043), <1.0E-200**	**0.74 (0.025), 1.6E-198**	**0.15 (0.04), 1.1E-4**	0.029 (0.059), 0.62	**0.18 (0.037), 1.5E-6**	**0.20 (0.041), 1.0E-6**	0.18 (0.054), 6.6E-04	0.15 (0.05), 0.0027	**0.24 (0.060), 7.1E-5**	0.16 (0.061), 0.0094	0.072 (0.029), 0.012	0.074 (0.04), 0.063
ER-positive		1	**0.60 (0.032), 1.0E-79**	**0.16 (0.043), 1.3E-4**	-0.016 (0.066), 0.81	**0.13 (0.036), 5.9E-4**	**0.15 (0.043), 4.1E-4**	0.13 (0.052), 0.011	0.11 (0.052), 0.032	0.19 (0.061), 0.0020	0.11 (0.062), 0.09	0.07 (0.03), 0.02	0.075 (0.043), 0.08
ER-negative			1	0.12 (0.059), 0.045	0.21 (0.086), 0.016	**0.29 (0.059), 1.2E-6**	**0.23 (0.063), 0.00024**	**0.30 (0.074), 5.7E-5**	**0.33 (0.075), 9.9E-6**	0.24 (0.089), 0.0064	0.17 (0.08), 0.02	0.051 (0.041), 0.22	0.016 (0.058), 0.78
**Colorectal cancer**				1	-0.067 (0.094), 0.47	**0.28 (0.057), 6.6E-7**	**0.28 (0.075), 0.00026**	**0.25 (0.070), 0.00025**	0.26 (0.082), 0.0014	-0.061 (0.090), 0.5	-0.044 (0.082), 0.59	0.11 (0.049), 0.023	0.12 (0.075), 0.11
**head and neck cancer**					1	**0.57 (0.10), 4.6E-8**	**0.49 (0.12), 0.000079**	**0.60 (0.13), 3.8E-6**	**0.63 (0.13), 1.7E-6**	0.095 (0.15), 0.52	0.23 (0.13), 0.07	0.15 (0.079), 0.05	0.28 (0.11), 0.011
**Lung cancer**						1	**0.84 (0.036), 1.18E-117**	**1.00 (0.023), <1.0E-200**	**0.93 (0.041), 1.1E-112**	0.16 (0.077), 0.03	0.18 (0.077), 0.02	-0.026 (0.043), 0.54	0.036 (0.063), 0.57
Adenocarcinoma							1	**0.89 (0.049), 5.1E-75**	**0.58 (0.10), 1.7E-8**	0.083 (0.096), 0.39	0.077 (0.09), 0.39	0.058 (0.050), 0.24	0.085 (0.074), 0.26
Ever smoking								1	**0.93 (0.052), 1.6E-71**	0.17 (0.11), 0.12	0.17 (0.10), 0.078	-0.039 (0.054), 0.47	0.0009 (0.086), 0.99
Squamous carcinoma									1	0.10 (0.10), 0.32	0.19 (0.09), 0.040	-0.058 (0.049), 0.24	0.020 (0.078), 0.80
**Ovarian cancer**										1	**0.97 (0.02), <1.0E-200**	0.024 (0.07), 0.73	0.055 (0.092), 0.55
Serous invasive											1	0.033 (0.06), 0.57	-0.0059 (0.08), 0.94
**Prostate cancer**												1	**0.92 (0.021), <1.0E-200**
Advanced stage													1

Estimates of SNP-heritability on observed scale and liability scales, were calculated based on all HapMap3 SNPs, as well as SNPs after excluding GWAS significant hits ($P < 5\times10^{-8}$) and its 500k base-pairs surrounded areas. Cumulative risks were cited from Lorelei Mucci et al. JAMA 2016 (315) paper, together with the sample prevalence (proportion of cases in the total samples), were used to convert SNP-heritability from observed scale to liability scale. The genetic correlations among cancer pairs, in the brackets were standard errors, followed by p-values. Bold font: results withstood multiple corrections (Bonferroni correction, $P < 0.05/78 = 0.00064$).

Supplementary Table 2. Estimates of SNP-heritability on the liability scale based on HapMap3 SNPs using LD score regression for each cancer, remove GWAS significant hits.

cancer type	All SNPs	Remove GWAS significant hits +/- 500 kb of the same cancer		Heritability after removing GWAS significant hits +/- 500 kb of different cancers											
		h^2	Prop. exp	Breast cancer	Prop. exp	Colorectal cancer	Prop. exp	Head/ neck cancer	Prop. exp	Lung cancer	Prop. exp	Ovarian cancer	Prop. exp	Prostate cancer	Prop. exp
**Breast**	0.14 (0.012)	0.077 (0.0043)	0.45	NA	NA	0.138 (0.011)	0.01	0.14 (0.012)	0.00	0.14 (0.011)	0.00	0.138 (0.012)	0.01	0.125 (0.009)	**0.11**
**Colorectal**	0.090 (0.0089)	0.072 (0.0074)	0.20	0.081 (0.0089)	0.10	NA	NA	0.089 (0.0088)	0.01	0.089 (0.0094)	0.01	0.089 (0.009)	0.01	0.087 (0.0094)	0.03
**Head/neck**	0.097 (0.023)	0.097 (0.023)	0.00	0.094 (0.022)	0.03	0.095 (0.022)	0.02	NA	NA	0.087 (0.021)	0.10	0.096 (0.022)	0.01	0.094 (0.023)	0.03
**Lung**	0.075 (0.011)	0.056 (0.0058)	0.25	0.068 (0.011)	0.09	0.074 (0.012)	0.01	0.074 (0.011)	0.01	NA	NA	0.074 (0.011)	0.01	0.070 (0.011)	0.07
**Ovarian**	0.033 (0.0065)	0.025 (0.0051)	0.24	0.028 (0.0064)	0.15	0.033 (0.0062)	0.00	0.032 (0.0065)	0.03	0.033 (0.0065)	0.00	NA	NA	0.028 (0.0061)	0.15
**Prostate**	0.18 (0.021)	0.083 (0.0058)	0.54	0.14 (0.013)	0.22	0.16 (0.013)	0.11	0.18 (0.022)	0.00	0.18 (0.012)	0.00	0.18 (0.021)	0.00	NA	NA

Supplementary Table 3. Enrichment estimates for the 24 non-cell-type specific annotations, meta-analyzed across the six cancer types.

Category	%SNP	24 main annotations		Breast Cancer		Colorectal Cancer		Head and Neck Cancer		Lung Cancer		Ovarian Cancer		Prostate Cancer	
		Enrichment (95CI%)	P-value	Enrichment (95%CI)	P-value	Enrichment (95%CI)	P-value	Enrichment (95%CI)	P-value	Enrichment (95%CI)	P-value	Enrichment (95%CI)	P-value	Enrichment (95%CI)	P-value
Conserved region	2.57%	9.78(5.72-13.84)	**2.27E-05**	6.09(0.66-11.52)	6.92E-02	5.44(-2.32-13.2)	2.52E-01	9.8(-21.14-40.74)	5.56E-01	13.04(4.84-21.24)	5.49E-03	24.49(-2.64-51.61)	8.32E-02	14.25(7.66-20.85)	6.46E-05
TSS	1.78%	5.42(2.47-8.37)	3.35E-03	5.83(1.29-10.37)	4.22E-02	6.4(-0.81-13.61)	1.25E-01	-12.81(-43.26-17.64)	3.04E-01	3.31(-5.36-11.97)	6.02E-01	0.3(-16.78-17.38)	9.35E-01	6.31(0.47-12.16)	7.19E-02
3'UTR	1.12%	4.94(1.11-8.77)	4.39E-02	1.55(-3.14-6.24)	8.18E-01	6.28(-2.56-15.12)	2.40E-01	-9.77(-37.25-17.72)	4.32E-01	3.55(-3.08-10.17)	4.57E-01	16.34(0.32-32.36)	2.78E-02	9.06(2.45-15.68)	1.40E-02
Coding region	1.43%	4.93(1.82-8.04)	1.32E-02	2.37(-2.29-7.04)	5.62E-01	4.87(-4.33-14.07)	4.07E-01	0.49(-29.63-30.61)	9.73E-01	4.94(-3.53-13.42)	3.68E-01	2.5(-13-18.01)	8.49E-01	9.99(3.83-16.15)	2.89E-03
TFBS	13.12%	4.04(2.91-5.17)	**1.43E-07**	5.15(3.23-7.08)	7.11E-05	5.31(1.86-8.75)	1.84E-02	-1.71(-14.11-10.7)	6.63E-01	4.09(1.16-7.01)	4.09E-02	4.34(-1.34-10.02)	2.53E-01	2.62(0.7-4.54)	9.52E-02
H3K9ac	12.54%	3.41(2.14-4.69)	**2.04E-04**	3.91(1.7-6.12)	9.95E-03	5.66(3.51-7.81)	2.22E-05	-1.69(-9.13-5.74)	4.36E-01	2.13(0.05-4.21)	2.93E-01	2.7(-1.79-7.19)	4.63E-01	3.1(1.6-4.59)	7.65E-03
5'UTR	0.55%	3.31(-1.01-7.63)	2.95E-01	3.47(-2.76-9.71)	4.36E-01	-0.22(-11.64-11.2)	8.33E-01	4.86(-33.36-43.07)	8.42E-01	-1.25(-14.81-12.31)	7.43E-01	-5.64(-29.02-17.74)	5.64E-01	8.46(-0.59-17.51)	1.04E-01
H3K4me3	13.30%	3.24(2.47-4)	**8.91E-09**	4.18(2.53-5.82)	3.71E-04	3.67(2.17-5.17)	3.65E-04	-1.45(-8.07-5.17)	4.45E-01	2.17(0.34-3.99)	2.08E-01	3.33(-0.08-6.75)	1.85E-01	2.99(1.58-4.41)	5.84E-03
Enhancer	4.20%	3.22(1.41-5.04)	1.62E-02	3.41(0.33-6.48)	1.23E-01	2.07(-2.47-6.61)	6.44E-01	-7.13(-24.59-10.33)	3.18E-01	3.43(-1.74-8.6)	3.64E-01	2.53(-5.38-10.45)	7.03E-01	4.02(0.74-7.3)	5.99E-02
Weak Enhancer	2.10%	3.1(-0.23-6.43)	2.17E-01	3.56(-1.9-9.01)	3.58E-01	7.16(-0.7-15.01)	1.21E-01	-7.38(-34.66-19.89)	5.39E-01	-1.83(-9.37-5.72)	4.58E-01	10.32(-7.24-27.88)	2.94E-01	2.88(-4.53-10.28)	6.17E-01
FANTOM5 Enhancer	0.43%	3.05(-6.17-12.27)	6.63E-01	3.22(-12.38-18.83)	7.80E-01	3.19(-18.32-24.7)	8.40E-01	-41.18(-122.43-40.06)	2.53E-01	-2.81(-22.59-16.96)	7.05E-01	-7.75(-50.5-34.99)	6.88E-01	14.91(-6.21-36.03)	1.86E-01
DHS	16.62%	2.83(1.57-4.08)	4.51E-03	3.31(1.51-5.1)	1.62E-02	4.62(2.13-7.11)	5.46E-03	-4.06(-14.2-6.08)	3.08E-01	0.55(-2.1-3.2)	7.35E-01	3.28(-1.59-8.15)	3.70E-01	2.8(1.13-4.48)	3.03E-02
Super Enhancer	16.72%	2.56(2.23-2.89)	**1.99E-20**	2.69(2.27-3.11)	3.66E-11	3.32(2.47-4.17)	1.40E-07	2.82(0.5-5.14)	6.09E-02	2.17(1.62-2.73)	1.03E-05	1.91(0.86-2.95)	9.05E-02	2.58(2.04-3.12)	7.43E-08
H3K27ac (PGC)	26.88%	2.36(1.91-2.8)	**2.12E-09**	2.26(1.42-3.11)	4.91E-03	2.82(1.8-3.83)	1.59E-04	3.17(-0.6-6.94)	2.15E-01	2.57(1.54-3.59)	3.79E-03	3.37(1.45-5.28)	1.45E-02	1.75(0.91-2.59)	7.68E-02
DGF	13.60%	2.11(0.82-3.4)	9.04E-02	2.47(0.22-4.73)	2.05E-01	4.06(0.72-7.41)	6.99E-02	-5.93(-19.66-7.8)	2.41E-01	0.2(-3.39-3.78)	6.61E-01	-0.59(-7.44-6.26)	6.44E-01	2.13(-0.04-4.29)	3.17E-01
Fetal DHS	8.39%	2.06(0.58-3.53)	1.60E-01	2.69(0.06-5.31)	2.14E-01	3.37(-0.32-7.05)	2.12E-01	-3.74(-18.77-11.29)	5.30E-01	0.16(-4.07-4.39)	6.94E-01	-1.55(-9.53-6.43)	5.02E-01	2.06(-0.41-4.53)	4.03E-01
H3K27ac	38.91%	1.9(1.65-2.15)	1.86E-12	2.01(1.7-2.32)	2.61E-08	2.16(1.74-2.59)	9.00E-09	1.96(0.32-3.59)	2.12E-01	1.38(0.99-1.78)	6.82E-02	1.87(1.17-2.58)	1.19E-02	2.02(1.7-2.34)	1.59E-08
H3K4me1	42.37%	1.84(1.56-2.12)	**2.57E-09**	2.04(1.59-2.49)	5.69E-06	1.89(1.25-2.53)	7.16E-03	-0.59(-3.9-2.72)	2.76E-01	1.58(0.82-2.33)	1.41E-01	1.87(0.32-3.42)	2.57E-01	1.72(1.17-2.26)	1.17E-02
Promoter	4.63%	1.33(-0.15-2.82)	6.61E-01	1.6(-0.31-3.52)	5.39E-01	1.45(-1.73-4.64)	7.78E-01	-3.32(-17.28-10.64)	5.48E-01	3.16(-2.42-8.75)	4.49E-01	8.81(0.69-16.93)	3.18E-02	-0.17(-2.56-2.21)	3.37E-01
Transcribed	34.60%	1.26(0.93-1.59)	1.19E-01	1.2(0.67-1.72)	4.57E-01	0.84(0.04-1.65)	7.00E-01	2.06(-0.86-4.99)	4.51E-01	1.73(0.81-2.65)	9.41E-02	1.82(0.16-3.48)	3.28E-01	1.27(0.64-1.9)	4.07E-01
Intron	38.75%	1.13(0.98-1.27)	8.14E-02	1.16(0.95-1.37)	1.19E-01	1.08(0.76-1.4)	6.15E-01	1.72(0.47-2.96)	2.04E-01	1.29(0.95-1.62)	7.77E-02	1.45(0.78-2.12)	1.44E-01	0.89(0.61-1.17)	4.49E-01
Promoter Flanking	0.83%	0.55(-3.87-4.97)	8.41E-01	-1.93(-7.76-3.89)	3.23E-01	1.05(-12.33-14.43)	9.94E-01	8.56(-35.93-53.05)	7.37E-01	10.35(-4.71-25.42)	2.30E-01	13.98(-16.25-44.21)	3.65E-01	1.39(-8.59-11.37)	9.38E-01
Repressed	46.09%	0.34(0.07-0.61)	**1.15E-06**	0.23(-0.19-0.66)	6.11E-04	0.33(-0.29-0.95)	3.71E-02	1.58(-0.82-3.99)	6.15E-01	0.06(-0.66-0.78)	9.90E-03	-0.11(-1.51-1.29)	1.26E-01	0.69(0.15-1.24)	2.78E-01
CTCF	2.39%	-2.1(-4.88-0.69)	2.95E-02	0.19(-4.05-4.43)	7.06E-01	-0.11(-7.32-7.09)	7.59E-01	-8.92(-45.53-27.69)	5.71E-01	-5.76(-14.44-2.91)	1.21E-01	-11.17(-27.13-4.79)	1.10E-01	-4.23(-9.52-1.07)	4.72E-02

This meta-analysis was performed by using the enrichment estimates and standard errors calculated in LD score regression for each individual cancer type.

Bold font indicates significance after Bonferroni correction ($P < 0.05/24$) in the meta-analysis.

TSS: transcription start site; UTR: untranslated region; TFBS: transcription factor binding sites; DHS: DNase I hypersensitive sites; DGF: digital genomic footprinting; CTCF: CCCTC-binding factor.

Supplementary Table 4. Enrichment estimates for the 24 non-cell-type specific annotations in each of the cancer subtypes.

Category	% SNP	Breast Cancer Subtypes				Lung Cancer Subtypes						Ovarian Cancer Subtypes		Prostate Cancer Subtypes	
		ER-positive		ER-negative		Adenocarcinoma		Ever smoking		Squamous cell		Serous		Advanced stage	
		Enrichment (95% CI)	P-value	Enrichment (95% CI)	P-value	Enrichment (95% CI)	P-value	Enrichment (95% CI)	P-value	Enrichment (95% CI)	P-value	Enrichment (95% CI)	P-value	Enrichment (95% CI)	P-value
Coding region	1.43%	1.86(-2.71-6.43)	7.12E-01	9.72(1.9-17.55)	3.55E-02	8.28(-3.24-19.81)	2.18E-01	2.12(-12.86-17.1)	8.82E-01	9.49(-8.95-27.92)	3.54E-01	8.5(-3.49-20.5)	2.10E-01	16.09(5.7-26.48)	1.37E-03
Conserved region	2.57%	8.36(2.14-14.57)	2.37E-02	6.73(-0.62-14.08)	1.28E-01	5.96(-3.72-15.65)	3.16E-01	11.73(0.55-22.9)	5.82E-02	28.26(6.14-50.39)	1.38E-03	21(-0.95-42.95)	6.20E-02	17.42(7.29-27.55)	4.81E-04
CTCF	2.39%	-1.24(-6.03-3.55)	3.58E-01	0.72(-5.28-6.73)	9.28E-01	-8.21(-18.9-2.48)	8.68E-02	-13.17(-27.39-1.05)	3.70E-02	-7.28(-25.42-10.86)	3.74E-01	-4.5(-15.57-6.57)	3.26E-01	-6.23(-15.12-2.66)	8.74E-02
DGF	13.60%	3.68(1.22-6.14)	3.93E-02	-0.5(-3.79-2.79)	3.68E-01	-2.42(-7.26-2.43)	1.68E-01	0.18(-6.42-6.78)	8.04E-01	3.03(-6.07-12.12)	6.44E-01	-1.29(-6.53-3.94)	3.75E-01	1.59(-2.22-5.39)	7.61E-01
DHS	16.62%	4.29(2.26-6.32)	3.34E-03	0.82(-1.86-3.51)	8.98E-01	1.5(-1.86-4.86)	7.71E-01	3.46(-0.7-7.61)	2.54E-01	3.51(-2.01-9.02)	3.67E-01	0.73(-2.93-4.38)	8.83E-01	4.19(1.1-7.28)	3.72E-02
FANTOM5 Enhancer	0.43%	2.54(-14.7-19.77)	8.61E-01	-7.79(-25.43-9.86)	3.19E-01	-9.29(-36.73-18.15)	4.58E-01	-18.77(-52.47-14.94)	2.46E-01	46.47(-3.26-96.21)	3.03E-02	8.79(-21.19-38.76)	6.01E-01	22.3(-10.1-54.7)	1.82E-01
Enhancer	4.20%	4.12(0.92-7.32)	5.37E-02	1.13(-3.39-5.65)	9.54E-01	7.74(1.85-13.64)	2.50E-02	6.58(-1.53-14.7)	1.78E-01	12.25(-0.12-24.61)	4.34E-02	2.06(-4.63-8.75)	7.54E-01	4.3(-0.88-9.47)	2.04E-01
Fetal DHS	8.39%	3.44(0.84-6.04)	7.32E-02	-1.04(-4.59-2.51)	2.58E-01	1.15(-4.43-6.73)	9.58E-01	2.53(-3.94-9)	6.45E-01	4.56(-3.26-12.39)	3.56E-01	-1.07(-7.59-5.44)	5.25E-01	2.18(-2.15-6.51)	5.91E-01
H3K27ac (Hnisz)	38.91%	1.96(1.65-2.26)	1.50E-07	2.04(1.65-2.42)	1.59E-06	1.39(0.9-1.88)	1.41E-01	1.25(0.62-1.89)	4.35E-01	1.28(0.49-2.06)	4.99E-01	1.65(1.03-2.27)	3.49E-02	2.3(1.77-2.82)	2.87E-07
H3K27ac (PGC)	26.88%	2.45(1.58-3.31)	1.90E-03	2.34(1.26-3.42)	1.09E-02	2.92(1.58-4.26)	7.61E-03	3.49(1.7-5.29)	3.96E-03	3.29(0.96-5.62)	4.25E-02	2.33(0.87-3.79)	8.55E-02	2.37(0.92-3.81)	5.69E-02
H3K4me1	42.37%	1.99(1.5-2.48)	1.15E-04	2.31(1.62-3)	2.33E-04	1.83(0.85-2.81)	1.01E-01	1.7(0.51-2.89)	2.57E-01	1.84(0.13-3.56)	3.31E-01	1.65(0.41-2.88)	3.04E-01	2.05(1.27-2.83)	9.92E-03
H3K4me3	13.30%	4.54(2.86-6.22)	6.64E-05	2.82(0.85-4.79)	7.51E-02	3.28(0.81-5.76)	6.80E-02	1.89(-0.92-4.71)	5.28E-01	1.28(-2.41-4.97)	8.82E-01	0.98(-1.69-3.64)	9.87E-01	2.95(0.86-5.04)	6.87E-02
H3K9ac	12.54%	4.3(2.2-6.4)	2.33E-03	2.11(-0.09-4.3)	3.26E-01	3.13(0.17-6.09)	1.61E-01	1.98(-1.08-5.03)	5.28E-01	2.27(-1.44-5.98)	4.99E-01	1.6(-1.76-4.96)	7.28E-01	4.93(2.38-7.47)	1.03E-03
Intron	38.75%	1.13(0.9-1.36)	2.71E-01	1.13(0.85-1.41)	3.48E-01	1.15(0.72-1.59)	4.85E-01	1.34(0.74-1.94)	2.28E-01	1.61(0.84-2.37)	6.55E-02	1.28(0.79-1.77)	2.43E-01	0.7(0.3-1.1)	1.27E-01
Promoter Flanking	0.83%	-5.33(-11.24-0.58)	3.59E-02	9.42(-4.73-23.57)	2.40E-01	13.33(-4.22-30.89)	1.81E-01	13.4(-11.97-38.77)	3.36E-01	-8.77(-35.92-18.37)	4.67E-01	-6.34(-28.97-16.29)	5.19E-01	5.39(-9.7-20.47)	5.67E-01
Promoter	4.63%	1.37(-0.52-3.27)	7.00E-01	0.23(-2.83-3.3)	6.25E-01	1.71(-4.49-7.91)	8.18E-01	7.21(-1.3-15.72)	1.73E-01	5.07(-4.36-14.5)	3.95E-01	6.17(0.98-11.36)	4.99E-02	-0.47(-4.26-3.32)	4.42E-01
Repressed region	46.09%	0.17(-0.26-0.61)	3.03E-04	0.38(-0.34-1.1)	9.78E-02	0.54(-0.4-1.48)	3.31E-01	0.22(-1.05-1.49)	2.17E-01	-0.17(-1.84-1.5)	1.13E-01	0.39(-0.72-1.5)	2.95E-01	0.96(0.18-1.74)	9.25E-01
Super Enhancer	16.72%	2.62(2.16-3.09)	1.18E-10	2.42(1.85-2.98)	8.93E-06	1.94(1.19-2.7)	1.05E-02	2.37(1.41-3.33)	2.29E-03	2.8(1.24-4.35)	3.81E-03	1.77(0.96-2.59)	6.73E-02	2.84(2.01-3.66)	7.94E-06
TFBS	13.12%	5.42(3.33-7.51)	1.24E-04	2.43(0.19-4.67)	2.15E-01	2.06(-1.47-5.59)	5.57E-01	5.47(0.67-10.27)	5.99E-02	3.58(-2.34-9.51)	3.83E-01	2.14(-2.81-7.1)	6.53E-01	1.86(-1.02-4.73)	5.62E-01
Transcribed	34.60%	1.17(0.62-1.71)	5.51E-01	1.43(0.59-2.27)	3.19E-01	1.6(0.35-2.85)	3.20E-01	1.83(0.27-3.4)	2.58E-01	1.46(-0.33-3.26)	5.91E-01	1.59(0.25-2.92)	3.88E-01	0.8(-0.04-1.63)	6.28E-01
TSS	1.78%	7.32(3-11.63)	5.56E-03	1.23(-4.69-7.15)	9.39E-01	-0.11(-11.57-11.36)	8.49E-01	2.79(-9.26-14.85)	7.70E-01	12.3(-2.93-27.54)	1.14E-01	1.74(-11.81-15.28)	9.15E-01	10.26(1.44-19.08)	2.84E-02
3' UTR	1.12%	0.82(-3.84-5.47)	9.38E-01	2.48(-2.9-7.86)	5.95E-01	5.93(-3.09-14.95)	2.73E-01	0.83(-9.29-10.94)	9.73E-01	-2.05(-18.07-13.96)	7.07E-01	17.54(4.52-30.55)	4.67E-03	13.9(3.97-23.83)	6.26E-03
5' UTR	0.55%	3.44(-3.64-10.51)	5.00E-01	11.74(0.54-22.94)	6.54E-02	5.12(-14.23-24.46)	6.78E-01	1.67(-16.59-19.93)	9.42E-01	-6.19(-32.08-19.69)	5.79E-01	3.79(-13.55-21.13)	7.53E-01	20.53(4.88-36.17)	8.33E-03
Weak Enhancer	2.10%	2.88(-2.58-8.34)	5.01E-01	3.1(-5.38-11.59)	6.27E-01	-4.19(-15.93-7.56)	3.77E-01	4.16(-8.74-17.06)	6.29E-01	8.84(-10.01-27.69)	3.97E-01	4.74(-8.73-18.22)	5.88E-01	5.4(-7.35-18.14)	4.96E-01

TSS: transcription start site; UTR: untranslated region; TFBS: transcription factor binding sites; DHS: DNase I hypersensitive sites; DGF: digital genomic footprinting; CTCF: CCCTC-binding factor.

Supplementary Table 5. Quality control and imputation procedures of each cancer.

Cancer type	Imputation algorithm	Imputation reference panel	Included SNPs for association	Other data cleaning strategy
**Breast**	Part of the data used SHAPEIT for phasing and IMPUTEv2 for imputation; Part of the data used MACH and Minimac for imputation.	The October 2014 (version 3) release of the 1000 Genomes Project dataset	Imputation r-square > 0.30	SNPs with a call rate <95% in any consortium, SNPs not in Hardy-Weinberg equilibrium ($P<10^{-7}$ in controls or $P <10^{-12}$ in cases) and SNPs with concordance <98% among duplicate sample pairs were excluded. For the imputation, SNPs with a MAF <1% and a call rate <98% in any consortium, SNPs that could not be linked to the 1000 Genomes Project reference or differed significantly in frequency from the 1000 Genomes Project dataset were additionally excluded. A further 1,128 SNPs where the cluster plot was judged to be not ideal on visual inspection were excluded.
**Colorectal**	SHAPEIT for phasing and IMPUTE2v2 for imputation.	The October 2014 (version 3) release of the 1000 Genomes Project dataset	Imputation info≥0.7, certainty ≥0.9, concordance ≥0.9 for directly measured markers as well as a MAF filter of ≥0.01	Standard QC filters were applied. A first round of filtering excluded samples with <80% call rate, then variants with <80% call rate. Next, samples with <95% call rate were excluded as well as those marked for removal from various QC checks such as replicate concordance (within and across platforms), unexpected replicate search (within and across platforms), genotyped vs. reported sex concordance, plate mix-ups, and removal due to lack of consent. Markers were then excluded based on the following criteria: 1) <95% call rate; 2) duplicate error rate >1% (only in matching reps with call rate >=99%) or heterozygote duplicate error rate >5% and >2 het mismatches, and 3) SNPs with duplicate probes.
**Head and neck**	SHAPEIT for phasing, and Minimac3 for imputation	The Haplotype Reference Consortium panel	Imputation r-square > 0.30	An initial filtering step on the complete dataset excluded samples with genotyping rate <80% and SNPs with call rate <80%. During the individuals QC, samples with unsolved genetic and reported sex discrepancies and individuals with outlying autosomic heterozygosity rate were removed. Identity-by-descent (IBD) analysis performed on the LD-pruned dataset identified 103 expected experimental duplicate-pairs (IBD > 0.9), from these the sample with lower genotyping rate were excluded. Additionally, 44 unexpected relative pairs (IBD > 0.3) were identified and excluded. SNPs with deviation of Hardy-Weinberg Equilibrium in controls ($P < 1x10^{-7}$) were further excluded.
**Lung**	SHAPEIT for phasing and IMPUTE2v2 for imputation.	The October 2014 (version 3) release of the 1000 Genomes Project dataset	Imputation r-square > 0.30 and info > 0.40	Standard quality control procedures were used to exclude underperforming individuals (DNAs) and genotyping assays (judged by success rate, genotype distributions deviated from that expected by Hardy Weinberg equilibrium). Samples were subjected to genotype calling rate and individual calling rate check. 1,708 individuals were removed for call rate less than 95%, and 16,149 SNPs with call rates of less than 95% were removed. After filtering, there were 517,482 SNPs available for analysis. We applied the standard OncoArray consortium filter for removing SNPs if they showed departure from Hardy-Weinberg equilibrium in the controls (P-value $<1 \times 10^{-7}$) or cases (P-value $<1 \times 10^{-12}$).
**Ovarian**	SHAPEIT for phasing and IMPUTE2v2 for imputation.	The October 2014 (version 3) release of the 1000 Genomes Project dataset	Imputation r-square > 0.30 and MAF > 0.01.	Samples were excluded if they had a genotyping call rate <95%, if they had excessively low or high heterozygosity, if they were not female or if they were duplicates. Duplicates and close relatives were identified using in-house software that calculates a concordance matrix for all individuals. SNP quality control was carried out according to the OncoArray QC Guidelines. Only SNPs that passed quality control for all consortia were used for imputation. SNPs with a call rate <95%, SNPs deviating from Hardy-Weinberg equilibrium ($P < 1 \times 10^{-7}$ in controls or unrelated samples in CIMBA and $P < 1 \times 10^{-12}$ in cases) and SNPs with concordance <98% among duplicate pairs were excluded.
**Prostate**	SHAPEIT for phasing and IMPUTE2v2 for imputation.	The October 2014 (version 3) release of the 1000 Genomes Project dataset	Imputation r-square > 0.30	Variants likely to have problematic clusters were selected for manual inspection on the basis of the following criteria: call rate <99%, MAF <0.001, poor Illumina intensity and clustering metrics, deviation from the MAF observed in the 1KGP. SNPs with a call rate <95% by study, not in Hardy-Weinberg equilibrium ($P < 10^{-7}$ in controls or $P < 10^{-12}$ in cases) or with concordance <98% among duplicate pairs were further excluded.

Supplementary Table 6. Numbers of individuals and SNPs involved in the GWAS summary data of 38 traits.

Trait Identifier	Reference	Numbers of individuals	Numbers of SNPs	Original files
Lupus (Systemic Lupus Erythematosus)	Bentham et al., 2015 Nat Genet	14,267	654,940	https://www.immunobase.org/downloads/protected_data/GWAS_Data/
Primary Biliary Cirrhosis	Cordell et al., 2015 Nat Commun	13,239	525,775	https://www.immunobase.org/downloads/protected_data/GWAS_Data/
Celiac Disease	Dubois et al., 2010 Nat Genet	15,283	245,449	https://www.immunobase.org/downloads/protected_data/GWAS_Data/
Autism	PGC Cross-Disorder Group, 2013 Lancet	10,263	1,173,308	http://www.med.unc.edu/pgc/files/resultfiles/pgcasdeuro.gz
Bipolar Disorder	BIP Working Group of the PGC, 2011 Nat Genet	16,731	750,636	http://www.med.unc.edu/pgc/downloads/
Anorexia	Boraska et al., 2014 Mol Psych	32,143	931,185	http://www.med.unc.edu/pgc/downloads/
Schizophrenia	SCZ Working Group of the PGC, 2014 Nature	70,100	1,083,015	http://www.med.unc.edu/pgc/downloads/
Fasting Glucose	Manning et. al., 2012 Nat Genet	46,186	1,115,625	http://www.magicinvestigators.org/downloads/
Inflammatory Bowel Disease	Jostins et al., 2012 Nature	34,652	1,078,061	http://www.ibdgenetics.org/downloads.html
Crohns Disease	Jostins et al., 2012 Nature	20,883	1,051,515	http://www.ibdgenetics.org/downloads.html
Ulcerative Colitis	Jostins et al., 2012 Nature	27,432	1,076,835	http://www.ibdgenetics.org/downloads.html
Type 2 Diabetes	Morris et al., 2012 Nat Genet	60,786	968,539	http://www.diagram-consortium.org/
Coronary Artery Disease	Schunkert et al., 2011 Nat Genet	77,210	925,224	http://www.cardiogramplusc4d.org/
Body Mass Index	Locke et al., 2015 Nature	322,151	2,554,638	http://www.broadinstitute.org/collaboration/giant/index.php/GIANT_consortium_data_files
Height	Wood et al., 2014 Nat Genet	253,280	2,550,859	http://www.broadinstitute.org/collaboration/giant/index.php/GIANT_consortium_data_files
Subject Well Being	Okbay et al., 2016 Nat Genet	298,420	477,900	http://ssgac.org/documents/SWB_Full.txt.gz
Neuroticism	Okbay et al., 2016 Nat Genet	170,911	1,115,394	http://ssgac.org/documents/Neuroticism_Full.txt.gz
Years of Education	Okbay et al., 2016 Nature	328,917	1,167,614	http://ssgac.org/documents/EduYears_Main.txt.gz
Depressive Symptoms	Okbay et al., 2016 Nat Genet	161,460	1,115,394	http://ssgac.org/documents/DS_Full.txt.gz
Rheumatoid Arthritis	Okada et al., 2014 Nature	37,681	562,682	http://plaza.umin.ac.jp/yokada/datasource/software.html
High-density Lipoprotein	Willer et al., 2013 Nat Genet	187,167	2,447,442	http://csg.sph.umich.edu//abecasis/public/lipids2013/
Triglycerides	Willer et al., 2013 Nat Genet	177,860	2,439,433	http://csg.sph.umich.edu//abecasis/public/lipids2013/
Low-density Lipoprotein	Willer et al., 2013 Nat Genet	173,082	2,437,752	http://csg.sph.umich.edu//abecasis/public/lipids2013/
Sleep Duration	Lane et al., 2016 Nat Genet	111,978	9,401,335	UKBiobank
Sleep Chronotype	Lane et al., 2016 Nat Genet	100,420	8,526,705	UKBiobank
Insomnia	Lane et al., 2016 Nat Genet	58,702	9,401,335	UKBiobank
Age at Menarche	UKBiobank	74,944	1,187,029	UKBiobank
Age at Menopause	UKBiobank	44,410	1,187,051	UKBiobank
Asthma	UKBiobank	145,416	1,187,053	UKBiobank
Diastolic Blood Pressure	UKBiobank	134,011	1,186,980	UKBiobank
Systolic Blood Pressure	UKBiobank	134,011	1,187,025	UKBiobank
Eczema	UKBiobank	145,416	1,187,034	UKBiobank
Heel T-Score	UKBiobank	141,441	1,185,277	UKBiobank
Hypertension	UKBiobank	145,379	1,187,099	UKBiobank
Lung FEV1/FVC Ratio	UKBiobank	123,935	1,186,631	UKBiobank
Forced Vital Capacity	UKBiobank	123,935	1,187,014	UKBiobank
Smoking Status	UKBiobank	145,227	1,187,111	UKBiobank
Waist Hip Ratio (BMI adjusted)	UKBiobank	145,375	1,186,973	UKBiobank

Supplementary Table 7. Total SNP-heritability and z-score in each of the 38 traits.

Trait Identifier	h^2	standard error	Z
Lupus (Systemic Lupus Erythematosus)	0.45	0.065	7.0
Celiac Disease	0.36	0.048	7.6
Fasting Glucose	0.10	0.013	7.7
Primary Biliary Cirrhosis	0.50	0.063	7.8
Autism	0.45	0.056	8.1
Type 2 Diabetes	0.09	0.011	8.3
Coronary Artery Disease	0.08	0.010	8.9
Subject Well Being	0.03	0.003	9.0
Neuroticism	0.08	0.009	9.2
Ulcerative Colitis	0.27	0.029	9.3
Bipolar Disorder	0.39	0.042	9.3
Age at Menopause	0.13	0.014	9.5
Triglycerides	0.11	0.012	9.8
Rheumatoid Arthritis	0.18	0.018	10.0
Low-density lipoprotein	0.10	0.010	10.4
Sleep Duration	0.06	0.006	10.4
Crohns Disease	0.55	0.052	10.5
Depressive Symptons	0.04	0.004	10.9
Anorexia	0.19	0.017	11.0
High-density lipoprotein	0.12	0.011	11.3
Asthma	0.07	0.006	11.4
Eczema	0.07	0.006	11.6
Inflammatory Bowel Disease	0.36	0.030	12.0
Insomnia	0.13	0.011	12.1
Body Mass Index	0.13	0.008	16.9
Sleep Chronotype	0.12	0.007	17.6
Age at Menarche	0.21	0.012	18.0
Heel T-Score	0.29	0.016	18.6
Hypertension	0.11	0.006	18.7
Waist Hip ratio	0.15	0.008	19.3
Height	0.22	0.011	19.7
Smoking Status	0.11	0.005	20.1
Schizophrenia	0.40	0.019	20.8
Lung FEV1/FVC Ratio	0.24	0.011	22.0
Systolic Blood Pressure	0.19	0.008	24.0
Diastolic Blood Pressure	0.20	0.008	24.5
Years of Education	0.10	0.004	26.6
Forced Vital Capacity	0.23	0.008	28.9

Z-scores were calculated for each trait dividing the SNP-heritability by its standard error. We restricted our analysis to traits for which the z-score was at least 7.

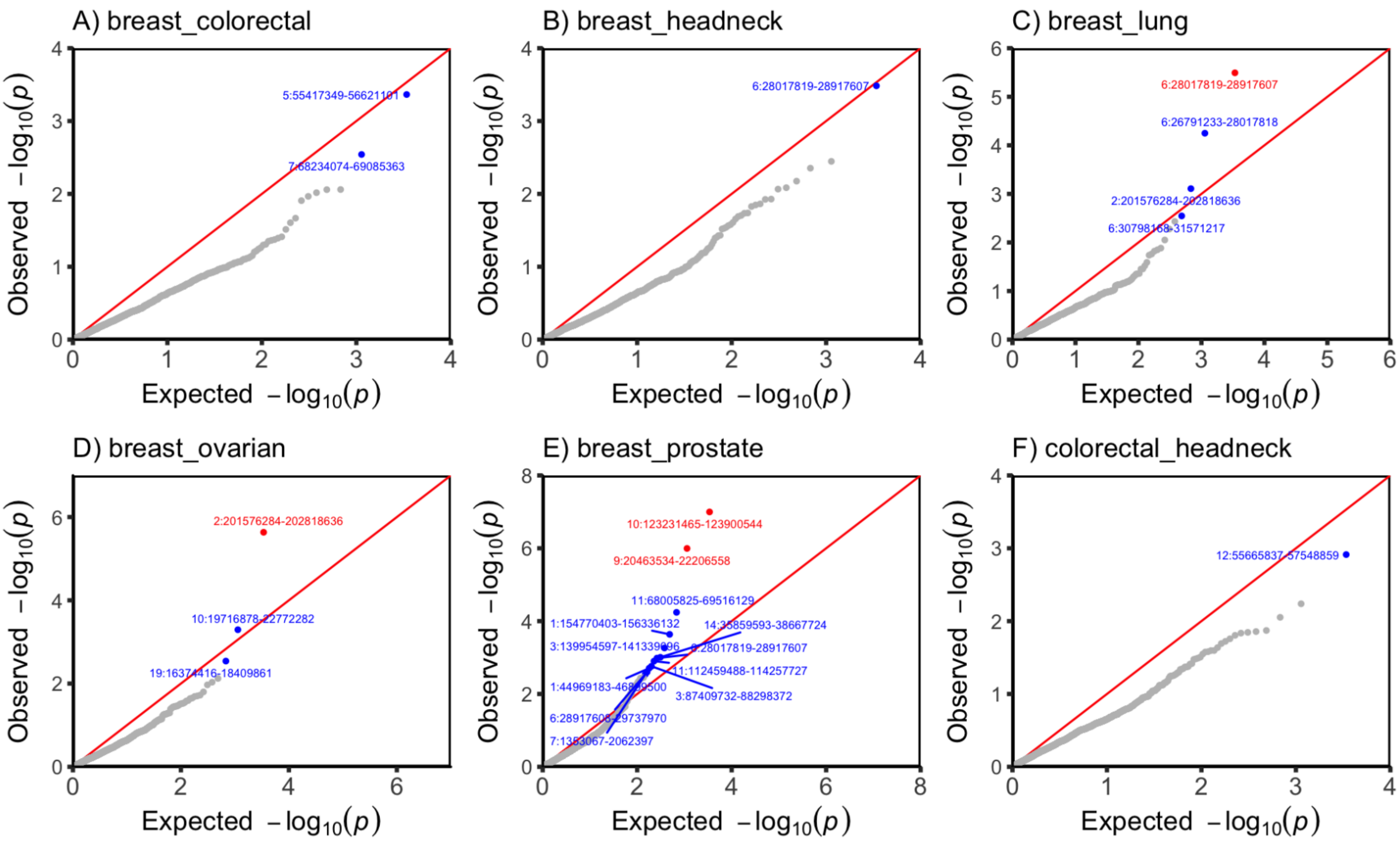

A) breast_colorectal
5:55417349-56621101
7:68234074-69085363
B) breast_headneck
6:28017819-28917607
C) breast_lung
6:28017819-28917607
6:26791233-28017818
2:201576284-202818636
6:30798168-31571217
D) breast_ovarian
2:201576284-202818636
10:19716878-22772282
19:16374416-18409861
E) breast_prostate
10:123231465-123900544
9:20463534-22206558
11:68005825-69516129
1:154770403-156336132
14:35859593-38667724
3:139954597-141339096
6:28017819-28917607
11:112459488-114257727
1:44969183-46849500
3:87409732-88298372
6:28917608-29737970
7:1353067-2062397
F) colorectal_headneck
12:55665837-57548859
Observed $-\log_{10}(p)$
Expected $-\log_{10}(p)$

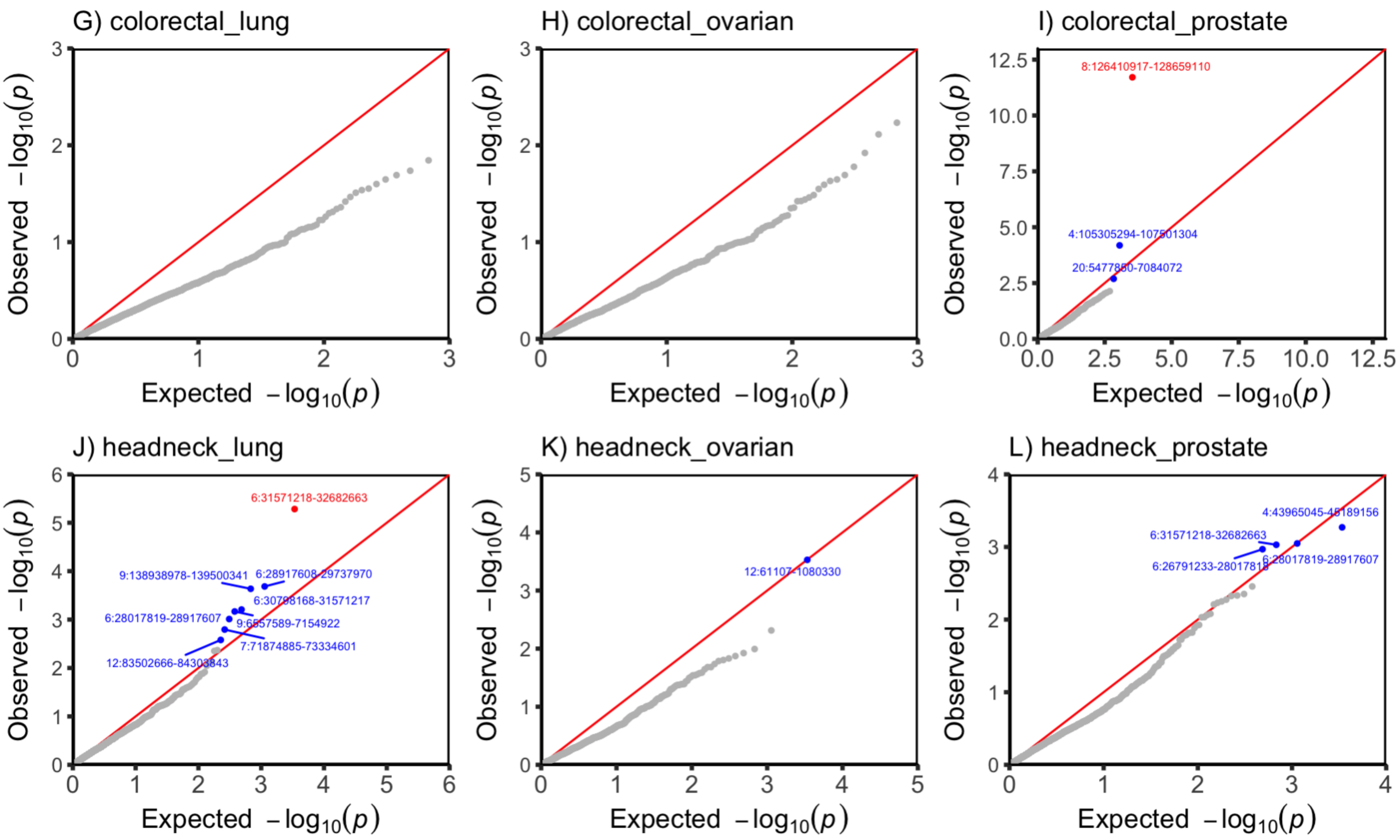

G) colorectal_lung
H) colorectal_ovarian
I) colorectal_prostate
8:126410917-128659110
4:105305294-107501304
20:5477850-7084072
J) headneck_lung
6:31571218-32682663
9:138938978-139500341
6:28917608-29737970
6:30798168-31571217
6:28017819-28917607
9:6557589-7154922
7:71874885-73334601
12:83502666-84303843
K) headneck_ovarian
12:61107-1080330
L) headneck_prostate
4:43965045-45189156
6:31571218-32682663
6:26791233-28017818
6:28017819-28917607
Observed $-\log_{10}(p)$
Expected $-\log_{10}(p)$

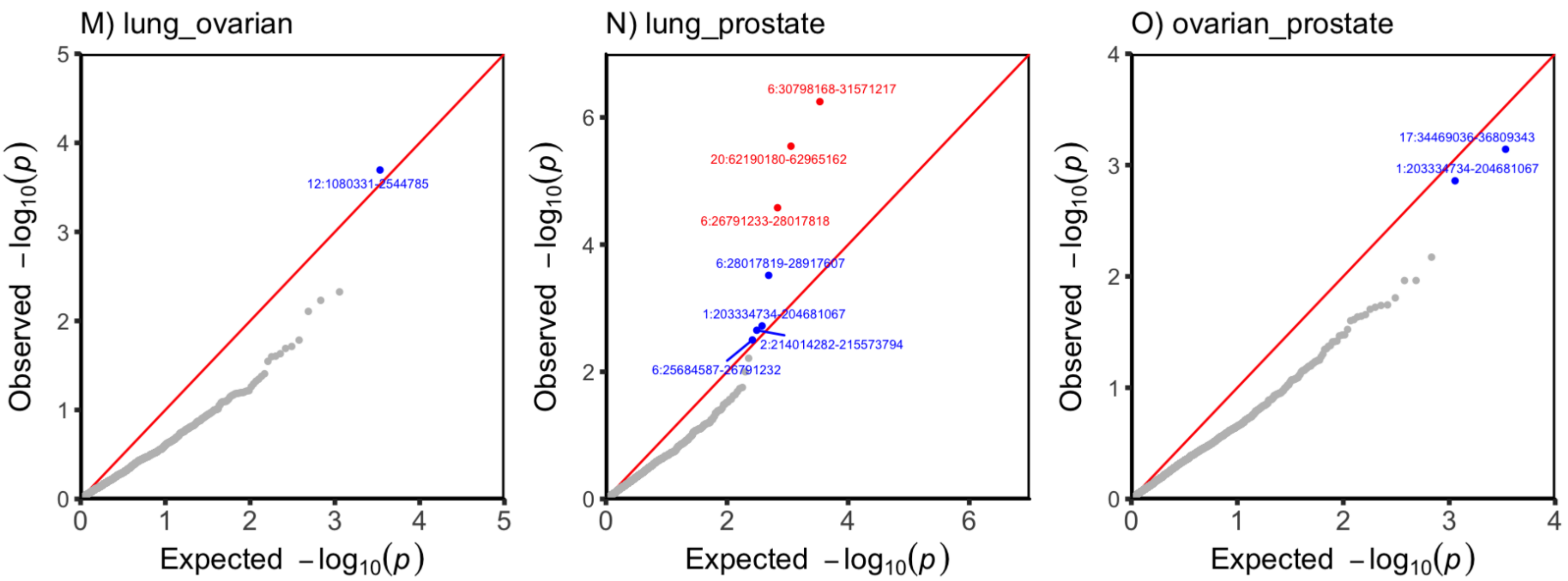

Supplementary Figure 1. Local genetic correlation between six cancers.

QQ-plots showing region-specific p-values for the local genetic covariance for breast and colorectal cancer (A), breast and head/neck cancer (B), breast and lung cancer (C), breast and ovarian cancer (D), breast and prostate cancer (E), colorectal and head/neck cancer (F), colorectal and lung cancer (G), colorectal and ovarian cancer (H), colorectal and prostate cancer (I), head/neck and lung cancer (J), head/neck and ovarian cancer (K), head/neck and prostate cancer (L), lung and ovarian cancer (M), lung and prostate cancer (N), ovarian and prostate cancer (O). Each dot presents a specific genomic region. In the QQ plots, red color indicates significance after multiple corrections (p<0.05/1,703 regions compared), and blue color indicates nominal significance (p<0.05/15 pairs of cancers compared).

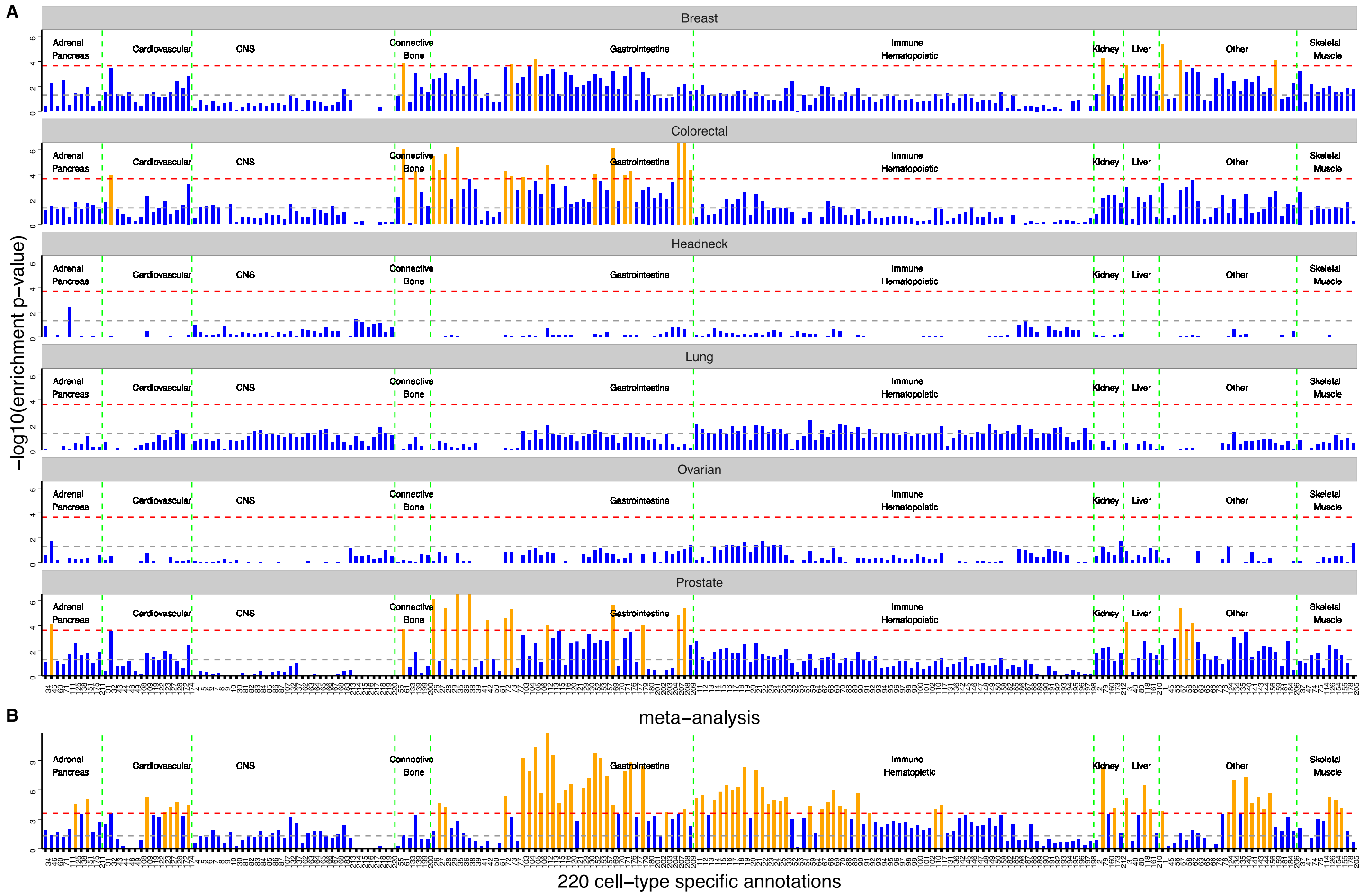

Supplementary Figure 2. Cell-type-specific functional enrichment in six cancers.

A) Enrichment p-values of 220 cell-type-specific annotations in six major cancer types. The x-axis represents each of the 220 cell types, y-axis represents the log-transformed p-values of enrichment. Annotations with statistical significance after Bonferroni corrections ($p<0.05/220$) were plotted in orange, otherwise blue. The horizontal grey dash line indicates p-threshold of 0.05; horizontal red dash line indicates p-threshold of 0.05/220. The vertical green dash lines separate 220 cell types into ten cell type groups: adrenal and pancreas, cardiovascular, central nervous system, connective and bone, gastrointestinal, immune and hematopoietic system, kidney, liver, skeletal muscle, and others. From top to bottom are six panels representing six cancers: breast cancer, colorectal cancer, head/neck cancer, lung cancer, ovarian cancer, and prostate cancer. B). Enrichment p-values of the 220 cell-type-specific annotations meta-analyzed across six cancers.

A)

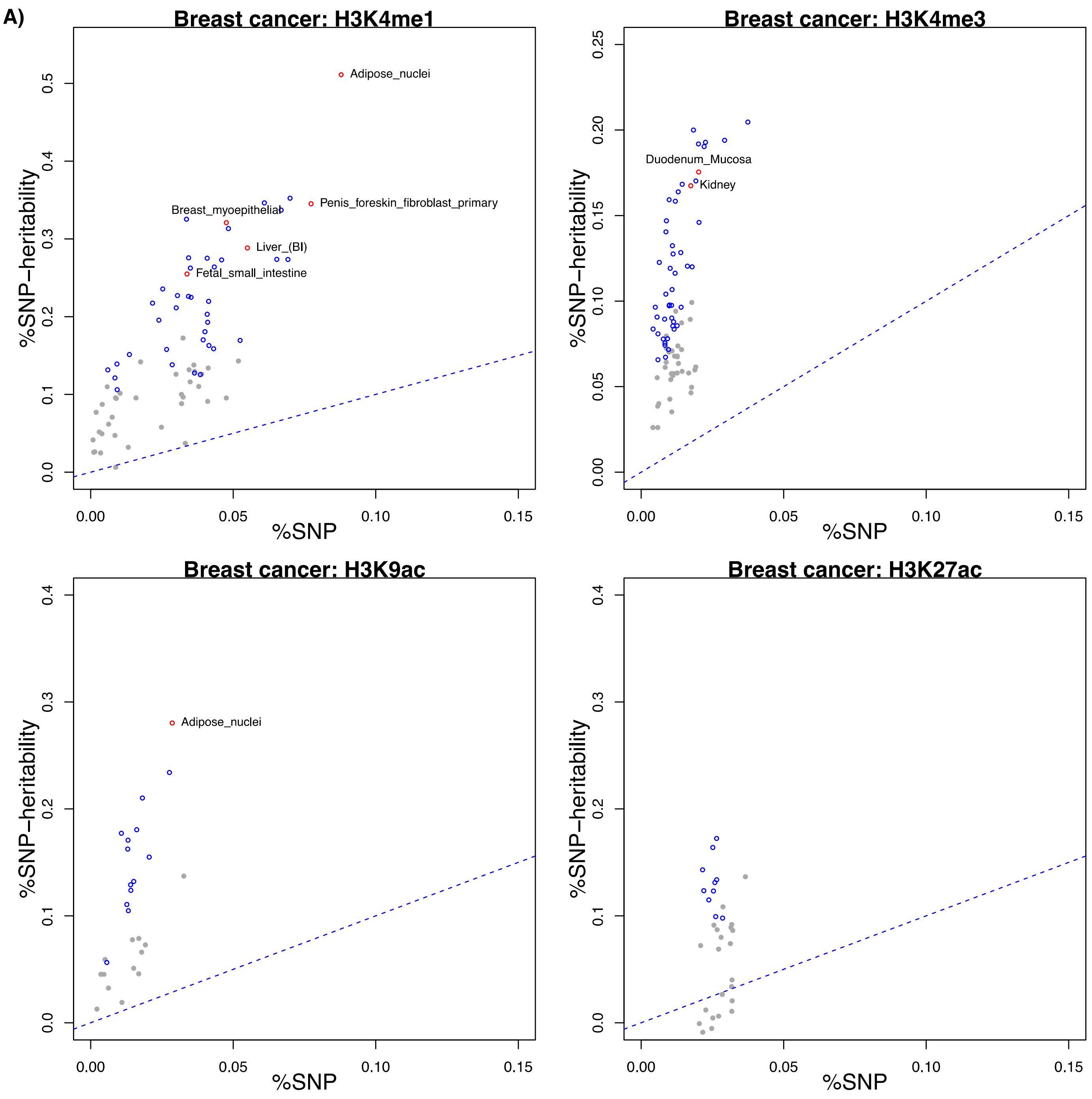

Breast cancer: H3K4me1
Adipose_nuclei
Penis_foreskin_fibroblast_primary
Breast_myoepithelial
Liver_(BI)
Fetal_small_intestine
Breast cancer: H3K4me3
Duodenum_Mucosa
Kidney
Breast cancer: H3K9ac
Adipose_nuclei
Breast cancer: H3K27ac
%SNP-heritability
%SNP
0.00
0.05
0.10
0.15
0.0
0.1
0.2
0.3
0.4
0.5
0.00
0.05
0.10
0.15
0.20
0.25

B)

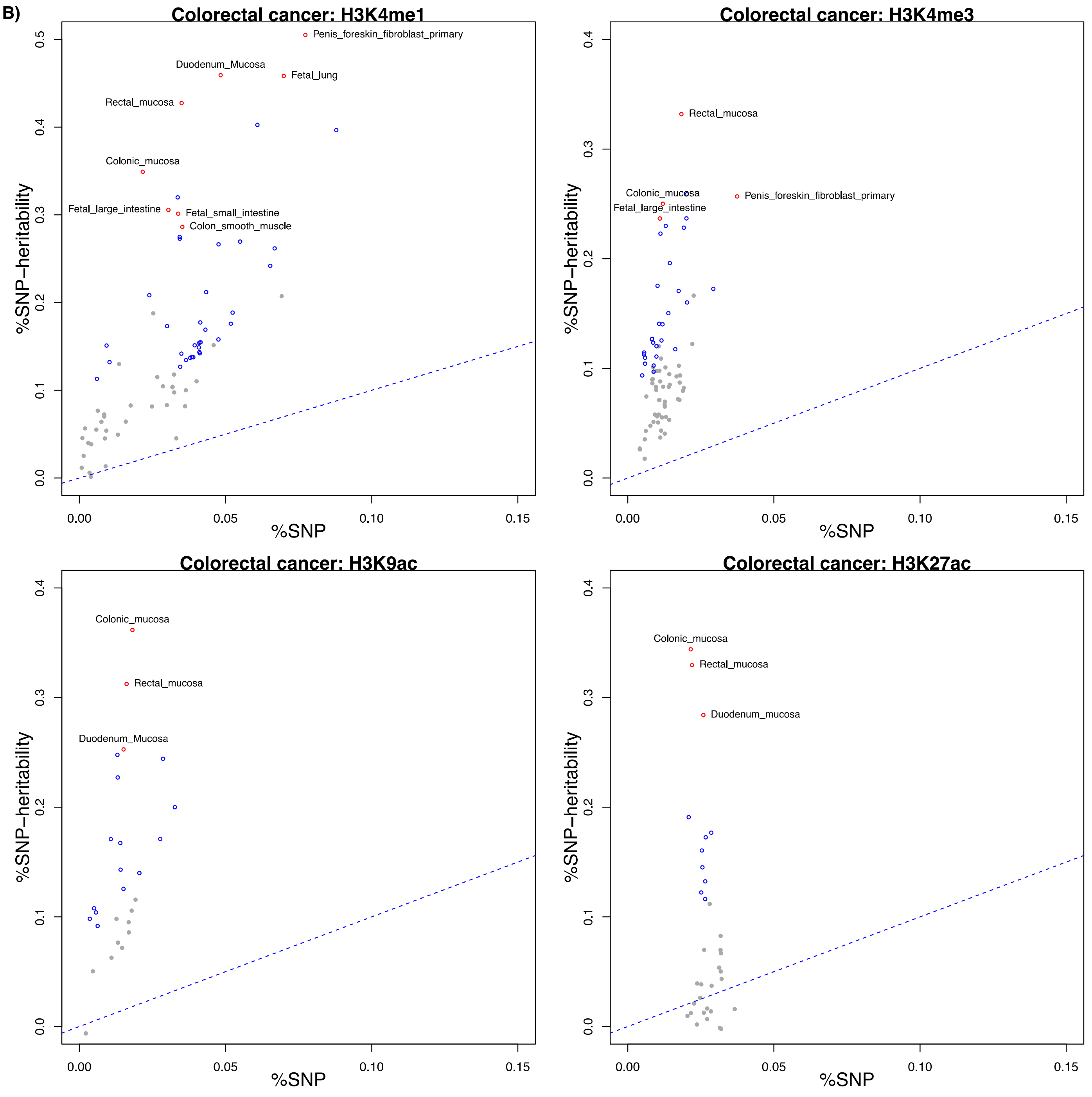
Colorectal cancer: H3K4me1
Penis_foreskin_fibroblast_primary
Duodenum_Mucosa
Fetal_lung
Rectal_mucosa
Colonic_mucosa
Fetal_large_intestine
Fetal_small_intestine
Colon_smooth_muscle
%SNP−heritability
%SNP
0.0
0.1
0.2
0.3
0.4
0.5
0.00
0.05
0.10
0.15
Colorectal cancer: H3K4me3
Rectal_mucosa
Colonic_mucosa
Fetal_large_intestine
Penis_foreskin_fibroblast_primary
%SNP−heritability
%SNP
Colorectal cancer: H3K9ac
Colonic_mucosa
Rectal_mucosa
Duodenum_Mucosa
%SNP−heritability
%SNP
Colorectal cancer: H3K27ac
Colonic_mucosa
Rectal_mucosa
Duodenum_mucosa
%SNP−heritability
%SNP

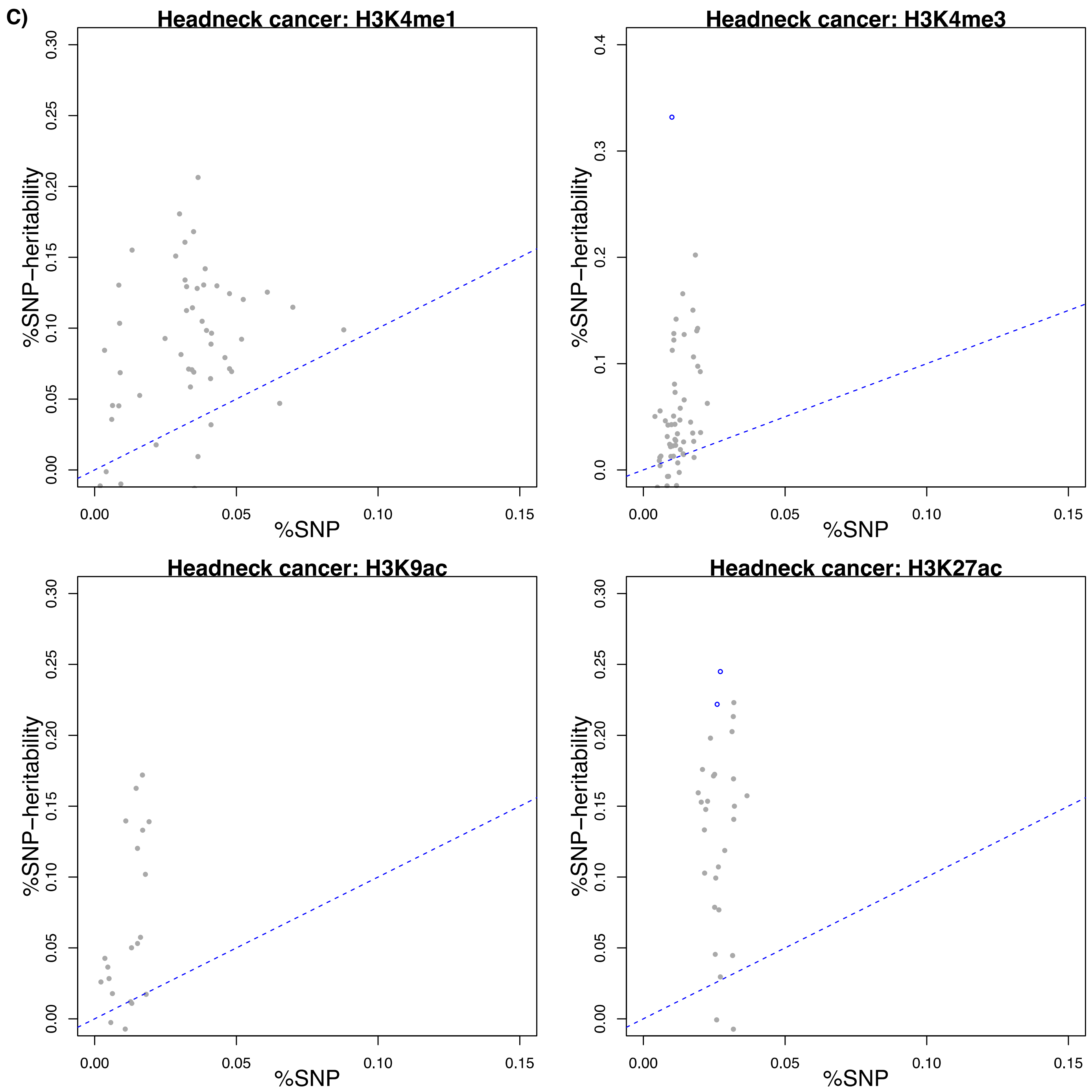
C)
Headneck cancer: H3K4me1
Headneck cancer: H3K4me3
Headneck cancer: H3K9ac
Headneck cancer: H3K27ac
%SNP−heritability
%SNP
0.00
0.05
0.10
0.15
0.20
0.25
0.30
0.0
0.1
0.2
0.3
0.4

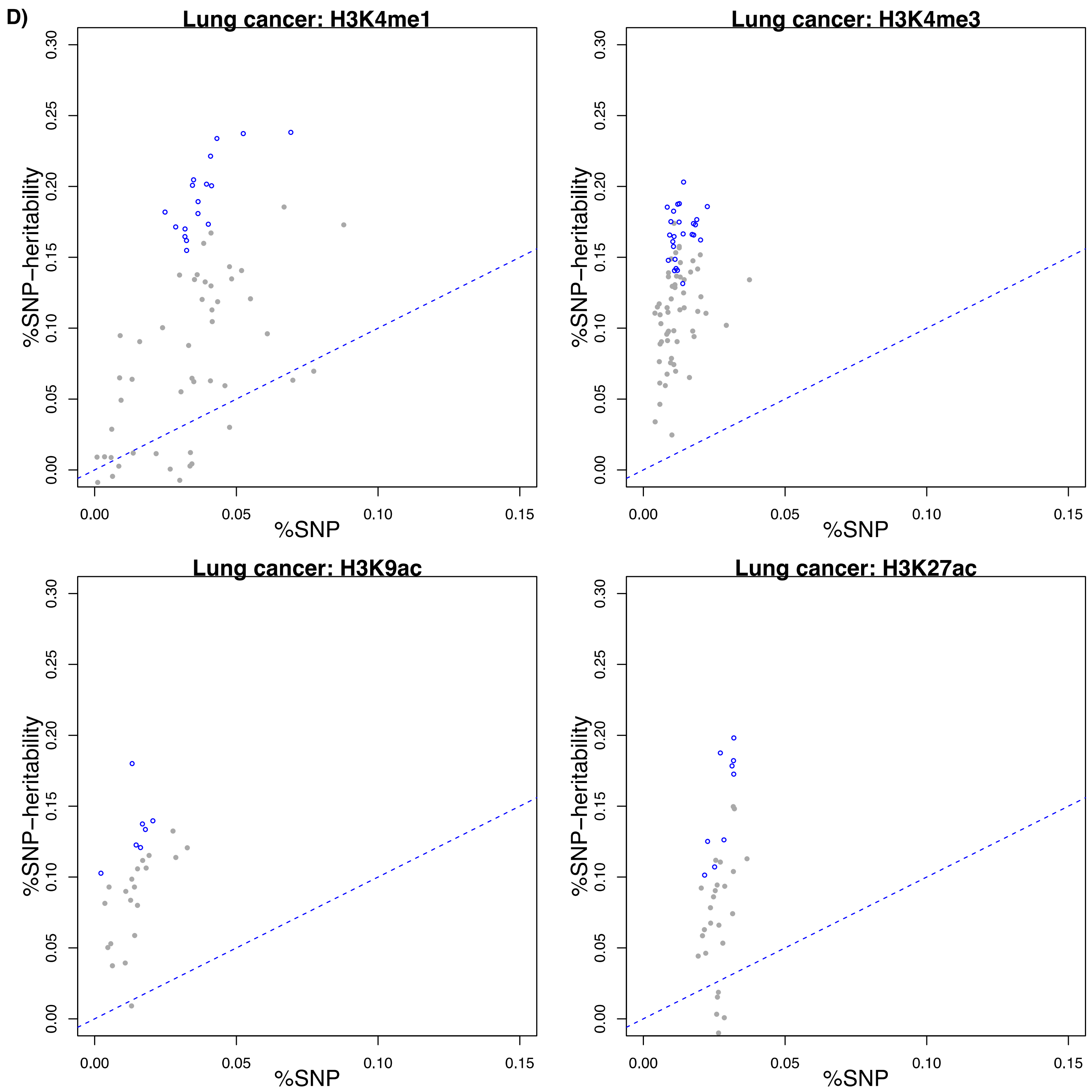
D)
Lung cancer: H3K4me1
Lung cancer: H3K4me3
Lung cancer: H3K9ac
Lung cancer: H3K27ac
%SNP−heritability
%SNP
0.00
0.05
0.10
0.15
0.20
0.25
0.30

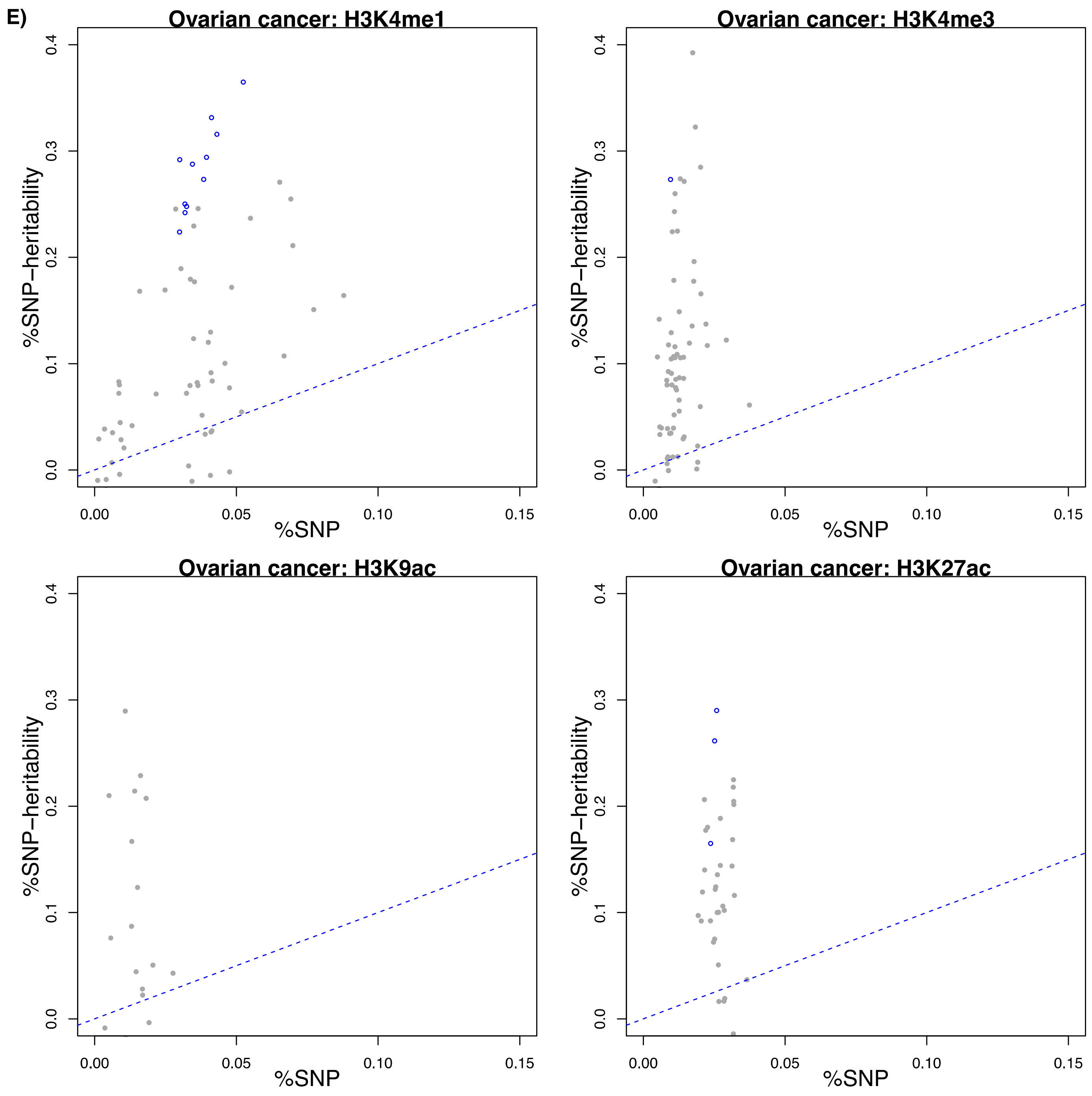

E)
Ovarian cancer: H3K4me1
Ovarian cancer: H3K4me3
Ovarian cancer: H3K9ac
Ovarian cancer: H3K27ac
%SNP−heritability
%SNP
0.0
0.1
0.2
0.3
0.4
0.00
0.05
0.10
0.15

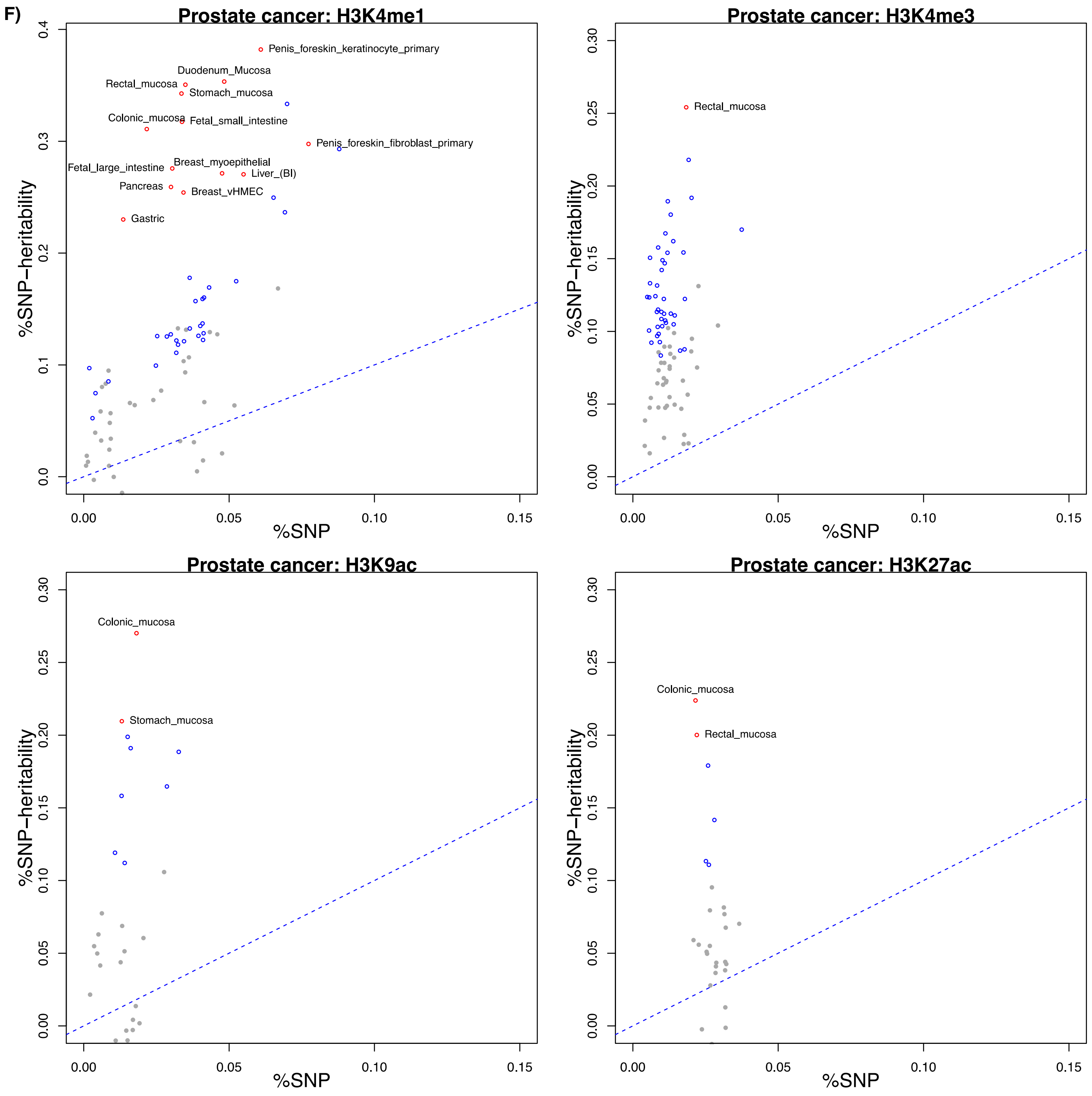
F)
Prostate cancer: H3K4me1
Penis_foreskin_keratinocyte_primary
Duodenum_Mucosa
Rectal_mucosa
Stomach_mucosa
Colonic_mucosa
Fetal_small_intestine
Penis_foreskin_fibroblast_primary
Fetal_large_intestine
Breast_myoepithelial
Liver_(BI)
Pancreas
Breast_vHMEC
Gastric
%SNP−heritability
%SNP
0.00
0.05
0.10
0.15
0.0
0.1
0.2
0.3
0.4
Prostate cancer: H3K4me3
Rectal_mucosa
%SNP−heritability
%SNP
0.00
0.05
0.10
0.15
0.20
0.25
0.30
Prostate cancer: H3K9ac
Colonic_mucosa
Stomach_mucosa
%SNP−heritability
%SNP
Prostate cancer: H3K27ac
Colonic_mucosa
Rectal_mucosa
%SNP−heritability
%SNP

Supplementary Figure 3. Cell-type-specific functional enrichment in six cancers by histone marks.

Enrichment of 220 cell-type-specific annotations in six major cancer types, plotted by histone marks (H3K4me1, H3K4me3, H3K9ac, H3K27ac). For each annotation, x-axis measures the proportion of SNPs accounted to that annotation, y-axis measures the proportion of heritability explained by that annotation. Annotations with statistical significance after Bonferroni corrections ($p<0.05/220$) are marked in red. Annotations with nominal significance ($p<0.05$) are marked in blue, the remaining annotations are marked in grey. **A)** breast cancer, **B)** colorectal cancer, **C)** head/neck cancer, **D)** lung cancer, **E)** ovarian cancer, and **F)** prostate cancer.

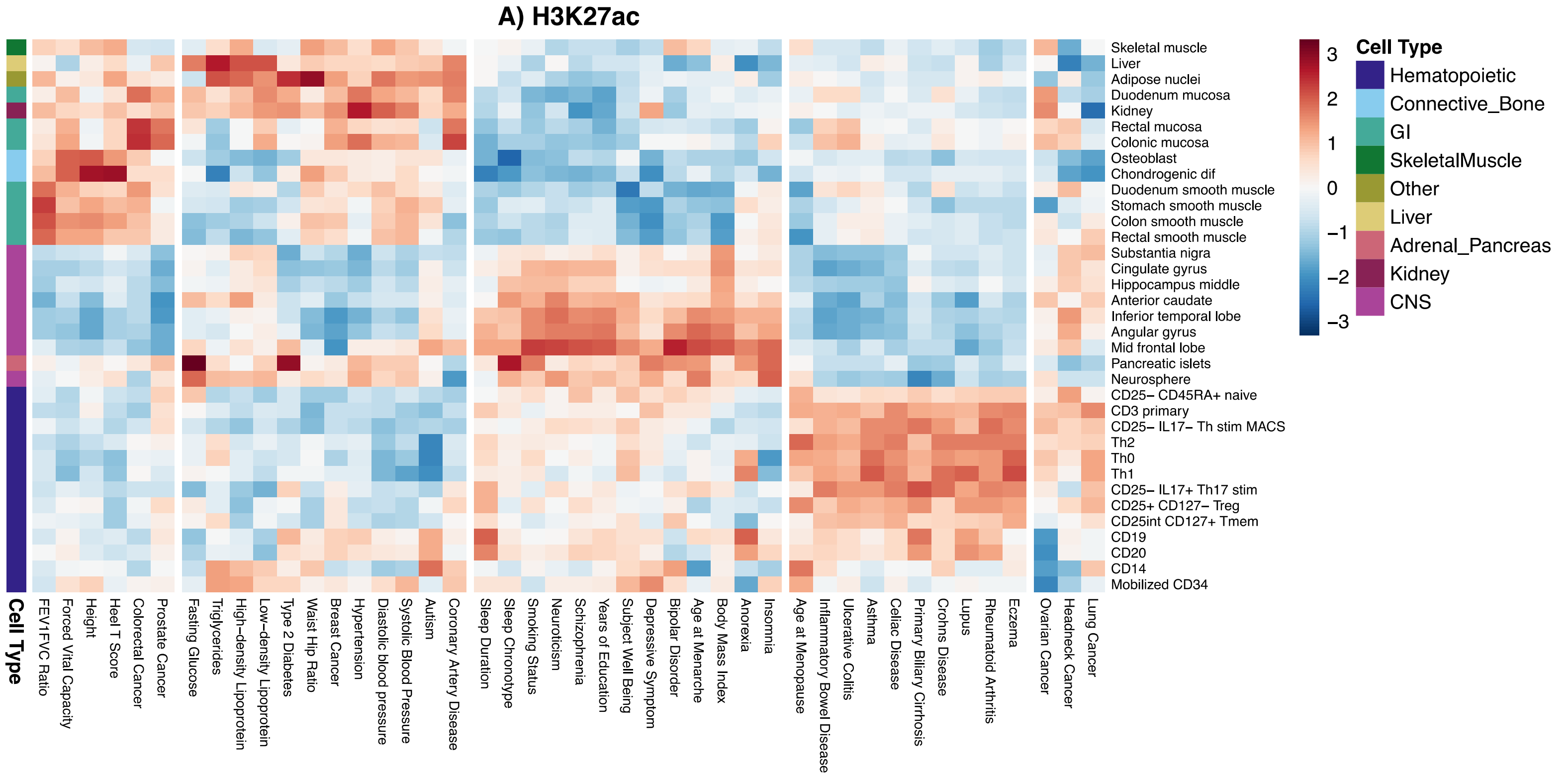
A) H3K27ac
Skeletal muscle
Liver
Adipose nuclei
Duodenum mucosa
Kidney
Rectal mucosa
Colonic mucosa
Osteoblast
Chondrogenic dif
Duodenum smooth muscle
Stomach smooth muscle
Colon smooth muscle
Rectal smooth muscle
Substantia nigra
Cingulate gyrus
Hippocampus middle
Anterior caudate
Inferior temporal lobe
Angular gyrus
Mid frontal lobe
Pancreatic islets
Neurosphere
CD25− CD45RA+ naive
CD3 primary
CD25− IL17− Th stim MACS
Th2
Th0
Th1
CD25− IL17+ Th17 stim
CD25+ CD127− Treg
CD25int CD127+ Tmem
CD19
CD20
CD14
Mobilized CD34
Cell Type
FEV1FVC Ratio
Forced Vital Capacity
Height
Heel T Score
Colorectal Cancer
Prostate Cancer
Fasting Glucose
Triglycerides
High−density Lipoprotein
Low−density Lipoprotein
Type 2 Diabetes
Waist Hip Ratio
Breast Cancer
Hypertension
Diastolic blood pressure
Systolic Blood Pressure
Autism
Coronary Artery Disease
Sleep Duration
Sleep Chronotype
Smoking Status
Neuroticism
Schizophrenia
Years of Education
Subject Well Being
Depressive Symptom
Bipolar Disorder
Age at Menarche
Body Mass Index
Anorexia
Insomnia
Age at Menopause
Inflammatory Bowel Disease
Ulcerative Colitis
Asthma
Celiac Disease
Primary Biliary Cirrhosis
Crohns Disease
Lupus
Rheumatoid Arthritis
Eczema
Ovarian Cancer
Headneck Cancer
Lung Cancer
3
2
1
0
−1
−2
−3
Cell Type
Hematopoietic
Connective_Bone
GI
SkeletalMuscle
Other
Liver
Adrenal_Pancreas
Kidney
CNS

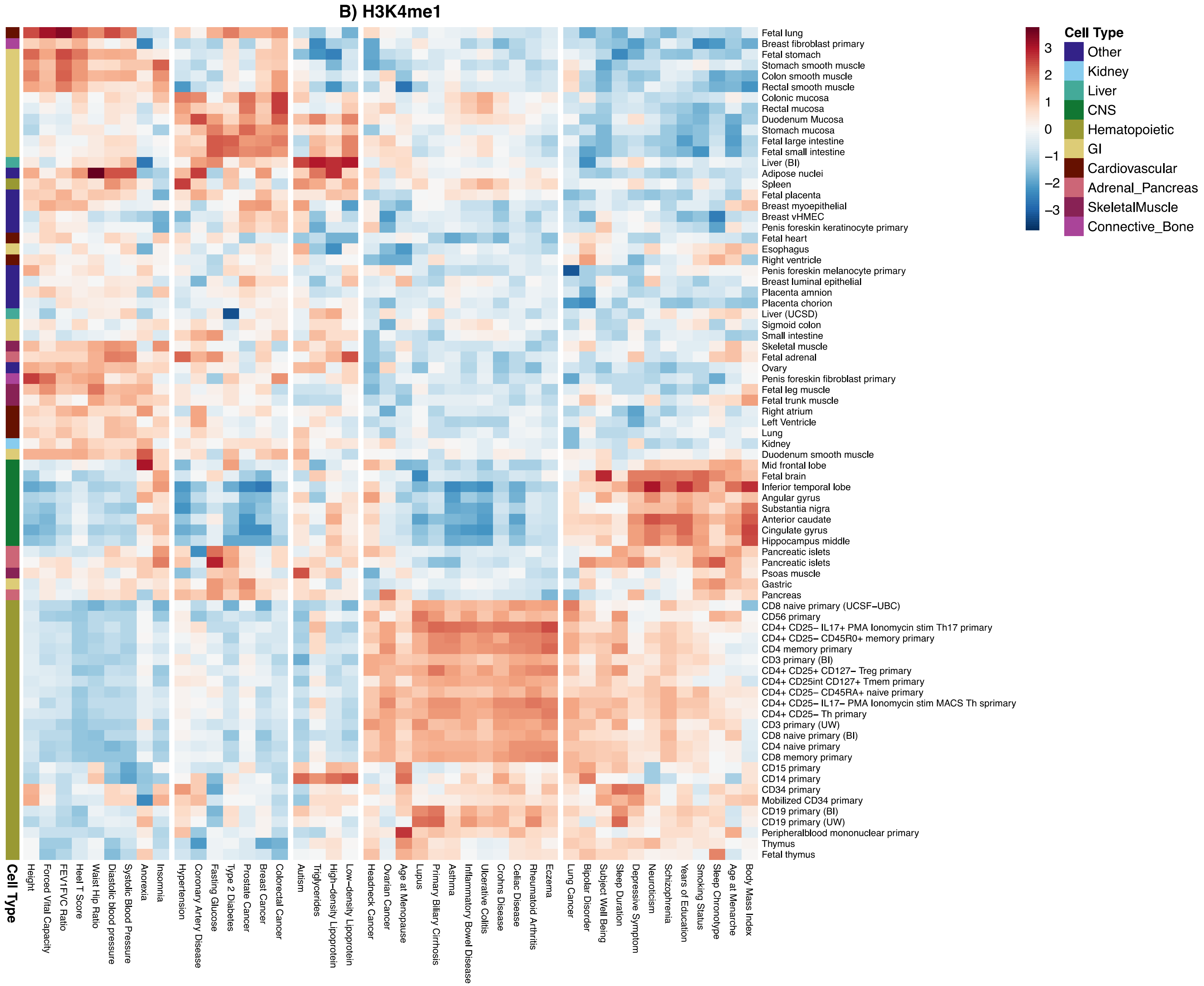

B) H3K4me1
Fetal lung
Breast fibroblast primary
Fetal stomach
Stomach smooth muscle
Colon smooth muscle
Rectal smooth muscle
Colonic mucosa
Rectal mucosa
Duodenum Mucosa
Stomach mucosa
Fetal large intestine
Fetal small intestine
Liver (BI)
Adipose nuclei
Spleen
Fetal placenta
Breast myoepithelial
Breast vHMEC
Penis foreskin keratinocyte primary
Fetal heart
Esophagus
Right ventricle
Penis foreskin melanocyte primary
Breast luminal epithelial
Placenta amnion
Placenta chorion
Liver (UCSD)
Sigmoid colon
Small intestine
Skeletal muscle
Fetal adrenal
Ovary
Penis foreskin fibroblast primary
Fetal leg muscle
Fetal trunk muscle
Right atrium
Left Ventricle
Lung
Kidney
Duodenum smooth muscle
Mid frontal lobe
Fetal brain
Inferior temporal lobe
Angular gyrus
Substantia nigra
Anterior caudate
Cingulate gyrus
Hippocampus middle
Pancreatic islets
Pancreatic islets
Psoas muscle
Gastric
Pancreas
CD8 naive primary (UCSF−UBC)
CD56 primary
CD4+ CD25− IL17+ PMA Ionomycin stim Th17 primary
CD4+ CD25− CD45R0+ memory primary
CD4 memory primary
CD3 primary (BI)
CD4+ CD25+ CD127− Treg primary
CD4+ CD25int CD127+ Tmem primary
CD4+ CD25− CD45RA+ naive primary
CD4+ CD25− IL17− PMA Ionomycin stim MACS Th sprimary
CD4+ CD25− Th primary
CD3 primary (UW)
CD8 naive primary (BI)
CD4 naive primary
CD8 memory primary
CD15 primary
CD14 primary
CD34 primary
Mobilized CD34 primary
CD19 primary (BI)
CD19 primary (UW)
Peripheralblood mononuclear primary
Thymus
Fetal thymus
Cell Type
Height
Forced Vital Capacity
FEV1FVC Ratio
Heel T Score
Waist Hip Ratio
Diastolic blood pressure
Systolic Blood Pressure
Anorexia
Insomnia
Hypertension
Coronary Artery Disease
Fasting Glucose
Type 2 Diabetes
Prostate Cancer
Breast Cancer
Colorectal Cancer
Autism
Triglycerides
High−density Lipoprotein
Low−density Lipoprotein
Headneck Cancer
Ovarian Cancer
Age at Menopause
Lupus
Primary Biliary Cirrhosis
Asthma
Inflammatory Bowel Disease
Ulcerative Colitis
Crohns Disease
Celiac Disease
Rheumatoid Arthritis
Eczema
Lung Cancer
Bipolar Disorder
Subject Well Being
Sleep Duration
Depressive Symptom
Neuroticism
Schizophrenia
Years of Education
Smoking Status
Sleep Chronotype
Age at Menarche
Body Mass Index
3
2
1
0
−1
−2
−3
Cell Type
Other
Kidney
Liver
CNS
Hematopoietic
GI
Cardiovascular
Adrenal_Pancreas
SkeletalMuscle
Connective_Bone

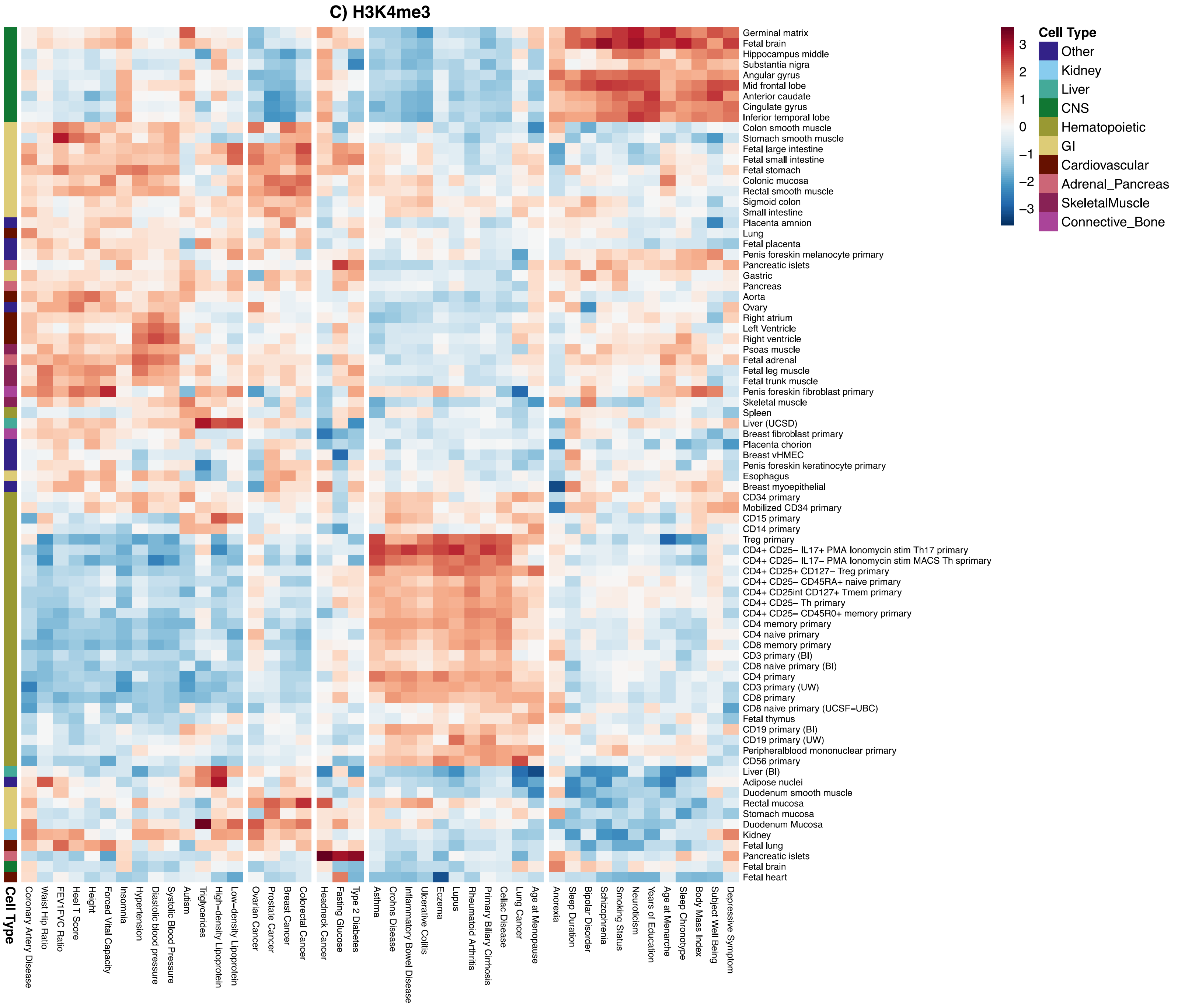

C) H3K4me3
Germinal matrix
Fetal brain
Hippocampus middle
Substantia nigra
Angular gyrus
Mid frontal lobe
Anterior caudate
Cingulate gyrus
Inferior temporal lobe
Colon smooth muscle
Stomach smooth muscle
Fetal large intestine
Fetal small intestine
Fetal stomach
Colonic mucosa
Rectal smooth muscle
Sigmoid colon
Small intestine
Placenta amnion
Lung
Fetal placenta
Penis foreskin melanocyte primary
Pancreatic islets
Gastric
Pancreas
Aorta
Ovary
Right atrium
Left Ventricle
Right ventricle
Psoas muscle
Fetal adrenal
Fetal leg muscle
Fetal trunk muscle
Penis foreskin fibroblast primary
Skeletal muscle
Spleen
Liver (UCSD)
Breast fibroblast primary
Placenta chorion
Breast vHMEC
Penis foreskin keratinocyte primary
Esophagus
Breast myoepithelial
CD34 primary
Mobilized CD34 primary
CD15 primary
CD14 primary
Treg primary
CD4+ CD25− IL17+ PMA Ionomycin stim Th17 primary
CD4+ CD25− IL17− PMA Ionomycin stim MACS Th sprimary
CD4+ CD25+ CD127− Treg primary
CD4+ CD25− CD45RA+ naive primary
CD4+ CD25int CD127+ Tmem primary
CD4+ CD25− Th primary
CD4+ CD25− CD45R0+ memory primary
CD4 memory primary
CD4 naive primary
CD8 memory primary
CD3 primary (BI)
CD8 naive primary (BI)
CD4 primary
CD3 primary (UW)
CD8 primary
CD8 naive primary (UCSF−UBC)
Fetal thymus
CD19 primary (BI)
CD19 primary (UW)
Peripheralblood mononuclear primary
CD56 primary
Liver (BI)
Adipose nuclei
Duodenum smooth muscle
Rectal mucosa
Stomach mucosa
Duodenum Mucosa
Kidney
Fetal lung
Pancreatic islets
Fetal brain
Fetal heart
Cell Type
Coronary Artery Disease
Waist Hip Ratio
FEV1FVC Ratio
Heel T Score
Height
Forced Vital Capacity
Insomnia
Hypertension
Diastolic blood pressure
Systolic Blood Pressure
Autism
Triglycerides
High−density Lipoprotein
Low−density Lipoprotein
Ovarian Cancer
Prostate Cancer
Breast Cancer
Colorectal Cancer
Headcheck Cancer
Fasting Glucose
Type 2 Diabetes
Asthma
Crohns Disease
Inflammatory Bowel Disease
Ulcerative Colitis
Eczema
Lupus
Rheumatoid Arthritis
Primary Biliary Cirrhosis
Celiac Disease
Lung Cancer
Age at Menopause
Anorexia
Sleep Duration
Bipolar Disorder
Schizophrenia
Smoking Status
Neuroticism
Years of Education
Age at Menarche
Sleep Chronotype
Body Mass Index
Subject Well Being
Depressive Symptom
3
2
1
0
−1
−2
−3
Cell Type
Other
Kidney
Liver
CNS
Hematopoietic
GI
Cardiovascular
Adrenal_Pancreas
SkeletalMuscle
Connective_Bone

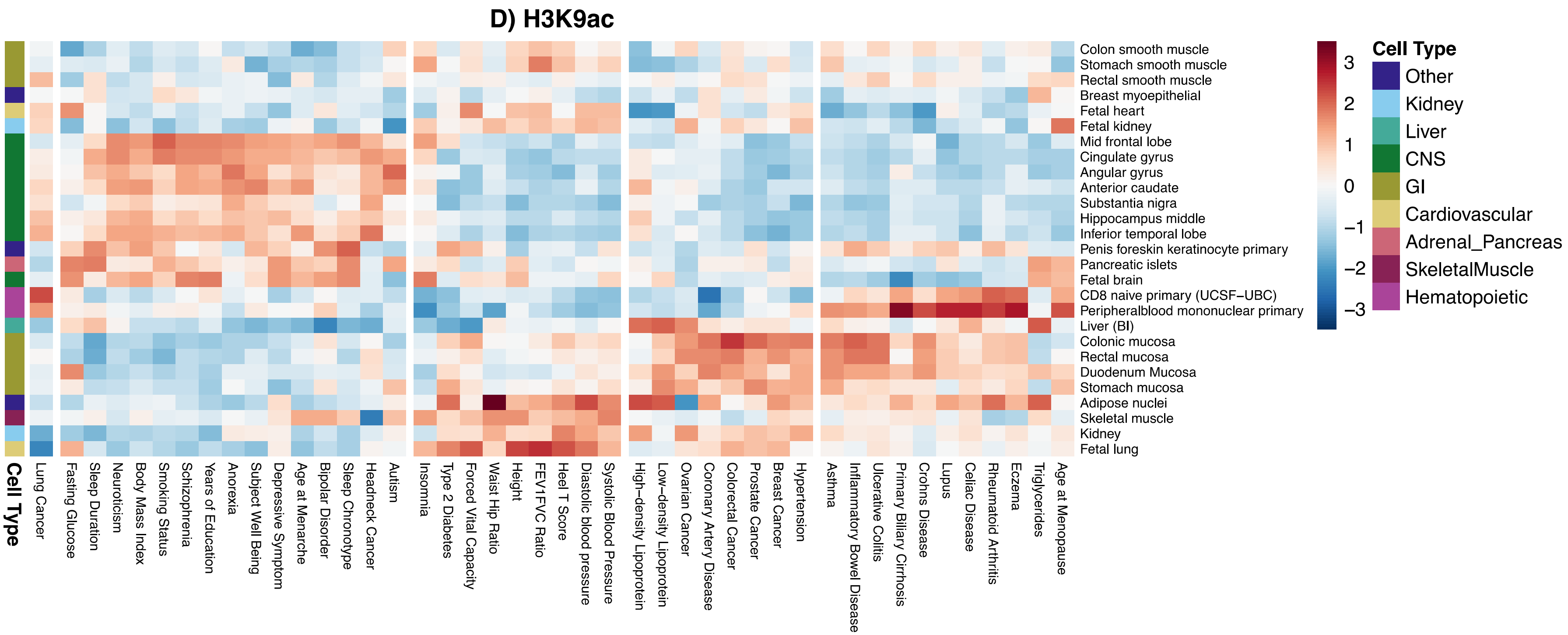

Supplementary Figure 4. Heat-maps showing bi-clustering of traits and cell-types.
We performed 220 cell-type-specific annotation analysis in each of the 38 traits; and compared these enrichment results to the enrichment results of six cancers. Each checker reflects the beta coefficient z-score, scaled by traits. Red indicates enrichment, blue indicates depletion. Deeper color represents stronger magnitude of effects. The category of cell types is color coded to the left. **A)** H3K27ac, **B)** H3K4me1, **C)** H3K4me3 and **D)** H3K9ac. GI: gastrointestinal cell types; CNS: central nervous system cell types.
