# Supplementary material for: Shared heritability and functional enrichment across six solid cancers
Source: Nat Commun. 2019 Jan 25;10:431. doi: 10.1038/s41467-018-08054-4 (PMC6347624; doi:10.1038/s41467-018-08054-4)
Supplement: Supplementary file 2 — Description of Additional Supplementary Files [file 41467_2018_8054_MOESM2_ESM.docx]

Description of Additional Supplementary Files

**Supplementary Data 1.** The number of regions (+/- 500 kb) for each cancer that reach the 5×10-8 threshold (p-values) in each cancer and the best SNP in the region.

**Supplementary Data 2.** The local genetic correlations of reported pleiotropic regions (conferring risks to two or more cancers) in the current analysis using OncoArray data.

**Supplementary Data 3.** Genetic correlations between cancers and non-cancer traits.

**Supplementary Data 4.** Enrichment estimates of the 220 cell-type-specific annotations over four histone marks H3K4me1, H3K4me3, H3K9ac, and H3K27ac, for six cancers.

**Supplementary Data 5.** Enrichment estimates of the 220 cell-type-specific annotations over four histone marks H3K4me1, H3K4me3, H3K9ac, and H3K27ac, for subtypes of cancers
