# Supplementary material for: Shared heritability and functional enrichment across six solid cancers
Source: Nat Commun. 2019 Jan 25;10:431. doi: 10.1038/s41467-018-08054-4 (PMC6347624; doi:10.1038/s41467-018-08054-4)
Supplement: Supplementary file 3 — Supplementary Data 1 [file 41467_2018_8054_MOESM3_ESM.docx]

**Shared heritability and functional enrichment across six solid cancers**

**Jiang et al.**

| **Supplementary Data 1. The number of regions (+/- 500 kb) for each cancer that reach the 5x10^-8^ threshold (p-values) in each cancer and the best SNP in the region.** | | | | | | |
| --- | --- | --- | --- | --- | --- | --- |
| **Chromosome** | **Region start** | **Region end** | **Best SNP** | **Position** | **Z-score** | **Combined P-value** |
| **Breast cancer** | | | | | | |
| 1 | 10011589 | 11138604 | rs2506889 | 10596022 | -9.32 | 1.71E-20 |
| 1 | 18299285 | 19311668 | rs2992756 | 18807339 | 8.00 | 1.58E-15 |
| 1 | 40880440 | 42640601 | rs4233486 | 41380440 | 5.77 | 6.60E-09 |
| 1 | 46100917 | 47103348 | rs1707302 | 46600917 | -5.62 | 2.46E-08 |
| 1 | 50329879 | 51360499 | rs145905639 | 50829879 | -5.53 | 2.92E-08 |
| 1 | 87656923 | 88929024 | rs17426269 | 88156923 | 5.64 | 1.70E-08 |
| 1 | 113673410 | 114951386 | rs7513707 | 114445880 | 6.77 | 1.33E-11 |
| 1 | 117695233 | 118758703 | rs7529522 | 118230221 | -6.37 | 1.54E-10 |
| 1 | 119758970 | 121977667 | rs11249433 | 121280613 | -15.20 | 1.34E-52 |
| 1 | 145088184 | 146213071 | rs36107432 | 145604791 | -6.40 | 1.93E-10 |
| 1 | 149392872 | 150495265 | rs11205303 | 149906413 | -7.77 | 1.05E-14 |
| 1 | 154643768 | 156171678 | rs4971059 | 155148781 | 6.66 | 3.22E-11 |
| 1 | 200937832 | 201937832 | rs35383942 | 201437832 | 7.25 | 3.68E-13 |
| 1 | 203266395 | 204447358 | rs59867004 | 203801249 | -6.46 | 1.19E-10 |
| 1 | 216700580 | 217722804 | rs11117758 | 217220574 | -5.92 | 3.10E-09 |
| 1 | 241523898 | 242547847 | rs72755295 | 242034263 | -7.69 | 1.46E-14 |
| 2 | 9594526 | 10646757 | rs113577745 | 10135681 | -6.27 | 3.33E-10 |
| 2 | 18768232 | 19927429 | rs11684853 | 19310918 | -7.19 | 6.13E-13 |
| 2 | 24577856 | 25973311 | rs6725517 | 25129473 | 7.00 | 2.25E-12 |
| 2 | 120576438 | 121764471 | rs4848599 | 121239360 | -9.31 | 1.35E-20 |
| 2 | 171869881 | 173474566 | rs2016394 | 172972971 | -6.82 | 6.78E-12 |
| 2 | 173698854 | 174719118 | rs2010610 | 174210908 | -6.29 | 3.75E-10 |
| 2 | 201614624 | 202867589 | rs3769821 | 202123430 | -8.74 | 2.04E-18 |
| 2 | 217223569 | 219239300 | rs4442975 | 217920769 | -20.89 | 2.57E-96 |
| 2 | 226711914 | 227743909 | rs12479355 | 227226952 | 5.61 | 2.13E-08 |
| 3 | 4228008 | 5296807 | rs6787391 | 4728574 | 8.81 | 1.03E-18 |
| 3 | 26531928 | 28149948 | rs7626742 | 27268398 | 16.90 | 2.90E-64 |
| 3 | 30167425 | 31189421 | rs17838698 | 30684907 | 7.67 | 1.67E-14 |
| 3 | 46246022 | 47407435 | rs56387622 | 46888198 | 8.95 | 4.64E-19 |
| 3 | 63328780 | 64520447 | rs3821902 | 63941697 | -7.02 | 2.29E-12 |
| 3 | 71017527 | 72044614 | rs6805189 | 71532113 | 5.50 | 3.21E-08 |
| 3 | 86537543 | 87537543 | rs13066793 | 87037543 | 6.12 | 9.71E-10 |
| 3 | 98875151 | 100342140 | rs9833888 | 99723580 | 6.26 | 4.70E-10 |
| 3 | 140533481 | 141836708 | rs7650602 | 141147414 | -7.75 | 5.60E-15 |
| 3 | 171770437 | 172794031 | rs58058861 | 172285237 | 6.41 | 1.72E-10 |
| 4 | 38263103 | 39394380 | rs6815814 | 38816338 | -7.25 | 4.27E-13 |
| 4 | 83864808 | 84956142 | rs9284657 | 84419143 | -5.87 | 3.58E-09 |
| 4 | 88740476 | 89746214 | rs10022462 | 89243818 | 6.06 | 1.30E-09 |
| 4 | 105563987 | 106856761 | rs62331150 | 106069013 | 6.81 | 1.15E-11 |
| 4 | 126343504 | 127343504 | rs77528541 | 126843504 | -6.11 | 9.35E-10 |
| 4 | 175318396 | 176425281 | rs7697216 | 175828036 | -10.70 | 1.27E-26 |
| 5 | -185065 | 1800070 | rs2853669 | 1295349 | 9.42 | 3.98E-21 |
| 5 | 15695004 | 16785704 | rs4702131 | 16233619 | 6.87 | 6.88E-12 |
| 5 | 32067732 | 33081186 | rs12519859 | 32581186 | 5.92 | 4.35E-09 |
| 5 | 43645931 | 46903779 | rs10941679 | 44706498 | -18.21 | 3.40E-73 |
| 5 | 49141645 | 50760139 | rs27279 | 50238519 | -6.57 | 3.86E-11 |
| 5 | 55412533 | 56829225 | rs62355901 | 56053535 | -21.18 | 3.14E-99 |
| 5 | 57684061 | 58895679 | rs1498608 | 58343067 | -6.28 | 2.87E-10 |
| 5 | 80615839 | 82110680 | rs4081859 | 81466669 | 7.21 | 6.67E-13 |
| 5 | 90153868 | 91290451 | rs332529 | 90789470 | -6.86 | 8.63E-12 |
| 5 | 110614379 | 111717786 | rs6882649 | 111217786 | 5.92 | 2.57E-09 |
| 5 | 131875335 | 132944509 | rs56083805 | 132442263 | 5.82 | 6.20E-09 |
| 5 | 157564697 | 158946223 | rs11135046 | 158230013 | -11.58 | 2.52E-31 |
| 5 | 169041551 | 170091487 | rs4562056 | 169591487 | 6.21 | 4.00E-10 |
| 6 | 13138243 | 14245518 | rs418053 | 13713366 | -7.52 | 8.52E-14 |
| 6 | 15899557 | 16899557 | rs3819405 | 16399557 | -5.67 | 1.25E-08 |
| 6 | 20036748 | 21206418 | rs2223621 | 20621238 | 6.38 | 2.47E-10 |
| 6 | 25675866 | 29856687 | rs34546498 | 26961280 | -6.13 | 7.40E-10 |
| 6 | 80572063 | 82887139 | rs9361840 | 82254932 | -7.53 | 5.00E-14 |
| 6 | 129828639 | 130903515 | rs6569648 | 130349119 | 7.05 | 1.75E-12 |
| 6 | 151316054 | 152974790 | rs60954078 | 151955914 | -15.60 | 4.07E-55 |
| 7 | 21440960 | 22440960 | rs7971 | 21940960 | 5.58 | 1.98E-08 |
| 7 | 27856889 | 28856889 | rs17156577 | 28356889 | -5.91 | 3.74E-09 |
| 7 | 90917816 | 92529302 | rs3753107 | 91629151 | -6.60 | 3.60E-11 |
| 7 | 93559899 | 94804344 | rs17268829 | 94113799 | -7.29 | 3.51E-13 |
| 7 | 101000996 | 102068195 | rs71559437 | 101552440 | -6.93 | 4.28E-12 |
| 7 | 130127014 | 131190824 | rs61729633 | 130668912 | 7.20 | 4.43E-13 |
| 7 | 139437791 | 140458544 | rs11977670 | 139942304 | 8.29 | 9.76E-17 |
| 7 | 143548902 | 144639419 | rs62485509 | 144048902 | -6.90 | 3.96E-12 |
| 8 | 28917238 | 30030479 | rs9693444 | 29509616 | 9.62 | 1.34E-21 |
| 8 | 36158914 | 37359186 | rs4286946 | 36849946 | 9.64 | 5.51E-22 |
| 8 | 75668870 | 77189287 | rs72658071 | 76305785 | -11.09 | 1.09E-28 |
| 8 | 101975114 | 102983100 | rs514192 | 102478959 | 5.92 | 3.95E-09 |
| 8 | 105821126 | 106872180 | rs12546444 | 106358620 | 6.50 | 7.22E-11 |
| 8 | 116667843 | 117709548 | rs13267382 | 117209548 | 6.74 | 1.19E-11 |
| 8 | 124059709 | 125257661 | rs58847541 | 124610166 | 7.21 | 4.33E-13 |
| 8 | 127773489 | 129724888 | rs10096351 | 128372172 | -17.05 | 4.97E-65 |
| 9 | 21449527 | 22603183 | rs1985742 | 21961227 | -9.23 | 4.12E-20 |
| 9 | 109797639 | 111573347 | rs630965 | 110885479 | 15.50 | 1.51E-54 |
| 9 | 118655568 | 119988626 | rs1895062 | 119313486 | 7.78 | 8.60E-15 |
| 9 | 128883199 | 129896434 | rs10760444 | 129396434 | -5.82 | 5.98E-09 |
| 9 | 135641870 | 136655000 | rs507666 | 136149399 | 5.58 | 2.73E-08 |
| 10 | 8576366 | 9628818 | rs67801543 | 9108324 | 6.02 | 1.79E-09 |
| 10 | 21189036 | 23415712 | rs7072776 | 22032942 | 9.12 | 1.25E-19 |
| 10 | 63389801 | 65352335 | rs10995201 | 64299890 | 15.15 | 6.79E-52 |
| 10 | 80310343 | 81392739 | rs1268974 | 80852378 | 12.62 | 1.38E-36 |
| 10 | 114232882 | 115764973 | rs12250948 | 115128491 | 7.82 | 5.94E-15 |
| 10 | 122591543 | 124306607 | rs34032268 | 123341525 | -37.49 | 0.00E+00 |
| 11 | 280827 | 1325110 | rs6597981 | 803017 | -7.05 | 1.34E-12 |
| 11 | 1371813 | 2539274 | rs1973765 | 1898664 | 12.98 | 4.78E-38 |
| 11 | 64993112 | 66183531 | rs3903072 | 65583066 | -7.02 | 1.59E-12 |
| 11 | 68422043 | 70009669 | rs78540526 | 69331418 | 24.42 | 2.34E-132 |
| 11 | 128952507 | 129976625 | rs11822830 | 129461016 | -7.70 | 1.25E-14 |
| 12 | 13880438 | 14922475 | rs12422552 | 14413931 | 7.91 | 2.22E-15 |
| 12 | 27517188 | 29774538 | rs7297051 | 28174817 | -16.47 | 1.73E-60 |
| 12 | 84498818 | 85509562 | rs10862899 | 85004551 | 5.48 | 3.91E-08 |
| 12 | 95521033 | 96538543 | rs17356907 | 96027759 | 13.16 | 8.43E-40 |
| 12 | 114602482 | 116338648 | rs2464264 | 115835798 | -13.11 | 8.46E-40 |
| 12 | 120332146 | 121336293 | rs1167362 | 120836293 | -5.60 | 2.24E-08 |
| 13 | 32339990 | 33339990 | rs56404467 | 32839990 | 6.54 | 6.31E-11 |
| 13 | 73306982 | 74467507 | rs6562760 | 73957681 | -6.10 | 1.10E-09 |
| 14 | 36518022 | 37779425 | rs7149262 | 37136545 | -9.53 | 1.82E-21 |
| 14 | 67862510 | 69572051 | rs11624333 | 68979835 | 13.68 | 2.11E-42 |
| 14 | 91242924 | 92490948 | rs941764 | 91841069 | -7.09 | 8.68E-13 |
| 14 | 92569980 | 93618229 | rs117068593 | 93118229 | -7.08 | 1.30E-12 |
| 14 | 104713978 | 105717921 | rs4983544 | 105213978 | -5.60 | 2.42E-08 |
| 15 | 90960302 | 92061182 | rs77554484 | 91509215 | -8.07 | 9.41E-16 |
| 16 | 51982782 | 53180827 | rs4784227 | 52599188 | 30.34 | 1.84E-202 |
| 16 | 53297908 | 55183802 | rs62048402 | 53803223 | -9.86 | 4.09E-23 |
| 16 | 55879792 | 56920987 | rs2432539 | 56420987 | 5.56 | 3.32E-08 |
| 16 | 80141031 | 81176117 | rs7500067 | 80648296 | -10.79 | 1.73E-27 |
| 16 | 86532855 | 87591139 | rs4496150 | 87085237 | -5.76 | 7.85E-09 |
| 17 | 28664023 | 29771319 | rs7223535 | 29211667 | -6.27 | 3.10E-10 |
| 17 | 40244470 | 41336389 | rs72826962 | 40836389 | 5.86 | 4.90E-09 |
| 17 | 42971489 | 45365603 | rs118045117 | 44252468 | -7.21 | 5.01E-13 |
| 17 | 52474643 | 53771918 | rs2787486 | 53209774 | 11.12 | 4.63E-29 |
| 17 | 77268654 | 78304936 | rs8082452 | 77771548 | 6.43 | 9.71E-11 |
| 18 | 23656018 | 25125756 | rs170801 | 24500899 | -8.47 | 1.99E-17 |
| 18 | 29405293 | 30540417 | rs117618124 | 29977689 | 6.94 | 4.42E-12 |
| 18 | 41871256 | 43419925 | rs9954058 | 42411803 | -7.32 | 2.13E-13 |
| 19 | 12658277 | 14456663 | rs78269692 | 13158277 | -6.01 | 1.89E-09 |
| 19 | 16890291 | 17907695 | rs56069439 | 17393925 | 5.81 | 6.73E-09 |
| 19 | 18010767 | 20157632 | rs8105994 | 18593553 | 11.08 | 2.22E-28 |
| 19 | 43781492 | 44932840 | rs1685191 | 44283232 | -9.16 | 2.87E-20 |
| 19 | 45679043 | 46683586 | rs11672660 | 46180184 | 5.88 | 4.06E-09 |
| 20 | 5448227 | 6448227 | rs16991615 | 5948227 | 6.05 | 1.45E-09 |
| 20 | 48439076 | 49477740 | rs6122906 | 48945911 | -6.36 | 1.87E-10 |
| 21 | 15839172 | 17132322 | rs2403907 | 16574455 | -11.85 | 1.47E-32 |
| 22 | 27695386 | 30610546 | rs132289 | 29551872 | 10.36 | 4.75E-25 |
| 22 | 38005356 | 39858037 | rs4820318 | 38570313 | -7.45 | 6.08E-14 |
| 22 | 39969249 | 42538786 | rs5995875 | 40960692 | 12.32 | 4.74E-35 |
| 22 | 45774072 | 46783297 | rs28512361 | 46283297 | 5.61 | 2.03E-08 |
| **Colorectal cancer** | | | | | | |
| 1 | 182581194 | 183582825 | rs6669796 | 183082825 | -5.53 | 2.78E-08 |
| 1 | 221589108 | 222719753 | rs114008224 | 222141545 | -6.30 | 3.08E-10 |
| 2 | 218584082 | 219684301 | rs12053514 | 219167965 | -5.92 | 3.23E-09 |
| 3 | 40406460 | 41510253 | rs35401364 | 40923718 | -7.27 | 3.89E-13 |
| 4 | 94443383 | 95449438 | rs1370821 | 94943383 | 5.50 | 3.99E-08 |
| 5 | 792983 | 1796486 | rs2735940 | 1296486 | -7.26 | 3.13E-13 |
| 5 | 39719972 | 40785970 | rs1445011 | 40280202 | -7.46 | 7.79E-14 |
| 5 | 133967220 | 135022977 | rs4976270 | 134467220 | -6.48 | 1.08E-10 |
| 6 | 35028378 | 36028378 | rs6906359 | 35528378 | -5.52 | 3.43E-08 |
| 6 | 55157261 | 56237971 | rs62404968 | 55714314 | -6.11 | 8.57E-10 |
| 8 | 117099247 | 118305397 | rs16892766 | 117630683 | -10.12 | 3.94E-24 |
| 8 | 127907190 | 128955694 | rs6983267 | 128413305 | -10.69 | 7.74E-27 |
| 10 | 8188998 | 9243313 | rs1537603 | 8734295 | -7.49 | 8.16E-14 |
| 10 | 52145248 | 53146093 | rs10994860 | 52645424 | -5.51 | 3.46E-08 |
| 10 | 80319132 | 81319132 | rs704017 | 80819132 | -5.59 | 1.96E-08 |
| 10 | 100843317 | 101847038 | rs35564340 | 101344263 | 5.91 | 3.64E-09 |
| 11 | 61049025 | 62097972 | rs1535 | 61597972 | 5.50 | 4.15E-08 |
| 11 | 73776167 | 75258059 | rs193143010 | 74656658 | -6.96 | 3.59E-12 |
| 11 | 110619694 | 111681130 | rs3087967 | 111156836 | 9.17 | 5.07E-20 |
| 12 | 3868607 | 4900808 | rs12818766 | 4376091 | 6.88 | 6.74E-12 |
| 12 | 50141572 | 51721127 | rs4307773 | 51144432 | 7.26 | 3.37E-13 |
| 12 | 111333788 | 113184221 | rs653178 | 112007756 | 6.38 | 1.86E-10 |
| 12 | 115375881 | 116436753 | rs7315438 | 115891403 | 6.25 | 4.38E-10 |
| 13 | 33521943 | 34594345 | rs10161980 | 34093518 | 5.86 | 4.66E-09 |
| 14 | 53910919 | 54919110 | rs35107139 | 54419106 | -7.69 | 1.84E-14 |
| 14 | 58683198 | 59711557 | rs17094971 | 59183198 | 6.27 | 3.82E-10 |
| 15 | 32494756 | 33543455 | rs2293582 | 33010412 | 8.85 | 7.21E-19 |
| 16 | 85838288 | 86840448 | rs2696839 | 86340448 | -5.62 | 2.02E-08 |
| 17 | 310559 | 1312534 | rs6598833 | 811968 | 5.69 | 1.12E-08 |
| 18 | 45948805 | 46969962 | rs11874392 | 46453156 | 11.54 | 6.13E-31 |
| 19 | 33004997 | 34024919 | rs8112217 | 33518718 | 6.78 | 1.20E-11 |
| 20 | 5815656 | 7206493 | rs6117251 | 6406440 | -7.57 | 3.68E-14 |
| 20 | 32412050 | 33720070 | rs2295444 | 33173883 | -5.94 | 3.31E-09 |
| 20 | 48478609 | 49563830 | rs1810502 | 49057488 | -5.74 | 1.02E-08 |
| 20 | 60390808 | 61486019 | rs1741640 | 60932414 | -9.03 | 1.89E-19 |
| **Lung cancer** | | | | | | |
| 1 | 77467507 | 79123626 | rs71658797 | 77967507 | 6.63 | 3.25E-11 |
| 3 | 8717383 | 9717383 | rs446975 | 9217383 | -5.86 | 4.68E-09 |
| 5 | 775857 | 1864439 | rs380286 | 1320247 | -11.88 | 1.51E-32 |
| 6 | 25184606 | 33283086 | rs116822326 | 31434111 | 8.91 | 5.29E-19 |
| 6 | 166869897 | 167912048 | rs239935 | 167411788 | 5.69 | 1.29E-08 |
| 8 | 26844719 | 27844719 | rs11780471 | 27344719 | -5.64 | 1.69E-08 |
| 11 | 117608331 | 118628455 | rs1629083 | 118126576 | 5.69 | 1.25E-08 |
| 12 | 498819 | 1572696 | rs7953330 | 998819 | -6.88 | 6.10E-12 |
| 13 | 32468550 | 33739130 | rs11571833 | 32972626 | 8.09 | 6.12E-16 |
| 15 | 46990614 | 48077451 | rs66759488 | 47577451 | 5.55 | 2.83E-08 |
| 15 | 48830854 | 49876624 | rs77468143 | 49376624 | -6.11 | 1.00E-09 |
| 15 | 78211803 | 79715568 | rs55781567 | 78857986 | 21.58 | 3.08E-103 |
| 19 | 40833284 | 41870338 | rs56113850 | 41353107 | -8.91 | 5.02E-19 |
| **Head and neck cancer** | | | | | | |
| 4 | 99739319 | 100762242 | rs1229984 | 100239319 | -7.15 | 8.32E-13 |
| 6 | 31979729 | 33180122 | rs3828805 | 32636120 | 7.45 | 9.62E-14 |
| 10 | 125657446 | 126657446 | rs201982221 | 126157446 | 5.74 | 9.50E-09 |
| **Ovarian cancer** | | | | | | |
| 2 | 176487112 | 177572189 | rs6755777 | 177043226 | -7.55 | 4.31E-14 |
| 3 | 155838528 | 157202477 | rs62274042 | 156435952 | 13.04 | 7.04E-39 |
| 5 | 779790 | 1785974 | rs4449583 | 1284135 | 6.88 | 5.93E-12 |
| 8 | 82159306 | 83161120 | rs78740005 | 82659306 | 6.13 | 8.53E-10 |
| 8 | 128826499 | 130090818 | rs73375000 | 129561866 | -8.09 | 5.81E-16 |
| 9 | 16336724 | 17482414 | rs62543619 | 16914716 | -12.52 | 5.97E-36 |
| 9 | 135649711 | 136655000 | rs635634 | 136155000 | 6.04 | 1.54E-09 |
| 10 | 21306832 | 22771669 | rs7084454 | 21821274 | 6.29 | 3.13E-10 |
| 15 | 91032869 | 92032869 | rs6496746 | 91532869 | -5.49 | 3.97E-08 |
| 17 | 42960181 | 45365603 | rs111960572 | 43563349 | 7.46 | 8.89E-14 |
| 17 | 45469808 | 47055268 | rs7217120 | 46484755 | 7.48 | 7.20E-14 |
| 19 | 16837555 | 17962094 | rs4808075 | 17390291 | 8.56 | 1.09E-17 |
| **Prostate cancer** | | | | | | |
| 1 | 87695672 | 88727120 | rs12139208 | 88213014 | 5.59 | 2.58E-08 |
| 1 | 149748767 | 151474162 | rs1811698 | 150772613 | -7.55 | 5.33E-14 |
| 1 | 153260104 | 156190186 | rs56103503 | 154980351 | 7.95 | 1.81E-15 |
| 1 | 203528177 | 205107227 | rs4245739 | 204518842 | 10.15 | 3.17E-24 |
| 1 | 205153056 | 206262406 | rs823121 | 205724302 | -5.90 | 3.38E-09 |
| 2 | 8097123 | 9098444 | rs62106670 | 8597123 | 5.82 | 7.11E-09 |
| 2 | 9591952 | 11362188 | rs1990613 | 10781975 | 8.56 | 1.59E-17 |
| 2 | 20378105 | 21429067 | rs9306894 | 20878105 | -9.48 | 1.92E-21 |
| 2 | 42955654 | 44354377 | rs7591218 | 43637998 | 9.88 | 2.96E-23 |
| 2 | 62210273 | 64950722 | rs58235267 | 63277843 | -14.87 | 1.11E-49 |
| 2 | 66138815 | 67220444 | rs74702681 | 66652885 | 6.01 | 1.96E-09 |
| 2 | 85192999 | 86390758 | rs2028900 | 85767735 | -10.25 | 6.67E-25 |
| 2 | 111359287 | 112415946 | rs11691517 | 111893096 | 6.98 | 3.51E-12 |
| 2 | 172710952 | 174734547 | rs28485589 | 173303031 | 13.87 | 1.11E-43 |
| 2 | 201624502 | 202630308 | rs6754084 | 202124997 | -5.49 | 3.53E-08 |
| 2 | 237851347 | 238949107 | rs11891348 | 238440449 | -6.98 | 3.98E-12 |
| 2 | 241254433 | 242943157 | rs77482050 | 242139600 | -9.77 | 1.51E-22 |
| 3 | 86466639 | 87974275 | rs17023964 | 87185759 | -14.32 | 3.08E-46 |
| 3 | 106458845 | 107465492 | rs1283104 | 106962521 | -5.73 | 8.81E-09 |
| 3 | 112446985 | 113809549 | rs12629813 | 113284149 | -10.64 | 1.98E-26 |
| 3 | 127210138 | 128784711 | rs11707297 | 127933203 | -11.51 | 5.80E-31 |
| 3 | 140606063 | 141650026 | rs6763927 | 141140366 | -5.74 | 8.47E-09 |
| 3 | 151492162 | 152715437 | rs182314334 | 152004202 | 6.62 | 4.06E-11 |
| 3 | 168593100 | 170660493 | rs78416326 | 170074517 | -17.28 | 5.60E-67 |
| 4 | 73199144 | 75034437 | rs17804499 | 74442349 | -8.03 | 9.15E-16 |
| 4 | 94909802 | 96097814 | rs12510147 | 95521863 | -10.33 | 4.34E-25 |
| 4 | 105434084 | 106928563 | rs10007915 | 106065308 | 14.84 | 8.27E-50 |
| 5 | 728166 | 2399523 | rs2242652 | 1280028 | -15.22 | 3.46E-52 |
| 5 | 43791404 | 44892142 | rs1482680 | 44392142 | -5.89 | 3.58E-09 |
| 5 | 133328356 | 134363352 | rs10793821 | 133836209 | 6.59 | 5.43E-11 |
| 5 | 172455855 | 173459030 | rs9686557 | 172959030 | -6.03 | 1.94E-09 |
| 5 | 177468915 | 178468915 | rs4976790 | 177968915 | 5.80 | 6.73E-09 |
| 6 | 10671163 | 11727328 | rs2018336 | 11217897 | 7.07 | 1.91E-12 |
| 6 | 29218220 | 33526185 | rs114489703 | 31301771 | 7.95 | 1.39E-15 |
| 6 | 34049699 | 35332661 | rs9469899 | 34793124 | 5.83 | 5.27E-09 |
| 6 | 41009901 | 42091125 | rs10947980 | 41525739 | -9.37 | 8.78E-21 |
| 6 | 43194598 | 44195371 | rs4711748 | 43694598 | 5.54 | 3.36E-08 |
| 6 | 75995882 | 76995882 | rs9443189 | 76495882 | 5.47 | 4.68E-08 |
| 6 | 108777908 | 109999259 | rs6941125 | 109287209 | 6.34 | 1.99E-10 |
| 6 | 116593340 | 117786939 | rs339351 | 117200434 | -9.69 | 2.90E-22 |
| 6 | 152858706 | 153950489 | rs6557265 | 153433402 | 9.63 | 1.04E-21 |
| 6 | 159571652 | 161865436 | rs140793115 | 160606525 | 12.84 | 6.48E-38 |
| 7 | 19939334 | 21564647 | rs12155172 | 20994491 | 9.84 | 9.12E-23 |
| 7 | 26704732 | 28538082 | rs10486567 | 27976563 | -14.20 | 2.04E-45 |
| 7 | 40282572 | 41400398 | rs17621345 | 40875192 | 7.53 | 6.72E-14 |
| 7 | 46937072 | 48014360 | rs4724578 | 47482829 | -7.05 | 1.60E-12 |
| 7 | 97134876 | 98595655 | rs4727386 | 97688440 | 13.19 | 1.17E-39 |
| 8 | 22882594 | 24048805 | rs11135766 | 23533623 | -16.14 | 5.09E-59 |
| 8 | 25391729 | 26434684 | rs11135910 | 25892142 | 7.11 | 9.19E-13 |
| 8 | 42973748 | 43973748 | rs8175525 | 43473748 | 7.29 | 3.19E-13 |
| 8 | 127235049 | 129170924 | rs11986220 | 128531689 | 29.10 | 1.10E-187 |
| 9 | 18051961 | 19598265 | rs1048169 | 19055965 | -7.52 | 6.53E-14 |
| 9 | 21541998 | 22541998 | rs17694493 | 22041998 | -6.37 | 2.26E-10 |
| 9 | 33303408 | 34549779 | rs10122495 | 34049779 | -5.69 | 1.34E-08 |
| 9 | 109642648 | 110807810 | rs77334358 | 110256979 | -7.83 | 5.17E-15 |
| 9 | 132049740 | 133097840 | rs1182 | 132576060 | 6.12 | 1.10E-09 |
| 9 | 139673894 | 140673894 | rs118073815 | 140173894 | 6.75 | 1.39E-11 |
| 10 | 334057 | 1452816 | rs34487581 | 897201 | 7.11 | 9.34E-13 |
| 10 | 45003571 | 46648326 | rs7075427 | 46104943 | 7.75 | 1.02E-14 |
| 10 | 47034989 | 48171874 | rs6602880 | 47546323 | -8.52 | 2.10E-17 |
| 10 | 50955870 | 52095095 | rs10993994 | 51549496 | 25.94 | 2.29E-147 |
| 10 | 89692003 | 90695149 | rs1935581 | 90195149 | -5.82 | 6.55E-09 |
| 10 | 103721854 | 105032828 | rs12570611 | 104421679 | -8.22 | 2.54E-16 |
| 10 | 114211755 | 115212154 | rs7094871 | 114712154 | -5.44 | 4.84E-08 |
| 10 | 122251994 | 123554018 | rs1004934 | 122796182 | 7.06 | 1.39E-12 |
| 10 | 126082160 | 127237997 | rs11245446 | 126650696 | -7.00 | 2.05E-12 |
| 11 | 1007512 | 2805799 | rs10840603 | 2233797 | 17.40 | 4.38E-68 |
| 11 | 7047587 | 8047587 | rs61890184 | 7547587 | 5.79 | 6.56E-09 |
| 11 | 61408440 | 62429242 | rs2277283 | 61908440 | -6.27 | 3.03E-10 |
| 11 | 66372320 | 67576064 | rs12785906 | 66951966 | 5.79 | 7.82E-09 |
| 11 | 68311777 | 69963679 | rs12795301 | 68992285 | 20.96 | 3.07E-98 |
| 11 | 75625116 | 76767477 | rs17749618 | 76251818 | 7.16 | 7.48E-13 |
| 11 | 101892380 | 102907191 | rs12285347 | 102396607 | 9.09 | 1.17E-19 |
| 11 | 107643456 | 108857137 | rs1800057 | 108143456 | -5.77 | 8.15E-09 |
| 11 | 113048935 | 114317286 | rs11214775 | 113807181 | -7.89 | 3.93E-15 |
| 11 | 133766372 | 134766372 | rs878987 | 134266372 | -5.46 | 4.77E-08 |
| 12 | 12371099 | 13371099 | rs2066827 | 12871099 | 6.00 | 2.31E-09 |
| 12 | 13905600 | 14923294 | rs10845938 | 14416918 | -7.15 | 9.80E-13 |
| 12 | 47863253 | 50211553 | rs10875943 | 49676010 | -7.91 | 2.35E-15 |
| 12 | 52727384 | 53882160 | rs73110464 | 53312612 | 13.65 | 1.11E-42 |
| 12 | 64484142 | 65581229 | rs7968403 | 65012824 | 6.93 | 3.38E-12 |
| 12 | 89625089 | 90727779 | rs35644221 | 90227779 | -6.86 | 8.14E-12 |
| 12 | 114110142 | 115190513 | rs10774740 | 114666202 | -9.04 | 1.63E-19 |
| 12 | 132551210 | 133636860 | rs7295014 | 133067989 | -6.14 | 9.50E-10 |
| 13 | 73201852 | 74642058 | rs7996468 | 73714290 | -6.92 | 4.37E-12 |
| 14 | 22805649 | 23805649 | rs1004030 | 23305649 | 5.63 | 1.55E-08 |
| 14 | 36587647 | 37641989 | rs11629412 | 37138294 | 6.99 | 2.34E-12 |
| 14 | 52663421 | 53970916 | rs62003551 | 53424320 | 8.59 | 8.46E-18 |
| 14 | 68526379 | 69634264 | rs767127 | 69134264 | -6.48 | 1.00E-10 |
| 14 | 70176661 | 71596344 | rs11158871 | 71091142 | -5.84 | 5.60E-09 |
| 15 | 40377322 | 41469222 | rs4924487 | 40922915 | 5.71 | 1.32E-08 |
| 15 | 55885868 | 56906361 | rs33984059 | 56385868 | 5.72 | 1.10E-08 |
| 15 | 66087581 | 67339282 | rs80326387 | 66705043 | 6.23 | 4.64E-10 |
| 16 | 57151924 | 58193055 | rs11863709 | 57654576 | -6.73 | 1.78E-11 |
| 16 | 81662812 | 82683403 | rs8052913 | 82166181 | -5.65 | 1.71E-08 |
| 16 | 89440386 | 90440386 | rs13332673 | 89940386 | 5.70 | 1.18E-08 |
| 17 | -64104 | 1185640 | rs684232 | 618965 | -10.15 | 4.34E-24 |
| 17 | 7071752 | 8424746 | rs28441558 | 7803118 | -8.28 | 1.02E-16 |
| 17 | 29580257 | 30604598 | rs142444269 | 30098749 | -6.28 | 3.19E-10 |
| 17 | 35541031 | 36719586 | rs11263763 | 36103565 | 25.41 | 3.06E-141 |
| 17 | 45458009 | 48013127 | rs117576373 | 46820676 | 10.41 | 2.03E-25 |
| 17 | 55956120 | 56987636 | rs2680708 | 56456120 | -5.63 | 1.58E-08 |
| 17 | 68550570 | 69743542 | rs9911515 | 69115358 | 20.48 | 6.47E-93 |
| 18 | 51269949 | 52272473 | rs8093601 | 51772473 | 5.57 | 2.31E-08 |
| 18 | 52730859 | 54015052 | rs28607662 | 53230859 | -5.57 | 2.85E-08 |
| 18 | 56241676 | 57246356 | rs12956892 | 56746315 | 5.79 | 7.68E-09 |
| 18 | 72535513 | 73543846 | rs10460109 | 73036165 | 5.51 | 3.48E-08 |
| 18 | 76234830 | 77302013 | rs9959454 | 76770820 | 9.69 | 5.35E-22 |
| 19 | 16680358 | 17728554 | rs11666569 | 17214073 | -5.73 | 8.17E-09 |
| 19 | 31667803 | 32668343 | rs118005503 | 32167803 | -5.78 | 7.31E-09 |
| 19 | 38047277 | 39377997 | rs12610267 | 38744733 | 12.23 | 4.87E-34 |
| 19 | 41475688 | 43200947 | rs74738513 | 41985931 | -10.17 | 1.64E-24 |
| 19 | 50835049 | 51918257 | rs62113212 | 51360840 | -19.04 | 4.26E-81 |
| 20 | 48997045 | 50091337 | rs7274624 | 49563100 | -7.12 | 9.90E-13 |
| 20 | 51917890 | 52971030 | rs6068688 | 52456926 | -8.10 | 4.36E-16 |
| 20 | 60501851 | 61517081 | rs2427347 | 61017081 | -5.56 | 2.76E-08 |
| 20 | 61698236 | 62931824 | rs1058319 | 62374389 | -10.17 | 1.92E-24 |
| 21 | 42271554 | 43415988 | rs145013758 | 42897136 | 7.11 | 1.15E-12 |
| 22 | 19249525 | 20258399 | rs1978060 | 19749525 | -6.84 | 8.54E-12 |
| 22 | 28388939 | 29388939 | rs9625483 | 28888939 | 5.58 | 2.43E-08 |
| 22 | 39902817 | 41442021 | rs6001723 | 40428706 | -7.36 | 2.23E-13 |
| 22 | 42679613 | 44069608 | rs5759167 | 43500212 | -17.79 | 5.55E-71 |
