# Supplementary material for: Shared heritability and functional enrichment across six solid cancers
Source: Nat Commun. 2019 Jan 25;10:431. doi: 10.1038/s41467-018-08054-4 (PMC6347624; doi:10.1038/s41467-018-08054-4)
Supplement: Supplementary file 4 — Supplementary Data 2 [file 41467_2018_8054_MOESM4_ESM.docx]

**Shared heritability and functional enrichment across six solid cancers**

**Jiang et al.**

| **Supplementary Data 2. The local genetic correlations of reported pleiotropic regions (conferring risks to two or more cancers) in the current analysis using OncoArray data.** | | | | | | | |  |
| --- | --- | --- | --- | --- | --- | --- | --- | --- |
| **Locus** | **Literature reports** | |  | **In our analysis** | | | | |
|  | **Index SNP** | **Cancers** |  | **Cancer pairs** | **Region** | **P-values for local genetic correlation** | **Significance** | |
| 1p22.3 | rs17426269, rs56391074 | Breast, Prostate |  | Breast, Prostate | 1:88,128,631-90,066,302 | 0.75 | NS | |
| 1p36.12 | rs72647484, rs3820282 | Colorectal, Ovarian |  | Colorectal, Ovarian | 1:21,736,588-23,086,882 | 0.61 | NS | |
| 1p36.22 | rs616488, rs636291 | Breast, Prostate |  | Breast, Prostate | 1:9,365,199-10,806,983 | 0.0037 | * | |
| 1q32.1 | rs6678914, rs4245739 | Breast, Prostate |  | Breast, Prostate | 1:203,334,734-204,681,067 | 0.0059 | * | |
| 1q41 | rs11117758, rs6691170 | Breast, Colorectal |  | Breast, Colorectal | 1:221,858,231-222,230,219 | 0.15 | NS | |
| 2p23.3 | rs200648189, rs6547741 | Breast, Head/neck |  | Breast, Head/neck | 2:28,598,777-29,217,558 | 0.25 | NS | |
| 2p24.1 | rs12710698, rs13385191 | Breast, Prostate |  | Breast, Prostate | 2:19,692,404-21,050,489 | 0.55 | NS | |
| 2p25.1 | rs113577745, rs62106670 | Breast, Prostate |  | Breast, Prostate | 2:8,422,147-10,298,468 | 0.039 | * | |
| 2q13 | rs71801447, rs2165109, rs11691517 | Breast, Ovarian, Prostate |  | Breast, Ovarian | 2:110,572,432-113,921,855 | 0.026 | * | |
|  |  |  |  | Breast, Prostate | 2:110,572,432-113,921,855 | 0.43 | NS | |
|  |  |  |  | Ovarian, Prostate | 2:110,572,432-113,921,855 | 0.61 | NS | |
| 2q31.1 | rs1550623, rs2016394, rs12621278, rs6755777 | Breast, Ovarian, Prostate |  | Breast, Ovarian | 2:173,138,905-175,588,928 | 0.24 | NS | |
|  |  |  |  | Breast, Prostate | 2:173,138,905-175,588,928 | 0.3 | NS | |
|  |  |  |  | Ovarian, Prostate | 2:175,588,929-177,363,635 | 0.64 | NS | |
| 2q33.1 | rs1830298, rs59308963 | Breast, Prostate |  | Breast, Prostate | 2:201,576,284-202,818,636 | 0.3 | NS | |
| 3p12.1 | rs13066793, rs2660753 | Breast, Prostate |  | Breast, Prostate | 3:87,409,732-88,298,372 | 0.00018 | * | |
| 3q23 | rs34207738, rs112071820, rs6763931 | Breast, Ovarian, Prostate |  | Breast, Ovarian | 3:139,954,597-141,339,096 | 0.67 | NS | |
|  |  |  |  | Breast, Prostate | 3:139,954,597-141,339,096 | 0.00054 | * | |
|  |  |  |  | Prostate, Ovarian | 3:139,954,597-141,339,096 | 0.62 | NS | |
| 3q26.2 | rs10936599, rs10936632 | Colorectal, Prostate |  | Colorectal, Prostate | 3:168,580,960-170,964,908 | 0.25 | NS | |
| 3q28 | rs13080835, rs9870207 | Lung, Ovarian |  | Lung, Ovarian | 3:188,856,214-190,226,606 | 0.97 | NS | |
| 4q21.23 | rs1494961 | Breast, Head/neck |  | Breast, Head/neck | 4:80,990,297-82,125,314 | 0.02 | * | |
| 4q24 | rs7679673, rs9790517 | Breast, Prostate |  | Breast, Prostate | 4:105,305,294-107,501,304 | 0.36 | NS | |
| 5p12 | rs2121875, rs4415084 | Breast, Prostate |  | Breast, Prostate | 5:43,983,499-50,163,397 | 0.45 | NS | |
| 5p15.33 | rs37004, rs2736100, rs10069690, rs2242652, rs401681, rs12653946, rs10462706 | Breast, Head/neck, Lung, Ovarian, Prostate |  | Breast, Head/neck | 5:982,252-2,132,441 | 0.84 | NS | |
|  |  |  |  | Breast, Lung | 5:982,252-2,132,441 | 0.89 | NS | |
|  |  |  |  | Breast, Ovarian | 5:982,252-2,132,441 | 0.62 | NS | |
|  |  |  |  | Breast, Prostate | 5:982,252-2,132,441 | 0.059 | NS | |
|  |  |  |  | Head/neck, Lung | 5:982,252-2,132,441 | 0.058 | NS | |
|  |  |  |  | Head/neck, Ovarian | 5:982,252-2,132,441 | 0.14 | NS | |
|  |  |  |  | Head/neck, Prostate | 5:982,252-2,132,441 | 0.13 | NS | |
|  |  |  |  | Lung, Ovarian | 5:982,252-2,132,441 | 0.017 | * | |
|  |  |  |  | Lung, Prostate | 5:982,252-2,132,441 | 0.23 | NS | |
|  |  |  |  | Ovarian, Prostate | 5:982,252-2,132,441 | 0.023 | * | |
| 5q31.1 | rs6596100, rs647161, rs10793821 | Breast, Colorectal, Prostate |  | Breast, Colorectal | 5:132,139,649-134,777,400 | 0.66 | NS | |
|  |  |  |  | Breast, Prostate | 5:132,139,649-134,777,400 | 0.83 | NS | |
|  |  |  |  | Colorectal, Prostate | 5:132,139,649-134,777,400 | 0.52 | NS | |
| 5q35.1 | rs4562056, rs76551843 | Breast, Prostate |  | Breast, Prostate | 5:168,525,318-169,505,663 | 0.85 | NS | |
| 6p21.32 | rs3828805, rs3096702 | Head/neck, Prostate |  | Head/neck, Prostate | 6:31,571,218-32,682,663 | 0.00093 | * | |
| 6p21.33 | rs116822326, rs12665339 | Lung, Prostate |  | Lung, Prostate | 6:30,798,168-31,571,217 | 5.6X10^-7^ | ** | |
| 6p22.1 | rs9257408, rs4324798, rs115344852, rs115457135 | Breast, Lung, Ovarian, Prostate |  | Breast, Lung | 6:28,017,819-28,917,607 | 3.2X10^-6^ | ** | |
|  |  |  |  | Breast, Ovarian | 6:28,017,819-28,917,607 | 0.028 | * | |
|  |  |  |  | Breast, Prostate | 6:28,017,819-28,917,607 | 0.001 | * | |
|  |  |  |  | Lung, Ovarian | 6:28,017,819-28,917,607 | 0.36 | NS | |
|  |  |  |  | Lung, Prostate | 6:28,017,819-28,917,607 | 0.0003 | * | |
|  |  |  |  | Ovarian, Prostate | 6:28,017,819-28,917,607 | 0.22 | NS | |
| 6q25.2 | rs2747652, rs1933488 | Breast, Prostate |  | Breast, Prostate | 6:153,094,496-154,974,119 | 0.23 | NS | |
| 6q25.3 | rs9364554, rs651164, rs7758229 | Colorectal, Prostate |  | Colorectal, Prostate | 6:160,580,497-162,169,563 | 0.45 | NS | |
| 7p15.1 | rs17156577, rs10486567 | Breast, Prostate |  | Breast, Prostate | 7:25,909,555-28,360,308 | 0.031 | * | |
| 7p15.3 | rs7971, rs12155172 | Breast, Prostate |  | Breast, Prostate | 7:20,124,908-22,507,628 | 0.27 | NS | |
| 7q21.3 | rs17268829, rs6465657 | Breast, Prostate |  | Breast, Prostate | 7:93,966,601-96,073,507 | 0.62 | NS | |
| 8p12 | rs9693444, rs4236709 | Breast, Lung |  | Breast, Lung | 8:29,327,896-31,133,728 | 0.09 | NS | |
| 8p21.2 | rs11780471, rs11135910 | Lung, Prostate |  | Lung, Prostate | 8:26,682,525-28,162,391 | 0.29 | NS | |
| 8q21.13 | rs6472903, rs11782652 | Breast, Ovarian |  | Breast, Ovarian | 8:73,817,199-75,445,063 | 0.62 | NS | |
| 8q23.3 | rs13267382, rs16892766 | Breast, Colorectal |  | Breast, Colorectal | 8:116,096,495-119,685,456 | 0.97 | NS | |
| 8q24 | rs10505477, rs6983267, rs10808556 | Breast, Colorectal, Prostate |  | Breast, Colorectal | 8:126,410,917-128,659,110 | 0.68 | NS | |
|  |  |  |  | Breast, Prostate | 8:126,410,917-128,659,110 | 0.0075 | * | |
|  |  |  |  | Colorectal, Prostate | 8:126410917-128659110 | 1.9X10^-12^ | ** | |
| 9p21 | rs1011970, rs1333040, rs8181047 | Breast, Lung, Head/neck |  | Breast, Head/neck | 9:20,463,534-22,206,558 | 0.11 | NS | |
|  |  |  |  | Breast, Lung | 9:20,463,534-22,206,558 | 0.0089 | * | |
|  |  |  |  | Lung, Head/neck | 9:20,463,534-22,206,558 | 0.23 | NS | |
| 9q31.2 | rs10759243, rs817826, rs865685 | Breast, Prostate |  | Breast, Prostate | 9:109,298,754-110,695,061 | 0.17 | NS | |
| 10p12 | rs7084454, rs7072776 | Breast, Ovarian |  | Breast, Ovarian | 10:19,716,878-22,772,282 | 0.00051 | * | |
| 10p14 | rs67958007, rs11255841 | Breast, Colorectal |  | Breast, Colorectal | 10:8,774,665-10,249,395 | 0.64 | NS | |
| 10q22.3 | rs704010, rs704017 | Breast, Colorectal |  | Breast, Colorectal | 10:78,706,814-80,876,748 | 0.19 | NS | |
| 10q24.33 | rs11591710, rs7902587 | Lung, Ovarian |  | Lung, Ovarian | 8:101,620,958-104,123,305 | 0.2 | NS | |
| 10q25.2 | rs7904519, rs12241008, rs7094871 | Breast, Colorectal, Prostate |  | Breast, Colorectal | 10:112,561,493-115,328,431 | 0.15 | NS | |
|  |  |  |  | Breast, Prostate | 10:112,561,493-115,328,431 | 0.0054 | * | |
|  |  |  |  | Colorectal, Prostate | 10:112,561,493-115,328,431 | 0.64 | NS | |
| 10q26.13 | rs4962416, rs11199914, rs11199874, rs2981582, rs201982221 | Breast, Prostate, Head/neck |  | Breast, Head/neck | 10:123,231,465-123,900,544 | 0.32 | NS | |
|  |  |  |  | Breast, Prostate | 10:123,231,465-123,900,544 | 1.0X10^-7^ | ** | |
|  |  |  |  | Head/neck, Prostate | 10:125,869,346-128,001,097 | 0.1 | NS | |
| 11p15.4 | rs1453414, rs61890184 | Head/neck, Prostate |  | Head/neck, Prostate | 11:6,322,869-7,436,700 | 0.87 | NS | |
| 11p15.5 | rs7127900, rs3817198 | Breast, Prostate |  | Breast, Prostate | 11:1,213,590-3,665,480 | 0.11 | NS | |
| 11q13 | rs3903072, rs7931342, rs554219, rs494406, rs75915166 | Breast, Prostate |  | Breast, Prostate | 11:68,005,825-69,516,129 | 5.7X10^-5^ | * | |
| 12q13.12, 12q13.13 | rs902774, rs11169552, rs10875943 | Colorectal, Prostate |  | Colorectal, Prostate | 12:49,001,866-51,776,493 | 0.16 | NS | |
| 11q22.3 | rs11374964, rs148883465, rs1800057 | Breast, Prostate |  | Breast, Prostate | 11:108,437,037-109,866,115 | 0.33 | NS | |
| 12p13.1 | rs12422552, rs2066827 | Breast, Prostate |  | Breast, Prostate | 12:12,733,528-15,241,483 | 0.23 | NS | |
| 12q24.21 | rs1292011, rs1270884 | Breast, Prostate |  | Breast, Prostate | 12:115,503,216-117,087,470 | 0.023 | * | |
| 12q24.31 | rs206966, rs7953249 | Breast, Ovarian |  | Breast, Ovarian | 12:119,754,110-122,007,650 | 0.022 | * | |
| 13q13.1 | rs11571833, rs11571818 | Breast, Lung |  | Breast, Lung | 13:32,301,815-33,776,902 | 0.42 | NS | |
| 14q13.3 | rs2236007, rs11629412 | Breast, Prostate |  | Breast, Prostate | 14:35,859,593-38,667,724 | 0.00098 | * | |
| 14q24 | rs2588809, rs999737, rs7141529 1 | Breast, Prostate |  | Breast, Prostate | 14:67,992,317-71,131,956 | 0.0059 | * | |
| 16q21 | rs8044477, rs11863709 | Ovarian, Prostate |  | Ovarian, Prostate | 16:57,664,330-59,045,404 | 0.22 | NS | |
| 17p13.3 | rs12603526, rs684232 | Colorectal, Prostate |  | Colorectal, Prostate | 17:56-1,172,398 | 0.67 | NS | |
| 17q11.2 | rs142444269 | Breast, Prostate |  | Breast, Prostate | 17:27,334,244-29,786,490 | 0.35 | NS | |
| 17q12 | rs4430796, rs11649743, rs757210, rs7501939 | Ovarian, Prostate |  | Ovarian, Prostate | 17:34,469,036-36,809,343 | 0.00072 | * | |
| 17q21.31 | rs2532263, rs1879586 | Breast, Ovarian |  | Breast, Ovarian | 17:43,056,905-45,876,021 | 0.033 | * | |
| 17q21.32 | rs11650494, rs7207826, rs138213197, rs146746174 | Ovarian, Prostate |  | Ovarian, Prostate | 17:45,876,022-47,517,399 | 0.018 | * | |
| 17q22 | rs2787486, rs2680708 | Breast, Prostate |  | Breast, Prostate | 17:55,357,541-57,487,511 | 0.09 | NS | |
| 18q11.2 | rs527616, rs8098244 | Breast, Ovarian |  | Breast, Ovarian | 18:24,026,191-25,927,681 | 0.1 | NS | |
| 18q22.3 | rs12970291, rs10460109 | Colorectal, Prostate |  | Colorectal, Prostate | 18:74,813,815-75,976,316 | 0.97 | NS | |
| 19p13.11 | rs2363956, rs4808075 | Breast, Ovarian |  | Breast, Ovarian | 19:16,374,416-18,409,861 | 0.0029 | * | |
| 19q12 | rs113701136, rs118005503 | Breast, Prostate |  | Breast, Prostate | 19:30,727,954-32,746,519 | 0.45 | NS | |
| 19q13.2 | rs1800469, rs56113850, rs688187, rs8102476 | Colorectal, Lung, Ovarian, Prostate |  | Colorectal, Lung | 19:40,984,601-42,131,572 | 0.11 | NS | |
|  |  |  |  | Colorectal, Ovarian | 19:40,984,601-42,131,572 | 0.49 | NS | |
|  |  |  |  | Colorectal, Prostate | 19:40,984,601-42,131,572 | 0.18 | NS | |
|  |  |  |  | Lung, Ovarian | 19:40,984,601-42,131,572 | 0.55 | NS | |
|  |  |  |  | Lung, Prostate | 19:40,984,601-42,131,572 | 0.77 | NS | |
|  |  |  |  | Ovarian, Prostate | 19:40,984,601-42,131,572 | 0.98 | NS | |
| 20p12.3 | rs16991615, rs2423279 | Breast, Colorectal |  | Breast, Colorectal | 20:7,084,073-8,117,010 | 0.05 | * | |
| 20q13.13 | rs6122906, rs6066825, rs12480328 | Breast, Colorectal, Prostate |  | Breast, Colorectal | 20:47,199,980-49,239,657 | 0.01 | * | |
|  |  |  |  | Breast, Prostate | 20:47,199,980-49,239,657 | 0.06 | NS | |
|  |  |  |  | Colorectal, Prostate | 20:47,199,980-49,239,657 | 0.027 | * | |
| 20q13.33 | rs2427308, rs41309931, rs2427345 | Colorectal, Lung, Prostate |  | Colorectal, Lung | 20:62,190,180-62,965,162 | 0.57 | NS | |
|  |  |  |  | Colorectal, Prostate | 20:62,190,180-62,965,162 | 0.7 | NS | |
|  |  |  |  | Lung, Prostate | 20:62,190,180-62,965,162 | 2.84X10^-6^ | ** | |
| 22q12 | rs17728461, rs132390, rs17879961, rs36500 | Breast, Lung |  | Breast, Lung | 22:27,834,752-29,651,798 | 0.79 | NS | |
| 22q13.1 | rs738321, rs9623117 | Breast, Prostate |  | Breast, Prostate | 22:37,570,269-39,307,893 | 0.02 | * | |
| 22q13.2 | rs73161324, rs5759167 | Breast, Prostate |  | Breast, Prostate | 22:42,690,818-43,714,199 | 0.016 | * | |
| NS: not significant, * P < 0.05, **: P < 0.05/1703 regions over the 22 chromosomes. Index SNPs are the most strongly associated SNP with one or more common cancers. | | | | | | | |  |
