# Supplementary material for: Shared heritability and functional enrichment across six solid cancers
Source: Nat Commun. 2019 Jan 25;10:431. doi: 10.1038/s41467-018-08054-4 (PMC6347624; doi:10.1038/s41467-018-08054-4)
Supplement: Supplementary file 5 — Supplementary Data 3 [file 41467_2018_8054_MOESM5_ESM.docx]

**Shared heritability and functional enrichment across six solid cancers**

**Jiang et al.**

| **Supplementary Data 3. Genetic correlations between cancers and non-cancer traits.** | | | | | | | | | | | | | | | | | | | |
| --- | --- | --- | --- | --- | --- | --- | --- | --- | --- | --- | --- | --- | --- | --- | --- | --- | --- | --- | --- |
| **Traits** | | **Cancer Types** | | | | | | | | | | | | | | | | | |
|  |  | **Breast cancer** | | |  | **Colorectal cancer** |  | **Head and neck cancer** |  | **Lung cancer** | | | |  | **Ovarian cancer** | |  | **Prostate cancer** | |
|  |  | **Overall** | **ER-positive** | **ER-negative** |  | **Overall** |  | **Overall** |  | **Overall** | **Adenocarcinoma** | **Ever smoking** | **Squamous carcinoma** |  | **Overall** | **Serous invasive** |  | **Overall** | **Advanced stage** |
| Autoimmune inflammatory diseases | Asthma | -0.012 (0.033), 0.71 | -0.045 (0.036), 0.21 | 0.043 (0.053), 0.42 |  | 0.018 (0.056), 0.75 |  | 0.027 (0.087), 0.76 |  | 0.0039 (0.059), 0.95 | -0.079 (0.069), 0.25 | -0.034 (0.072), 0.64 | 0.06 (0.076), 0.43 |  | -0.12 (0.074), 0.11 | -0.08 (0.072), 0.26 |  | -0.022 (0.040), 0.57 | -0.013 (0.059), 0.83 |
|  | Celiac Disease | -0.030 (0.053), 0.57 | -0.014 (0.056), 0.80 | -0.0059 (0.074), 0.94 |  | -0.14 (0.096), 0.15 |  | -0.026 (0.16), 0.87 |  | -0.18 (0.079), 0.019 | -0.11 (0.095), 0.26 | -0.13 (0.12), 0.26 | -0.30 (0.13), 0.015 |  | -0.11 (0.11), 0.32 | -0.11 (0.11), 0.27 |  | 0.10 (0.052), 0.043 | 0.066 (0.079), 0.41 |
|  | Crohn’s Disease | 0.048 (0.037), 0.20 | 0.047 (0.041), 0.25 | 0.019 (0.052), 0.71 |  | -0.088 (0.063), 0.16 |  | 0.17 (0.099), 0.084 |  | 0.18 (0.062), 0.0038 | 0.18 (0.072), 0.013 | 0.17 (0.080), 0.031 | 0.16 (0.082), 0.046 |  | -0.0042 (0.075), 0.96 | -0.0072 (0.071), 0.92 |  | 0.020 (0.040), 0.61 | 0.073 (0.054), 0.18 |
|  | Primary Biliary Cirrhosis | 0.059 (0.046), 0.20 | 0.078 (0.050), 0.12 | 0.037 (0.066), 0.57 |  | -0.011 (0.080), 0.89 |  | 0.18 (0.14), 0.20 |  | -0.014 (0.066), 0.83 | -0.07 (0.093), 0.45 | -0.058 (0.086), 0.50 | -0.042 (0.097), 0.67 |  | 0.13 (0.10), 0.22 | 0.076 (0.97), 0.43 |  | 0.0042 (0.054), 0.94 | -0.038 (0.081), 0.64 |
|  | Eczema | 0.061 (0.033), 0.070 | 0.052 (0.036), 0.15 | 0.078 (0.046), 0.088 |  | -0.084 (0.055), 0.12 |  | -0.10 (0.091), 0.26 |  | -0.17 (0.053), 0.0019 | -0.11 (0.065), 0.097 | -0.17 (0.069), 0.013 | -0.22 (0.071), 0.0018 |  | -0.056 (0.073), 0.45 | -0.054 (0.067), 0.42 |  | 0.0036 (0.040), 0.93 | 0.0058 (0.057), 0.92 |
|  | Lupus | 0.065 (0.047), 0.16 | 0.057 (0.048), 0.24 | 0.060 (0.076), 0.43 |  | -0.060 (0.069), 0.39 |  | -0.039 (0.12), 0.734 |  | -0.0082 (0.069), 0.91 | -0.093 (0.091), 0.30 | -0.071 (0.091), 0.43 | 0.096 (0.086), 0.27 |  | 0.018 (0.10), 0.86 | 0.021 (0.097), 0.83 |  | 0.031 (0.055), 0.57 | 0.051 (0.087), 0.56 |
|  | Ulcerative Colitis | 0.081 (0.044), 0.063 | 0.066 (0.048), 0.17 | 0.074 (0.069), 0.28 |  | -0.051 (0.068), 0.45 |  | 0.18 (0.12), 0.14 |  | 0.013 (0.062), 0.83 | 0.069 (0.076), 0.36 | -0.020 (0.076), 0.79 | -0.076 (0.089), 0.39 |  | 0.15 (0.099), 0.13 | 0.11 (0.10), 0.25 |  | 0.0088 (0.049), 0.86 | -0.052 (0.071), 0.46 |
|  | Inflammatory Bowel Disease | 0.11 (0.032), 0.00080 | 0.079 (0.034), 0.020 | 0.065 (0.047), 0.17 |  | -0.077 (0.055), 0.16 |  | 0.16 (0.089), 0.074 |  | 0.13 (0.055), 0.016 | 0.15 (0.062), 0.017 | 0.086 (0.065), 0.19 | 0.087 (0.064), 0.18 |  | 0.068 (0.069), 0.33 | 0.045 (0.066), 0.50 |  | 0.0078 (0.037), 0.83 | 0.015 (0.049), 0.76 |
|  | Rheumatoid Arthritis | 0.13 (0.045), 0.0039 | 0.082 (0.049), 0.092 | 0.17 (0.057), 0.0036 |  | -0.018 (0.068), 0.79 |  | 0.19 (0.10), 0.074 |  | 0.11 (0.077), 0.17 | 0.12 (0.082), 0.14 | 0.031 (0.075), 0.67 | -0.043 (0.092), 0.64 |  | 0.15 (0.089), 0.092 | 0.13 (0.082), 0.11 |  | -0.0051 (0.045), 0.91 | 0.046 (0.063), 0.47 |
| Metabolic traits | Type 2 Diabetes | -0.0011 (0.046), 0.98 | 0.0031 (0.047), 0.95 | -0.11 (0.070), 0.11 |  | 0.12 (0.073), 0.098 |  | -0.022 (0.13), 0.86 |  | -0.02 (0.069), 0.77 | -0.16 (0.083), 0.058 | -0.0032 (0.086), 0.97 | -0.077 (0.10), 0.44 |  | -0.084 (0.10), 0.41 | -0.10 (0.088), 0.28 |  | 0.0077 (0.049), 0.87 | 0.042 (0.072), 0.56 |
|  | Body Mass Index | -0.055 (0.026), 0.031 | -0.041 (0.026), 0.11 | -0.040 (0.035), 0.25 |  | **0.15 (0.033), 0.0000056** |  | 0.039 (0.052), 0.45 |  | 0.069 (0.035), 0.051 | -0.024 (0.040), 0.55 | 0.035 (0.039), 0.37 | 0.12 (0.041), 0.0045 |  | -0.031 (0.045), 0.49 | -0.059 (0.041), 0.16 |  | -0.069 (0.025), 0.0056 | -0.0073 (0.036), 0.84 |
|  | Hypertension | 0.0041 (0.024), 0.87 | 0.020 (0.026), 0.43 | 0.059 (0.037), 0.11 |  | 0.054 (0.045), 0.23 |  | -0.034 (0.076), 0.66 |  | 0.026 (0.046), 0.57 | -0.077 (0.053), 0.14 | 0.0078 (0.053), 0.88 | 0.063 (0.067), 0.34 |  | 0.051 (0.064), 0.42 | -0.024 (0.06), 0.67 |  | -0.020 (0.033), 0.54 | 0.034 (0.049), 0.49 |
|  | Diastolic Blood Pressure | 0.0054 (0.024), 0.82 | 0.012 (0.025), 0.62 | 0.021 (0.036), 0.56 |  | -0.0045 (0.042), 0.91 |  | -0.002 (0.076), 0.98 |  | 0.0074 (0.041), 0.86 | 0.021 (0.045), 0.65 | -0.025 (0.049), 0.61 | -0.012 (0.059), 0.85 |  | 0.036 (0.058), 0.53 | 0.023 (0.049), 0.65 |  | 0.068 (0.030), 0.023 | 0.082 (0.046), 0.072 |
|  | Systolic Blood Pressure | 0.0098 (0.024), 0.69 | 0.0082 (0.025), 0.75 | 0.052 (0.035), 0.14 |  | 0.011 (0.042), 0.80 |  | 0.013 (0.068), 0.84 |  | 0.0006 (0.040), 0.99 | 0.0031 (0.045), 0.94 | -0.0073 (0.046), 0.87 | 0.015 (0.059), 0.80 |  | 0.042 (0.057), 0.46 | 0.011 (0.052), 0.83 |  | 0.039 (0.029), 0.18 | 0.0017 (0.044), 0.97 |
|  | Coronary Artery Disease | 0.008 (0.043), 0.85 | 0.0038 (0.047), 0.94 | 0.029 (0.062), 0.64 |  | 0.036 (0.067), 0.59 |  | 0.033 (0.13), 0.79 |  | 0.12 (0.073), 0.010 | 0.066 (0.083), 0.42 | 0.10 (0.095), 0.29 | 0.13 (0.098), 0.18 |  | 0.0039 (0.099), 0.97 | 0.062 (0.090), 0.49 |  | 0.081 (0.054), 0.13 | 0.061 (0.080), 0.44 |
|  | Fasting Glucose | 0.036 (0.037), 0.34 | 0.028 (0.041), 0.49 | 0.051 (0.057), 0.37 |  | 0.0083 (0.066), 0.90 |  | 0.38 (0.12), 0.0015 |  | 0.19 (0.058), 0.00081 | 0.040 (0.064), 0.53 | 0.15 (0.074), 0.036 | 0.36 (0.095), 0.00013 |  | -0.0061 (0.091), 0.95 | -0.051 (0.084), 0.55 |  | -0.094 (0.050), 0.061 | -0.12 (0.070), 0.091 |
|  | Waist Hip Ratio adjusted for BMI | 0.05 (0.027), 0.060 | 0.031 (0.030), 0.30 | 0.12 (0.035), 0.00074 |  | 0.080 (0.042), 0.057 |  | 0.050 (0.065), 0.45 |  | **0.16 (0.038), 0.000019** | 0.12 (0.046), 0.0070 | 0.17 (0.049), 0.00074 | 0.13 (0.052), 0.015 |  | -0.0066 (0.055), 0.90 | 0.059 (0.053), 0.26 |  | -0.050 (0.030), 0.091 | -0.078 (0.045), 0.085 |
|  | Triglycerides | -0.024 (0.021), 0.26 | -0.030 (0.024), 0.20 | -0.015 (0.030), 0.60 |  | 0.058 (0.040), 0.15 |  | -0.037 (0.052), 0.48 |  | 0.077 (0.038), 0.042 | 0.052 (0.042), 0.21 | 0.051 (0.048), 0.29 | 0.12 (0.054), 0.023 |  | 0.073 (0.051), 0.15 | 0.028 (0.047), 0.54 |  | -0.017 (0.029), 0.56 | 0.0048 (0.037), 0.90 |
|  | High-density Lipoprotein | 0.043 (0.022), 0.052 | 0.039 (0.024), 0.11 | 0.051 (0.030), 0.085 |  | -0.09 (0.038), 0.019 |  | 0.088 (0.064), 0.17 |  | **-0.14 (0.035), 0.000058** | -0.081 (0.041), 0.046 | -0.15 (0.047), 0.0010 | -0.14 (0.045), 0.0026 |  | -0.058 (0.062), 0.35 | -0.084 (0.054), 0.12 |  | 0.026 (0.024), 0.27 | -0.019 (0.034), 0.58 |
|  | Low-density Lipoprotein | 0.051 (0.027), 0.058 | 0.030 (0.029), 0.29 | 0.074 (0.036), 0.040 |  | 0.10 (0.047), 0.032 |  | 0.15 (0.070), 0.026 |  | 0.049 (0.038), 0.20 | 0.022 (0.045), 0.62 | 0.077 (0.047), 0.10 | 0.11 (0.054), 0.035 |  | 0.032 (0.056), 0.57 | 0.007 (0.049), 0.89 |  | 0.042 (0.031), 0.18 | 0.049 (0.043), 0.25 |
| Others | Age at Menopause | -0.011 (0.033), 0.74 | 0.0005 (0.035), 0.99 | -0.097 (0.050), 0.052 |  | -0.13 (0.060), 0.028 |  | -0.23 (0.10), 0.024 |  | **-0.25 (0.054), 0.0000046** | -0.21 (0.068), 0.0021 | -0.16 (0.068), 0.018 | -0.20 (0.076), 0.0084 |  | 0.056 (0.077), 0.46 | 0.08 (0.073), 0.28 |  | -0.041 (0.039), 0.29 | -0.030 (0.064), 0.64 |
|  | Age at Menarche | -0.029 (0.026), 0.27 | 0.0059 (0.028), 0.83 | -0.085 (0.038), 0.025 |  | -0.020 (0.04), 0.61 |  | 0.036 (0.066), 0.59 |  | -0.023 (0.041), 0.58 | -0.027 (0.049), 0.58 | -0.032 (0.055), 0.56 | 0.0039 (0.058), 0.95 |  | -0.12 (0.063), 0.051 | -0.10 (0.05), 0.071 |  | -0.01 (0.033), 0.76 | 0.031 (0.045), 0.50 |
|  | Heel T Score | -0.017 (0.023), 0.48 | -0.0072 (0.024), 0.76 | -0.036 (0.035), 0.30 |  | -0.078 (0.040), 0.052 |  | -0.058 (0.061), 0.34 |  | -0.061 (0.035), 0.085 | -0.062 (0.047), 0.19 | -0.039 (0.041), 0.35 | -0.063 (0.047), 0.18 |  | -0.015 (0.046), 0.75 | -0.014 (0.045), 0.75 |  | 0.0002 (0.028), 0.99 | -0.061 (0.045), 0.17 |
|  | Forced Vital Capacity | -0.031 (0.023), 0.18 | -0.0042 (0.024), 0.86 | **-0.12 (0.030), 0.000059** |  | -0.048 (0.038), 0.21 |  | -0.059 (0.062), 0.34 |  | -0.086 (0.036), 0.017 | 0.013 (0.045), 0.78 | -0.054 (0.054), 0.32 | -0.097 (0.052), 0.061 |  | -0.066 (0.048), 0.17 | -0.073 (0.044), 0.095 |  | 0.040 (0.035), 0.26 | 0.045 (0.048), 0.35 |
|  | Lung FEV1/FVC Ratio | 0.016 (0.025), 0.53 | 0.014 (0.027), 0.60 | -0.039 (0.034), 0.26 |  | 0.050 (0.041), 0.22 |  | -0.12 (0.061), 0.052 |  | **-0.15 (0.035), 0.000015** | -0.147 (0.043), 0.00053 | -0.16 (0.047), 0.00076 | -0.14 (0.056), 0.012 |  | 0.12 (0.056), 0.035 | 0.11 (0.049), 0.030 |  | -0.010 (0.029), 0.73 | -0.036 (0.042), 0.38 |
|  | Years of Education | -0.051 (0.021), 0.014 | -0.030 (0.023), 0.19 | **-0.17 (0.030), 0.0000000092** |  | **-0.17 (0.037), 0.0000046** |  | **-0.42 (0.068), 0.00000000060** |  | **-0.39 (0.044), 1.54E-18** | **-0.28 (0.045), 0.0000000012** | **-0.32 (0.055), 0.0000000036** | **-0.41 (0.058), 0.0000000000021** |  | -0.11 (0.051), 0.036 | -0.17 (0.046), 0.0003 |  | 0.046 (0.023), 0.046 | -0.021 (0.033), 0.53 |
|  | Smoking Status | 0.0015 (0.024), 0.95 | -0.026 (0.025), 0.30 | 0.093 (0.039), 0.017 |  | 0.10 (0.044), 0.020 |  | **0.47 (0.082), 0.000000013** |  | **0.56 (0.058), 3.45E-22** | **0.44 (0.057), 0.000000000000012** | **0.43 (0.067), 0.000000000064** | **0.58 (0.068), 1.21E-17** |  | 0.006 (0.058), 0.92 | 0.070 (0.054), 0.20 |  | -0.062 (0.033), 0.063 | -0.0084 (0.052), 0.87 |
|  | Height | 0.041 (0.024), 0.083 | 0.057 (0.024), 0.016 | 0.037 (0.032), 0.25 |  | -0.0007 (0.034), 0.98 |  | -0.19 (0.057), 0.0011 |  | -0.027 (0.032), 0.41 | -0.021 (0.038), 0.58 | 0.0091 (0.044), 0.83 | -0.013 (0.040), 0.75 |  | 0.062 (0.049), 0.21 | 0.018 (0.043), 0.68 |  | 0.006 (0.023), 0.79 | 0.048 (0.033), 0.15 |
|  | Sleep Chronotype | 0.037 (0.027), 0.17 | 0.031 (0.028), 0.27 | 0.057 (0.040), 0.15 |  | -0.035 (0.047), 0.45 |  | -0.050 (0.083), 0.55 |  | -0.051 (0.044), 0.25 | 0.0069 (0.055), 0.90 | 0.020 (0.056), 0.72 | -0.056 (0.051), 0.27 |  | -0.070 (0.071), 0.33 | -0.052 (0.066), 0.43 |  | 0.031 (0.031), 0.31 | 0.076 (0.052), 0.14 |
|  | Sleep Duration | 0.050 (0.04), 0.21 | 0.036 (0.044), 0.40 | 0.050 (0.059), 0.40 |  | -0.039 (0.062), 0.54 |  | -0.11 (0.10), 0.29 |  | -0.027 (0.062), 0.67 | -0.036 (0.074), 0.63 | -0.050 (0.081), 0.54 | -0.061 (0.071), 0.39 |  | -0.025 (0.083), 0.76 | -0.048 (0.082), 0.56 |  | 0.10 (0.043), 0.018 | 0.18 (0.067), 0.0086 |
|  | Insomnia | 0.073 (0.035), 0.036 | 0.069 (0.038), 0.071 | 0.12 (0.050), 0.019 |  | 0.099 (0.057), 0.079 |  | 0.069 (0.093), 0.46 |  | 0.024 (0.055), 0.66 | -0.019 (0.073), 0.79 | 0.040 (0.067), 0.55 | 0.072 (0.068), 0.28 |  | 0.11 (0.081), 0.16 | 0.086 (0.071), 0.22 |  | -0.0472 (0.038), 0.21 | -0.12 (0.060), 0.04 |
| Psychiatric traits | Autism | -0.088 (0.041), 0.032 | -0.076 (0.045), 0.090 | -0.069 (0.066), 0.30 |  | -0.096 (0.065), 0.14 |  | -0.044 (0.12), 0.72 |  | -0.082 (0.069), 0.24 | -0.025 (0.083), 0.76 | -0.021 (0.088), 0.81 | 0.0073 (0.092), 0.94 |  | 0.0089 (0.093), 0.92 | -0.034 (0.092), 0.71 |  | -0.021 (0.048), 0.66 | -0.071 (0.073), 0.33 |
|  | Subject Well Being | -0.092 (0.057), 0.11 | -0.17 (0.062), 0.0066 | -0.10 (0.087), 0.24 |  | 0.040 (0.093), 0.67 |  | -0.17 (0.17), 0.33 |  | -0.13 (0.094), 0.16 | -0.18 (0.11), 0.11 | -0.22 (0.13), 0.081 | -0.15 (0.14), 0.29 |  | -0.16 (0.12), 0.21 | -0.019 (0.12), 0.88 |  | 0.10 (0.066), 0.12 | 0.19 (0.10), 0.065 |
|  | Anorexia | 0.048 (0.041), 0.24 | 0.035 (0.042), 0.40 | 0.029 (0.056), 0.60 |  | 0.0069 (0.063), 0.91 |  | -0.15 (0.11), 0.17 |  | -0.049 (0.062), 0.43 | 0.019 (0.073), 0.79 | 0.0067 (0.083), 0.94 | -0.17 (0.085), 0.042 |  | -0.030 (0.082), 0.72 | -0.0045 (0.078), 0.95 |  | -0.027 (0.041), 0.52 | -0.021 (0.061), 0.73 |
|  | Bipolar Disorder | 0.078 (0.041), 0.058 | 0.091 (0.044), 0.039 | -0.008 (0.055), 0.88 |  | -0.16 (0.072), 0.031 |  | -0.25 (0.12), 0.040 |  | -0.032 (0.068), 0.64 | 0.041 (0.081), 0.61 | -0.026 (0.082), 0.75 | -0.076 (0.086), 0.38 |  | -0.046 (0.098), 0.64 | -0.037 (0.08), 0.66 |  | 0.070 (0.042), 0.098 | 0.050 (0.066), 0.45 |
|  | Neuroticism | 0.092 (0.031), 0.0030 | 0.080 (0.034), 0.020 | 0.14 (0.047), 0.0029 |  | 0.051 (0.045), 0.25 |  | 0.060 (0.077), 0.43 |  | 0.14 (0.051), 0.0043 | 0.11 (0.060), 0.067 | 0.15 (0.057), 0.011 | 0.22 (0.069), 0.0017 |  | 0.079 (0.065), 0.22 | 0.034 (0.063), 0.58 |  | -0.020 (0.037), 0.58 | -0.058 (0.057), 0.31 |
|  | Depressive Symptoms | 0.11 (0.036), 0.0033 | 0.074 (0.040), 0.061 | **0.21 (0.049), 0.000015** |  | 0.025 (0.062), 0.69 |  | 0.048 (0.094), 0.61 |  | **0.25 (0.059), 0.000025** | 0.15 (0.071), 0.032 | 0.23 (0.076), 0.0020 | 0.30 (0.080), 0.00017 |  | 0.13 (0.081), 0.099 | 0.063 (0.076), 0.41 |  | -0.070 (0.041), 0.084 | -0.073 (0.059), 0.22 |
|  | Schizophrenia | **0.14 (0.025), 0.000000026** | **0.12 (0.027), 0.0000075** | 0.066 (0.034), 0.052 |  | -0.011 (0.036), 0.75 |  | 0.050 (0.067), 0.45 |  | 0.071 (0.038), 0.061 | 0.018 (0.049), 0.72 | -0.012 (0.053), 0.83 | 0.055 (0.058), 0.34 |  | 0.11 (0.059), 0.064 | 0.062 (0.06), 0.28 |  | 0.037 (0.028), 0.19 | 0.0093 (0.041), 0.82 |
| Results were shown for the six major cancers and its subsets.  In each checker, we presented the genetic correlation estimates, the standard errors in brackets, followed by P-values.  P < 0.05 / (13*38) were highlighted in bold. | | | | | | | | | | | | | | | | | | | |
