# Supplementary material for: Shared heritability and functional enrichment across six solid cancers
Source: Nat Commun. 2019 Jan 25;10:431. doi: 10.1038/s41467-018-08054-4 (PMC6347624; doi:10.1038/s41467-018-08054-4)
Supplement: Supplementary file 6 — Supplementary Data 4 [file 41467_2018_8054_MOESM6_ESM.docx]

**Shared heritability and functional enrichment across six solid cancers**

**Jiang et al.**

| **Supplementary Data 4. Enrichment estimates of the 220 cell-type-specific annotations over four histone marks H3K4me1, H3K4me3, H3K9ac, and H3K27ac, for six cancers.** | | | | | | |
| --- | --- | --- | --- | --- | --- | --- |
| **Cell type** | **Mark** | **Enrichment** | **Enrichment standard error** | **Enrichment P-values** | **Cancer** | **Category** |
| Colonic_mucosa | H3K27ac | 15.97 | 2.79 | **2.95E-07** | Colorectal | GI |
| Rectal_mucosa | H3K4me1 | 10.02 | 1.77 | **3.06E-07** | Prostate | GI |
| Stomach_mucosa | H3K4me1 | 10.19 | 1.81 | **3.14E-07** | Prostate | GI |
| Rectal_mucosa | H3K27ac | 14.99 | 2.80 | **3.24E-07** | Colorectal | GI |
| Rectal_mucosa | H3K4me1 | 12.22 | 2.23 | **6.59E-07** | Colorectal | GI |
| Colonic_mucosa | H3K4me1 | 14.34 | 2.71 | **8.04E-07** | Prostate | GI |
| Colonic_mucosa | H3K9ac | 19.93 | 3.92 | **8.30E-07** | Colorectal | GI |
| Penis_foreskin_fibroblast_primary | H3K4me1 | 6.53 | 1.10 | **9.52E-07** | Colorectal | Connective_Bone |
| Colonic_mucosa | H3K9ac | 14.88 | 2.97 | **2.24E-06** | Prostate | GI |
| Duodenum_Mucosa | H3K4me1 | 9.50 | 1.73 | **2.73E-06** | Colorectal | GI |
| Colonic_mucosa | H3K27ac | 10.39 | 2.02 | **3.74E-06** | Prostate | GI |
| Colonic_mucosa | H3K4me1 | 16.10 | 3.06 | **3.92E-06** | Colorectal | GI |
| Adipose_nuclei | H3K4me1 | 5.82 | 0.99 | **4.09E-06** | Breast | Other |
| Breast_myoepithelial | H3K4me1 | 5.70 | 1.07 | **4.28E-06** | Prostate | Other |
| Duodenum_Mucosa | H3K4me1 | 7.31 | 1.41 | **4.32E-06** | Prostate | GI |
| Fetal_small_intestine | H3K4me1 | 9.40 | 1.87 | **5.10E-06** | Prostate | GI |
| Rectal_mucosa | H3K27ac | 9.10 | 1.77 | **1.47E-05** | Prostate | GI |
| Rectal_mucosa | H3K4me3 | 18.08 | 4.29 | **1.77E-05** | Colorectal | GI |
| Fetal_large_intestine | H3K4me1 | 9.06 | 1.93 | **2.40E-05** | Prostate | GI |
| Gastric | H3K4me1 | 16.92 | 3.79 | **3.59E-05** | Prostate | GI |
| Duodenum_mucosa | H3K27ac | 10.98 | 2.45 | **4.59E-05** | Colorectal | GI |
| Colon_smooth_muscle | H3K4me1 | 8.13 | 1.82 | **4.91E-05** | Colorectal | GI |
| Liver_(BI) | H3K4me1 | 4.92 | 0.90 | **4.96E-05** | Prostate | Liver |
| Rectal_mucosa | H3K9ac | 19.35 | 4.80 | **5.27E-05** | Colorectal | GI |
| Fetal_large_intestine | H3K4me1 | 10.04 | 2.07 | **5.38E-05** | Colorectal | GI |
| Penis_foreskin_fibroblast_primary | H3K4me3 | 6.86 | 1.44 | **5.67E-05** | Colorectal | Connective_Bone |
| Kidney | H3K4me3 | 9.62 | 2.07 | **6.05E-05** | Breast | Kidney |
| Penis_foreskin_keratinocyte_primary | H3K4me1 | 6.27 | 1.29 | **6.59E-05** | Prostate | Other |
| Duodenum_Mucosa | H3K4me3 | 8.69 | 1.90 | **6.72E-05** | Breast | GI |
| Pancreas | H3K4me1 | 8.64 | 1.90 | **6.98E-05** | Prostate | Adrenal_Pancreas |
| Breast_myoepithelial | H3K4me1 | 6.74 | 1.42 | **7.90E-05** | Breast | Other |
| Adipose_nuclei | H3K9ac | 9.80 | 2.20 | **8.34E-05** | Breast | Other |
| Stomach_mucosa | H3K9ac | 16.01 | 3.93 | **9.18E-05** | Prostate | GI |
| Rectal_mucosa | H3K4me3 | 13.84 | 3.18 | **9.32E-05** | Prostate | GI |
| Fetal_large_intestine | H3K4me3 | 21.59 | 5.62 | **0.000103355** | Colorectal | GI |
| Fetal_lung | H3K4me1 | 6.55 | 1.31 | **0.000111117** | Colorectal | Cardiovascular |
| Duodenum_Mucosa | H3K9ac | 16.73 | 4.35 | **0.000129429** | Colorectal | GI |
| Penis_foreskin_fibroblast_primary | H3K4me1 | 4.46 | 0.92 | **0.000138391** | Breast | Connective_Bone |
| Fetal_small_intestine | H3K4me1 | 8.92 | 1.95 | **0.000148212** | Colorectal | GI |
| Colonic_mucosa | H3K4me3 | 20.83 | 5.56 | **0.000161369** | Colorectal | GI |
| Fetal_small_intestine | H3K4me1 | 7.54 | 1.71 | **0.000177927** | Breast | GI |
| Penis_foreskin_fibroblast_primary | H3K4me1 | 3.85 | 0.75 | **0.000178334** | Prostate | Connective_Bone |
| Breast_vHMEC | H3K4me1 | 7.41 | 1.66 | **0.000183845** | Prostate | Other |
| Liver_(BI) | H3K4me1 | 5.25 | 1.18 | **0.000195392** | Breast | Liver |
| Colon_smooth_muscle | H3K4me3 | 11.68 | 2.75 | 0.000235982 | Breast | GI |
| Stomach_mucosa | H3K4me1 | 9.52 | 2.18 | 0.000240876 | Colorectal | GI |
| Fetal_lung | H3K4me1 | 4.77 | 1.04 | 0.000256853 | Prostate | Cardiovascular |
| Penis_foreskin_keratinocyte_primary | H3K4me1 | 6.61 | 1.44 | 0.000272453 | Colorectal | Other |
| Stomach_mucosa | H3K4me3 | 25.47 | 6.80 | 0.000275556 | Prostate | GI |
| Fetal_large_intestine | H3K4me1 | 7.46 | 1.74 | 0.00029231 | Breast | GI |
| Stomach_mucosa | H3K4me1 | 9.68 | 2.40 | 0.000296253 | Breast | GI |
| Rectal_mucosa | H3K9ac | 11.83 | 3.06 | 0.000299544 | Prostate | GI |
| Rectal_mucosa | H3K9ac | 11.18 | 2.75 | 0.000301061 | Breast | GI |
| Fetal_small_intestine | H3K4me3 | 19.92 | 5.58 | 0.000318316 | Colorectal | GI |
| Penis_foreskin_keratinocyte_primary | H3K4me3 | 11.36 | 2.81 | 0.000337398 | Prostate | Other |
| Fetal_lung | H3K4me1 | 5.04 | 1.07 | 0.000347864 | Breast | Cardiovascular |
| Duodenum_Mucosa | H3K4me3 | 12.81 | 3.50 | 0.000352844 | Colorectal | GI |
| Penis_foreskin_keratinocyte_primary | H3K4me1 | 5.69 | 1.32 | 0.00037148 | Breast | Other |
| Rectal_smooth_muscle | H3K4me3 | 12.59 | 3.09 | 0.000387061 | Breast | GI |
| Duodenum_smooth_muscle | H3K27ac | 9.15 | 2.41 | 0.000449173 | Colorectal | GI |
| Small_intestine | H3K4me3 | 24.99 | 7.15 | 0.000515453 | Prostate | GI |
| Adipose_nuclei | H3K4me1 | 4.51 | 1.01 | 0.000558547 | Colorectal | Other |
| Colonic_mucosa | H3K9ac | 11.58 | 3.04 | 0.000563006 | Breast | GI |
| Rectal_smooth_muscle | H3K4me3 | 17.66 | 5.10 | 0.000589097 | Colorectal | GI |
| Colonic_mucosa | H3K4me3 | 15.78 | 4.22 | 0.000589581 | Prostate | GI |
| Fetal_lung | H3K9ac | 19.06 | 5.29 | 0.000592613 | Colorectal | Cardiovascular |
| Skeletal_muscle | H3K4me1 | 5.04 | 1.14 | 0.000633768 | Breast | SkeletalMuscle |
| Breast_vHMEC | H3K4me1 | 6.59 | 1.70 | 0.000669659 | Breast | Other |
| Stomach_smooth_muscle | H3K4me3 | 9.55 | 2.39 | 0.000776292 | Breast | GI |
| Stomach_mucosa | H3K9ac | 13.05 | 3.54 | 0.00081436 | Breast | GI |
| Penis_foreskin_melanocyte_primary | H3K4me1 | 4.19 | 0.92 | 0.000837101 | Breast | Other |
| Stomach_smooth_muscle | H3K4me3 | 11.79 | 3.32 | 0.000845273 | Colorectal | GI |
| Breast_myoepithelial | H3K4me3 | 11.66 | 3.20 | 0.000863788 | Prostate | Other |
| Duodenum_Mucosa | H3K4me1 | 6.48 | 1.66 | 0.000947452 | Breast | GI |
| Adipose_nuclei | H3K4me3 | 6.63 | 1.65 | 0.000952787 | Breast | Other |
| Liver_(BI) | H3K4me1 | 4.90 | 1.15 | 0.000971965 | Colorectal | Liver |
| Stomach_smooth_muscle | H3K9ac | 11.94 | 3.34 | 0.000983869 | Colorectal | GI |
| Penis_foreskin_fibroblast_primary | H3K4me3 | 5.47 | 1.32 | 0.001001645 | Breast | Connective_Bone |
| Breast_luminal_epithelial | H3K4me1 | 50.66 | 14.83 | 0.001023686 | Prostate | Other |
| Duodenum_Mucosa | H3K9ac | 13.16 | 3.76 | 0.00106613 | Prostate | GI |
| Rectal_smooth_muscle | H3K4me3 | 13.85 | 3.84 | 0.00109829 | Prostate | GI |
| Breast_vHMEC | H3K4me1 | 8.01 | 2.08 | 0.001118662 | Colorectal | Other |
| Fetal_large_intestine | H3K4me3 | 12.07 | 3.18 | 0.00116871 | Breast | GI |
| Rectal_mucosa | H3K4me3 | 10.89 | 2.98 | 0.001174784 | Breast | GI |
| Liver_(BI) | H3K4me3 | 7.41 | 1.98 | 0.001353442 | Breast | Liver |
| Fetal_small_intestine | H3K4me3 | 14.95 | 4.49 | 0.001381183 | Prostate | GI |
| Esophagus | H3K4me3 | 16.15 | 4.50 | 0.001398427 | Breast | GI |
| Liver_(BI) | H3K9ac | 8.78 | 2.39 | 0.001449605 | Breast | Liver |
| Fetal_lung | H3K9ac | 12.50 | 3.39 | 0.001468477 | Breast | Cardiovascular |
| Placenta_amnion | H3K4me3 | 19.23 | 5.26 | 0.001507238 | Breast | Other |
| Stomach_smooth_muscle | H3K4me1 | 8.71 | 2.45 | 0.001528398 | Colorectal | GI |
| Liver_(UCSD) | H3K4me3 | 9.78 | 2.65 | 0.001562378 | Breast | Liver |
| Duodenum_smooth_muscle | H3K4me3 | 7.19 | 1.94 | 0.001562586 | Breast | GI |
| Penis_foreskin_keratinocyte_primary | H3K9ac | 5.77 | 1.50 | 0.001618161 | Prostate | Other |
| Rectal_smooth_muscle | H3K4me1 | 16.25 | 4.81 | 0.001618402 | Colorectal | GI |
| Duodenum_Mucosa | H3K9ac | 8.75 | 2.42 | 0.001675587 | Breast | GI |
| Colon_smooth_muscle | H3K4me3 | 13.61 | 4.09 | 0.001676829 | Colorectal | GI |
| Liver_(UCSD) | H3K4me3 | 12.95 | 3.65 | 0.001683409 | Prostate | Liver |
| Fetal_stomach | H3K4me3 | 14.59 | 4.48 | 0.001718542 | Prostate | GI |
| Breast_myoepithelial | H3K4me1 | 5.60 | 1.40 | 0.001739069 | Colorectal | Other |
| Stomach_mucosa | H3K9ac | 17.35 | 5.55 | 0.00174492 | Colorectal | GI |
| Colonic_mucosa | H3K4me3 | 13.20 | 3.78 | 0.00175212 | Breast | GI |
| Gastric | H3K4me3 | 22.50 | 7.02 | 0.001760994 | Prostate | GI |
| CD15_primary | H3K4me1 | 4.88 | 1.27 | 0.001815288 | Prostate | Hematopoietic |
| Fetal_stomach | H3K4me1 | 7.95 | 2.26 | 0.001896719 | Colorectal | GI |
| Penis_foreskin_melanocyte_primary | H3K4me1 | 3.82 | 0.93 | 0.00193834 | Prostate | Other |
| Fetal_small_intestine | H3K4me3 | 11.40 | 3.17 | 0.002048536 | Breast | GI |
| Fetal_stomach | H3K4me3 | 17.17 | 5.42 | 0.002068821 | Colorectal | GI |
| Colon_smooth_muscle | H3K4me1 | 6.39 | 1.72 | 0.002098882 | Breast | GI |
| Kidney | H3K27ac | 6.52 | 1.78 | 0.002194591 | Breast | Kidney |
| Stomach_smooth_muscle | H3K9ac | 9.21 | 2.56 | 0.002196442 | Breast | GI |
| Duodenum_Mucosa | H3K4me3 | 9.50 | 2.80 | 0.002243393 | Prostate | GI |
| Adipose_nuclei | H3K9ac | 8.54 | 2.56 | 0.002260688 | Colorectal | Other |
| Esophagus | H3K4me3 | 18.13 | 5.47 | 0.002281984 | Prostate | GI |
| Fetal_placenta | H3K4me1 | 9.32 | 2.66 | 0.002300462 | Breast | Other |
| Fetal_large_intestine | H3K4me3 | 13.39 | 4.21 | 0.002415107 | Prostate | GI |
| Breast_vHMEC | H3K4me3 | 16.02 | 5.06 | 0.002508418 | Prostate | Other |
| Osteoblast | H3K27ac | 6.19 | 1.78 | 0.002534841 | Colorectal | Connective_Bone |
| Stomach_smooth_muscle | H3K4me1 | 8.17 | 2.30 | 0.002579832 | Breast | GI |
| Pancreas | H3K4me3 | 15.75 | 4.99 | 0.002595148 | Prostate | Adrenal_Pancreas |
| Rectal_mucosa | H3K4me1 | 7.50 | 2.20 | 0.002630075 | Breast | GI |
| Penis_foreskin_keratinocyte_primary | H3K4me3 | 8.87 | 2.50 | 0.002660808 | Breast | Other |
| CD4+_CD25-_Th_primary | H3K4me1 | 3.92 | 1.00 | 0.002702202 | Prostate | Hematopoietic |
| Skeletal_muscle | H3K4me1 | 3.92 | 0.97 | 0.0027985 | Colorectal | SkeletalMuscle |
| CD4+_CD25-_IL17-_PMA_Ionomycin_stim_MACS_Th_sprimary | H3K4me1 | 3.60 | 0.91 | 0.002798551 | Colorectal | Hematopoietic |
| Colonic_mucosa | H3K4me1 | 10.03 | 3.04 | 0.002827907 | Breast | GI |
| CD4+_CD25-_IL17-_PMA_Ionomycin_stim_MACS_Th_sprimary | H3K4me1 | 3.34 | 0.81 | 0.003105356 | Prostate | Hematopoietic |
| Fetal_adrenal | H3K4me1 | 6.08 | 1.67 | 0.003180072 | Breast | Adrenal_Pancreas |
| Psoas_muscle | H3K4me3 | 14.34 | 4.41 | 0.00356144 | Prostate | SkeletalMuscle |
| Stomach_smooth_muscle | H3K27ac | 6.47 | 1.80 | 0.003580973 | Colorectal | GI |
| Duodenum_smooth_muscle | H3K4me3 | 7.88 | 2.41 | 0.00367294 | Colorectal | GI |
| Pancreatic_islets | H3K4me3 | 32.92 | 13.61 | 0.003698806 | Headneck | Adrenal_Pancreas |
| Fetal_lung | H3K9ac | 12.17 | 3.87 | 0.003700543 | Prostate | Cardiovascular |
| CD8_naive_primary_(UCSF-UBC) | H3K4me1 | 7.33 | 2.02 | 0.003717808 | Lung | Hematopoietic |
| Duodenum_mucosa | H3K27ac | 6.92 | 1.97 | 0.003754001 | Prostate | GI |
| Breast_myoepithelial | H3K4me3 | 9.24 | 2.73 | 0.003766318 | Breast | Other |
| Spleen | H3K4me1 | 5.94 | 1.61 | 0.003866514 | Breast | Hematopoietic |
| Adipose_nuclei | H3K4me1 | 3.34 | 0.81 | 0.003881706 | Prostate | Other |
| Small_intestine | H3K4me3 | 19.49 | 6.05 | 0.004139429 | Breast | GI |
| CD4+_CD25-_Th_primary | H3K4me1 | 3.92 | 1.08 | 0.004201895 | Colorectal | Hematopoietic |
| Penis_foreskin_keratinocyte_primary | H3K4me3 | 11.89 | 3.71 | 0.004207201 | Colorectal | Other |
| Right_ventricle | H3K4me3 | 9.85 | 2.95 | 0.004317815 | Breast | Cardiovascular |
| Placenta_chorion | H3K4me3 | 13.26 | 4.24 | 0.004426885 | Prostate | Other |
| Adipose_nuclei | H3K4me3 | 5.89 | 1.74 | 0.004572362 | Colorectal | Other |
| Fetal_kidney | H3K9ac | 21.39 | 7.38 | 0.004586594 | Colorectal | Kidney |
| Liver_(BI) | H3K9ac | 10.15 | 3.33 | 0.004637757 | Colorectal | Liver |
| Penis_foreskin_melanocyte_primary | H3K4me3 | 6.71 | 1.94 | 0.004843106 | Breast | Other |
| Kidney | H3K9ac | 15.89 | 5.31 | 0.005074705 | Colorectal | Kidney |
| Kidney | H3K9ac | 11.07 | 3.68 | 0.005284121 | Prostate | Kidney |
| Fetal_placenta | H3K4me3 | 13.74 | 4.53 | 0.005775277 | Prostate | Other |
| Fetal_stomach | H3K4me1 | 8.03 | 2.40 | 0.005824285 | Breast | GI |
| Fetal_lung | H3K4me3 | 13.10 | 4.36 | 0.005833432 | Colorectal | Cardiovascular |
| Pancreas | H3K4me1 | 7.05 | 2.18 | 0.005892265 | Breast | Adrenal_Pancreas |
| Adipose_nuclei | H3K27ac | 6.51 | 2.02 | 0.006098068 | Breast | Other |
| Kidney | H3K4me3 | 8.86 | 2.81 | 0.006122852 | Prostate | Kidney |
| Liver_(BI) | H3K4me3 | 7.23 | 2.32 | 0.006272928 | Colorectal | Liver |
| CD4+_CD25-_CD45RA+_naive_primary | H3K4me1 | 4.08 | 1.15 | 0.006413227 | Prostate | Hematopoietic |
| Colonic_mucosa | H3K27ac | 6.64 | 2.01 | 0.006609099 | Breast | GI |
| Mobilized_CD34_primary | H3K4me1 | 3.42 | 0.91 | 0.006955548 | Prostate | Hematopoietic |
| Fetal_placenta | H3K4me3 | 15.34 | 5.45 | 0.007040583 | Colorectal | Other |
| Breast_fibroblast_primary | H3K4me1 | 12.81 | 4.61 | 0.007110265 | Colorectal | Connective_Bone |
| Fetal_leg_muscle | H3K4me1 | 6.73 | 2.01 | 0.00740226 | Breast | SkeletalMuscle |
| Sigmoid_colon | H3K4me3 | 18.05 | 6.45 | 0.007411367 | Prostate | GI |
| CD56_primary | H3K4me3 | 22.01 | 7.14 | 0.007487663 | Lung | Hematopoietic |
| Fetal_leg_muscle | H3K4me3 | 11.54 | 3.76 | 0.007489969 | Prostate | SkeletalMuscle |
| Sigmoid_colon | H3K4me3 | 16.27 | 5.43 | 0.007521911 | Breast | GI |
| CD4+_CD25+_CD127-_Treg_primary | H3K4me1 | 4.39 | 1.29 | 0.007815777 | Prostate | Hematopoietic |
| CD15_primary | H3K4me1 | 4.97 | 1.40 | 0.00786642 | Lung | Hematopoietic |
| Kidney | H3K4me3 | 9.80 | 3.35 | 0.007965882 | Colorectal | Kidney |
| Colon_smooth_muscle | H3K27ac | 6.33 | 2.02 | 0.008122449 | Colorectal | GI |
| Kidney | H3K9ac | 16.48 | 5.87 | 0.008372602 | Breast | Kidney |
| CD19_primary_(BI) | H3K4me1 | 4.97 | 1.43 | 0.00845286 | Breast | Hematopoietic |
| CD56_primary | H3K4me1 | 5.87 | 1.63 | 0.008634558 | Lung | Hematopoietic |
| Sigmoid_colon | H3K4me3 | 20.24 | 7.60 | 0.008745086 | Colorectal | GI |
| Fetal_stomach | H3K4me3 | 11.68 | 3.91 | 0.009457567 | Breast | GI |
| CD14_primary | H3K4me1 | 4.50 | 1.31 | 0.009718409 | Breast | Hematopoietic |
| CD15_primary | H3K4me3 | 11.87 | 3.88 | 0.009757519 | Lung | Hematopoietic |
| CD4+_CD25-_Th_primary | H3K4me1 | 5.42 | 1.55 | 0.010269627 | Lung | Hematopoietic |
| Rectal_smooth_muscle | H3K4me1 | 11.42 | 3.96 | 0.010289998 | Breast | GI |
| CD4+_CD25-_CD45RA+_naive_primary | H3K4me1 | 3.59 | 1.06 | 0.010422718 | Colorectal | Hematopoietic |
| CD3_primary | H3K27ac | 6.20 | 2.05 | 0.010432887 | Lung | Hematopoietic |
| Placenta_amnion | H3K4me3 | 14.45 | 5.09 | 0.010451169 | Prostate | Other |
| Lung | H3K4me3 | 21.93 | 7.91 | 0.010513653 | Prostate | Cardiovascular |
| Psoas_muscle | H3K4me3 | 9.86 | 3.30 | 0.010514494 | Breast | SkeletalMuscle |
| Breast_myoepithelial | H3K4me3 | 10.82 | 3.84 | 0.010613282 | Colorectal | Other |
| Duodenum_smooth_muscle | H3K4me1 | 18.85 | 7.04 | 0.010635604 | Colorectal | GI |
| CD19_primary_(UW) | H3K4me1 | 4.70 | 1.38 | 0.010950418 | Breast | Hematopoietic |
| CD4+_CD25-_IL17+_PMA_Ionomycin_stim_Th17_primary | H3K4me1 | 3.83 | 1.16 | 0.010963826 | Colorectal | Hematopoietic |
| Rectal_mucosa | H3K27ac | 5.62 | 1.77 | 0.010975933 | Breast | GI |
| Rectal_smooth_muscle | H3K27ac | 5.68 | 1.79 | 0.011031449 | Colorectal | GI |
| Rectal_mucosa | H3K4me3 | 9.42 | 3.41 | 0.011305911 | Lung | GI |
| CD4+_CD25-_IL17-_PMA_Ionomycin_stim_MACS_Th_sprimary | H3K4me1 | 4.53 | 1.28 | 0.011690079 | Lung | Hematopoietic |
| Penis_foreskin_fibroblast_primary | H3K4me3 | 4.54 | 1.45 | 0.011918393 | Prostate | Connective_Bone |
| Fetal_adrenal | H3K4me3 | 9.89 | 3.35 | 0.012063075 | Breast | Adrenal_Pancreas |
| CD4+_CD25-_CD45R0+_memory_primary | H3K4me1 | 5.82 | 1.85 | 0.012103721 | Lung | Hematopoietic |
| Osteoblast | H3K27ac | 3.43 | 1.01 | 0.012127415 | Breast | Connective_Bone |
| CD4_memory_primary | H3K4me1 | 4.86 | 1.48 | 0.012203262 | Lung | Hematopoietic |
| Adipose_nuclei | H3K9ac | 5.76 | 1.89 | 0.012234313 | Prostate | Other |
| Gastric | H3K4me3 | 13.67 | 4.83 | 0.01266299 | Breast | GI |
| Skeletal_muscle | H3K4me3 | 8.62 | 3.00 | 0.012720237 | Breast | SkeletalMuscle |
| CD34_primary | H3K4me3 | 9.16 | 3.13 | 0.012870791 | Breast | Hematopoietic |
| Mobilized_CD34_primary | H3K4me1 | 3.95 | 1.19 | 0.012924732 | Breast | Hematopoietic |
| Treg_primary | H3K4me3 | 9.32 | 3.10 | 0.012999845 | Lung | Hematopoietic |
| CD34_primary | H3K4me3 | 11.48 | 4.27 | 0.013341172 | Prostate | Hematopoietic |
| Colon_smooth_muscle | H3K9ac | 18.35 | 7.02 | 0.013958945 | Colorectal | GI |
| CD34_primary | H3K4me3 | 17.14 | 5.93 | 0.013982102 | Lung | Hematopoietic |
| CD4_memory_primary | H3K4me1 | 3.45 | 1.03 | 0.014050792 | Colorectal | Hematopoietic |
| CD3_primary_(BI) | H3K4me1 | 4.25 | 1.36 | 0.014058551 | Prostate | Hematopoietic |
| Pancreatic_islets | H3K27ac | 5.05 | 1.63 | 0.014100916 | Prostate | Adrenal_Pancreas |
| Fetal_heart | H3K9ac | 7.55 | 2.65 | 0.01419638 | Breast | Cardiovascular |
| Skeletal_muscle | H3K9ac | 8.47 | 3.00 | 0.014200051 | Breast | SkeletalMuscle |
| Fetal_placenta | H3K4me3 | 10.84 | 3.80 | 0.014344064 | Breast | Other |
| Lung | H3K4me3 | 20.34 | 8.05 | 0.014750639 | Colorectal | Cardiovascular |
| CD19_primary_(UW) | H3K4me1 | 3.76 | 1.13 | 0.015096195 | Colorectal | Hematopoietic |
| CD4+_CD25-_CD45R0+_memory_primary | H3K4me1 | 3.51 | 1.05 | 0.015167934 | Prostate | Hematopoietic |
| Th1 | H3K27ac | 5.41 | 1.73 | 0.015201523 | Lung | Hematopoietic |
| CD3_primary_(UW) | H3K4me1 | 3.64 | 1.10 | 0.015251044 | Prostate | Hematopoietic |
| Fetal_lung | H3K4me3 | 10.44 | 3.89 | 0.015309225 | Prostate | Cardiovascular |
| Substantia_nigra | H3K27ac | 4.26 | 1.41 | 0.015418397 | Lung | CNS |
| Liver_(BI) | H3K9ac | 7.95 | 2.79 | 0.015597106 | Prostate | Liver |
| Fetal_brain | H3K9ac | 8.72 | 3.07 | 0.015692079 | Breast | CNS |
| CD4+_CD25-_IL17+_PMA_Ionomycin_stim_Th17_primary | H3K4me1 | 3.19 | 0.93 | 0.015809404 | Prostate | Hematopoietic |
| Fetal_trunk_muscle | H3K4me1 | 3.87 | 1.13 | 0.015953144 | Prostate | SkeletalMuscle |
| Penis_foreskin_melanocyte_primary | H3K4me1 | 3.70 | 1.11 | 0.01604224 | Colorectal | Other |
| Kidney | H3K27ac | 4.51 | 1.44 | 0.01614767 | Prostate | Kidney |
| Kidney | H3K4me1 | 10.03 | 3.82 | 0.016308693 | Prostate | Kidney |
| Fetal_adrenal | H3K4me3 | 11.04 | 4.10 | 0.016544133 | Prostate | Adrenal_Pancreas |
| Skeletal_muscle | H3K9ac | 6.19 | 2.13 | 0.016779204 | Colorectal | SkeletalMuscle |
| Th0 | H3K27ac | 5.73 | 1.91 | 0.017041325 | Lung | Hematopoietic |
| CD14 | H3K27ac | 4.44 | 1.32 | 0.017076055 | Lung | Hematopoietic |
| Rectal_smooth_muscle | H3K9ac | 27.49 | 11.46 | 0.017079997 | Colorectal | GI |
| Ovary | H3K4me3 | 7.46 | 2.67 | 0.017158804 | Breast | Other |
| Small_intestine | H3K4me3 | 18.91 | 7.82 | 0.017368041 | Colorectal | GI |
| Pancreas | H3K4me3 | 15.20 | 6.22 | 0.017442401 | Colorectal | Adrenal_Pancreas |
| Colon_smooth_muscle | H3K9ac | 9.94 | 3.72 | 0.017581196 | Breast | GI |
| Skeletal_muscle | H3K27ac | 4.84 | 1.58 | 0.017653126 | Breast | SkeletalMuscle |
| Liver_(UCSD) | H3K4me3 | 11.78 | 4.76 | 0.017953476 | Colorectal | Liver |
| Gastric | H3K4me3 | 18.54 | 7.57 | 0.018021444 | Colorectal | GI |
| Right_atrium | H3K4me3 | 9.66 | 3.72 | 0.018307008 | Prostate | Cardiovascular |
| Kidney | H3K27ac | 10.40 | 3.87 | 0.018677824 | Ovarian | Kidney |
| CD4_memory_primary | H3K4me1 | 8.04 | 2.50 | 0.018829692 | Ovarian | Hematopoietic |
| CD4+_CD25-_IL17-_PMA_Ionomycin_stim_MACS_Th_sprimary | H3K4me1 | 3.23 | 0.93 | 0.018858711 | Breast | Hematopoietic |
| Fetal_heart | H3K4me1 | 3.73 | 1.14 | 0.019015977 | Colorectal | Cardiovascular |
| Pancreas | H3K4me1 | 9.73 | 3.96 | 0.019216359 | Ovarian | Adrenal_Pancreas |
| CD8_memory_primary | H3K4me1 | 5.17 | 1.76 | 0.019474158 | Lung | Hematopoietic |
| CD4_memory_primary | H3K4me1 | 3.11 | 0.93 | 0.01948515 | Prostate | Hematopoietic |
| CD4+_CD25+_CD127-_Treg_primary | H3K4me3 | 14.31 | 5.13 | 0.019795533 | Lung | Hematopoietic |
| CD15_primary | H3K4me1 | 3.50 | 1.07 | 0.019864041 | Breast | Hematopoietic |
| CD8_naive_primary_(UCSF-UBC) | H3K4me1 | 4.00 | 1.31 | 0.020079772 | Prostate | Hematopoietic |
| Kidney | H3K27ac | 4.86 | 1.61 | 0.020219578 | Colorectal | Kidney |
| Duodenum_smooth_muscle | H3K4me1 | 21.92 | 9.10 | 0.020344106 | Breast | GI |
| Inferior_temporal_lobe | H3K9ac | 8.16 | 3.20 | 0.020944552 | Lung | CNS |
| CD4+_CD25-_IL17-_PMA_Ionomycin_stim_MACS_Th_sprimary | H3K4me1 | 6.96 | 2.15 | 0.020997226 | Ovarian | Hematopoietic |
| Pancreatic_islets | H3K4me3 | 10.27 | 4.06 | 0.021166739 | Prostate | Adrenal_Pancreas |
| Placenta_amnion | H3K4me1 | 18.46 | 7.50 | 0.021402607 | Prostate | Other |
| CD4+_CD25+_CD127-_Treg_primary | H3K4me1 | 6.00 | 2.06 | 0.021416381 | Lung | Hematopoietic |
| CD19_primary_(BI) | H3K4me1 | 5.41 | 1.79 | 0.021508013 | Lung | Hematopoietic |
| Pancreatic_islets | H3K4me3 | 9.32 | 3.74 | 0.021671984 | Prostate | Adrenal_Pancreas |
| CD25+_CD127-_Treg | H3K27ac | 5.70 | 2.01 | 0.021742486 | Lung | Hematopoietic |
| Fetal_trunk_muscle | H3K4me3 | 11.08 | 4.25 | 0.02179576 | Prostate | SkeletalMuscle |
| Breast_vHMEC | H3K4me3 | 10.04 | 3.83 | 0.022578133 | Breast | Other |
| Fetal_adrenal | H3K4me3 | 12.23 | 5.06 | 0.022669943 | Colorectal | Adrenal_Pancreas |
| CD8_naive_primary_(BI) | H3K4me1 | 5.33 | 1.76 | 0.02269397 | Lung | Hematopoietic |
| Fetal_leg_muscle | H3K4me1 | 3.89 | 1.21 | 0.02295566 | Prostate | SkeletalMuscle |
| CD14_primary | H3K4me1 | 4.32 | 1.42 | 0.02359148 | Lung | Hematopoietic |
| CD4+_CD25-_CD45R0+_memory_primary | H3K4me1 | 3.68 | 1.24 | 0.023826944 | Colorectal | Hematopoietic |
| CD8_primary | H3K4me3 | 15.50 | 5.83 | 0.023959733 | Lung | Hematopoietic |
| CD19_primary_(BI) | H3K4me1 | 3.64 | 1.19 | 0.024027821 | Colorectal | Hematopoietic |
| Liver | H3K27ac | 4.24 | 1.39 | 0.024196754 | Prostate | Liver |
| Duodenum_mucosa | H3K27ac | 5.07 | 1.79 | 0.024370008 | Breast | GI |
| Skeletal_muscle | H3K27ac | 6.95 | 2.67 | 0.024434647 | Ovarian | SkeletalMuscle |
| Fetal_large_intestine | H3K4me3 | 12.82 | 4.96 | 0.024554311 | Lung | GI |
| Hippocampus_middle | H3K4me3 | 9.37 | 3.65 | 0.024676803 | Lung | CNS |
| Substantia_nigra | H3K4me1 | 3.61 | 1.19 | 0.024939152 | Colorectal | CNS |
| CD8_naive_primary_(BI) | H3K4me1 | 3.82 | 1.29 | 0.025295232 | Prostate | Hematopoietic |
| Right_ventricle | H3K4me3 | 15.18 | 6.14 | 0.025359416 | Lung | Cardiovascular |
| Penis_foreskin_keratinocyte_primary | H3K9ac | 6.13 | 2.25 | 0.025444673 | Colorectal | Other |
| CD4+_CD25-_IL17+_PMA_Ionomycin_stim_Th17_primary | H3K4me1 | 4.31 | 1.44 | 0.025484527 | Breast | Hematopoietic |
| Colon_smooth_muscle | H3K4me3 | 7.70 | 2.92 | 0.025578402 | Prostate | GI |
| CD3_primary_(UW) | H3K4me1 | 5.19 | 1.69 | 0.025662097 | Lung | Hematopoietic |
| Duodenum_Mucosa | H3K4me3 | 8.03 | 3.11 | 0.025802103 | Lung | GI |
| Germinal_matrix | H3K4me3 | 10.86 | 4.62 | 0.025874208 | Colorectal | CNS |
| Peripheralblood_mononuclear_primary | H3K4me3 | 18.02 | 6.99 | 0.026051172 | Lung | Hematopoietic |
| Liver | H3K27ac | 3.80 | 1.27 | 0.026347695 | Breast | Liver |
| Esophagus | H3K4me3 | 14.19 | 6.05 | 0.026355334 | Colorectal | GI |
| CD4+_CD25-_CD45RA+_naive_primary | H3K4me3 | 8.59 | 3.45 | 0.026435247 | Prostate | Hematopoietic |
| CD4_naive_primary | H3K4me1 | 3.64 | 1.22 | 0.026777117 | Prostate | Hematopoietic |
| Hippocampus_middle | H3K9ac | 7.47 | 2.98 | 0.027376227 | Lung | CNS |
| Fetal_placenta | H3K4me1 | 4.98 | 1.80 | 0.027573135 | Prostate | Other |
| Cingulate_gyrus | H3K4me3 | 9.83 | 3.94 | 0.027630722 | Lung | CNS |
| Fetal_heart | H3K9ac | 6.82 | 2.57 | 0.028411352 | Colorectal | Cardiovascular |
| Chondrogenic_dif | H3K27ac | 5.04 | 1.84 | 0.028573931 | Breast | Connective_Bone |
| Hippocampus_middle | H3K4me1 | 3.39 | 1.10 | 0.028633241 | Colorectal | CNS |
| Right_atrium | H3K4me3 | 7.68 | 2.98 | 0.029423891 | Breast | Cardiovascular |
| Fetal_heart | H3K4me1 | 3.93 | 1.34 | 0.029452562 | Breast | Cardiovascular |
| Pancreatic_islets | H3K9ac | 14.69 | 6.08 | 0.029640811 | Colorectal | Adrenal_Pancreas |
| Fetal_trunk_muscle | H3K4me3 | 16.56 | 6.99 | 0.030065389 | Breast | SkeletalMuscle |
| Penis_foreskin_melanocyte_primary | H3K4me3 | 6.83 | 2.76 | 0.030540084 | Prostate | Other |
| Fetal_leg_muscle | H3K4me3 | 16.21 | 6.85 | 0.03058296 | Breast | SkeletalMuscle |
| CD8_naive_primary_(UCSF-UBC) | H3K9ac | 47.01 | 19.61 | 0.03058711 | Lung | Hematopoietic |
| CD4+_CD25-_Th_primary | H3K4me1 | 3.68 | 1.21 | 0.030628186 | Breast | Hematopoietic |
| Colonic_mucosa | H3K4me3 | 11.73 | 5.05 | 0.030846067 | Lung | GI |
| Fetal_thymus | H3K4me3 | 8.41 | 3.33 | 0.031010354 | Breast | Hematopoietic |
| Fetal_trunk_muscle | H3K4me1 | 5.31 | 1.93 | 0.031208918 | Breast | SkeletalMuscle |
| Right_atrium | H3K4me1 | 15.09 | 6.44 | 0.031270823 | Breast | Cardiovascular |
| CD4+_CD25-_IL17+_PMA_Ionomycin_stim_Th17_primary | H3K4me1 | 5.11 | 1.79 | 0.031296613 | Lung | Hematopoietic |
| Aorta | H3K4me3 | 7.88 | 3.14 | 0.031306086 | Breast | Cardiovascular |
| CD15_primary | H3K4me3 | 6.87 | 2.68 | 0.032168672 | Breast | Hematopoietic |
| Adipose_nuclei | H3K27ac | 4.39 | 1.60 | 0.032305861 | Colorectal | Other |
| Placenta_chorion | H3K4me3 | 12.01 | 5.01 | 0.032772318 | Breast | Other |
| Pancreas | H3K4me1 | 5.77 | 2.11 | 0.03289601 | Colorectal | Adrenal_Pancreas |
| CD14_primary | H3K4me3 | 11.56 | 4.98 | 0.032952873 | Prostate | Hematopoietic |
| Mid_frontal_lobe | H3K9ac | 8.32 | 3.40 | 0.033005761 | Colorectal | CNS |
| Spleen | H3K4me3 | 20.05 | 8.51 | 0.033173794 | Breast | Hematopoietic |
| CD3_primary_(UW) | H3K4me1 | 3.69 | 1.32 | 0.033732103 | Colorectal | Hematopoietic |
| CD19_primary_(BI) | H3K4me1 | 3.35 | 1.15 | 0.034011269 | Prostate | Hematopoietic |
| Aorta | H3K4me3 | 12.08 | 5.05 | 0.034021275 | Prostate | Cardiovascular |
| Pancreas | H3K4me3 | 9.04 | 3.67 | 0.03408806 | Breast | Adrenal_Pancreas |
| Fetal_trunk_muscle | H3K4me1 | 4.29 | 1.50 | 0.034546137 | Colorectal | SkeletalMuscle |
| Fetal_small_intestine | H3K4me3 | 13.27 | 5.42 | 0.034846861 | Lung | GI |
| CD3_primary_(UW) | H3K4me3 | 14.84 | 6.12 | 0.035103904 | Lung | Hematopoietic |
| CD19_primary_(UW) | H3K4me1 | 2.98 | 0.99 | 0.035168103 | Prostate | Hematopoietic |
| CD8_memory_primary | H3K4me1 | 3.48 | 1.21 | 0.035659816 | Prostate | Hematopoietic |
| Cingulate_gyrus | H3K4me1 | 3.50 | 1.21 | 0.036094904 | Colorectal | CNS |
| CD19_primary_(BI) | H3K4me3 | 15.46 | 6.47 | 0.036119924 | Lung | Hematopoietic |
| Chondrogenic_dif | H3K27ac | 4.99 | 1.92 | 0.036148354 | Colorectal | Connective_Bone |
| CD4+_CD25-_Th_primary | H3K4me3 | 14.79 | 5.94 | 0.036244595 | Lung | Hematopoietic |
| CD4_naive_primary | H3K4me1 | 4.77 | 1.72 | 0.036340781 | Lung | Hematopoietic |
| Breast_myoepithelial | H3K4me3 | 9.46 | 4.13 | 0.036810213 | Lung | Other |
| Inferior_temporal_lobe | H3K27ac | 8.52 | 3.76 | 0.037213835 | Headneck | CNS |
| Gastric | H3K4me1 | 11.13 | 4.82 | 0.037278168 | Breast | GI |
| CD4+_CD25-_CD45R0+_memory_primary | H3K4me1 | 8.34 | 3.01 | 0.037382707 | Ovarian | Hematopoietic |
| Fetal_thymus | H3K4me3 | 9.98 | 4.40 | 0.037478733 | Prostate | Hematopoietic |
| Fetal_adrenal | H3K4me1 | 4.89 | 1.84 | 0.038962272 | Colorectal | Adrenal_Pancreas |
| CD14_primary | H3K4me1 | 3.36 | 1.17 | 0.03896234 | Prostate | Hematopoietic |
| Liver_(BI) | H3K4me3 | 5.34 | 2.07 | 0.038970875 | Prostate | Liver |
| CD4+_CD25+_CD127-_Treg_primary | H3K4me1 | 4.83 | 1.80 | 0.039186757 | Breast | Hematopoietic |
| Anterior_caudate | H3K9ac | 8.39 | 3.76 | 0.039239497 | Lung | CNS |
| Treg_primary | H3K4me3 | 4.93 | 1.92 | 0.039585713 | Prostate | Hematopoietic |
| Mobilized_CD34_primary | H3K4me3 | 8.55 | 3.62 | 0.039615556 | Breast | Hematopoietic |
| Inferior_temporal_lobe | H3K4me1 | 3.32 | 1.15 | 0.03978195 | Colorectal | CNS |
| Duodenum_mucosa | H3K27ac | 11.22 | 5.09 | 0.039848412 | Ovarian | GI |
| CD4+_CD25-_CD45R0+_memory_primary | H3K4me3 | 16.82 | 7.22 | 0.039978128 | Lung | Hematopoietic |
| CD8_naive_primary_(BI) | H3K4me1 | 7.59 | 2.82 | 0.040323468 | Ovarian | Hematopoietic |
| CD4+_CD25-_Th_primary | H3K4me1 | 7.32 | 2.59 | 0.040885207 | Ovarian | Hematopoietic |
| Rectal_mucosa | H3K9ac | 7.48 | 3.27 | 0.040912012 | Lung | GI |
| CD4+_CD25-_CD45RA+_naive_primary | H3K4me1 | 7.11 | 2.51 | 0.040954125 | Ovarian | Hematopoietic |
| Left_Ventricle | H3K4me1 | 5.93 | 2.39 | 0.041051699 | Breast | Cardiovascular |
| Anterior_caudate | H3K4me1 | 3.54 | 1.26 | 0.041589281 | Colorectal | CNS |
| CD56_primary | H3K4me1 | 4.06 | 1.56 | 0.041658391 | Colorectal | Hematopoietic |
| CD8_memory_primary | H3K4me1 | 7.86 | 2.90 | 0.041947717 | Ovarian | Hematopoietic |
| CD15_primary | H3K4me3 | 7.47 | 3.18 | 0.042055958 | Prostate | Hematopoietic |
| CD4+_CD25-_IL17+_PMA_Ionomycin_stim_Th17_primary | H3K4me3 | 13.86 | 5.94 | 0.042172859 | Lung | Hematopoietic |
| CD19_primary_(UW) | H3K4me3 | 11.58 | 5.36 | 0.04219145 | Colorectal | Hematopoietic |
| Pancreatic_islets | H3K4me3 | 7.73 | 3.21 | 0.042383811 | Breast | Adrenal_Pancreas |
| Fetal_lung | H3K4me3 | 8.39 | 3.53 | 0.042666774 | Breast | Cardiovascular |
| Anterior_caudate | H3K27ac | 4.69 | 1.98 | 0.043014193 | Lung | CNS |
| Mobilized_CD34_primary | H3K4me3 | 8.24 | 3.29 | 0.043167377 | Lung | Hematopoietic |
| Fetal_leg_muscle | H3K4me3 | 11.28 | 5.28 | 0.043190619 | Colorectal | SkeletalMuscle |
| Kidney | H3K4me1 | 14.27 | 6.61 | 0.043429665 | Breast | Kidney |
| CD4+_CD25-_IL17+_PMA_Ionomycin_stim_Th17_primary | H3K4me3 | 6.79 | 2.80 | 0.04349866 | Breast | Hematopoietic |
| CD14_primary | H3K4me3 | 8.87 | 3.72 | 0.043600435 | Breast | Hematopoietic |
| CD4_naive_primary | H3K4me1 | 7.64 | 2.85 | 0.043691712 | Ovarian | Hematopoietic |
| CD34_primary | H3K4me1 | 5.00 | 1.88 | 0.043742945 | Lung | Hematopoietic |
| CD4+_CD25-_IL17-_PMA_Ionomycin_stim_MACS_Th_sprimary | H3K4me3 | 9.66 | 3.92 | 0.044415996 | Lung | Hematopoietic |
| CD4+_CD25-_CD45RA+_naive_primary | H3K4me1 | 3.27 | 1.10 | 0.044552994 | Breast | Hematopoietic |
| Ovary | H3K4me3 | 28.44 | 14.99 | 0.044617154 | Ovarian | Other |
| Germinal_matrix | H3K4me3 | 12.30 | 5.58 | 0.044831458 | Lung | CNS |
| CD25-_IL17+_Th17_stim | H3K27ac | 6.90 | 2.71 | 0.044986689 | Lung | Hematopoietic |
| Fetal_heart | H3K9ac | 6.80 | 2.77 | 0.045263606 | Lung | Cardiovascular |
| CD3_primary_(BI) | H3K4me1 | 7.48 | 2.78 | 0.045579222 | Ovarian | Hematopoietic |
| CD4+_CD25-_IL17+_PMA_Ionomycin_stim_Th17_primary | H3K4me1 | 7.45 | 2.74 | 0.045646771 | Ovarian | Hematopoietic |
| Stomach_mucosa | H3K4me3 | 11.11 | 4.93 | 0.046016466 | Breast | GI |
| Peripheralblood_mononuclear_primary | H3K9ac | 13.63 | 5.94 | 0.046630231 | Lung | Hematopoietic |
| Sigmoid_colon | H3K4me1 | 17.38 | 8.32 | 0.046794296 | Prostate | GI |
| Stomach_mucosa | H3K4me3 | 17.63 | 8.65 | 0.047327805 | Colorectal | GI |
| CD25-_IL17-_Th_stim_MACS | H3K27ac | 5.52 | 2.23 | 0.047461514 | Lung | Hematopoietic |
| Ovary | H3K4me3 | 8.68 | 3.84 | 0.048257744 | Prostate | Other |
| CD25-_CD45RA+_naive | H3K27ac | 9.01 | 4.29 | 0.04851145 | Headneck | Hematopoietic |
| Mobilized_CD34_primary | H3K4me1 | 3.44 | 1.13 | 0.048528099 | Lung | Hematopoietic |
| Left_Ventricle | H3K4me3 | 10.98 | 5.14 | 0.049120262 | Colorectal | Cardiovascular |
| Colon_smooth_muscle | H3K27ac | 4.86 | 1.91 | 0.049130752 | Breast | GI |
| Fetal_thymus | H3K4me3 | 17.87 | 7.89 | 0.049430223 | Lung | Hematopoietic |
| Peripheralblood_mononuclear_primary | H3K9ac | 7.94 | 3.43 | 0.049581924 | Breast | Hematopoietic |
| Germinal_matrix | H3K4me3 | 7.24 | 3.11 | 0.04979377 | Breast | CNS |
| Mobilized_CD34_primary | H3K4me3 | 7.37 | 3.44 | 0.050374432 | Colorectal | Hematopoietic |
| CD4_naive_primary | H3K4me3 | 12.37 | 5.38 | 0.05185361 | Lung | Hematopoietic |
| CD4+_CD25-_CD45RA+_naive_primary | H3K4me1 | 4.16 | 1.51 | 0.05212217 | Lung | Hematopoietic |
| Inferior_temporal_lobe | H3K4me3 | 8.44 | 3.89 | 0.052528406 | Lung | CNS |
| CD3_primary_(BI) | H3K4me1 | 4.59 | 1.80 | 0.052637584 | Lung | Hematopoietic |
| CD8_memory_primary | H3K4me1 | 3.25 | 1.21 | 0.052814572 | Colorectal | Hematopoietic |
| Left_Ventricle | H3K4me3 | 9.69 | 4.57 | 0.053380857 | Prostate | Cardiovascular |
| CD8_naive_primary_(BI) | H3K4me3 | 13.43 | 6.00 | 0.054054927 | Lung | Hematopoietic |
| Fetal_thymus | H3K4me1 | 3.81 | 1.45 | 0.054088303 | Breast | Hematopoietic |
| Rectal_smooth_muscle | H3K27ac | 4.38 | 1.63 | 0.054524127 | Lung | GI |
| CD34_primary | H3K4me1 | 5.33 | 2.27 | 0.054673993 | Breast | Hematopoietic |
| Small_intestine | H3K4me3 | 23.23 | 11.18 | 0.054776155 | Lung | GI |
| CD3_primary_(BI) | H3K4me3 | 6.97 | 3.18 | 0.056479062 | Prostate | Hematopoietic |
| Placenta_chorion | H3K4me1 | 11.03 | 5.26 | 0.056494729 | Prostate | Other |
| Mobilized_CD34_primary | H3K4me1 | 3.00 | 1.08 | 0.056586815 | Colorectal | Hematopoietic |
| CD4_memory_primary | H3K4me1 | 3.25 | 1.16 | 0.057001189 | Breast | Hematopoietic |
| CD8_naive_primary_(BI) | H3K4me1 | 3.27 | 1.24 | 0.057214151 | Colorectal | Hematopoietic |
| Rectal_smooth_muscle | H3K4me3 | 10.45 | 4.84 | 0.057362624 | Lung | GI |
| Fetal_trunk_muscle | H3K4me3 | 10.94 | 5.40 | 0.057917339 | Colorectal | SkeletalMuscle |
| Fetal_placenta | H3K4me1 | 7.43 | 3.26 | 0.057922745 | Colorectal | Other |
| Mid_frontal_lobe | H3K4me3 | 8.37 | 4.01 | 0.058024047 | Lung | CNS |
| Lung | H3K4me1 | 8.09 | 3.72 | 0.059063619 | Breast | Cardiovascular |
| CD4+_CD25-_CD45R0+_memory_primary | H3K4me1 | 3.82 | 1.46 | 0.059287697 | Breast | Hematopoietic |
| Angular_gyrus | H3K27ac | 8.37 | 4.09 | 0.059718102 | Headneck | CNS |
| Left_Ventricle | H3K4me1 | 4.32 | 1.78 | 0.059808078 | Colorectal | Cardiovascular |
| CD25-_CD45RA+_naive | H3K27ac | 3.51 | 1.34 | 0.05981197 | Prostate | Hematopoietic |
| Kidney | H3K4me3 | 22.54 | 10.42 | 0.059893226 | Ovarian | Kidney |
| CD19_primary_(BI) | H3K4me3 | 7.76 | 3.46 | 0.060108908 | Breast | Hematopoietic |
| Treg_primary | H3K4me3 | 4.90 | 2.17 | 0.060246522 | Colorectal | Hematopoietic |
| CD3_primary_(UW) | H3K4me1 | 6.74 | 2.63 | 0.060329386 | Ovarian | Hematopoietic |
| CD4_primary | H3K4me3 | 12.45 | 5.64 | 0.060396216 | Lung | Hematopoietic |
| CD4+_CD25-_IL17-_PMA_Ionomycin_stim_MACS_Th_sprimary | H3K4me3 | 5.19 | 2.18 | 0.060444245 | Breast | Hematopoietic |
| Placenta_chorion | H3K4me3 | 11.66 | 5.71 | 0.061064157 | Colorectal | Other |
| Pancreatic_islets | H3K4me3 | 9.58 | 4.70 | 0.061076746 | Colorectal | Adrenal_Pancreas |
| Esophagus | H3K4me3 | 12.79 | 6.15 | 0.061391031 | Lung | GI |
| CD3_primary_(BI) | H3K4me1 | 4.21 | 1.66 | 0.061467543 | Breast | Hematopoietic |
| Psoas_muscle | H3K4me3 | 9.85 | 4.85 | 0.061475658 | Colorectal | SkeletalMuscle |
| Breast_fibroblast_primary | H3K4me1 | 9.84 | 4.94 | 0.061950974 | Breast | Connective_Bone |
| Fetal_kidney | H3K9ac | 11.74 | 5.61 | 0.062278062 | Breast | Kidney |
| Fetal_thymus | H3K4me1 | 2.95 | 1.08 | 0.062450291 | Prostate | Hematopoietic |
| CD14_primary | H3K4me3 | 13.68 | 6.47 | 0.062684875 | Lung | Hematopoietic |
| CD8_memory_primary | H3K4me3 | 11.56 | 5.39 | 0.062829429 | Lung | Hematopoietic |
| Neurosphere | H3K27ac | 5.35 | 2.48 | 0.063118585 | Ovarian | CNS |
| CD4+_CD25int_CD127+_Tmem_primary | H3K4me3 | 16.06 | 7.45 | 0.063295607 | Lung | Hematopoietic |
| Pancreatic_islets | H3K4me1 | 12.21 | 5.98 | 0.063891046 | Colorectal | Adrenal_Pancreas |
| Pancreatic_islets | H3K4me1 | 12.78 | 6.40 | 0.063934673 | Prostate | Adrenal_Pancreas |
| Liver_(BI) | H3K9ac | 15.20 | 7.15 | 0.064019702 | Ovarian | Liver |
| Hippocampus_middle | H3K27ac | 4.51 | 2.05 | 0.064578735 | Lung | CNS |
| Colon_smooth_muscle | H3K4me1 | 3.73 | 1.45 | 0.064695374 | Prostate | GI |
| CD34_primary | H3K4me3 | 11.27 | 5.81 | 0.065229748 | Colorectal | Hematopoietic |
| Substantia_nigra | H3K4me3 | 9.33 | 4.48 | 0.065291327 | Lung | CNS |
| Left_Ventricle | H3K4me3 | 15.43 | 7.51 | 0.065316953 | Lung | Cardiovascular |
| Colonic_mucosa | H3K9ac | 5.86 | 2.77 | 0.065640502 | Lung | GI |
| CD34_primary | H3K4me1 | 4.10 | 1.72 | 0.066057692 | Prostate | Hematopoietic |
| Cingulate_gyrus | H3K9ac | 6.03 | 2.75 | 0.06609744 | Colorectal | CNS |
| Duodenum_Mucosa | H3K4me3 | 14.10 | 6.13 | 0.066188728 | Ovarian | GI |
| Lung | H3K4me3 | 9.82 | 4.67 | 0.067093148 | Breast | Cardiovascular |
| Right_ventricle | H3K4me3 | 8.25 | 4.03 | 0.067186845 | Prostate | Cardiovascular |
| Cingulate_gyrus | H3K9ac | 6.00 | 2.78 | 0.067195804 | Lung | CNS |
| Right_atrium | H3K4me1 | 6.16 | 2.87 | 0.067476683 | Prostate | Cardiovascular |
| Skeletal_muscle | H3K4me3 | 5.54 | 2.49 | 0.067560971 | Colorectal | SkeletalMuscle |
| Fetal_leg_muscle | H3K4me3 | 12.28 | 6.10 | 0.06803836 | Lung | SkeletalMuscle |
| CD19_primary_(BI) | H3K4me3 | 8.44 | 4.11 | 0.068086993 | Prostate | Hematopoietic |
| CD4+_CD25-_Th_primary | H3K4me3 | 7.05 | 3.39 | 0.068529411 | Prostate | Hematopoietic |
| Stomach_mucosa | H3K4me3 | 18.51 | 9.22 | 0.068742033 | Lung | GI |
| Penis_foreskin_keratinocyte_primary | H3K9ac | 4.20 | 1.75 | 0.06883576 | Breast | Other |
| CD4+_CD25+_CD127-_Treg_primary | H3K4me1 | 3.66 | 1.53 | 0.06892177 | Colorectal | Hematopoietic |
| Mobilized_CD34_primary | H3K4me3 | 5.81 | 2.68 | 0.069488864 | Prostate | Hematopoietic |
| Duodenum_smooth_muscle | H3K27ac | 3.46 | 1.33 | 0.069524828 | Breast | GI |
| Fetal_small_intestine | H3K4me3 | 23.22 | 10.46 | 0.069908431 | Ovarian | GI |
| CD4+_CD25+_CD127-_Treg_primary | H3K4me3 | 6.15 | 2.75 | 0.070026798 | Breast | Hematopoietic |
| Substantia_nigra | H3K9ac | 6.61 | 3.17 | 0.070600903 | Lung | CNS |
| Pancreatic_islets | H3K27ac | 3.98 | 1.61 | 0.071098268 | Colorectal | Adrenal_Pancreas |
| CD4+_CD25+_CD127-_Treg_primary | H3K4me3 | 5.77 | 2.69 | 0.071360048 | Prostate | Hematopoietic |
| Esophagus | H3K4me1 | 10.09 | 5.03 | 0.071546521 | Prostate | GI |
| Fetal_leg_muscle | H3K4me1 | 3.75 | 1.50 | 0.07232375 | Colorectal | SkeletalMuscle |
| Pancreatic_islets | H3K4me1 | 8.23 | 4.05 | 0.072537078 | Colorectal | Adrenal_Pancreas |
| Left_Ventricle | H3K4me3 | 9.01 | 4.33 | 0.072804239 | Breast | Cardiovascular |
| Anterior_caudate | H3K4me3 | 7.40 | 3.59 | 0.072990372 | Lung | CNS |
| CD3_primary | H3K27ac | 6.31 | 2.88 | 0.073461387 | Ovarian | Hematopoietic |
| CD4_memory_primary | H3K4me3 | 11.67 | 5.65 | 0.073893766 | Lung | Hematopoietic |
| CD4+_CD25int_CD127+_Tmem_primary | H3K4me3 | 7.23 | 3.56 | 0.07389642 | Prostate | Hematopoietic |
| CD3_primary_(UW) | H3K4me1 | 3.55 | 1.40 | 0.074010235 | Breast | Hematopoietic |
| Colonic_mucosa | H3K27ac | 9.58 | 4.71 | 0.074262176 | Ovarian | GI |
| Skeletal_muscle | H3K4me1 | 2.52 | 0.84 | 0.074368912 | Prostate | SkeletalMuscle |
| Fetal_thymus | H3K4me1 | 3.81 | 1.49 | 0.075317445 | Lung | Hematopoietic |
| Fetal_adrenal | H3K4me3 | 15.15 | 7.59 | 0.076819283 | Lung | Adrenal_Pancreas |
| CD25-_IL17-_Th_stim_MACS | H3K27ac | 7.95 | 3.67 | 0.078621299 | Ovarian | Hematopoietic |
| Fetal_kidney | H3K9ac | 12.48 | 6.60 | 0.079069701 | Prostate | Kidney |
| Right_ventricle | H3K4me3 | 9.02 | 4.67 | 0.079163692 | Colorectal | Cardiovascular |
| Sigmoid_colon | H3K4me3 | 21.02 | 10.95 | 0.079323667 | Lung | GI |
| Duodenum_smooth_muscle | H3K4me3 | 4.67 | 2.12 | 0.079469786 | Prostate | GI |
| Substantia_nigra | H3K27ac | 6.86 | 3.39 | 0.079954223 | Headneck | CNS |
| Peripheralblood_mononuclear_primary | H3K4me1 | 10.57 | 5.29 | 0.080045571 | Breast | Hematopoietic |
| Fetal_brain | H3K4me3 | 5.73 | 2.65 | 0.080542133 | Breast | CNS |
| Esophagus | H3K4me1 | 18.97 | 10.23 | 0.081066535 | Breast | GI |
| Inferior_temporal_lobe | H3K27ac | 3.62 | 1.65 | 0.08159284 | Lung | CNS |
| Rectal_smooth_muscle | H3K9ac | 15.33 | 8.09 | 0.081703025 | Prostate | GI |
| CD4+_CD25int_CD127+_Tmem_primary | H3K4me1 | 4.16 | 1.83 | 0.081952037 | Prostate | Hematopoietic |
| CD4_naive_primary | H3K4me1 | 3.01 | 1.20 | 0.0826492 | Colorectal | Hematopoietic |
| Peripheralblood_mononuclear_primary | H3K4me3 | 8.06 | 4.13 | 0.083358881 | Prostate | Hematopoietic |
| Stomach_smooth_muscle | H3K27ac | 3.26 | 1.29 | 0.083497747 | Breast | GI |
| Breast_luminal_epithelial | H3K4me1 | 40.16 | 22.56 | 0.083878902 | Breast | Other |
| Hippocampus_middle | H3K9ac | 5.91 | 2.89 | 0.084620768 | Colorectal | CNS |
| Pancreatic_islets | H3K4me1 | 11.14 | 5.80 | 0.084708745 | Prostate | Adrenal_Pancreas |
| CD19_primary_(UW) | H3K4me3 | 8.17 | 4.00 | 0.084803577 | Breast | Hematopoietic |
| Gastric | H3K4me1 | 9.56 | 4.81 | 0.084820174 | Colorectal | GI |
| CD25-_CD45RA+_naive | H3K27ac | 5.31 | 2.63 | 0.086775261 | Ovarian | Hematopoietic |
| Right_atrium | H3K4me3 | 11.74 | 6.13 | 0.086919006 | Lung | Cardiovascular |
| CD4+_CD25int_CD127+_Tmem_primary | H3K4me1 | 6.01 | 2.86 | 0.08697805 | Breast | Hematopoietic |
| Fetal_large_intestine | H3K4me3 | 22.16 | 10.83 | 0.089287783 | Ovarian | GI |
| Fetal_stomach | H3K4me3 | 12.69 | 6.60 | 0.090186995 | Lung | GI |
| CD4+_CD25+_CD127-_Treg_primary | H3K4me3 | 6.67 | 3.49 | 0.09087828 | Colorectal | Hematopoietic |
| CD19_primary_(UW) | H3K4me3 | 15.70 | 8.28 | 0.091047829 | Lung | Hematopoietic |
| Colon_smooth_muscle | H3K4me3 | 18.84 | 9.36 | 0.091129519 | Ovarian | GI |
| CD4_naive_primary | H3K4me3 | 5.86 | 2.92 | 0.091838012 | Prostate | Hematopoietic |
| Cingulate_gyrus | H3K27ac | 6.92 | 3.57 | 0.091913491 | Headneck | CNS |
| Stomach_mucosa | H3K9ac | 7.52 | 3.87 | 0.092352247 | Lung | GI |
| CD25-_CD45RA+_naive | H3K27ac | 4.07 | 1.76 | 0.092527459 | Lung | Hematopoietic |
| Cingulate_gyrus | H3K27ac | 3.47 | 1.55 | 0.092798974 | Lung | CNS |
| Th2 | H3K27ac | 4.72 | 2.25 | 0.093154041 | Lung | Hematopoietic |
| Rectal_smooth_muscle | H3K27ac | 3.58 | 1.50 | 0.093693989 | Breast | GI |
| Liver_(UCSD) | H3K4me1 | 18.00 | 10.27 | 0.093757686 | Breast | Liver |
| Peripheralblood_mononuclear_primary | H3K4me3 | 7.22 | 3.59 | 0.094392535 | Breast | Hematopoietic |
| Fetal_brain | H3K4me3 | 7.83 | 4.22 | 0.09515283 | Colorectal | CNS |
| Rectal_smooth_muscle | H3K9ac | 22.79 | 12.63 | 0.095187235 | Lung | GI |
| Psoas_muscle | H3K4me1 | 10.02 | 5.45 | 0.095218654 | Prostate | SkeletalMuscle |
| CD4+_CD25-_CD45RA+_naive_primary | H3K4me3 | 11.20 | 5.70 | 0.095657515 | Lung | Hematopoietic |
| CD19_primary_(UW) | H3K4me1 | 4.07 | 1.71 | 0.09577332 | Lung | Hematopoietic |
| CD4_primary | H3K4me3 | 5.99 | 3.05 | 0.095986506 | Prostate | Hematopoietic |
| Ovary | H3K4me1 | 10.98 | 5.96 | 0.096037094 | Breast | Other |
| CD4+_CD25+_CD127-_Treg_primary | H3K4me1 | 8.58 | 3.96 | 0.097174007 | Ovarian | Hematopoietic |
| CD3_primary | H3K27ac | 6.99 | 3.70 | 0.097306833 | Headneck | Hematopoietic |
| Fetal_brain | H3K4me3 | 27.03 | 15.13 | 0.097351842 | Lung | CNS |
| Pancreatic_islets | H3K9ac | 12.40 | 7.08 | 0.097564268 | Prostate | Adrenal_Pancreas |
| Skeletal_muscle | H3K9ac | 3.83 | 1.70 | 0.097721295 | Prostate | SkeletalMuscle |
| Treg_primary | H3K4me3 | 5.58 | 2.70 | 0.098083102 | Breast | Hematopoietic |
| Stomach_smooth_muscle | H3K4me3 | 7.56 | 3.82 | 0.098525822 | Lung | GI |
| Breast_myoepithelial | H3K9ac | 9.78 | 5.18 | 0.09865439 | Breast | Other |
| Duodenum_Mucosa | H3K9ac | 7.00 | 3.73 | 0.099380479 | Lung | GI |
| Liver | H3K27ac | 5.19 | 2.70 | 0.099681013 | Ovarian | Liver |
| Penis_foreskin_fibroblast_primary | H3K4me3 | 3.58 | 1.55 | 0.099907707 | Lung | Connective_Bone |
| Fetal_brain | H3K4me3 | 8.77 | 4.73 | 0.099999368 | Lung | CNS |
| CD4+_CD25-_CD45R0+_memory_primary | H3K4me3 | 7.31 | 3.74 | 0.101067887 | Breast | Hematopoietic |
| Fetal_brain | H3K4me3 | 6.56 | 3.45 | 0.101120234 | Prostate | CNS |
| Angular_gyrus | H3K4me1 | 11.76 | 6.98 | 0.101336034 | Headneck | CNS |
| Anterior_caudate | H3K27ac | 6.47 | 3.52 | 0.101668771 | Ovarian | CNS |
| Angular_gyrus | H3K9ac | 8.19 | 4.44 | 0.102016867 | Lung | CNS |
| CD8_naive_primary_(UCSF-UBC) | H3K4me3 | 16.67 | 9.15 | 0.103063748 | Lung | Hematopoietic |
| Spleen | H3K4me1 | 3.30 | 1.41 | 0.104340991 | Colorectal | Hematopoietic |
| Small_intestine | H3K4me1 | 43.74 | 26.57 | 0.104461885 | Colorectal | GI |
| Spleen | H3K4me1 | 2.77 | 1.12 | 0.105943336 | Prostate | Hematopoietic |
| CD14_primary | H3K4me3 | 10.70 | 6.25 | 0.107003642 | Colorectal | Hematopoietic |
| Penis_foreskin_melanocyte_primary | H3K4me1 | 4.15 | 1.98 | 0.108264451 | Ovarian | Other |
| Rectal_mucosa | H3K27ac | 8.06 | 4.32 | 0.108338223 | Ovarian | GI |
| Fetal_brain | H3K9ac | 7.74 | 4.18 | 0.108798857 | Colorectal | CNS |
| CD8_naive_primary_(UCSF-UBC) | H3K4me1 | 3.29 | 1.47 | 0.108954056 | Colorectal | Hematopoietic |
| CD4_memory_primary | H3K4me3 | 5.79 | 2.92 | 0.110744701 | Breast | Hematopoietic |
| CD56_primary | H3K4me1 | 2.67 | 1.07 | 0.111230534 | Prostate | Hematopoietic |
| Th0 | H3K27ac | 7.07 | 3.90 | 0.111265781 | Ovarian | Hematopoietic |
| Liver_(BI) | H3K4me1 | 4.31 | 2.01 | 0.111398368 | Ovarian | Liver |
| Aorta | H3K4me3 | 10.57 | 6.22 | 0.111655073 | Colorectal | Cardiovascular |
| CD56_primary | H3K4me3 | 10.25 | 6.01 | 0.112062598 | Colorectal | Hematopoietic |
| CD8_memory_primary | H3K4me1 | 3.14 | 1.33 | 0.112584252 | Breast | Hematopoietic |
| Adipose_nuclei | H3K4me3 | 3.55 | 1.63 | 0.112928969 | Prostate | Other |
| Placenta_amnion | H3K4me3 | 11.65 | 6.94 | 0.114377698 | Colorectal | Other |
| Rectal_smooth_muscle | H3K27ac | 4.86 | 2.36 | 0.114561299 | Ovarian | GI |
| CD25int_CD127+_Tmem | H3K27ac | 4.61 | 2.35 | 0.116132179 | Lung | Hematopoietic |
| Skeletal_muscle | H3K9ac | 4.79 | 2.33 | 0.117045161 | Lung | SkeletalMuscle |
| Rectal_smooth_muscle | H3K9ac | 12.69 | 7.23 | 0.11713477 | Breast | GI |
| Fetal_adrenal | H3K4me1 | 2.98 | 1.27 | 0.117262474 | Prostate | Adrenal_Pancreas |
| CD3_primary_(BI) | H3K4me3 | 8.80 | 4.69 | 0.120486051 | Lung | Hematopoietic |
| CD3_primary_(UW) | H3K4me3 | 6.38 | 3.53 | 0.120567434 | Prostate | Hematopoietic |
| Mid_frontal_lobe | H3K4me1 | 24.32 | 15.50 | 0.120889973 | Headneck | CNS |
| CD8_naive_primary_(UCSF-UBC) | H3K4me1 | 6.82 | 3.39 | 0.121194461 | Ovarian | Hematopoietic |
| CD8_naive_primary_(UCSF-UBC) | H3K4me3 | 8.76 | 5.05 | 0.121455965 | Prostate | Hematopoietic |
| Inferior_temporal_lobe | H3K9ac | 5.65 | 3.06 | 0.121785614 | Colorectal | CNS |
| Anterior_caudate | H3K9ac | 5.31 | 2.79 | 0.121975652 | Breast | CNS |
| Duodenum_smooth_muscle | H3K4me3 | 6.01 | 3.09 | 0.122396552 | Lung | GI |
| CD56_primary | H3K4me3 | 7.27 | 3.96 | 0.12326411 | Breast | Hematopoietic |
| Anterior_caudate | H3K4me1 | 3.40 | 1.59 | 0.123373671 | Lung | CNS |
| Colon_smooth_muscle | H3K4me1 | 5.03 | 2.58 | 0.123957818 | Ovarian | GI |
| CD4+_CD25int_CD127+_Tmem_primary | H3K4me1 | 10.59 | 5.62 | 0.124688512 | Ovarian | Hematopoietic |
| Fetal_large_intestine | H3K4me1 | 6.22 | 3.27 | 0.126800547 | Ovarian | GI |
| Anterior_caudate | H3K4me1 | 3.24 | 1.50 | 0.127480512 | Breast | CNS |
| Pancreatic_islets | H3K4me1 | 15.31 | 10.23 | 0.129689021 | Headneck | Adrenal_Pancreas |
| CD3_primary_(BI) | H3K4me3 | 5.05 | 2.60 | 0.129829969 | Breast | Hematopoietic |
| Ovary | H3K4me3 | 8.66 | 5.15 | 0.130274566 | Colorectal | Other |
| Placenta_chorion | H3K4me3 | 11.21 | 6.71 | 0.130784903 | Lung | Other |
| Th1 | H3K27ac | 6.41 | 3.57 | 0.131149782 | Ovarian | Hematopoietic |
| Inferior_temporal_lobe | H3K4me1 | 3.01 | 1.31 | 0.131379696 | Lung | CNS |
| Fetal_placenta | H3K4me3 | 11.60 | 6.94 | 0.131744407 | Lung | Other |
| CD25+_CD127-_Treg | H3K27ac | 2.60 | 1.05 | 0.132334077 | Prostate | Hematopoietic |
| Penis_foreskin_melanocyte_primary | H3K4me3 | 10.95 | 6.29 | 0.133146311 | Ovarian | Other |
| Colon_smooth_muscle | H3K27ac | 3.56 | 1.64 | 0.133717069 | Lung | GI |
| CD8_primary | H3K4me3 | 6.09 | 3.46 | 0.135944642 | Prostate | Hematopoietic |
| CD4_naive_primary | H3K4me1 | 2.98 | 1.31 | 0.136970386 | Breast | Hematopoietic |
| Rectal_mucosa | H3K4me3 | 17.57 | 10.04 | 0.137383586 | Ovarian | GI |
| Cingulate_gyrus | H3K4me1 | 3.16 | 1.45 | 0.138528147 | Lung | CNS |
| CD4+_CD25int_CD127+_Tmem_primary | H3K4me3 | 6.54 | 3.64 | 0.138571623 | Breast | Hematopoietic |
| Colon_smooth_muscle | H3K27ac | 4.80 | 2.47 | 0.139524428 | Ovarian | GI |
| CD25-_IL17-_Th_stim_MACS | H3K27ac | 6.77 | 4.03 | 0.139659161 | Headneck | Hematopoietic |
| Penis_foreskin_melanocyte_primary | H3K4me3 | 5.23 | 2.92 | 0.140104656 | Colorectal | Other |
| Inferior_temporal_lobe | H3K4me3 | 5.86 | 3.35 | 0.140860756 | Colorectal | CNS |
| CD56_primary | H3K4me1 | 3.33 | 1.58 | 0.141746053 | Breast | Hematopoietic |
| CD4+_CD25-_Th_primary | H3K4me3 | 5.34 | 2.89 | 0.143217279 | Breast | Hematopoietic |
| CD4_primary | H3K4me3 | 5.33 | 2.89 | 0.14415177 | Breast | Hematopoietic |
| CD20 | H3K27ac | 3.78 | 1.87 | 0.144386326 | Breast | Hematopoietic |
| CD20 | H3K27ac | 3.26 | 1.56 | 0.144766703 | Lung | Hematopoietic |
| Lung | H3K4me3 | 13.58 | 8.55 | 0.145148447 | Lung | Cardiovascular |
| Placenta_amnion | H3K4me1 | 21.54 | 13.83 | 0.145167233 | Breast | Other |
| Lung | H3K4me1 | 4.71 | 2.59 | 0.145178804 | Colorectal | Cardiovascular |
| Neurosphere | H3K27ac | 2.83 | 1.23 | 0.1452316 | Breast | CNS |
| Placenta_chorion | H3K4me1 | 9.37 | 5.76 | 0.146432116 | Breast | Other |
| Cingulate_gyrus | H3K9ac | 3.80 | 1.91 | 0.146455123 | Breast | CNS |
| Peripheralblood_mononuclear_primary | H3K4me3 | 8.26 | 5.19 | 0.146482152 | Colorectal | Hematopoietic |
| Right_atrium | H3K4me3 | 7.92 | 4.89 | 0.146756448 | Colorectal | Cardiovascular |
| Placenta_amnion | H3K4me3 | 14.18 | 8.80 | 0.146908679 | Lung | Other |
| Peripheralblood_mononuclear_primary | H3K4me1 | 10.56 | 6.39 | 0.147177281 | Lung | Hematopoietic |
| Kidney | H3K4me1 | 8.52 | 5.10 | 0.148264397 | Colorectal | Kidney |
| CD19 | H3K27ac | 3.73 | 1.86 | 0.148464285 | Breast | Hematopoietic |
| Hippocampus_middle | H3K4me1 | 2.76 | 1.24 | 0.148634425 | Breast | CNS |
| Colon_smooth_muscle | H3K4me3 | 7.94 | 4.77 | 0.153360704 | Lung | GI |
| Angular_gyrus | H3K4me3 | 9.14 | 5.75 | 0.153608895 | Lung | CNS |
| Stomach_smooth_muscle | H3K4me3 | 4.29 | 2.32 | 0.154364983 | Prostate | GI |
| CD8_naive_primary_(BI) | H3K4me1 | 2.77 | 1.23 | 0.154770663 | Breast | Hematopoietic |
| Fetal_small_intestine | H3K4me1 | 5.31 | 2.81 | 0.155419464 | Ovarian | GI |
| Substantia_nigra | H3K4me1 | 3.17 | 1.52 | 0.155732015 | Lung | CNS |
| Kidney | H3K9ac | 26.91 | 17.29 | 0.156692565 | Ovarian | Kidney |
| CD4+_CD25int_CD127+_Tmem_primary | H3K4me1 | 5.71 | 3.30 | 0.156935967 | Lung | Hematopoietic |
| Th2 | H3K27ac | 6.72 | 4.15 | 0.157812464 | Headneck | Hematopoietic |
| Fetal_trunk_muscle | H3K4me3 | 11.03 | 6.97 | 0.15827637 | Lung | SkeletalMuscle |
| Peripheralblood_mononuclear_primary | H3K9ac | 5.21 | 3.04 | 0.15836069 | Prostate | Hematopoietic |
| Stomach_smooth_muscle | H3K9ac | 6.63 | 3.92 | 0.159237772 | Lung | GI |
| CD3_primary_(BI) | H3K4me1 | 2.77 | 1.31 | 0.1598994 | Colorectal | Hematopoietic |
| Rectal_smooth_muscle | H3K4me3 | 21.04 | 13.14 | 0.160051885 | Ovarian | GI |
| Hippocampus_middle | H3K27ac | 7.48 | 4.64 | 0.160092841 | Headneck | CNS |
| Mid_frontal_lobe | H3K27ac | 8.23 | 5.34 | 0.161168038 | Headneck | CNS |
| Rectal_mucosa | H3K9ac | 14.17 | 8.64 | 0.162548119 | Ovarian | GI |
| Thymus | H3K4me1 | 7.39 | 4.35 | 0.162904478 | Lung | Hematopoietic |
| CD8_memory_primary | H3K4me3 | 5.07 | 2.87 | 0.163871934 | Breast | Hematopoietic |
| Breast_myoepithelial | H3K9ac | 10.74 | 7.06 | 0.164915138 | Prostate | Other |
| Skeletal_muscle | H3K4me1 | 2.78 | 1.22 | 0.165654123 | Lung | SkeletalMuscle |
| Fetal_stomach | H3K4me3 | 21.96 | 13.98 | 0.165864704 | Ovarian | GI |
| Mobilized_CD34 | H3K27ac | 3.27 | 1.66 | 0.166986907 | Lung | Hematopoietic |
| CD25+_CD127-_Treg | H3K27ac | 6.47 | 3.96 | 0.1682158 | Headneck | Hematopoietic |
| Rectal_mucosa | H3K4me1 | 6.56 | 3.80 | 0.168576917 | Ovarian | GI |
| CD25+_CD127-_Treg | H3K27ac | 4.59 | 2.53 | 0.16932749 | Ovarian | Hematopoietic |
| Treg_primary | H3K4me3 | 9.98 | 5.89 | 0.169640453 | Ovarian | Hematopoietic |
| Duodenum_smooth_muscle | H3K27ac | 8.43 | 5.61 | 0.169882971 | Headneck | GI |
| Fetal_kidney | H3K9ac | 18.43 | 12.45 | 0.170456868 | Lung | Kidney |
| Left_Ventricle | H3K4me1 | 2.89 | 1.39 | 0.170523168 | Prostate | Cardiovascular |
| Mid_frontal_lobe | H3K4me3 | 5.56 | 3.39 | 0.171469301 | Colorectal | CNS |
| CD19_primary_(BI) | H3K4me3 | 6.87 | 4.44 | 0.171505104 | Colorectal | Hematopoietic |
| CD8_naive_primary_(BI) | H3K4me3 | 5.05 | 2.89 | 0.171657416 | Breast | Hematopoietic |
| Pancreatic_islets | H3K27ac | 2.85 | 1.38 | 0.173395312 | Breast | Adrenal_Pancreas |
| Rectal_mucosa | H3K27ac | 6.72 | 4.43 | 0.17351501 | Headneck | GI |
| CD8_naive_primary_(BI) | H3K4me3 | 5.69 | 3.50 | 0.173765787 | Prostate | Hematopoietic |
| Germinal_matrix | H3K4me3 | 5.71 | 3.51 | 0.173871273 | Prostate | CNS |
| Colon_smooth_muscle | H3K4me1 | 3.81 | 2.02 | 0.174076104 | Lung | GI |
| Substantia_nigra | H3K4me1 | 2.91 | 1.43 | 0.174316333 | Breast | CNS |
| Lung | H3K4me1 | 3.65 | 2.00 | 0.175440097 | Prostate | Cardiovascular |
| CD4+_CD25-_IL17-_PMA_Ionomycin_stim_MACS_Th_sprimary | H3K4me3 | 3.84 | 2.13 | 0.175732166 | Prostate | Hematopoietic |
| Penis_foreskin_keratinocyte_primary | H3K9ac | 3.69 | 1.99 | 0.176288782 | Lung | Other |
| Inferior_temporal_lobe | H3K9ac | 10.21 | 7.02 | 0.17631155 | Headneck | CNS |
| CD56_primary | H3K4me3 | 7.63 | 4.99 | 0.176739207 | Prostate | Hematopoietic |
| CD25-_IL17-_Th_stim_MACS | H3K27ac | 2.46 | 1.08 | 0.17693465 | Prostate | Hematopoietic |
| CD4+_CD25int_CD127+_Tmem_primary | H3K4me1 | 4.05 | 2.33 | 0.176999455 | Colorectal | Hematopoietic |
| Substantia_nigra | H3K9ac | 5.07 | 3.07 | 0.17744668 | Colorectal | CNS |
| Angular_gyrus | H3K27ac | 3.31 | 1.84 | 0.178198489 | Lung | CNS |
| Chondrogenic_dif | H3K27ac | 3.00 | 1.48 | 0.178633341 | Prostate | Connective_Bone |
| CD4+_CD25-_CD45RA+_naive_primary | H3K4me3 | 4.86 | 2.81 | 0.178821843 | Breast | Hematopoietic |
| Fetal_lung | H3K4me3 | 16.61 | 11.10 | 0.181917041 | Ovarian | Cardiovascular |
| CD19_primary_(UW) | H3K4me3 | 8.26 | 5.52 | 0.183488785 | Prostate | Hematopoietic |
| Peripheralblood_mononuclear_primary | H3K9ac | 5.79 | 3.72 | 0.184145945 | Colorectal | Hematopoietic |
| Colon_smooth_muscle | H3K9ac | 7.32 | 4.71 | 0.184866132 | Prostate | GI |
| Fetal_brain | H3K4me1 | 2.65 | 1.32 | 0.186447409 | Lung | CNS |
| Substantia_nigra | H3K4me3 | 5.92 | 3.79 | 0.189797641 | Colorectal | CNS |
| Substantia_nigra | H3K9ac | 4.66 | 2.81 | 0.190298161 | Breast | CNS |
| Penis_foreskin_keratinocyte_primary | H3K4me3 | 5.83 | 3.58 | 0.191679323 | Lung | Other |
| CD15_primary | H3K4me3 | 5.92 | 3.83 | 0.191834913 | Colorectal | Hematopoietic |
| Breast_fibroblast_primary | H3K4me3 | 8.20 | 5.58 | 0.193143692 | Prostate | Connective_Bone |
| CD3_primary_(UW) | H3K4me3 | 5.42 | 3.33 | 0.193480103 | Breast | Hematopoietic |
| Right_ventricle | H3K4me1 | 51.24 | 38.47 | 0.193780496 | Breast | Cardiovascular |
| CD34_primary | H3K4me1 | 3.64 | 2.10 | 0.194364216 | Colorectal | Hematopoietic |
| CD4_naive_primary | H3K4me3 | 11.75 | 7.47 | 0.195865893 | Ovarian | Hematopoietic |
| Stomach_smooth_muscle | H3K4me1 | 2.87 | 1.44 | 0.196369519 | Prostate | GI |
| Penis_foreskin_melanocyte_primary | H3K4me3 | 5.26 | 3.20 | 0.196607029 | Lung | Other |
| Psoas_muscle | H3K4me1 | 12.57 | 8.96 | 0.197901976 | Breast | SkeletalMuscle |
| Gastric | H3K4me3 | 15.02 | 10.65 | 0.198458001 | Lung | GI |
| Kidney | H3K4me3 | 5.63 | 3.56 | 0.199174533 | Lung | Kidney |
| Hippocampus_middle | H3K9ac | 3.69 | 2.11 | 0.200361669 | Breast | CNS |
| Breast_luminal_epithelial | H3K4me1 | 29.48 | 21.71 | 0.201311162 | Colorectal | Other |
| Angular_gyrus | H3K4me1 | 4.85 | 3.13 | 0.201327833 | Lung | CNS |
| Colonic_mucosa | H3K9ac | 11.43 | 7.48 | 0.201851856 | Ovarian | GI |
| Hippocampus_middle | H3K4me1 | 2.71 | 1.31 | 0.202293635 | Lung | CNS |
| Liver_(UCSD) | H3K4me3 | 7.61 | 5.10 | 0.202735693 | Lung | Liver |
| Breast_fibroblast_primary | H3K4me3 | 6.65 | 4.39 | 0.202958718 | Breast | Connective_Bone |
| Osteoblast | H3K27ac | 3.57 | 2.26 | 0.203394287 | Ovarian | Connective_Bone |
| Spleen | H3K4me3 | 9.26 | 6.59 | 0.204291662 | Prostate | Hematopoietic |
| CD4_naive_primary | H3K4me3 | 4.58 | 2.78 | 0.204917525 | Breast | Hematopoietic |
| Small_intestine | H3K4me1 | 24.67 | 18.63 | 0.205100255 | Breast | GI |
| Sigmoid_colon | H3K4me1 | 17.17 | 12.88 | 0.205311462 | Breast | GI |
| CD3_primary_(BI) | H3K4me1 | 6.03 | 3.99 | 0.206166328 | Headneck | Hematopoietic |
| Aorta | H3K4me3 | 10.70 | 7.55 | 0.206345459 | Lung | Cardiovascular |
| Duodenum_smooth_muscle | H3K27ac | 5.72 | 3.68 | 0.206470205 | Ovarian | GI |
| Mid_frontal_lobe | H3K9ac | 5.31 | 3.55 | 0.206810304 | Lung | CNS |
| Fetal_brain | H3K9ac | 6.59 | 4.44 | 0.206913353 | Lung | CNS |
| CD4+_CD25-_IL17-_PMA_Ionomycin_stim_MACS_Th_sprimary | H3K4me3 | 4.18 | 2.59 | 0.209747952 | Colorectal | Hematopoietic |
| Substantia_nigra | H3K4me3 | 4.10 | 2.48 | 0.210826485 | Breast | CNS |
| Skeletal_muscle | H3K4me3 | 5.00 | 3.15 | 0.211183808 | Lung | SkeletalMuscle |
| Rectal_mucosa | H3K4me3 | 11.01 | 8.31 | 0.211361184 | Headneck | GI |
| CD8_primary | H3K4me3 | 5.21 | 3.30 | 0.211802429 | Breast | Hematopoietic |
| CD8_naive_primary_(UCSF-UBC) | H3K4me3 | 6.47 | 4.29 | 0.212023848 | Breast | Hematopoietic |
| CD3_primary_(UW) | H3K4me1 | 5.66 | 3.69 | 0.213179957 | Headneck | Hematopoietic |
| Stomach_mucosa | H3K9ac | 12.75 | 8.71 | 0.213655935 | Ovarian | GI |
| CD4+_CD25-_IL17+_PMA_Ionomycin_stim_Th17_primary | H3K4me3 | 4.34 | 2.73 | 0.215001714 | Prostate | Hematopoietic |
| Colonic_mucosa | H3K27ac | 6.19 | 4.35 | 0.215686628 | Headneck | GI |
| CD19 | H3K27ac | 3.09 | 1.70 | 0.215851729 | Lung | Hematopoietic |
| Sigmoid_colon | H3K4me3 | 25.43 | 18.49 | 0.216055117 | Ovarian | GI |
| Breast_myoepithelial | H3K9ac | 10.87 | 8.18 | 0.218371417 | Colorectal | Other |
| CD3_primary | H3K27ac | 2.12 | 0.91 | 0.222221676 | Prostate | Hematopoietic |
| Fetal_kidney | H3K9ac | 41.65 | 32.48 | 0.22497218 | Ovarian | Kidney |
| CD4+_CD25int_CD127+_Tmem_primary | H3K4me3 | 6.57 | 4.77 | 0.226558542 | Colorectal | Hematopoietic |
| Th2 | H3K27ac | 6.87 | 4.84 | 0.226941535 | Ovarian | Hematopoietic |
| Breast_myoepithelial | H3K4me3 | 11.92 | 9.64 | 0.228553757 | Headneck | Other |
| Mobilized_CD34_primary | H3K4me1 | 3.68 | 2.13 | 0.230099033 | Ovarian | Hematopoietic |
| Mid_frontal_lobe | H3K27ac | 5.02 | 3.48 | 0.23101475 | Ovarian | CNS |
| Fetal_stomach | H3K4me1 | 3.01 | 1.68 | 0.232147166 | Prostate | GI |
| Colonic_mucosa | H3K4me3 | 18.72 | 13.62 | 0.233202408 | Ovarian | GI |
| CD4+_CD25-_CD45R0+_memory_primary | H3K4me3 | 5.42 | 3.74 | 0.233472404 | Prostate | Hematopoietic |
| Pancreatic_islets | H3K4me1 | 9.74 | 8.05 | 0.23710532 | Ovarian | Adrenal_Pancreas |
| CD4+_CD25-_Th_primary | H3K4me3 | 5.48 | 3.92 | 0.239852072 | Colorectal | Hematopoietic |
| Fetal_heart | H3K4me1 | 2.52 | 1.28 | 0.23992188 | Lung | Cardiovascular |
| Duodenum_smooth_muscle | H3K4me3 | 8.15 | 5.61 | 0.24010369 | Ovarian | GI |
| Peripheralblood_mononuclear_primary | H3K4me1 | 5.36 | 3.79 | 0.2401868 | Prostate | Hematopoietic |
| Hippocampus_middle | H3K4me3 | 3.17 | 1.87 | 0.240846622 | Breast | CNS |
| Mid_frontal_lobe | H3K4me1 | 7.14 | 5.31 | 0.24179507 | Breast | CNS |
| CD4+_CD25-_IL17-_PMA_Ionomycin_stim_MACS_Th_sprimary | H3K4me3 | 7.87 | 5.37 | 0.243049407 | Ovarian | Hematopoietic |
| CD4+_CD25-_IL17+_PMA_Ionomycin_stim_Th17_primary | H3K4me3 | 5.31 | 3.80 | 0.243160717 | Colorectal | Hematopoietic |
| CD4_primary | H3K4me3 | 5.14 | 3.63 | 0.243176126 | Colorectal | Hematopoietic |
| Angular_gyrus | H3K9ac | 12.71 | 10.28 | 0.243496836 | Headneck | CNS |
| Anterior_caudate | H3K4me3 | 3.21 | 1.90 | 0.243998729 | Breast | CNS |
| Mid_frontal_lobe | H3K4me3 | 3.48 | 2.12 | 0.244034685 | Breast | CNS |
| Liver | H3K27ac | 2.67 | 1.44 | 0.248701194 | Colorectal | Liver |
| Stomach_smooth_muscle | H3K4me1 | 4.19 | 2.72 | 0.251390378 | Lung | GI |
| Pancreatic_islets | H3K27ac | 3.78 | 2.56 | 0.252508053 | Ovarian | Adrenal_Pancreas |
| Chondrogenic_dif | H3K27ac | 3.78 | 2.58 | 0.252793231 | Ovarian | Connective_Bone |
| CD14_primary | H3K4me1 | 2.75 | 1.56 | 0.25330096 | Colorectal | Hematopoietic |
| Duodenum_smooth_muscle | H3K27ac | 2.83 | 1.59 | 0.254154456 | Prostate | GI |
| Sigmoid_colon | H3K4me1 | 13.28 | 10.94 | 0.255738525 | Colorectal | GI |
| Anterior_caudate | H3K9ac | 4.91 | 3.48 | 0.255974226 | Colorectal | CNS |
| Fetal_thymus | H3K4me3 | 6.24 | 4.73 | 0.256580863 | Colorectal | Hematopoietic |
| Liver_(BI) | H3K4me3 | 7.34 | 5.32 | 0.258022391 | Ovarian | Liver |
| Anterior_caudate | H3K9ac | 11.12 | 9.30 | 0.258841761 | Headneck | CNS |
| Skeletal_muscle | H3K4me3 | 3.40 | 2.14 | 0.261128464 | Prostate | SkeletalMuscle |
| Duodenum_Mucosa | H3K4me1 | 2.79 | 1.71 | 0.261447879 | Lung | GI |
| Angular_gyrus | H3K4me3 | 6.62 | 5.10 | 0.261780811 | Colorectal | CNS |
| Colon_smooth_muscle | H3K9ac | 9.34 | 7.37 | 0.263484911 | Lung | GI |
| CD15_primary | H3K4me1 | 2.75 | 1.58 | 0.26389614 | Colorectal | Hematopoietic |
| Psoas_muscle | H3K4me3 | 7.94 | 6.24 | 0.265350779 | Lung | SkeletalMuscle |
| Hippocampus_middle | H3K27ac | 4.51 | 3.14 | 0.26738951 | Ovarian | CNS |
| CD4_memory_primary | H3K4me3 | 4.17 | 2.89 | 0.267696458 | Prostate | Hematopoietic |
| Fetal_heart | H3K9ac | 2.94 | 1.78 | 0.268995504 | Prostate | Cardiovascular |
| Duodenum_smooth_muscle | H3K4me1 | 5.40 | 4.09 | 0.270327139 | Prostate | GI |
| Fetal_leg_muscle | H3K4me3 | 13.15 | 10.48 | 0.271816919 | Ovarian | SkeletalMuscle |
| Inferior_temporal_lobe | H3K27ac | 3.84 | 2.66 | 0.273493172 | Ovarian | CNS |
| Fetal_lung | H3K4me1 | 3.02 | 1.81 | 0.277460423 | Ovarian | Cardiovascular |
| Right_atrium | H3K4me1 | 5.85 | 4.52 | 0.278684334 | Colorectal | Cardiovascular |
| Th0 | H3K27ac | 5.32 | 3.96 | 0.27971701 | Headneck | Hematopoietic |
| Pancreas | H3K4me3 | 8.09 | 6.56 | 0.280826156 | Lung | Adrenal_Pancreas |
| CD8_memory_primary | H3K4me1 | 5.05 | 3.75 | 0.281717681 | Headneck | Hematopoietic |
| Stomach_smooth_muscle | H3K9ac | 3.66 | 2.47 | 0.281802127 | Prostate | GI |
| Angular_gyrus | H3K9ac | 5.72 | 4.47 | 0.282898496 | Colorectal | CNS |
| CD20 | H3K27ac | 4.14 | 3.01 | 0.283548448 | Headneck | Hematopoietic |
| Kidney | H3K4me1 | 8.48 | 7.36 | 0.286213584 | Ovarian | Kidney |
| Placenta_chorion | H3K4me1 | 8.50 | 7.02 | 0.292522574 | Colorectal | Other |
| CD25-_IL17+_Th17_stim | H3K27ac | 2.54 | 1.46 | 0.294725789 | Breast | Hematopoietic |
| CD19 | H3K27ac | 4.30 | 3.23 | 0.296295108 | Headneck | Hematopoietic |
| Duodenum_Mucosa | H3K4me1 | 3.55 | 2.40 | 0.297930068 | Ovarian | GI |
| Anterior_caudate | H3K4me3 | 4.28 | 3.19 | 0.298866954 | Colorectal | CNS |
| Skeletal_muscle | H3K27ac | 2.84 | 1.77 | 0.298944231 | Lung | SkeletalMuscle |
| CD8_memory_primary | H3K4me3 | 4.26 | 3.18 | 0.300666556 | Prostate | Hematopoietic |
| Adipose_nuclei | H3K4me3 | 3.48 | 2.36 | 0.303270961 | Lung | Other |
| Angular_gyrus | H3K27ac | 3.90 | 2.90 | 0.30346161 | Ovarian | CNS |
| Substantia_nigra | H3K9ac | 7.87 | 6.84 | 0.304322038 | Headneck | CNS |
| CD4+_CD25+_CD127-_Treg_primary | H3K4me1 | 5.28 | 4.12 | 0.304359469 | Headneck | Hematopoietic |
| Fetal_heart | H3K4me3 | 6.06 | 4.97 | 0.305459139 | Colorectal | Cardiovascular |
| Liver_(BI) | H3K4me1 | 2.20 | 1.15 | 0.307349376 | Lung | Liver |
| Pancreatic_islets | H3K4me3 | 5.58 | 4.59 | 0.309047613 | Colorectal | Adrenal_Pancreas |
| Adipose_nuclei | H3K9ac | 3.98 | 2.87 | 0.309137203 | Lung | Other |
| CD3_primary_(UW) | H3K4me3 | 5.44 | 4.49 | 0.311210532 | Colorectal | Hematopoietic |
| Fetal_lung | H3K4me3 | 6.92 | 5.78 | 0.311785101 | Lung | Cardiovascular |
| Fetal_trunk_muscle | H3K4me3 | 13.25 | 11.59 | 0.313464869 | Ovarian | SkeletalMuscle |
| Hippocampus_middle | H3K4me3 | 4.21 | 3.22 | 0.315235114 | Colorectal | CNS |
| Cingulate_gyrus | H3K9ac | 7.24 | 6.30 | 0.315241447 | Headneck | CNS |
| CD56_primary | H3K4me1 | 4.82 | 3.78 | 0.315352851 | Headneck | Hematopoietic |
| CD8_naive_primary_(UCSF-UBC) | H3K9ac | 9.85 | 8.91 | 0.318060095 | Prostate | Hematopoietic |
| CD4+_CD25-_CD45R0+_memory_primary | H3K4me3 | 5.82 | 4.96 | 0.318352333 | Colorectal | Hematopoietic |
| Germinal_matrix | H3K4me3 | 12.27 | 11.66 | 0.319365543 | Headneck | CNS |
| Fetal_brain | H3K4me3 | 6.37 | 5.40 | 0.31970376 | Breast | CNS |
| Mid_frontal_lobe | H3K9ac | 3.38 | 2.39 | 0.321231906 | Breast | CNS |
| Liver_(BI) | H3K4me3 | 4.01 | 3.00 | 0.321685856 | Lung | Liver |
| Neurosphere | H3K27ac | 2.44 | 1.46 | 0.322614347 | Prostate | CNS |
| Right_atrium | H3K4me3 | 10.42 | 9.06 | 0.322900125 | Ovarian | Cardiovascular |
| Angular_gyrus | H3K4me1 | 3.74 | 2.81 | 0.323302886 | Colorectal | CNS |
| Thymus | H3K4me1 | 11.76 | 10.81 | 0.323952279 | Headneck | Hematopoietic |
| Adipose_nuclei | H3K27ac | 4.05 | 3.20 | 0.326552292 | Headneck | Other |
| CD4_memory_primary | H3K4me3 | 4.72 | 3.88 | 0.327642278 | Colorectal | Hematopoietic |
| Rectal_smooth_muscle | H3K4me1 | 5.29 | 4.39 | 0.329160869 | Lung | GI |
| CD25-_IL17+_Th17_stim | H3K27ac | 6.94 | 6.01 | 0.329219645 | Ovarian | Hematopoietic |
| Pancreatic_islets | H3K4me3 | 4.23 | 3.33 | 0.329435743 | Breast | Adrenal_Pancreas |
| Fetal_thymus | H3K4me1 | 2.26 | 1.32 | 0.331342049 | Colorectal | Hematopoietic |
| Cingulate_gyrus | H3K4me1 | 2.22 | 1.28 | 0.332460778 | Breast | CNS |
| Rectal_smooth_muscle | H3K4me1 | 3.66 | 2.75 | 0.33536643 | Prostate | GI |
| Breast_vHMEC | H3K4me3 | 6.16 | 5.41 | 0.337336819 | Colorectal | Other |
| Small_intestine | H3K4me3 | 21.49 | 20.57 | 0.337442216 | Ovarian | GI |
| Skeletal_muscle | H3K4me3 | 6.21 | 5.17 | 0.340086633 | Ovarian | SkeletalMuscle |
| CD19_primary_(BI) | H3K4me1 | 3.17 | 2.21 | 0.342023577 | Ovarian | Hematopoietic |
| CD8_naive_primary_(UCSF-UBC) | H3K4me1 | 2.33 | 1.40 | 0.343456389 | Breast | Hematopoietic |
| CD8_memory_primary | H3K4me3 | 9.48 | 8.41 | 0.343753086 | Ovarian | Hematopoietic |
| Ovary | H3K4me3 | 7.87 | 7.23 | 0.345472408 | Lung | Other |
| Th1 | H3K27ac | 4.41 | 3.56 | 0.346349814 | Headneck | Hematopoietic |
| Pancreatic_islets | H3K4me3 | 6.12 | 5.41 | 0.347749587 | Lung | Adrenal_Pancreas |
| CD8_naive_primary_(UCSF-UBC) | H3K4me3 | 6.94 | 6.47 | 0.34830981 | Colorectal | Hematopoietic |
| Mid_frontal_lobe | H3K9ac | 7.97 | 7.55 | 0.348791018 | Headneck | CNS |
| CD25int_CD127+_Tmem | H3K27ac | 3.61 | 2.78 | 0.351944872 | Ovarian | Hematopoietic |
| Cingulate_gyrus | H3K4me3 | 4.03 | 3.27 | 0.351949321 | Colorectal | CNS |
| Adipose_nuclei | H3K27ac | 2.08 | 1.15 | 0.352000693 | Prostate | Other |
| CD4+_CD25-_CD45RA+_naive_primary | H3K4me3 | 4.26 | 3.58 | 0.352098555 | Colorectal | Hematopoietic |
| Mobilized_CD34 | H3K27ac | 2.60 | 1.78 | 0.353063255 | Colorectal | Hematopoietic |
| Fetal_lung | H3K4me3 | 11.95 | 12.01 | 0.355112878 | Headneck | Cardiovascular |
| Mobilized_CD34 | H3K27ac | 2.89 | 2.07 | 0.357053491 | Breast | Hematopoietic |
| Breast_vHMEC | H3K4me3 | 7.68 | 7.21 | 0.358452817 | Lung | Other |
| Liver_(UCSD) | H3K4me3 | 9.14 | 8.65 | 0.359467452 | Ovarian | Liver |
| CD8_primary | H3K4me3 | 4.87 | 4.31 | 0.3598718 | Colorectal | Hematopoietic |
| Fetal_trunk_muscle | H3K4me1 | 2.73 | 1.83 | 0.360645737 | Lung | SkeletalMuscle |
| Pancreatic_islets | H3K9ac | 5.20 | 4.59 | 0.361642923 | Breast | Adrenal_Pancreas |
| CD25int_CD127+_Tmem | H3K27ac | 4.67 | 3.99 | 0.361795592 | Headneck | Hematopoietic |
| Inferior_temporal_lobe | H3K4me3 | 8.60 | 8.61 | 0.365755379 | Headneck | CNS |
| CD56_primary | H3K4me1 | 3.54 | 2.68 | 0.366701154 | Ovarian | Hematopoietic |
| Colonic_mucosa | H3K27ac | 2.92 | 2.31 | 0.366977402 | Lung | GI |
| Ovary | H3K4me1 | 5.18 | 4.65 | 0.367509211 | Colorectal | Other |
| Stomach_smooth_muscle | H3K27ac | 2.48 | 1.61 | 0.368678452 | Lung | GI |
| Inferior_temporal_lobe | H3K4me1 | 2.01 | 1.14 | 0.368963016 | Breast | CNS |
| Anterior_caudate | H3K27ac | 4.75 | 4.19 | 0.371698619 | Headneck | CNS |
| Breast_fibroblast_primary | H3K4me3 | 10.57 | 10.77 | 0.376217323 | Lung | Connective_Bone |
| Duodenum_smooth_muscle | H3K27ac | 2.81 | 2.05 | 0.378369159 | Lung | GI |
| Fetal_brain | H3K4me3 | 6.58 | 6.34 | 0.37845898 | Colorectal | CNS |
| Liver_(BI) | H3K9ac | 4.17 | 3.57 | 0.378952728 | Lung | Liver |
| Duodenum_Mucosa | H3K9ac | 8.18 | 7.82 | 0.380139331 | Ovarian | GI |
| CD14_primary | H3K4me1 | 2.99 | 2.30 | 0.380288031 | Ovarian | Hematopoietic |
| CD25+_CD127-_Treg | H3K27ac | 2.37 | 1.55 | 0.380752534 | Breast | Hematopoietic |
| Liver_(UCSD) | H3K4me1 | 17.37 | 18.74 | 0.382006555 | Colorectal | Liver |
| Pancreatic_islets | H3K4me1 | 5.55 | 5.25 | 0.384122659 | Breast | Adrenal_Pancreas |
| Ovary | H3K4me1 | 9.19 | 10.03 | 0.385215699 | Ovarian | Other |
| Angular_gyrus | H3K4me3 | 11.37 | 12.25 | 0.385496009 | Headneck | CNS |
| CD4+_CD25int_CD127+_Tmem_primary | H3K4me3 | 9.82 | 9.81 | 0.388405269 | Ovarian | Hematopoietic |
| Fetal_heart | H3K4me3 | 4.49 | 4.07 | 0.390660781 | Breast | Cardiovascular |
| CD3_primary_(BI) | H3K4me3 | 7.47 | 7.39 | 0.390750992 | Ovarian | Hematopoietic |
| Inferior_temporal_lobe | H3K4me3 | 2.65 | 1.93 | 0.391273265 | Breast | CNS |
| Cingulate_gyrus | H3K4me3 | 2.81 | 2.12 | 0.392939299 | Breast | CNS |
| CD4+_CD25-_CD45RA+_naive_primary | H3K4me3 | 8.08 | 8.00 | 0.392985334 | Ovarian | Hematopoietic |
| Colon_smooth_muscle | H3K27ac | 2.01 | 1.17 | 0.393945869 | Prostate | GI |
| Rectal_smooth_muscle | H3K27ac | 3.89 | 3.40 | 0.395029114 | Headneck | GI |
| CD19 | H3K27ac | 1.92 | 1.09 | 0.395237226 | Prostate | Hematopoietic |
| CD34_primary | H3K4me3 | 10.01 | 10.51 | 0.397933157 | Ovarian | Hematopoietic |
| CD8_naive_primary_(BI) | H3K4me1 | 4.20 | 3.76 | 0.39880004 | Headneck | Hematopoietic |
| Pancreatic_islets | H3K4me1 | 9.83 | 10.53 | 0.399651241 | Breast | Adrenal_Pancreas |
| Inferior_temporal_lobe | H3K9ac | 2.72 | 2.07 | 0.406129202 | Breast | CNS |
| CD25-_CD45RA+_naive | H3K27ac | 0.23 | 0.95 | 0.40836808 | Breast | Hematopoietic |
| Pancreatic_islets | H3K4me3 | 7.93 | 8.36 | 0.408718193 | Ovarian | Adrenal_Pancreas |
| Neurosphere | H3K27ac | 2.35 | 1.61 | 0.409166869 | Lung | CNS |
| Fetal_placenta | H3K4me3 | 10.22 | 10.74 | 0.411607628 | Ovarian | Other |
| Breast_myoepithelial | H3K9ac | 10.84 | 11.88 | 0.412571708 | Lung | Other |
| Rectal_smooth_muscle | H3K27ac | 1.94 | 1.13 | 0.413244654 | Prostate | GI |
| CD4+_CD25-_CD45R0+_memory_primary | H3K4me3 | 10.52 | 11.18 | 0.414141594 | Ovarian | Hematopoietic |
| Fetal_heart | H3K4me3 | 7.96 | 8.60 | 0.415993613 | Lung | Cardiovascular |
| CD4_naive_primary | H3K4me1 | 3.99 | 3.64 | 0.416367752 | Headneck | Hematopoietic |
| Placenta_amnion | H3K4me1 | 9.49 | 10.43 | 0.418064058 | Colorectal | Other |
| Fetal_brain | H3K9ac | 3.45 | 3.09 | 0.423158127 | Prostate | CNS |
| Anterior_caudate | H3K4me1 | 3.64 | 3.33 | 0.425263034 | Headneck | CNS |
| Substantia_nigra | H3K4me3 | 8.86 | 10.05 | 0.425751161 | Headneck | CNS |
| Fetal_stomach | H3K4me3 | 11.03 | 12.94 | 0.427135365 | Headneck | GI |
| Small_intestine | H3K4me1 | 17.97 | 21.70 | 0.431019531 | Prostate | GI |
| Substantia_nigra | H3K27ac | 2.99 | 2.54 | 0.431373287 | Ovarian | CNS |
| CD3_primary_(BI) | H3K4me3 | 3.74 | 3.56 | 0.434772611 | Colorectal | Hematopoietic |
| Fetal_adrenal | H3K4me3 | 10.62 | 12.10 | 0.437544916 | Ovarian | Adrenal_Pancreas |
| Psoas_muscle | H3K4me3 | 9.16 | 10.25 | 0.437576169 | Ovarian | SkeletalMuscle |
| Th0 | H3K27ac | 0.34 | 0.88 | 0.441915313 | Breast | Hematopoietic |
| Angular_gyrus | H3K4me3 | 3.28 | 2.97 | 0.442545963 | Breast | CNS |
| Hippocampus_middle | H3K4me3 | 6.94 | 7.93 | 0.450119461 | Headneck | CNS |
| Colon_smooth_muscle | H3K9ac | 13.41 | 16.41 | 0.451283744 | Ovarian | GI |
| CD8_naive_primary_(BI) | H3K4me3 | 7.46 | 8.32 | 0.451806948 | Ovarian | Hematopoietic |
| Hippocampus_middle | H3K4me3 | 3.00 | 2.70 | 0.454086257 | Prostate | CNS |
| CD4+_CD25-_CD45RA+_naive_primary | H3K4me1 | 3.39 | 3.17 | 0.454788741 | Headneck | Hematopoietic |
| Substantia_nigra | H3K4me3 | 3.45 | 3.31 | 0.455272742 | Prostate | CNS |
| Anterior_caudate | H3K4me3 | 6.94 | 8.06 | 0.45738627 | Headneck | CNS |
| CD4+_CD25-_Th_primary | H3K4me3 | 6.83 | 7.63 | 0.45915831 | Ovarian | Hematopoietic |
| CD25int_CD127+_Tmem | H3K27ac | 2.69 | 2.30 | 0.461768916 | Breast | Hematopoietic |
| Right_ventricle | H3K4me1 | 12.20 | 15.29 | 0.464930011 | Prostate | Cardiovascular |
| Pancreas | H3K4me3 | 9.58 | 11.48 | 0.465493219 | Ovarian | Adrenal_Pancreas |
| Adipose_nuclei | H3K4me3 | 4.17 | 4.21 | 0.466312859 | Ovarian | Other |
| Anterior_caudate | H3K4me1 | 0.12 | 1.20 | 0.467130264 | Prostate | CNS |
| CD8_naive_primary_(BI) | H3K4me3 | 3.79 | 3.94 | 0.469324661 | Colorectal | Hematopoietic |
| Cingulate_gyrus | H3K27ac | 2.91 | 2.66 | 0.470580204 | Ovarian | CNS |
| Fetal_adrenal | H3K4me1 | 2.74 | 2.44 | 0.476098265 | Lung | Adrenal_Pancreas |
| CD4+_CD25-_CD45R0+_memory_primary | H3K4me1 | 3.32 | 3.23 | 0.478403729 | Headneck | Hematopoietic |
| Substantia_nigra | H3K27ac | 0.18 | 1.15 | 0.479682642 | Breast | CNS |
| Th0 | H3K27ac | 0.40 | 0.87 | 0.486835226 | Prostate | Hematopoietic |
| Fetal_thymus | H3K4me1 | 3.54 | 3.62 | 0.489404582 | Headneck | Hematopoietic |
| Hippocampus_middle | H3K9ac | 5.70 | 6.77 | 0.489420538 | Headneck | CNS |
| Fetal_heart | H3K4me1 | 1.61 | 0.91 | 0.494496221 | Prostate | Cardiovascular |
| CD4+_CD25-_Th_primary | H3K4me1 | 3.01 | 2.91 | 0.494630306 | Headneck | Hematopoietic |
| Osteoblast | H3K27ac | 0.03 | 1.48 | 0.496527288 | Lung | Connective_Bone |
| Mid_frontal_lobe | H3K27ac | 2.28 | 1.98 | 0.498136851 | Lung | CNS |
| Esophagus | H3K4me1 | 9.52 | 12.71 | 0.49938844 | Colorectal | GI |
| Th2 | H3K27ac | 2.20 | 1.78 | 0.502035805 | Colorectal | Hematopoietic |
| Thymus | H3K4me1 | 2.75 | 2.68 | 0.511258042 | Prostate | Hematopoietic |
| Th1 | H3K27ac | 2.09 | 1.66 | 0.511436504 | Colorectal | Hematopoietic |
| CD4+_CD25+_CD127-_Treg_primary | H3K4me3 | 6.07 | 7.57 | 0.51171432 | Ovarian | Hematopoietic |
| Mid_frontal_lobe | H3K4me1 | 11.09 | 15.72 | 0.514709487 | Ovarian | CNS |
| CD8_naive_primary_(UCSF-UBC) | H3K4me1 | 3.74 | 4.20 | 0.518978455 | Headneck | Hematopoietic |
| Mid_frontal_lobe | H3K4me3 | 2.80 | 2.84 | 0.52263995 | Prostate | CNS |
| Mobilized_CD34_primary | H3K4me3 | 5.19 | 6.57 | 0.525673353 | Ovarian | Hematopoietic |
| CD4_memory_primary | H3K4me3 | 6.42 | 8.52 | 0.529872318 | Ovarian | Hematopoietic |
| Pancreatic_islets | H3K4me3 | 6.81 | 9.11 | 0.53172801 | Ovarian | Adrenal_Pancreas |
| Angular_gyrus | H3K27ac | 0.08 | 1.47 | 0.532095349 | Colorectal | CNS |
| CD4_naive_primary | H3K4me3 | 3.19 | 3.59 | 0.534857929 | Colorectal | Hematopoietic |
| Adipose_nuclei | H3K4me1 | 1.97 | 1.58 | 0.535402458 | Lung | Other |
| Fetal_brain | H3K4me3 | 5.19 | 6.83 | 0.537429537 | Prostate | CNS |
| Liver_(UCSD) | H3K4me1 | 9.14 | 13.36 | 0.541666596 | Prostate | Liver |
| Penis_foreskin_fibroblast_primary | H3K4me1 | 1.95 | 1.52 | 0.541842243 | Ovarian | Connective_Bone |
| Spleen | H3K4me3 | 8.13 | 11.81 | 0.544115973 | Lung | Hematopoietic |
| Cingulate_gyrus | H3K4me3 | 6.02 | 8.46 | 0.548786748 | Headneck | CNS |
| Kidney | H3K27ac | 3.13 | 3.52 | 0.549159419 | Headneck | Kidney |
| CD8_memory_primary | H3K4me3 | 3.31 | 3.93 | 0.549647611 | Colorectal | Hematopoietic |
| Right_ventricle | H3K4me1 | 14.41 | 22.74 | 0.551050823 | Colorectal | Cardiovascular |
| Spleen | H3K4me3 | 6.25 | 9.01 | 0.555726807 | Colorectal | Hematopoietic |
| Fetal_brain | H3K4me3 | 12.29 | 19.48 | 0.55879553 | Headneck | CNS |
| Inferior_temporal_lobe | H3K4me1 | 2.61 | 2.76 | 0.560366985 | Headneck | CNS |
| Angular_gyrus | H3K4me1 | 2.43 | 2.49 | 0.560628055 | Breast | CNS |
| Substantia_nigra | H3K4me1 | 2.77 | 3.05 | 0.562882213 | Headneck | CNS |
| CD15_primary | H3K4me1 | 2.18 | 2.12 | 0.566345771 | Ovarian | Hematopoietic |
| Fetal_large_intestine | H3K4me3 | 7.36 | 11.37 | 0.570269574 | Headneck | GI |
| Pancreatic_islets | H3K27ac | 1.90 | 1.61 | 0.570444324 | Lung | Adrenal_Pancreas |
| Cingulate_gyrus | H3K4me1 | 0.36 | 1.14 | 0.571279863 | Prostate | CNS |
| Pancreatic_islets | H3K9ac | 5.99 | 8.89 | 0.572208479 | Lung | Adrenal_Pancreas |
| CD19_primary_(UW) | H3K4me1 | 2.23 | 2.13 | 0.572660058 | Ovarian | Hematopoietic |
| Peripheralblood_mononuclear_primary | H3K4me1 | 7.65 | 11.80 | 0.573190607 | Headneck | Hematopoietic |
| Stomach_smooth_muscle | H3K27ac | 2.88 | 3.34 | 0.573567329 | Headneck | GI |
| CD8_naive_primary_(UCSF-UBC) | H3K9ac | 5.85 | 8.65 | 0.575687244 | Breast | Hematopoietic |
| Kidney | H3K9ac | 3.66 | 4.80 | 0.577571488 | Lung | Kidney |
| Liver_(UCSD) | H3K4me1 | 19.99 | 36.83 | 0.578208478 | Ovarian | Liver |
| CD20 | H3K27ac | 1.51 | 0.94 | 0.579733504 | Prostate | Hematopoietic |
| Fetal_lung | H3K9ac | 6.69 | 10.71 | 0.582489048 | Ovarian | Cardiovascular |
| Penis_foreskin_keratinocyte_primary | H3K4me3 | 5.08 | 7.62 | 0.583472667 | Headneck | Other |
| Rectal_mucosa | H3K27ac | 2.10 | 2.15 | 0.587700163 | Lung | GI |
| Fetal_heart | H3K4me1 | 2.02 | 1.94 | 0.594680889 | Ovarian | Cardiovascular |
| CD25+_CD127-_Treg | H3K27ac | 1.71 | 1.38 | 0.596219369 | Colorectal | Hematopoietic |
| Inferior_temporal_lobe | H3K4me1 | 0.44 | 1.06 | 0.596468277 | Prostate | CNS |
| CD4+_CD25-_IL17+_PMA_Ionomycin_stim_Th17_primary | H3K4me3 | 5.21 | 7.92 | 0.598195331 | Ovarian | Hematopoietic |
| CD34_primary | H3K4me1 | 3.47 | 4.64 | 0.599945648 | Headneck | Hematopoietic |
| CD4+_CD25-_IL17-_PMA_Ionomycin_stim_MACS_Th_sprimary | H3K4me1 | 2.29 | 2.46 | 0.601700507 | Headneck | Hematopoietic |
| Adipose_nuclei | H3K4me1 | 1.87 | 1.69 | 0.61161663 | Ovarian | Other |
| CD19 | H3K27ac | 0.43 | 1.13 | 0.61446969 | Colorectal | Hematopoietic |
| Fetal_thymus | H3K4me1 | 2.27 | 2.53 | 0.620696642 | Ovarian | Hematopoietic |
| CD4+_CD25-_IL17+_PMA_Ionomycin_stim_Th17_primary | H3K4me1 | 2.49 | 3.00 | 0.620747808 | Headneck | Hematopoietic |
| Pancreatic_islets | H3K4me1 | 5.57 | 9.62 | 0.621618802 | Ovarian | Adrenal_Pancreas |
| Stomach_mucosa | H3K4me3 | 9.40 | 17.18 | 0.622589441 | Headneck | GI |
| Stomach_smooth_muscle | H3K4me3 | 4.60 | 7.41 | 0.623556656 | Headneck | GI |
| Fetal_brain | H3K4me1 | 0.11 | 1.82 | 0.626413759 | Ovarian | CNS |
| Fetal_small_intestine | H3K4me3 | 6.52 | 11.52 | 0.627092834 | Headneck | GI |
| CD4_memory_primary | H3K4me1 | 2.34 | 2.75 | 0.629306554 | Headneck | Hematopoietic |
| Skeletal_muscle | H3K27ac | 1.66 | 1.36 | 0.629523111 | Colorectal | SkeletalMuscle |
| Spleen | H3K4me1 | 2.18 | 2.54 | 0.630337289 | Ovarian | Hematopoietic |
| Stomach_mucosa | H3K4me1 | 0.08 | 1.97 | 0.637614398 | Lung | GI |
| Duodenum_smooth_muscle | H3K4me1 | 4.78 | 8.37 | 0.640028228 | Lung | GI |
| Colonic_mucosa | H3K4me1 | 3.29 | 4.91 | 0.642010167 | Ovarian | GI |
| Stomach_mucosa | H3K4me1 | 2.36 | 2.94 | 0.64696712 | Ovarian | GI |
| Rectal_smooth_muscle | H3K9ac | 11.93 | 24.47 | 0.651990257 | Headneck | GI |
| Rectal_mucosa | H3K4me1 | 1.78 | 1.75 | 0.652499078 | Lung | GI |
| Osteoblast | H3K27ac | 1.43 | 0.98 | 0.658049087 | Prostate | Connective_Bone |
| CD4_primary | H3K4me3 | 4.37 | 7.81 | 0.662702711 | Ovarian | Hematopoietic |
| Adipose_nuclei | H3K27ac | 1.92 | 2.18 | 0.667388285 | Ovarian | Other |
| Pancreatic_islets | H3K4me1 | 7.24 | 14.67 | 0.670190114 | Headneck | Adrenal_Pancreas |
| CD25-_IL17+_Th17_stim | H3K27ac | 0.25 | 1.77 | 0.671473866 | Colorectal | Hematopoietic |
| Kidney | H3K4me1 | 5.32 | 10.34 | 0.67445512 | Headneck | Kidney |
| Placenta_amnion | H3K4me3 | 6.18 | 12.64 | 0.680075159 | Ovarian | Other |
| Breast_vHMEC | H3K4me1 | 0.12 | 2.14 | 0.683587001 | Lung | Other |
| Angular_gyrus | H3K4me3 | 2.49 | 3.72 | 0.686383318 | Prostate | CNS |
| Substantia_nigra | H3K27ac | 1.53 | 1.34 | 0.689866496 | Colorectal | CNS |
| Fetal_stomach | H3K4me1 | 1.88 | 2.23 | 0.691169176 | Lung | GI |
| Fetal_heart | H3K4me3 | 5.72 | 11.78 | 0.691397534 | Ovarian | Cardiovascular |
| Right_ventricle | H3K4me1 | 11.18 | 26.58 | 0.695524348 | Lung | Cardiovascular |
| Cingulate_gyrus | H3K4me1 | 2.16 | 2.98 | 0.697815578 | Headneck | CNS |
| CD14 | H3K27ac | 0.48 | 1.34 | 0.700207445 | Colorectal | Hematopoietic |
| Right_ventricle | H3K4me3 | 4.78 | 9.92 | 0.701062108 | Ovarian | Cardiovascular |
| Fetal_large_intestine | H3K4me1 | 2.67 | 4.36 | 0.701167349 | Headneck | GI |
| CD25-_IL17-_Th_stim_MACS | H3K27ac | 0.53 | 1.25 | 0.701434009 | Breast | Hematopoietic |
| Fetal_heart | H3K4me3 | 2.77 | 4.74 | 0.706844344 | Prostate | Cardiovascular |
| Penis_foreskin_keratinocyte_primary | H3K4me1 | 1.58 | 1.54 | 0.707732632 | Lung | Other |
| Penis_foreskin_keratinocyte_primary | H3K4me1 | 2.06 | 2.86 | 0.707943971 | Headneck | Other |
| Inferior_temporal_lobe | H3K27ac | 0.48 | 1.38 | 0.708351287 | Colorectal | CNS |
| Peripheralblood_mononuclear_primary | H3K4me1 | 4.96 | 11.00 | 0.709583865 | Ovarian | Hematopoietic |
| CD4+_CD25int_CD127+_Tmem_primary | H3K4me1 | 3.31 | 6.19 | 0.709741321 | Headneck | Hematopoietic |
| Colon_smooth_muscle | H3K4me3 | 4.57 | 9.68 | 0.71080305 | Headneck | GI |
| Angular_gyrus | H3K4me1 | 3.16 | 5.84 | 0.712191746 | Ovarian | CNS |
| Fetal_brain | H3K4me1 | 2.15 | 3.19 | 0.71573498 | Headneck | CNS |
| Th0 | H3K27ac | 1.58 | 1.60 | 0.717656211 | Colorectal | Hematopoietic |
| Rectal_smooth_muscle | H3K4me3 | 4.46 | 9.65 | 0.71991185 | Headneck | GI |
| Left_Ventricle | H3K4me1 | 0.02 | 2.99 | 0.721679936 | Lung | Cardiovascular |
| Rectal_mucosa | H3K9ac | 3.56 | 7.18 | 0.72208992 | Headneck | GI |
| Cingulate_gyrus | H3K9ac | 0.10 | 2.56 | 0.723838159 | Prostate | CNS |
| Fetal_brain | H3K4me1 | 1.36 | 1.07 | 0.732050622 | Colorectal | CNS |
| Breast_fibroblast_primary | H3K4me3 | 6.99 | 18.17 | 0.733894787 | Ovarian | Connective_Bone |
| Skeletal_muscle | H3K4me1 | 1.61 | 1.76 | 0.734020692 | Ovarian | SkeletalMuscle |
| Fetal_large_intestine | H3K4me1 | 1.81 | 2.51 | 0.73437423 | Lung | GI |
| CD3_primary | H3K27ac | 0.64 | 1.07 | 0.73536883 | Breast | Hematopoietic |
| CD8_naive_primary_(UCSF-UBC) | H3K9ac | 11.88 | 32.61 | 0.73789701 | Headneck | Hematopoietic |
| Breast_vHMEC | H3K4me3 | 5.96 | 15.07 | 0.739411765 | Headneck | Other |
| Duodenum_Mucosa | H3K9ac | 3.52 | 7.59 | 0.741389184 | Headneck | GI |
| Duodenum_smooth_muscle | H3K4me1 | 5.94 | 15.30 | 0.743387214 | Headneck | GI |
| Aorta | H3K4me3 | 4.56 | 11.00 | 0.744336283 | Ovarian | Cardiovascular |
| Duodenum_mucosa | H3K27ac | 0.12 | 2.74 | 0.744712937 | Lung | GI |
| Fetal_heart | H3K9ac | 2.46 | 4.43 | 0.745022494 | Ovarian | Cardiovascular |
| Rectal_smooth_muscle | H3K4me1 | 3.06 | 6.33 | 0.746962172 | Ovarian | GI |
| Hippocampus_middle | H3K27ac | 0.47 | 1.64 | 0.747244235 | Colorectal | CNS |
| Anterior_caudate | H3K27ac | 0.57 | 1.36 | 0.74911671 | Colorectal | CNS |
| Th1 | H3K27ac | 1.26 | 0.81 | 0.749215328 | Breast | Hematopoietic |
| CD34_primary | H3K4me3 | 4.76 | 11.82 | 0.750567957 | Headneck | Hematopoietic |
| CD3_primary_(UW) | H3K4me3 | 3.70 | 8.70 | 0.751268345 | Ovarian | Hematopoietic |
| CD34_primary | H3K4me1 | 2.23 | 4.11 | 0.756211061 | Ovarian | Hematopoietic |
| Stomach_smooth_muscle | H3K4me3 | 2.96 | 6.56 | 0.760004214 | Ovarian | GI |
| Mobilized_CD34 | H3K27ac | 1.39 | 1.27 | 0.760571891 | Prostate | Hematopoietic |
| Breast_fibroblast_primary | H3K4me3 | 3.04 | 6.77 | 0.76151353 | Colorectal | Connective_Bone |
| CD25-_CD45RA+_naive | H3K27ac | 0.60 | 1.31 | 0.763453769 | Colorectal | Hematopoietic |
| CD25int_CD127+_Tmem | H3K27ac | 1.32 | 1.08 | 0.764050798 | Prostate | Hematopoietic |
| Fetal_leg_muscle | H3K4me1 | 1.54 | 1.82 | 0.764845873 | Lung | SkeletalMuscle |
| Anterior_caudate | H3K9ac | 3.03 | 6.80 | 0.76546342 | Ovarian | CNS |
| Breast_myoepithelial | H3K9ac | 7.86 | 23.19 | 0.768971219 | Headneck | Other |
| CD14 | H3K27ac | 1.28 | 0.96 | 0.769021184 | Prostate | Hematopoietic |
| Hippocampus_middle | H3K4me1 | 1.78 | 2.66 | 0.770544535 | Headneck | CNS |
| Fetal_thymus | H3K4me3 | 3.69 | 9.23 | 0.770748028 | Ovarian | Hematopoietic |
| Fetal_lung | H3K9ac | 3.85 | 9.85 | 0.771225109 | Headneck | Cardiovascular |
| CD20 | H3K27ac | 1.30 | 1.04 | 0.775260722 | Colorectal | Hematopoietic |
| Psoas_muscle | H3K4me3 | 4.30 | 11.65 | 0.776980439 | Headneck | SkeletalMuscle |
| Esophagus | H3K4me3 | 4.85 | 13.69 | 0.777786328 | Headneck | GI |
| Fetal_small_intestine | H3K4me1 | 0.36 | 2.33 | 0.779909217 | Lung | GI |
| CD15_primary | H3K4me1 | 0.26 | 2.79 | 0.787859965 | Headneck | Hematopoietic |
| Fetal_kidney | H3K9ac | 5.63 | 17.31 | 0.789349267 | Headneck | Kidney |
| Substantia_nigra | H3K9ac | 0.25 | 2.86 | 0.790747234 | Prostate | CNS |
| Breast_myoepithelial | H3K4me1 | 0.63 | 1.41 | 0.792933986 | Lung | Other |
| Mobilized_CD34_primary | H3K4me3 | 2.77 | 6.82 | 0.795851152 | Headneck | Hematopoietic |
| Angular_gyrus | H3K9ac | 1.74 | 2.87 | 0.797212987 | Breast | CNS |
| Pancreatic_islets | H3K4me3 | 2.45 | 5.72 | 0.797782732 | Lung | Adrenal_Pancreas |
| Fetal_brain | H3K4me3 | 3.65 | 10.39 | 0.798691126 | Headneck | CNS |
| Inferior_temporal_lobe | H3K4me1 | 1.62 | 2.47 | 0.800895558 | Ovarian | CNS |
| Right_atrium | H3K4me3 | 3.86 | 11.41 | 0.801651025 | Headneck | Cardiovascular |
| Peripheralblood_mononuclear_primary | H3K4me3 | 3.56 | 10.48 | 0.801962042 | Ovarian | Hematopoietic |
| Liver | H3K27ac | 0.59 | 1.69 | 0.805925185 | Lung | Liver |
| Th2 | H3K27ac | 1.21 | 0.85 | 0.808815186 | Prostate | Hematopoietic |
| Cingulate_gyrus | H3K4me3 | 1.63 | 2.69 | 0.812714416 | Prostate | CNS |
| CD25int_CD127+_Tmem | H3K27ac | 1.35 | 1.50 | 0.813457799 | Colorectal | Hematopoietic |
| Mid_frontal_lobe | H3K4me1 | 2.68 | 7.44 | 0.81666989 | Lung | CNS |
| Rectal_mucosa | H3K4me1 | 1.97 | 4.20 | 0.816729306 | Headneck | GI |
| Breast_vHMEC | H3K4me1 | 2.06 | 4.73 | 0.821075575 | Headneck | Other |
| Colon_smooth_muscle | H3K27ac | 1.79 | 3.54 | 0.823297706 | Headneck | GI |
| Hippocampus_middle | H3K4me1 | 1.23 | 1.05 | 0.825927607 | Prostate | CNS |
| Spleen | H3K4me1 | 1.72 | 3.32 | 0.826914503 | Headneck | Hematopoietic |
| Penis_foreskin_fibroblast_primary | H3K4me3 | 1.63 | 2.96 | 0.831022931 | Ovarian | Connective_Bone |
| Fetal_lung | H3K4me1 | 1.64 | 3.01 | 0.831784015 | Headneck | Cardiovascular |
| CD56_primary | H3K4me3 | 3.73 | 13.30 | 0.837518121 | Headneck | Hematopoietic |
| CD19_primary_(BI) | H3K4me1 | 1.58 | 2.89 | 0.842759503 | Headneck | Hematopoietic |
| Mid_frontal_lobe | H3K4me3 | 2.70 | 8.81 | 0.847117699 | Headneck | CNS |
| CD14 | H3K27ac | 0.58 | 2.19 | 0.849428795 | Ovarian | Hematopoietic |
| CD15_primary | H3K4me3 | 2.09 | 5.84 | 0.852098862 | Ovarian | Hematopoietic |
| Fetal_brain | H3K4me1 | 1.12 | 0.63 | 0.853192731 | Breast | CNS |
| Fetal_small_intestine | H3K4me1 | 1.73 | 4.05 | 0.856721556 | Headneck | GI |
| Stomach_smooth_muscle | H3K27ac | 0.61 | 2.16 | 0.856861869 | Ovarian | GI |
| Colonic_mucosa | H3K4me1 | 0.53 | 2.63 | 0.856909053 | Lung | GI |
| Adipose_nuclei | H3K27ac | 0.71 | 1.72 | 0.858478003 | Lung | Other |
| Colonic_mucosa | H3K4me3 | 2.83 | 10.43 | 0.860341698 | Headneck | GI |
| Pancreatic_islets | H3K4me1 | 0.31 | 4.03 | 0.863670603 | Lung | Adrenal_Pancreas |
| Breast_fibroblast_primary | H3K4me1 | 2.01 | 6.03 | 0.866595013 | Ovarian | Connective_Bone |
| Breast_myoepithelial | H3K4me1 | 1.50 | 3.02 | 0.868562325 | Headneck | Other |
| Substantia_nigra | H3K4me1 | 0.82 | 1.15 | 0.872028711 | Prostate | CNS |
| CD8_memory_primary | H3K4me3 | 2.58 | 9.87 | 0.87258025 | Headneck | Hematopoietic |
| Substantia_nigra | H3K4me3 | 2.16 | 7.55 | 0.877663768 | Ovarian | CNS |
| Hippocampus_middle | H3K4me3 | 0.05 | 6.39 | 0.880148176 | Ovarian | CNS |
| CD20 | H3K27ac | 0.67 | 2.26 | 0.881454485 | Ovarian | Hematopoietic |
| Skeletal_muscle | H3K9ac | 1.55 | 3.98 | 0.887275431 | Ovarian | SkeletalMuscle |
| CD8_naive_primary_(BI) | H3K4me3 | 2.41 | 10.28 | 0.890065582 | Headneck | Hematopoietic |
| Substantia_nigra | H3K4me1 | 1.36 | 2.61 | 0.89077211 | Ovarian | CNS |
| Pancreatic_islets | H3K9ac | 2.85 | 13.48 | 0.891055084 | Headneck | Adrenal_Pancreas |
| Fetal_thymus | H3K4me3 | 2.60 | 11.85 | 0.892842554 | Headneck | Hematopoietic |
| Spleen | H3K4me1 | 1.29 | 2.44 | 0.898318965 | Lung | Hematopoietic |
| Duodenum_Mucosa | H3K4me1 | 1.44 | 3.48 | 0.899924575 | Headneck | GI |
| Neurosphere | H3K27ac | 1.42 | 3.42 | 0.902841093 | Headneck | CNS |
| Penis_foreskin_melanocyte_primary | H3K4me1 | 0.72 | 2.32 | 0.903611228 | Headneck | Other |
| Kidney | H3K4me3 | 1.99 | 8.35 | 0.905511424 | Headneck | Kidney |
| Penis_foreskin_keratinocyte_primary | H3K4me3 | 0.38 | 5.76 | 0.913016277 | Ovarian | Other |
| Inferior_temporal_lobe | H3K9ac | 1.66 | 6.16 | 0.913480056 | Ovarian | CNS |
| Duodenum_Mucosa | H3K4me3 | 1.74 | 6.83 | 0.913916435 | Headneck | GI |
| Inferior_temporal_lobe | H3K4me3 | 1.29 | 2.73 | 0.915097773 | Prostate | CNS |
| Peripheralblood_mononuclear_primary | H3K4me1 | 1.49 | 4.65 | 0.915784103 | Colorectal | Hematopoietic |
| CD8_primary | H3K4me3 | 2.18 | 11.23 | 0.916471911 | Headneck | Hematopoietic |
| Thymus | H3K4me1 | 0.72 | 2.68 | 0.917634099 | Breast | Hematopoietic |
| Psoas_muscle | H3K4me1 | 0.35 | 6.44 | 0.91915243 | Colorectal | SkeletalMuscle |
| Penis_foreskin_fibroblast_primary | H3K4me1 | 0.90 | 0.98 | 0.919289827 | Lung | Connective_Bone |
| CD3_primary_(BI) | H3K4me3 | 1.86 | 8.84 | 0.922519136 | Headneck | Hematopoietic |
| Ovary | H3K4me3 | 2.28 | 13.34 | 0.923651476 | Headneck | Other |
| Pancreatic_islets | H3K4me3 | 2.04 | 10.91 | 0.924014405 | Headneck | Adrenal_Pancreas |
| Mid_frontal_lobe | H3K4me1 | 1.75 | 8.44 | 0.928757846 | Colorectal | CNS |
| Esophagus | H3K4me1 | 1.51 | 5.76 | 0.929403341 | Lung | GI |
| Hippocampus_middle | H3K9ac | 0.76 | 2.74 | 0.930187966 | Prostate | CNS |
| Treg_primary | H3K4me3 | 1.51 | 5.88 | 0.930619811 | Headneck | Hematopoietic |
| CD19_primary_(UW) | H3K4me1 | 0.78 | 2.91 | 0.938518577 | Headneck | Hematopoietic |
| Anterior_caudate | H3K4me3 | 1.20 | 2.74 | 0.942096133 | Prostate | CNS |
| Th2 | H3K27ac | 1.06 | 0.91 | 0.942483594 | Breast | Hematopoietic |
| Fetal_heart | H3K4me3 | 2.02 | 14.90 | 0.945595409 | Headneck | Cardiovascular |
| CD8_naive_primary_(UCSF-UBC) | H3K4me3 | 2.11 | 16.37 | 0.945854971 | Headneck | Hematopoietic |
| Fetal_lung | H3K4me1 | 0.90 | 1.47 | 0.946846338 | Lung | Cardiovascular |
| CD14 | H3K27ac | 0.93 | 1.06 | 0.948649436 | Breast | Hematopoietic |
| Adipose_nuclei | H3K4me1 | 1.12 | 2.12 | 0.953193744 | Headneck | Other |
| CD25-_IL17-_Th_stim_MACS | H3K27ac | 0.93 | 1.27 | 0.953551938 | Colorectal | Hematopoietic |
| Fetal_lung | H3K9ac | 0.70 | 5.91 | 0.95719305 | Lung | Cardiovascular |
| Substantia_nigra | H3K9ac | 1.32 | 6.09 | 0.957501455 | Ovarian | CNS |
| Fetal_brain | H3K4me1 | 0.96 | 0.78 | 0.959971793 | Prostate | CNS |
| CD4+_CD25-_CD45RA+_naive_primary | H3K4me3 | 1.47 | 9.33 | 0.960005314 | Headneck | Hematopoietic |
| Placenta_chorion | H3K4me3 | 1.41 | 8.69 | 0.961214632 | Ovarian | Other |
| Anterior_caudate | H3K4me1 | 0.86 | 2.89 | 0.961849853 | Ovarian | CNS |
| CD19_primary_(BI) | H3K4me3 | 0.55 | 9.52 | 0.962042925 | Headneck | Hematopoietic |
| Cingulate_gyrus | H3K4me1 | 0.87 | 2.74 | 0.96218343 | Ovarian | CNS |
| Cingulate_gyrus | H3K27ac | 1.06 | 1.28 | 0.963193276 | Colorectal | CNS |
| Stomach_smooth_muscle | H3K27ac | 1.04 | 0.95 | 0.963845618 | Prostate | GI |
| Penis_foreskin_melanocyte_primary | H3K4me3 | 0.66 | 7.54 | 0.963889137 | Headneck | Other |
| Fetal_trunk_muscle | H3K4me1 | 0.89 | 2.54 | 0.964697654 | Ovarian | SkeletalMuscle |
| Ovary | H3K4me1 | 1.12 | 2.88 | 0.967813543 | Prostate | Other |
| Colonic_mucosa | H3K4me1 | 0.81 | 6.11 | 0.975827997 | Headneck | GI |
| Lung | H3K4me3 | 1.56 | 18.83 | 0.97608993 | Headneck | Cardiovascular |
| Gastric | H3K4me1 | 0.88 | 4.14 | 0.976146093 | Lung | GI |
| Anterior_caudate | H3K4me3 | 1.18 | 6.12 | 0.976884225 | Ovarian | CNS |
| CD14_primary | H3K4me3 | 0.71 | 10.84 | 0.97776546 | Ovarian | Hematopoietic |
| Peripheralblood_mononuclear_primary | H3K4me3 | 1.30 | 12.82 | 0.981061069 | Headneck | Hematopoietic |
| CD56_primary | H3K4me3 | 1.26 | 12.22 | 0.981397264 | Ovarian | Hematopoietic |
| Hippocampus_middle | H3K4me1 | 1.05 | 2.39 | 0.981799263 | Ovarian | CNS |
| CD25-_IL17+_Th17_stim | H3K27ac | 1.09 | 4.19 | 0.98303859 | Headneck | Hematopoietic |
| CD3_primary_(UW) | H3K4me3 | 1.21 | 11.19 | 0.984771098 | Headneck | Hematopoietic |
| CD8_primary | H3K4me3 | 1.17 | 9.29 | 0.984913895 | Ovarian | Hematopoietic |
| Gastric | H3K4me3 | 0.67 | 17.98 | 0.985273032 | Headneck | GI |
| Stomach_mucosa | H3K9ac | 0.83 | 9.36 | 0.985477045 | Headneck | GI |
| Duodenum_smooth_muscle | H3K4me1 | 1.15 | 13.06 | 0.990322245 | Ovarian | GI |
| CD15_primary | H3K4me3 | 1.08 | 6.69 | 0.990603521 | Headneck | Hematopoietic |
| Colonic_mucosa | H3K9ac | 0.95 | 6.49 | 0.993856759 | Headneck | GI |
| Fetal_brain | H3K9ac | 0.95 | 10.43 | 0.995930782 | Headneck | CNS |
| CD19_primary_(BI) | H3K4me3 | 1.03 | 7.84 | 0.997268532 | Ovarian | Hematopoietic |
| CD19 | H3K27ac | 1.01 | 2.22 | 0.998141443 | Ovarian | Hematopoietic |
| CD4+_CD25+_CD127-_Treg_primary | H3K4me3 | 1.02 | 8.36 | 0.998325958 | Headneck | Hematopoietic |
| Bold font: P < 0.05/220; GI: gastrointestinal; CNS: central nervous system. When the same cell type in the same histone mark from more than one institution was used, the name of institution is given in parentheses. | | | | | | |
