# Supplementary material for: Shared heritability and functional enrichment across six solid cancers
Source: Nat Commun. 2019 Jan 25;10:431. doi: 10.1038/s41467-018-08054-4 (PMC6347624; doi:10.1038/s41467-018-08054-4)
Supplement: Supplementary file 7 — Supplementary Data 5 [file 41467_2018_8054_MOESM7_ESM.docx]

**Shared heritability and functional enrichment across six solid cancers**

**Jiang et al.**

| **Supplementary Data 5. Enrichment estimates of the 220 cell-type-specific annotations over four histone marks H3K4me1, H3K4me3, H3K9ac, and H3K27ac, for subtypes of cancers.** | | | | | | |
| --- | --- | --- | --- | --- | --- | --- |
| **Cell type** | **Mark** | **Enrichment** | **Enrichment standard error** | **Enrichment p-values** | **Cancer subtype** | **Category** |
| Rectal_mucosa | H3K4me1 | 10.69 | 2.38 | **1.90E-05** | Advanced stage prostate cancer | GI |
| Colonic_mucosa | H3K4me1 | 15.85 | 3.82 | **4.18E-05** | Advanced stage prostate cancer | GI |
| Colonic_mucosa | H3K9ac | 16.19 | 4.05 | **5.01E-05** | Advanced stage prostate cancer | GI |
| Stomach_mucosa | H3K4me1 | 10.86 | 2.56 | **8.71E-05** | Advanced stage prostate cancer | GI |
| Rectal_mucosa | H3K27ac | 10.60 | 2.54 | **9.81E-05** | Advanced stage prostate cancer | GI |
| Rectal_mucosa | H3K4me3 | 15.32 | 4.02 | **0.000129836** | Advanced stage prostate cancer | GI |
| Duodenum_Mucosa | H3K4me1 | 8.41 | 2.09 | **0.000198614** | Advanced stage prostate cancer | GI |
| Colonic_mucosa | H3K27ac | 11.02 | 2.81 | **0.000219453** | Advanced stage prostate cancer | GI |
| CD4+_CD25-_IL17-_PMA_Ionomycin_stim_MACS_Th_sprimary | H3K4me1 | 5.12 | 1.20 | 0.000230808 | Advanced stage prostate cancer | Hematopoietic |
| CD4+_CD25-_Th_primary | H3K4me1 | 5.81 | 1.43 | 0.000328147 | Advanced stage prostate cancer | Hematopoietic |
| Mobilized_CD34_primary | H3K4me1 | 5.16 | 1.22 | 0.000342929 | Advanced stage prostate cancer | Hematopoietic |
| Penis_foreskin_fibroblast_primary | H3K4me1 | 4.40 | 0.99 | 0.00057764 | Advanced stage prostate cancer | Connective_Bone |
| CD4+_CD25-_IL17+_PMA_Ionomycin_stim_Th17_primary | H3K4me1 | 5.47 | 1.40 | 0.000589576 | Advanced stage prostate cancer | Hematopoietic |
| Fetal_small_intestine | H3K4me1 | 9.15 | 2.47 | 0.000654129 | Advanced stage prostate cancer | GI |
| Anterior_caudate | H3K27ac | -4.11 | 1.53 | 0.000671081 | Advanced stage prostate cancer | CNS |
| Penis_foreskin_keratinocyte_primary | H3K4me1 | 7.35 | 1.91 | 0.000743836 | Advanced stage prostate cancer | Other |
| Inferior_temporal_lobe | H3K27ac | -3.20 | 1.29 | 0.000834645 | Advanced stage prostate cancer | CNS |
| CD4+_CD25-_CD45RA+_naive_primary | H3K4me1 | 6.43 | 1.71 | 0.000840886 | Advanced stage prostate cancer | Hematopoietic |
| CD3_primary_(BI) | H3K4me1 | 7.86 | 2.23 | 0.000885105 | Advanced stage prostate cancer | Hematopoietic |
| CD4+_CD25+_CD127-_Treg_primary | H3K4me1 | 6.58 | 1.79 | 0.001020868 | Advanced stage prostate cancer | Hematopoietic |
| CD3_primary_(UW) | H3K4me1 | 6.37 | 1.72 | 0.001067768 | Advanced stage prostate cancer | Hematopoietic |
| Pancreas | H3K4me1 | 9.93 | 2.78 | 0.001068056 | Advanced stage prostate cancer | Adrenal_Pancreas |
| Breast_myoepithelial | H3K4me1 | 5.53 | 1.47 | 0.001669724 | Advanced stage prostate cancer | Other |
| Rectal_mucosa | H3K9ac | 12.51 | 3.90 | 0.001680687 | Advanced stage prostate cancer | GI |
| CD4_naive_primary | H3K4me1 | 6.46 | 1.86 | 0.001868629 | Advanced stage prostate cancer | Hematopoietic |
| Stomach_mucosa | H3K9ac | 16.23 | 5.18 | 0.001932926 | Advanced stage prostate cancer | GI |
| CD15_primary | H3K4me1 | 5.55 | 1.59 | 0.002332856 | Advanced stage prostate cancer | Hematopoietic |
| Penis_foreskin_keratinocyte_primary | H3K9ac | 6.98 | 2.02 | 0.002344578 | Advanced stage prostate cancer | Other |
| Fetal_large_intestine | H3K4me1 | 8.36 | 2.50 | 0.002507238 | Advanced stage prostate cancer | GI |
| CD8_memory_primary | H3K4me1 | 6.22 | 1.83 | 0.002667892 | Advanced stage prostate cancer | Hematopoietic |
| Fetal_lung | H3K4me1 | 5.16 | 1.41 | 0.002872154 | Advanced stage prostate cancer | Cardiovascular |
| CD4+_CD25-_CD45R0+_memory_primary | H3K4me1 | 5.23 | 1.51 | 0.002977461 | Advanced stage prostate cancer | Hematopoietic |
| Breast_vHMEC | H3K4me1 | 8.49 | 2.56 | 0.003382332 | Advanced stage prostate cancer | Other |
| CD4_memory_primary | H3K4me1 | 4.74 | 1.35 | 0.003445466 | Advanced stage prostate cancer | Hematopoietic |
| Penis_foreskin_melanocyte_primary | H3K4me1 | 4.60 | 1.29 | 0.003684998 | Advanced stage prostate cancer | Other |
| Fetal_thymus | H3K4me1 | 5.77 | 1.73 | 0.003735878 | Advanced stage prostate cancer | Hematopoietic |
| Rectal_smooth_muscle | H3K4me3 | 14.09 | 4.79 | 0.004134099 | Advanced stage prostate cancer | GI |
| CD8_naive_primary_(UCSF-UBC) | H3K4me1 | 6.54 | 2.05 | 0.004531185 | Advanced stage prostate cancer | Hematopoietic |
| CD8_naive_primary_(BI) | H3K4me1 | 6.44 | 2.05 | 0.005095008 | Advanced stage prostate cancer | Hematopoietic |
| CD19_primary_(UW) | H3K4me1 | 4.53 | 1.37 | 0.005634359 | Advanced stage prostate cancer | Hematopoietic |
| Gastric | H3K4me1 | 15.93 | 5.31 | 0.00565538 | Advanced stage prostate cancer | GI |
| Adipose_nuclei | H3K4me1 | 3.77 | 1.01 | 0.006162715 | Advanced stage prostate cancer | Other |
| Breast_myoepithelial | H3K4me3 | 13.53 | 4.82 | 0.006273618 | Advanced stage prostate cancer | Other |
| Kidney | H3K9ac | 12.96 | 4.72 | 0.006502702 | Advanced stage prostate cancer | Kidney |
| Colonic_mucosa | H3K4me3 | 14.42 | 5.27 | 0.007157544 | Advanced stage prostate cancer | GI |
| Liver_(BI) | H3K4me1 | 4.21 | 1.20 | 0.007444403 | Advanced stage prostate cancer | Liver |
| Angular_gyrus | H3K27ac | -2.73 | 1.40 | 0.007489684 | Advanced stage prostate cancer | CNS |
| Breast_luminal_epithelial | H3K4me1 | 65.78 | 24.35 | 0.008749056 | Advanced stage prostate cancer | Other |
| Duodenum_Mucosa | H3K9ac | 13.85 | 5.16 | 0.009131638 | Advanced stage prostate cancer | GI |
| Fetal_leg_muscle | H3K4me1 | 5.91 | 1.84 | 0.009174269 | Advanced stage prostate cancer | SkeletalMuscle |
| CD19_primary_(BI) | H3K4me1 | 4.71 | 1.56 | 0.010684894 | Advanced stage prostate cancer | Hematopoietic |
| Stomach_mucosa | H3K4me3 | 24.31 | 9.60 | 0.010702848 | Advanced stage prostate cancer | GI |
| Duodenum_Mucosa | H3K4me3 | 9.89 | 3.65 | 0.010739517 | Advanced stage prostate cancer | GI |
| Penis_foreskin_fibroblast_primary | H3K4me3 | 5.24 | 1.75 | 0.011366049 | Advanced stage prostate cancer | Connective_Bone |
| CD4+_CD25int_CD127+_Tmem_primary | H3K4me1 | 8.37 | 3.00 | 0.011662152 | Advanced stage prostate cancer | Hematopoietic |
| Fetal_lung | H3K9ac | 13.78 | 5.26 | 0.011837852 | Advanced stage prostate cancer | Cardiovascular |
| Penis_foreskin_keratinocyte_primary | H3K4me3 | 11.55 | 4.38 | 0.012146919 | Advanced stage prostate cancer | Other |
| Kidney | H3K4me1 | 13.43 | 5.10 | 0.012373874 | Advanced stage prostate cancer | Kidney |
| CD56_primary | H3K4me1 | 4.73 | 1.57 | 0.012404905 | Advanced stage prostate cancer | Hematopoietic |
| Mid_frontal_lobe | H3K27ac | -3.12 | 1.78 | 0.013480517 | Advanced stage prostate cancer | CNS |
| Duodenum_mucosa | H3K27ac | 7.54 | 2.65 | 0.015512963 | Advanced stage prostate cancer | GI |
| Adipose_nuclei | H3K9ac | 6.54 | 2.38 | 0.015515084 | Advanced stage prostate cancer | Other |
| Fetal_stomach | H3K4me3 | 14.44 | 5.95 | 0.015994776 | Advanced stage prostate cancer | GI |
| Cingulate_gyrus | H3K27ac | -1.93 | 1.24 | 0.017280173 | Advanced stage prostate cancer | CNS |
| Hippocampus_middle | H3K27ac | -2.66 | 1.57 | 0.018376008 | Advanced stage prostate cancer | CNS |
| Kidney | H3K4me3 | 9.46 | 3.72 | 0.019504589 | Advanced stage prostate cancer | Kidney |
| CD25-_IL17-_Th_stim_MACS | H3K27ac | 4.99 | 1.77 | 0.021785954 | Advanced stage prostate cancer | Hematopoietic |
| Small_intestine | H3K4me3 | 22.70 | 10.24 | 0.022046268 | Advanced stage prostate cancer | GI |
| CD25-_CD45RA+_naive | H3K27ac | 5.43 | 1.99 | 0.0223579 | Advanced stage prostate cancer | Hematopoietic |
| Fetal_small_intestine | H3K4me3 | 13.84 | 6.01 | 0.022424796 | Advanced stage prostate cancer | GI |
| CD34_primary | H3K4me1 | 6.37 | 2.46 | 0.02281774 | Advanced stage prostate cancer | Hematopoietic |
| Breast_vHMEC | H3K4me3 | 16.48 | 7.32 | 0.024659622 | Advanced stage prostate cancer | Other |
| Colon_smooth_muscle | H3K4me1 | 4.91 | 1.80 | 0.025295499 | Advanced stage prostate cancer | GI |
| Liver_(UCSD) | H3K4me3 | 12.74 | 5.41 | 0.025446012 | Advanced stage prostate cancer | Liver |
| Fetal_large_intestine | H3K4me3 | 13.34 | 5.89 | 0.026444093 | Advanced stage prostate cancer | GI |
| Esophagus | H3K4me3 | 16.23 | 7.09 | 0.030735993 | Advanced stage prostate cancer | GI |
| Fetal_adrenal | H3K4me1 | 4.76 | 1.81 | 0.032971683 | Advanced stage prostate cancer | Adrenal_Pancreas |
| Spleen | H3K4me1 | 4.45 | 1.64 | 0.033272479 | Advanced stage prostate cancer | Hematopoietic |
| CD34_primary | H3K4me3 | 13.45 | 6.21 | 0.033478425 | Advanced stage prostate cancer | Hematopoietic |
| Sigmoid_colon | H3K4me1 | 29.43 | 13.37 | 0.034286813 | Advanced stage prostate cancer | GI |
| Fetal_trunk_muscle | H3K4me1 | 5.10 | 1.89 | 0.036054674 | Advanced stage prostate cancer | SkeletalMuscle |
| CD4+_CD25-_CD45RA+_naive_primary | H3K4me3 | 11.22 | 5.03 | 0.036295154 | Advanced stage prostate cancer | Hematopoietic |
| Fetal_adrenal | H3K4me3 | 12.37 | 5.70 | 0.037606162 | Advanced stage prostate cancer | Adrenal_Pancreas |
| Treg_primary | H3K4me3 | 6.19 | 2.59 | 0.038396201 | Advanced stage prostate cancer | Hematopoietic |
| Colon_smooth_muscle | H3K4me3 | 8.35 | 3.75 | 0.04246756 | Advanced stage prostate cancer | GI |
| CD3_primary_(BI) | H3K4me3 | 10.00 | 4.66 | 0.043307917 | Advanced stage prostate cancer | Hematopoietic |
| Placenta_chorion | H3K4me3 | 12.88 | 6.22 | 0.046194885 | Advanced stage prostate cancer | Other |
| Sigmoid_colon | H3K4me3 | 19.17 | 9.65 | 0.047001925 | Advanced stage prostate cancer | GI |
| CD15_primary | H3K4me3 | 8.82 | 4.11 | 0.05082382 | Advanced stage prostate cancer | Hematopoietic |
| CD4+_CD25-_IL17-_PMA_Ionomycin_stim_MACS_Th_sprimary | H3K4me3 | 6.40 | 2.93 | 0.051680484 | Advanced stage prostate cancer | Hematopoietic |
| Aorta | H3K4me3 | 14.01 | 6.94 | 0.055291609 | Advanced stage prostate cancer | Cardiovascular |
| Mobilized_CD34_primary | H3K4me3 | 7.48 | 3.57 | 0.059929503 | Advanced stage prostate cancer | Hematopoietic |
| CD19_primary_(BI) | H3K4me3 | 11.13 | 5.61 | 0.060264811 | Advanced stage prostate cancer | Hematopoietic |
| Liver_(BI) | H3K9ac | 7.96 | 3.78 | 0.060560142 | Advanced stage prostate cancer | Liver |
| Pancreatic_islets | H3K27ac | 4.67 | 1.99 | 0.062212896 | Advanced stage prostate cancer | Adrenal_Pancreas |
| Psoas_muscle | H3K4me3 | 12.40 | 6.37 | 0.062514783 | Advanced stage prostate cancer | SkeletalMuscle |
| Gastric | H3K4me3 | 18.52 | 9.91 | 0.063522231 | Advanced stage prostate cancer | GI |
| Fetal_thymus | H3K4me3 | 12.19 | 6.47 | 0.069923128 | Advanced stage prostate cancer | Hematopoietic |
| Pancreatic_islets | H3K4me3 | 11.73 | 6.18 | 0.077115093 | Advanced stage prostate cancer | Adrenal_Pancreas |
| Th2 | H3K27ac | 3.79 | 1.60 | 0.077864676 | Advanced stage prostate cancer | Hematopoietic |
| Peripheralblood_mononuclear_primary | H3K9ac | 8.54 | 4.49 | 0.078017886 | Advanced stage prostate cancer | Hematopoietic |
| Penis_foreskin_melanocyte_primary | H3K4me3 | 6.91 | 3.47 | 0.078463995 | Advanced stage prostate cancer | Other |
| Stomach_smooth_muscle | H3K4me3 | 6.18 | 3.04 | 0.078480801 | Advanced stage prostate cancer | GI |
| Liver_(BI) | H3K4me3 | 5.33 | 2.52 | 0.080329022 | Advanced stage prostate cancer | Liver |
| Thymus | H3K4me1 | 9.64 | 5.04 | 0.082094771 | Advanced stage prostate cancer | Hematopoietic |
| Substantia_nigra | H3K27ac | -1.06 | 1.19 | 0.083932983 | Advanced stage prostate cancer | CNS |
| CD4_naive_primary | H3K4me3 | 8.08 | 4.30 | 0.087352203 | Advanced stage prostate cancer | Hematopoietic |
| Fetal_brain | H3K4me3 | 8.05 | 4.34 | 0.087875395 | Advanced stage prostate cancer | CNS |
| Kidney | H3K27ac | 3.95 | 1.72 | 0.087998953 | Advanced stage prostate cancer | Kidney |
| Left_Ventricle | H3K4me1 | 4.26 | 1.95 | 0.090069581 | Advanced stage prostate cancer | Cardiovascular |
| CD4+_CD25+_CD127-_Treg_primary | H3K4me3 | 6.93 | 3.66 | 0.091671303 | Advanced stage prostate cancer | Hematopoietic |
| Psoas_muscle | H3K4me1 | 13.05 | 7.46 | 0.091936501 | Advanced stage prostate cancer | SkeletalMuscle |
| Pancreatic_islets | H3K4me1 | 12.87 | 7.45 | 0.097058117 | Advanced stage prostate cancer | Adrenal_Pancreas |
| Right_atrium | H3K4me1 | 6.95 | 3.71 | 0.097337957 | Advanced stage prostate cancer | Cardiovascular |
| Stomach_smooth_muscle | H3K4me1 | 4.25 | 2.01 | 0.097924576 | Advanced stage prostate cancer | GI |
| Lung | H3K4me3 | 19.62 | 11.63 | 0.101772043 | Advanced stage prostate cancer | Cardiovascular |
| Skeletal_muscle | H3K4me1 | 2.75 | 1.08 | 0.103645172 | Advanced stage prostate cancer | SkeletalMuscle |
| CD14_primary | H3K4me1 | 3.45 | 1.55 | 0.104064298 | Advanced stage prostate cancer | Hematopoietic |
| Fetal_lung | H3K4me3 | 9.10 | 5.19 | 0.10779222 | Advanced stage prostate cancer | Cardiovascular |
| Pancreas | H3K4me3 | 12.93 | 7.74 | 0.107948375 | Advanced stage prostate cancer | Adrenal_Pancreas |
| Peripheralblood_mononuclear_primary | H3K4me1 | 9.66 | 5.64 | 0.11046526 | Advanced stage prostate cancer | Hematopoietic |
| Pancreatic_islets | H3K4me3 | 8.55 | 5.05 | 0.111726079 | Advanced stage prostate cancer | Adrenal_Pancreas |
| Fetal_kidney | H3K9ac | 14.41 | 8.76 | 0.112174826 | Advanced stage prostate cancer | Kidney |
| Left_Ventricle | H3K4me3 | 10.68 | 6.39 | 0.114562441 | Advanced stage prostate cancer | Cardiovascular |
| Duodenum_smooth_muscle | H3K4me3 | 5.05 | 2.67 | 0.115492197 | Advanced stage prostate cancer | GI |
| Liver | H3K27ac | 3.71 | 1.70 | 0.115779259 | Advanced stage prostate cancer | Liver |
| Right_atrium | H3K4me3 | 8.65 | 5.13 | 0.119397503 | Advanced stage prostate cancer | Cardiovascular |
| CD19_primary_(UW) | H3K4me3 | 11.96 | 7.37 | 0.121848167 | Advanced stage prostate cancer | Hematopoietic |
| Stomach_smooth_muscle | H3K9ac | 6.30 | 3.52 | 0.122251765 | Advanced stage prostate cancer | GI |
| Fetal_leg_muscle | H3K4me3 | 8.71 | 5.21 | 0.12758829 | Advanced stage prostate cancer | SkeletalMuscle |
| Neurosphere | H3K27ac | 5.07 | 2.64 | 0.129057345 | Advanced stage prostate cancer | CNS |
| Placenta_chorion | H3K4me1 | 11.73 | 7.35 | 0.141075033 | Advanced stage prostate cancer | Other |
| Fetal_stomach | H3K4me1 | 4.35 | 2.29 | 0.141721925 | Advanced stage prostate cancer | GI |
| Fetal_heart | H3K9ac | 4.34 | 2.39 | 0.147291296 | Advanced stage prostate cancer | Cardiovascular |
| Skeletal_muscle | H3K9ac | 4.14 | 2.25 | 0.148676918 | Advanced stage prostate cancer | SkeletalMuscle |
| CD25+_CD127-_Treg | H3K27ac | 3.19 | 1.52 | 0.149094376 | Advanced stage prostate cancer | Hematopoietic |
| Chondrogenic_dif | H3K27ac | 3.56 | 1.77 | 0.153326154 | Advanced stage prostate cancer | Connective_Bone |
| Right_ventricle | H3K4me3 | 8.75 | 5.66 | 0.153976177 | Advanced stage prostate cancer | Cardiovascular |
| Placenta_amnion | H3K4me3 | 11.86 | 7.81 | 0.154315359 | Advanced stage prostate cancer | Other |
| Lung | H3K4me1 | 4.86 | 2.79 | 0.155051586 | Advanced stage prostate cancer | Cardiovascular |
| CD8_primary | H3K4me3 | 7.68 | 4.89 | 0.15578462 | Advanced stage prostate cancer | Hematopoietic |
| CD4+_CD25-_IL17+_PMA_Ionomycin_stim_Th17_primary | H3K4me3 | 6.36 | 4.00 | 0.16233229 | Advanced stage prostate cancer | Hematopoietic |
| CD4+_CD25-_Th_primary | H3K4me3 | 7.65 | 4.95 | 0.163021462 | Advanced stage prostate cancer | Hematopoietic |
| Colon_smooth_muscle | H3K9ac | 9.37 | 6.19 | 0.16579436 | Advanced stage prostate cancer | GI |
| CD14_primary | H3K4me3 | 10.42 | 7.13 | 0.172969053 | Advanced stage prostate cancer | Hematopoietic |
| Adipose_nuclei | H3K4me3 | 3.69 | 2.04 | 0.173926193 | Advanced stage prostate cancer | Other |
| CD8_naive_primary_(BI) | H3K4me3 | 7.57 | 5.13 | 0.183170648 | Advanced stage prostate cancer | Hematopoietic |
| CD8_memory_primary | H3K4me3 | 7.00 | 4.67 | 0.184443321 | Advanced stage prostate cancer | Hematopoietic |
| Peripheralblood_mononuclear_primary | H3K4me3 | 8.37 | 5.82 | 0.191235079 | Advanced stage prostate cancer | Hematopoietic |
| Placenta_amnion | H3K4me1 | 16.31 | 11.98 | 0.200990565 | Advanced stage prostate cancer | Other |
| Fetal_placenta | H3K4me3 | 9.02 | 6.41 | 0.203301624 | Advanced stage prostate cancer | Other |
| Colon_smooth_muscle | H3K27ac | 2.98 | 1.63 | 0.226445288 | Advanced stage prostate cancer | GI |
| CD8_naive_primary_(UCSF-UBC) | H3K4me3 | 9.02 | 6.89 | 0.230940953 | Advanced stage prostate cancer | Hematopoietic |
| Fetal_brain | H3K4me3 | 12.04 | 9.36 | 0.231786291 | Advanced stage prostate cancer | CNS |
| CD20 | H3K27ac | 2.37 | 1.18 | 0.233353584 | Advanced stage prostate cancer | Hematopoietic |
| Skeletal_muscle | H3K27ac | -0.74 | 1.49 | 0.233524551 | Advanced stage prostate cancer | SkeletalMuscle |
| Duodenum_smooth_muscle | H3K27ac | 3.60 | 2.20 | 0.240591176 | Advanced stage prostate cancer | GI |
| CD56_primary | H3K4me3 | 8.65 | 6.80 | 0.243656814 | Advanced stage prostate cancer | Hematopoietic |
| Th0 | H3K27ac | 2.77 | 1.51 | 0.244981908 | Advanced stage prostate cancer | Hematopoietic |
| CD4_primary | H3K4me3 | 5.81 | 4.27 | 0.245020977 | Advanced stage prostate cancer | Hematopoietic |
| Pancreatic_islets | H3K9ac | 10.19 | 8.28 | 0.249051519 | Advanced stage prostate cancer | Adrenal_Pancreas |
| Fetal_trunk_muscle | H3K4me3 | 7.27 | 5.72 | 0.264105684 | Advanced stage prostate cancer | SkeletalMuscle |
| Germinal_matrix | H3K4me3 | 5.59 | 4.29 | 0.271869028 | Advanced stage prostate cancer | CNS |
| Rectal_smooth_muscle | H3K9ac | 12.74 | 11.10 | 0.279493375 | Advanced stage prostate cancer | GI |
| Stomach_smooth_muscle | H3K27ac | 2.57 | 1.44 | 0.280035182 | Advanced stage prostate cancer | GI |
| Ovary | H3K4me3 | 6.31 | 5.22 | 0.298980415 | Advanced stage prostate cancer | Other |
| Hippocampus_middle | H3K4me1 | 2.34 | 1.31 | 0.299851447 | Advanced stage prostate cancer | CNS |
| Fetal_heart | H3K4me1 | 2.25 | 1.23 | 0.302103327 | Advanced stage prostate cancer | Cardiovascular |
| CD4+_CD25int_CD127+_Tmem_primary | H3K4me3 | 5.77 | 4.77 | 0.303888058 | Advanced stage prostate cancer | Hematopoietic |
| CD3_primary_(UW) | H3K4me3 | 5.73 | 4.83 | 0.311654609 | Advanced stage prostate cancer | Hematopoietic |
| CD25-_IL17+_Th17_stim | H3K27ac | 2.68 | 1.72 | 0.317213952 | Advanced stage prostate cancer | Hematopoietic |
| Pancreatic_islets | H3K4me1 | 8.63 | 8.00 | 0.338835792 | Advanced stage prostate cancer | Adrenal_Pancreas |
| Esophagus | H3K4me1 | 7.87 | 7.21 | 0.339266757 | Advanced stage prostate cancer | GI |
| Rectal_smooth_muscle | H3K27ac | 2.45 | 1.50 | 0.339321815 | Advanced stage prostate cancer | GI |
| Fetal_heart | H3K4me3 | 7.52 | 7.16 | 0.354542739 | Advanced stage prostate cancer | Cardiovascular |
| Adipose_nuclei | H3K27ac | 2.54 | 1.68 | 0.358674883 | Advanced stage prostate cancer | Other |
| CD8_naive_primary_(UCSF-UBC) | H3K9ac | 12.32 | 12.72 | 0.365253303 | Advanced stage prostate cancer | Hematopoietic |
| CD25int_CD127+_Tmem | H3K27ac | 2.45 | 1.68 | 0.379066562 | Advanced stage prostate cancer | Hematopoietic |
| Breast_fibroblast_primary | H3K4me1 | -2.13 | 3.65 | 0.390857701 | Advanced stage prostate cancer | Connective_Bone |
| CD4_memory_primary | H3K4me3 | 4.36 | 4.02 | 0.391806288 | Advanced stage prostate cancer | Hematopoietic |
| CD4+_CD25-_CD45R0+_memory_primary | H3K4me3 | 5.37 | 5.36 | 0.40600543 | Advanced stage prostate cancer | Hematopoietic |
| Th1 | H3K27ac | 2.22 | 1.47 | 0.407052975 | Advanced stage prostate cancer | Hematopoietic |
| Fetal_brain | H3K9ac | 4.15 | 3.91 | 0.413259736 | Advanced stage prostate cancer | CNS |
| Fetal_brain | H3K4me1 | 0.17 | 1.05 | 0.4349596 | Advanced stage prostate cancer | CNS |
| Mid_frontal_lobe | H3K4me3 | 3.59 | 3.54 | 0.456212819 | Advanced stage prostate cancer | CNS |
| CD3_primary | H3K27ac | 2.11 | 1.52 | 0.4603311 | Advanced stage prostate cancer | Hematopoietic |
| Rectal_smooth_muscle | H3K4me1 | 4.10 | 4.26 | 0.46391212 | Advanced stage prostate cancer | GI |
| Skeletal_muscle | H3K4me3 | 2.98 | 2.86 | 0.480281781 | Advanced stage prostate cancer | SkeletalMuscle |
| CD19 | H3K27ac | 2.00 | 1.48 | 0.495736478 | Advanced stage prostate cancer | Hematopoietic |
| Breast_myoepithelial | H3K9ac | 8.03 | 10.75 | 0.50923166 | Advanced stage prostate cancer | Other |
| Angular_gyrus | H3K4me3 | 4.16 | 4.97 | 0.516116962 | Advanced stage prostate cancer | CNS |
| Cingulate_gyrus | H3K4me3 | 3.06 | 3.34 | 0.528257768 | Advanced stage prostate cancer | CNS |
| Hippocampus_middle | H3K4me3 | 2.99 | 3.25 | 0.532136818 | Advanced stage prostate cancer | CNS |
| Fetal_placenta | H3K4me1 | 2.54 | 2.51 | 0.538213634 | Advanced stage prostate cancer | Other |
| Right_ventricle | H3K4me1 | 11.78 | 18.00 | 0.546566265 | Advanced stage prostate cancer | Cardiovascular |
| Anterior_caudate | H3K4me3 | 3.06 | 3.52 | 0.548445912 | Advanced stage prostate cancer | CNS |
| Angular_gyrus | H3K4me1 | -0.82 | 3.03 | 0.553481137 | Advanced stage prostate cancer | CNS |
| Spleen | H3K4me3 | 6.52 | 9.72 | 0.561529482 | Advanced stage prostate cancer | Hematopoietic |
| Osteoblast | H3K27ac | 1.70 | 1.38 | 0.614867442 | Advanced stage prostate cancer | Connective_Bone |
| Inferior_temporal_lobe | H3K4me1 | 1.68 | 1.37 | 0.614879048 | Advanced stage prostate cancer | CNS |
| CD14 | H3K27ac | 0.41 | 1.25 | 0.634923083 | Advanced stage prostate cancer | Hematopoietic |
| Small_intestine | H3K4me1 | 21.64 | 43.91 | 0.635133709 | Advanced stage prostate cancer | GI |
| Angular_gyrus | H3K9ac | -1.02 | 4.27 | 0.637078614 | Advanced stage prostate cancer | CNS |
| Substantia_nigra | H3K4me1 | 1.60 | 1.47 | 0.680982846 | Advanced stage prostate cancer | CNS |
| Hippocampus_middle | H3K9ac | -0.05 | 3.21 | 0.743327148 | Advanced stage prostate cancer | CNS |
| Mobilized_CD34 | H3K27ac | 1.60 | 1.88 | 0.748935992 | Advanced stage prostate cancer | Hematopoietic |
| Cingulate_gyrus | H3K4me1 | 1.45 | 1.47 | 0.756965487 | Advanced stage prostate cancer | CNS |
| Duodenum_smooth_muscle | H3K4me1 | 2.60 | 5.47 | 0.767199197 | Advanced stage prostate cancer | GI |
| Inferior_temporal_lobe | H3K4me3 | 1.86 | 3.40 | 0.79842298 | Advanced stage prostate cancer | CNS |
| Mid_frontal_lobe | H3K4me1 | -0.82 | 8.62 | 0.832940432 | Advanced stage prostate cancer | CNS |
| Substantia_nigra | H3K9ac | 0.31 | 3.45 | 0.84128196 | Advanced stage prostate cancer | CNS |
| Liver_(UCSD) | H3K4me1 | 4.24 | 16.30 | 0.841952696 | Advanced stage prostate cancer | Liver |
| Inferior_temporal_lobe | H3K9ac | 0.43 | 3.27 | 0.861312498 | Advanced stage prostate cancer | CNS |
| Ovary | H3K4me1 | 1.64 | 4.17 | 0.87746356 | Advanced stage prostate cancer | Other |
| Anterior_caudate | H3K9ac | 0.49 | 3.87 | 0.895391742 | Advanced stage prostate cancer | CNS |
| Mid_frontal_lobe | H3K9ac | 0.53 | 3.65 | 0.896504207 | Advanced stage prostate cancer | CNS |
| Substantia_nigra | H3K4me3 | 1.54 | 4.21 | 0.897761008 | Advanced stage prostate cancer | CNS |
| Anterior_caudate | H3K4me1 | 0.85 | 1.55 | 0.921719033 | Advanced stage prostate cancer | CNS |
| Breast_fibroblast_primary | H3K4me3 | 0.49 | 8.08 | 0.949160946 | Advanced stage prostate cancer | Connective_Bone |
| Cingulate_gyrus | H3K9ac | 0.88 | 3.08 | 0.967804089 | Advanced stage prostate cancer | CNS |
| Adipose_nuclei | H3K4me1 | 3.99 | 1.03 | 0.003446777 | ER-negative breast cancer | Other |
| Stomach_mucosa | H3K4me1 | 6.14 | 1.79 | 0.003597221 | ER-negative breast cancer | GI |
| Breast_myoepithelial | H3K4me1 | 6.99 | 1.95 | 0.003640011 | ER-negative breast cancer | Other |
| Placenta_amnion | H3K4me3 | 19.19 | 6.27 | 0.004299735 | ER-negative breast cancer | Other |
| Stomach_mucosa | H3K9ac | 11.07 | 3.78 | 0.006658984 | ER-negative breast cancer | GI |
| Skeletal_muscle | H3K4me1 | 3.62 | 0.96 | 0.007521678 | ER-negative breast cancer | SkeletalMuscle |
| Fetal_stomach | H3K4me3 | 14.60 | 5.36 | 0.009997271 | ER-negative breast cancer | GI |
| Breast_myoepithelial | H3K4me3 | 9.31 | 3.23 | 0.010418358 | ER-negative breast cancer | Other |
| Fetal_leg_muscle | H3K4me3 | 15.74 | 5.87 | 0.011479743 | ER-negative breast cancer | SkeletalMuscle |
| Stomach_smooth_muscle | H3K4me3 | 7.41 | 2.55 | 0.011646449 | ER-negative breast cancer | GI |
| Sigmoid_colon | H3K4me3 | 20.69 | 7.86 | 0.012228158 | ER-negative breast cancer | GI |
| Breast_vHMEC | H3K4me1 | 5.96 | 2.02 | 0.015256406 | ER-negative breast cancer | Other |
| Right_atrium | H3K4me1 | 9.81 | 3.60 | 0.015565344 | ER-negative breast cancer | Cardiovascular |
| Ovary | H3K4me3 | 11.33 | 4.36 | 0.015610619 | ER-negative breast cancer | Other |
| Penis_foreskin_fibroblast_primary | H3K4me1 | 3.17 | 0.92 | 0.016075587 | ER-negative breast cancer | Connective_Bone |
| Duodenum_Mucosa | H3K4me1 | 4.55 | 1.50 | 0.016426529 | ER-negative breast cancer | GI |
| Adipose_nuclei | H3K9ac | 6.28 | 2.27 | 0.01707386 | ER-negative breast cancer | Other |
| Fetal_small_intestine | H3K4me3 | 11.43 | 4.52 | 0.018230399 | ER-negative breast cancer | GI |
| Right_ventricle | H3K4me3 | 11.22 | 4.32 | 0.018266573 | ER-negative breast cancer | Cardiovascular |
| Right_atrium | H3K4me3 | 11.04 | 4.31 | 0.020351376 | ER-negative breast cancer | Cardiovascular |
| Kidney | H3K4me3 | 9.38 | 3.71 | 0.021217539 | ER-negative breast cancer | Kidney |
| Stomach_smooth_muscle | H3K4me1 | 5.76 | 2.07 | 0.021454737 | ER-negative breast cancer | GI |
| CD4+_CD25-_IL17-_PMA_Ionomycin_stim_MACS_Th_sprimary | H3K4me1 | 3.21 | 0.97 | 0.02573186 | ER-negative breast cancer | Hematopoietic |
| Duodenum_smooth_muscle | H3K4me1 | 15.62 | 6.47 | 0.026050521 | ER-negative breast cancer | GI |
| CD4_memory_primary | H3K4me1 | 3.48 | 1.09 | 0.026108113 | ER-negative breast cancer | Hematopoietic |
| Rectal_mucosa | H3K4me3 | 8.98 | 3.65 | 0.028330626 | ER-negative breast cancer | GI |
| CD4+_CD25-_Th_primary | H3K4me1 | 3.61 | 1.17 | 0.028569695 | ER-negative breast cancer | Hematopoietic |
| Small_intestine | H3K4me3 | 21.09 | 9.26 | 0.029260885 | ER-negative breast cancer | GI |
| Fetal_stomach | H3K4me1 | 4.99 | 1.82 | 0.030466042 | ER-negative breast cancer | GI |
| Colonic_mucosa | H3K4me3 | 11.17 | 4.74 | 0.030613171 | ER-negative breast cancer | GI |
| Penis_foreskin_keratinocyte_primary | H3K4me1 | 4.40 | 1.55 | 0.031302154 | ER-negative breast cancer | Other |
| Adipose_nuclei | H3K27ac | 6.19 | 2.47 | 0.032942671 | ER-negative breast cancer | Other |
| Duodenum_Mucosa | H3K4me3 | 7.87 | 3.27 | 0.033211084 | ER-negative breast cancer | GI |
| CD4+_CD25-_IL17+_PMA_Ionomycin_stim_Th17_primary | H3K4me1 | 3.68 | 1.25 | 0.03349569 | ER-negative breast cancer | Hematopoietic |
| Psoas_muscle | H3K4me3 | 10.35 | 4.43 | 0.035680526 | ER-negative breast cancer | SkeletalMuscle |
| Left_Ventricle | H3K4me1 | 4.51 | 1.65 | 0.035973436 | ER-negative breast cancer | Cardiovascular |
| Fetal_trunk_muscle | H3K4me3 | 13.87 | 6.17 | 0.03645451 | ER-negative breast cancer | SkeletalMuscle |
| Skeletal_muscle | H3K4me3 | 6.87 | 2.83 | 0.037739613 | ER-negative breast cancer | SkeletalMuscle |
| Liver_(UCSD) | H3K4me3 | 10.01 | 4.45 | 0.038183039 | ER-negative breast cancer | Liver |
| Colonic_mucosa | H3K4me1 | 6.62 | 2.68 | 0.038190329 | ER-negative breast cancer | GI |
| Fetal_large_intestine | H3K4me3 | 10.28 | 4.56 | 0.038404172 | ER-negative breast cancer | GI |
| Fetal_brain | H3K4me1 | 3.01 | 0.95 | 0.038593535 | ER-negative breast cancer | CNS |
| Lung | H3K4me3 | 12.96 | 5.84 | 0.038896059 | ER-negative breast cancer | Cardiovascular |
| Colon_smooth_muscle | H3K4me3 | 8.99 | 3.94 | 0.041288455 | ER-negative breast cancer | GI |
| CD4+_CD25+_CD127-_Treg_primary | H3K4me1 | 3.99 | 1.45 | 0.041989159 | ER-negative breast cancer | Hematopoietic |
| Rectal_smooth_muscle | H3K4me3 | 9.21 | 4.07 | 0.042386622 | ER-negative breast cancer | GI |
| Fetal_adrenal | H3K4me3 | 10.67 | 4.79 | 0.043006825 | ER-negative breast cancer | Adrenal_Pancreas |
| Pancreatic_islets | H3K4me1 | 12.41 | 5.58 | 0.043249259 | ER-negative breast cancer | Adrenal_Pancreas |
| Fetal_leg_muscle | H3K4me1 | 4.49 | 1.69 | 0.044293912 | ER-negative breast cancer | SkeletalMuscle |
| Kidney | H3K9ac | 9.01 | 4.01 | 0.044378547 | ER-negative breast cancer | Kidney |
| Colonic_mucosa | H3K27ac | 5.53 | 2.23 | 0.044460707 | ER-negative breast cancer | GI |
| Adipose_nuclei | H3K4me3 | 5.25 | 2.15 | 0.04478395 | ER-negative breast cancer | Other |
| Penis_foreskin_keratinocyte_primary | H3K4me3 | 7.14 | 3.06 | 0.04482718 | ER-negative breast cancer | Other |
| Spleen | H3K4me1 | 3.96 | 1.47 | 0.045314281 | ER-negative breast cancer | Hematopoietic |
| Rectal_mucosa | H3K4me1 | 4.88 | 1.95 | 0.046898178 | ER-negative breast cancer | GI |
| Skeletal_muscle | H3K27ac | 3.94 | 1.47 | 0.048001411 | ER-negative breast cancer | SkeletalMuscle |
| Duodenum_smooth_muscle | H3K4me3 | 7.24 | 3.22 | 0.049087512 | ER-negative breast cancer | GI |
| CD4+_CD25-_CD45R0+_memory_primary | H3K4me1 | 3.47 | 1.25 | 0.049977623 | ER-negative breast cancer | Hematopoietic |
| Fetal_small_intestine | H3K4me1 | 4.40 | 1.80 | 0.051632608 | ER-negative breast cancer | GI |
| Anterior_caudate | H3K4me1 | 3.79 | 1.41 | 0.052377546 | ER-negative breast cancer | CNS |
| Colon_smooth_muscle | H3K4me1 | 4.15 | 1.63 | 0.052757571 | ER-negative breast cancer | GI |
| Anterior_caudate | H3K4me3 | 6.67 | 2.95 | 0.052886747 | ER-negative breast cancer | CNS |
| Esophagus | H3K4me3 | 10.74 | 4.96 | 0.053440431 | ER-negative breast cancer | GI |
| Germinal_matrix | H3K4me3 | 11.03 | 5.26 | 0.054014609 | ER-negative breast cancer | CNS |
| Fetal_brain | H3K4me3 | 8.54 | 3.97 | 0.055440262 | ER-negative breast cancer | CNS |
| Mobilized_CD34_primary | H3K4me1 | 2.93 | 1.01 | 0.056907982 | ER-negative breast cancer | Hematopoietic |
| Gastric | H3K4me3 | 14.92 | 7.42 | 0.05797322 | ER-negative breast cancer | GI |
| Pancreatic_islets | H3K27ac | 3.10 | 1.10 | 0.058316177 | ER-negative breast cancer | Adrenal_Pancreas |
| Colonic_mucosa | H3K9ac | 6.20 | 2.79 | 0.058802165 | ER-negative breast cancer | GI |
| CD4+_CD25-_CD45RA+_naive_primary | H3K4me1 | 3.27 | 1.20 | 0.061354199 | ER-negative breast cancer | Hematopoietic |
| Mid_frontal_lobe | H3K4me3 | 7.31 | 3.42 | 0.061761668 | ER-negative breast cancer | CNS |
| Fetal_adrenal | H3K4me1 | 4.04 | 1.59 | 0.062457432 | ER-negative breast cancer | Adrenal_Pancreas |
| Fetal_large_intestine | H3K4me1 | 4.32 | 1.83 | 0.063817741 | ER-negative breast cancer | GI |
| Fetal_trunk_muscle | H3K4me1 | 4.11 | 1.64 | 0.064836979 | ER-negative breast cancer | SkeletalMuscle |
| CD8_memory_primary | H3K4me1 | 3.39 | 1.28 | 0.065756161 | ER-negative breast cancer | Hematopoietic |
| Liver_(BI) | H3K4me1 | 3.34 | 1.30 | 0.065835824 | ER-negative breast cancer | Liver |
| Lung | H3K4me1 | 5.58 | 2.59 | 0.072241985 | ER-negative breast cancer | Cardiovascular |
| CD34_primary | H3K4me3 | 8.76 | 4.35 | 0.073180006 | ER-negative breast cancer | Hematopoietic |
| CD4_naive_primary | H3K4me1 | 3.35 | 1.30 | 0.075556936 | ER-negative breast cancer | Hematopoietic |
| Pancreas | H3K4me1 | 4.29 | 1.90 | 0.076638578 | ER-negative breast cancer | Adrenal_Pancreas |
| Placenta_chorion | H3K4me3 | 10.72 | 5.55 | 0.077585691 | ER-negative breast cancer | Other |
| Liver_(BI) | H3K4me3 | 7.16 | 3.64 | 0.083694659 | ER-negative breast cancer | Liver |
| CD19_primary_(BI) | H3K4me3 | 8.10 | 4.08 | 0.083947098 | ER-negative breast cancer | Hematopoietic |
| CD19_primary_(BI) | H3K4me1 | 3.53 | 1.46 | 0.08413956 | ER-negative breast cancer | Hematopoietic |
| Cingulate_gyrus | H3K4me1 | 3.08 | 1.20 | 0.085653715 | ER-negative breast cancer | CNS |
| Inferior_temporal_lobe | H3K4me1 | 2.91 | 1.12 | 0.089449803 | ER-negative breast cancer | CNS |
| Hippocampus_middle | H3K4me1 | 2.94 | 1.13 | 0.090462751 | ER-negative breast cancer | CNS |
| Rectal_mucosa | H3K9ac | 6.11 | 3.08 | 0.093085269 | ER-negative breast cancer | GI |
| Duodenum_Mucosa | H3K9ac | 5.79 | 2.90 | 0.093683615 | ER-negative breast cancer | GI |
| Th1 | H3K27ac | 2.95 | 1.17 | 0.094099997 | ER-negative breast cancer | Hematopoietic |
| Th2 | H3K27ac | 3.01 | 1.22 | 0.09496371 | ER-negative breast cancer | Hematopoietic |
| CD3_primary_(UW) | H3K4me1 | 3.39 | 1.43 | 0.099808465 | ER-negative breast cancer | Hematopoietic |
| Angular_gyrus | H3K4me3 | 7.92 | 4.24 | 0.100718498 | ER-negative breast cancer | CNS |
| Substantia_nigra | H3K4me1 | 3.00 | 1.23 | 0.102474879 | ER-negative breast cancer | CNS |
| Fetal_placenta | H3K4me3 | 9.84 | 5.53 | 0.107417243 | ER-negative breast cancer | Other |
| CD25+_CD127-_Treg | H3K27ac | 2.85 | 1.12 | 0.109325184 | ER-negative breast cancer | Hematopoietic |
| Mobilized_CD34_primary | H3K4me3 | 5.46 | 2.81 | 0.109494568 | ER-negative breast cancer | Hematopoietic |
| Chondrogenic_dif | H3K27ac | 3.61 | 1.70 | 0.115884793 | ER-negative breast cancer | Connective_Bone |
| CD19_primary_(UW) | H3K4me1 | 3.24 | 1.43 | 0.11895338 | ER-negative breast cancer | Hematopoietic |
| Fetal_brain | H3K9ac | 7.21 | 3.93 | 0.119030881 | ER-negative breast cancer | CNS |
| Penis_foreskin_melanocyte_primary | H3K4me1 | 2.62 | 1.04 | 0.119067009 | ER-negative breast cancer | Other |
| Skeletal_muscle | H3K9ac | 4.53 | 2.25 | 0.119170218 | ER-negative breast cancer | SkeletalMuscle |
| Pancreatic_islets | H3K4me3 | 7.71 | 4.32 | 0.119456018 | ER-negative breast cancer | Adrenal_Pancreas |
| Stomach_smooth_muscle | H3K27ac | 2.94 | 1.26 | 0.122991107 | ER-negative breast cancer | GI |
| Stomach_mucosa | H3K4me3 | 12.63 | 7.67 | 0.123604526 | ER-negative breast cancer | GI |
| Inferior_temporal_lobe | H3K4me3 | 6.04 | 3.32 | 0.124236694 | ER-negative breast cancer | CNS |
| CD34_primary | H3K4me1 | 3.61 | 1.70 | 0.124241476 | ER-negative breast cancer | Hematopoietic |
| Cingulate_gyrus | H3K4me3 | 5.86 | 3.25 | 0.12790129 | ER-negative breast cancer | CNS |
| Fetal_placenta | H3K4me1 | 5.02 | 2.59 | 0.129341955 | ER-negative breast cancer | Other |
| Fetal_lung | H3K4me3 | 8.50 | 5.01 | 0.130337518 | ER-negative breast cancer | Cardiovascular |
| CD14_primary | H3K4me3 | 8.21 | 4.81 | 0.132463986 | ER-negative breast cancer | Hematopoietic |
| CD15_primary | H3K4me3 | 5.81 | 3.24 | 0.136965317 | ER-negative breast cancer | Hematopoietic |
| Left_Ventricle | H3K4me3 | 8.49 | 5.11 | 0.142910445 | ER-negative breast cancer | Cardiovascular |
| Pancreas | H3K4me3 | 8.38 | 5.06 | 0.143310261 | ER-negative breast cancer | Adrenal_Pancreas |
| Osteoblast | H3K27ac | 2.95 | 1.36 | 0.14460791 | ER-negative breast cancer | Connective_Bone |
| Rectal_mucosa | H3K27ac | 3.91 | 2.03 | 0.145885937 | ER-negative breast cancer | GI |
| Breast_luminal_epithelial | H3K4me1 | 25.62 | 16.81 | 0.147376191 | ER-negative breast cancer | Other |
| CD8_naive_primary_(BI) | H3K4me1 | 2.89 | 1.30 | 0.1490865 | ER-negative breast cancer | Hematopoietic |
| Fetal_kidney | H3K9ac | 11.33 | 7.27 | 0.151342308 | ER-negative breast cancer | Kidney |
| Fetal_thymus | H3K4me3 | 8.50 | 5.25 | 0.156517763 | ER-negative breast cancer | Hematopoietic |
| Breast_fibroblast_primary | H3K4me1 | 7.41 | 4.61 | 0.157426462 | ER-negative breast cancer | Connective_Bone |
| CD3_primary_(BI) | H3K4me1 | 3.05 | 1.44 | 0.157516037 | ER-negative breast cancer | Hematopoietic |
| CD19_primary_(UW) | H3K4me3 | 8.17 | 5.08 | 0.162037007 | ER-negative breast cancer | Hematopoietic |
| CD19 | H3K27ac | 3.44 | 1.78 | 0.169140184 | ER-negative breast cancer | Hematopoietic |
| Penis_foreskin_fibroblast_primary | H3K4me3 | 3.43 | 1.79 | 0.17283504 | ER-negative breast cancer | Connective_Bone |
| Substantia_nigra | H3K4me3 | 5.96 | 3.81 | 0.18604252 | ER-negative breast cancer | CNS |
| CD4+_CD25-_Th_primary | H3K4me3 | 5.78 | 3.61 | 0.189144551 | ER-negative breast cancer | Hematopoietic |
| CD4+_CD25int_CD127+_Tmem_primary | H3K4me1 | 3.96 | 2.25 | 0.189161599 | ER-negative breast cancer | Hematopoietic |
| Fetal_thymus | H3K4me1 | 2.82 | 1.43 | 0.206456114 | ER-negative breast cancer | Hematopoietic |
| Gastric | H3K4me1 | 5.67 | 3.73 | 0.208350063 | ER-negative breast cancer | GI |
| Peripheralblood_mononuclear_primary | H3K4me3 | 6.50 | 4.42 | 0.213841762 | ER-negative breast cancer | Hematopoietic |
| Spleen | H3K4me3 | 11.01 | 8.28 | 0.2214094 | ER-negative breast cancer | Hematopoietic |
| CD4+_CD25-_IL17+_PMA_Ionomycin_stim_Th17_primary | H3K4me3 | 4.86 | 3.20 | 0.226841101 | ER-negative breast cancer | Hematopoietic |
| Ovary | H3K4me1 | 8.38 | 6.27 | 0.233671854 | ER-negative breast cancer | Other |
| CD20 | H3K27ac | 3.05 | 1.73 | 0.236840776 | ER-negative breast cancer | Hematopoietic |
| Fetal_heart | H3K4me1 | 2.39 | 1.18 | 0.237394064 | ER-negative breast cancer | Cardiovascular |
| Treg_primary | H3K4me3 | 3.34 | 1.98 | 0.240372312 | ER-negative breast cancer | Hematopoietic |
| Pancreatic_islets | H3K4me1 | 8.21 | 6.22 | 0.244049269 | ER-negative breast cancer | Adrenal_Pancreas |
| Colon_smooth_muscle | H3K27ac | 2.61 | 1.40 | 0.249158126 | ER-negative breast cancer | GI |
| CD4+_CD25-_CD45R0+_memory_primary | H3K4me3 | 5.74 | 4.13 | 0.252376572 | ER-negative breast cancer | Hematopoietic |
| CD56_primary | H3K4me3 | 6.70 | 4.98 | 0.255414246 | ER-negative breast cancer | Hematopoietic |
| Breast_vHMEC | H3K4me3 | 6.12 | 4.59 | 0.258831653 | ER-negative breast cancer | Other |
| Duodenum_smooth_muscle | H3K27ac | 3.02 | 1.87 | 0.272449261 | ER-negative breast cancer | GI |
| Rectal_smooth_muscle | H3K27ac | 2.44 | 1.32 | 0.272798084 | ER-negative breast cancer | GI |
| Kidney | H3K27ac | 2.77 | 1.62 | 0.276581002 | ER-negative breast cancer | Kidney |
| CD4_memory_primary | H3K4me3 | 4.55 | 3.27 | 0.278031019 | ER-negative breast cancer | Hematopoietic |
| Hippocampus_middle | H3K4me3 | 4.20 | 3.02 | 0.282140353 | ER-negative breast cancer | CNS |
| Rectal_smooth_muscle | H3K4me1 | 4.26 | 3.13 | 0.296680167 | ER-negative breast cancer | GI |
| Aorta | H3K4me3 | 5.04 | 3.98 | 0.307659176 | ER-negative breast cancer | Cardiovascular |
| Stomach_smooth_muscle | H3K9ac | 3.82 | 2.79 | 0.309117814 | ER-negative breast cancer | GI |
| Placenta_amnion | H3K4me1 | 11.77 | 10.56 | 0.310007495 | ER-negative breast cancer | Other |
| CD15_primary | H3K4me1 | 2.27 | 1.26 | 0.313327359 | ER-negative breast cancer | Hematopoietic |
| Kidney | H3K4me1 | 5.11 | 4.08 | 0.316836638 | ER-negative breast cancer | Kidney |
| CD56_primary | H3K4me1 | 2.40 | 1.44 | 0.328644624 | ER-negative breast cancer | Hematopoietic |
| Cingulate_gyrus | H3K9ac | 3.43 | 2.48 | 0.329451607 | ER-negative breast cancer | CNS |
| Penis_foreskin_melanocyte_primary | H3K4me3 | 3.92 | 3.02 | 0.330243572 | ER-negative breast cancer | Other |
| CD25int_CD127+_Tmem | H3K27ac | 2.40 | 1.45 | 0.338667099 | ER-negative breast cancer | Hematopoietic |
| Psoas_muscle | H3K4me1 | 7.32 | 6.67 | 0.346768186 | ER-negative breast cancer | SkeletalMuscle |
| CD8_naive_primary_(UCSF-UBC) | H3K9ac | -8.30 | 10.53 | 0.369884797 | ER-negative breast cancer | Hematopoietic |
| CD4+_CD25int_CD127+_Tmem_primary | H3K4me3 | 4.41 | 3.88 | 0.380885161 | ER-negative breast cancer | Hematopoietic |
| Fetal_lung | H3K4me1 | 1.95 | 1.09 | 0.386214346 | ER-negative breast cancer | Cardiovascular |
| Angular_gyrus | H3K27ac | 0.10 | 1.05 | 0.388545952 | ER-negative breast cancer | CNS |
| CD25-_CD45RA+_naive | H3K27ac | 2.21 | 1.40 | 0.3888431 | ER-negative breast cancer | Hematopoietic |
| Small_intestine | H3K4me1 | 18.85 | 20.59 | 0.388896778 | ER-negative breast cancer | GI |
| Th0 | H3K27ac | 2.01 | 1.17 | 0.388902727 | ER-negative breast cancer | Hematopoietic |
| CD14_primary | H3K4me1 | 2.11 | 1.32 | 0.392955221 | ER-negative breast cancer | Hematopoietic |
| Angular_gyrus | H3K4me1 | 2.98 | 2.32 | 0.392985857 | ER-negative breast cancer | CNS |
| CD4+_CD25-_IL17-_PMA_Ionomycin_stim_MACS_Th_sprimary | H3K4me3 | 3.06 | 2.46 | 0.398384493 | ER-negative breast cancer | Hematopoietic |
| Mid_frontal_lobe | H3K4me1 | 5.82 | 5.69 | 0.400669911 | ER-negative breast cancer | CNS |
| CD4+_CD25+_CD127-_Treg_primary | H3K4me3 | 3.46 | 2.92 | 0.40111742 | ER-negative breast cancer | Hematopoietic |
| Pancreatic_islets | H3K4me3 | 4.97 | 4.80 | 0.403952611 | ER-negative breast cancer | Adrenal_Pancreas |
| Mobilized_CD34 | H3K27ac | 2.33 | 1.61 | 0.404270063 | ER-negative breast cancer | Hematopoietic |
| Fetal_brain | H3K4me3 | 9.11 | 9.90 | 0.40979125 | ER-negative breast cancer | CNS |
| CD8_memory_primary | H3K4me3 | 3.76 | 3.34 | 0.412193115 | ER-negative breast cancer | Hematopoietic |
| Liver_(BI) | H3K9ac | 3.41 | 3.11 | 0.43085723 | ER-negative breast cancer | Liver |
| Substantia_nigra | H3K9ac | 2.91 | 2.45 | 0.433822471 | ER-negative breast cancer | CNS |
| Fetal_lung | H3K9ac | 3.60 | 3.66 | 0.473525085 | ER-negative breast cancer | Cardiovascular |
| CD4_naive_primary | H3K4me3 | 3.31 | 3.25 | 0.476709288 | ER-negative breast cancer | Hematopoietic |
| CD3_primary_(BI) | H3K4me3 | 3.15 | 3.04 | 0.478468048 | ER-negative breast cancer | Hematopoietic |
| Fetal_heart | H3K9ac | 2.60 | 2.30 | 0.484020527 | ER-negative breast cancer | Cardiovascular |
| Penis_foreskin_keratinocyte_primary | H3K9ac | 2.43 | 2.06 | 0.486167431 | ER-negative breast cancer | Other |
| CD4+_CD25-_CD45RA+_naive_primary | H3K4me3 | 3.22 | 3.19 | 0.486397823 | ER-negative breast cancer | Hematopoietic |
| Liver | H3K27ac | 2.03 | 1.55 | 0.499529611 | ER-negative breast cancer | Liver |
| CD4_primary | H3K4me3 | 3.27 | 3.41 | 0.506583571 | ER-negative breast cancer | Hematopoietic |
| CD3_primary_(UW) | H3K4me3 | 3.58 | 3.97 | 0.518525276 | ER-negative breast cancer | Hematopoietic |
| CD8_naive_primary_(UCSF-UBC) | H3K4me1 | 1.89 | 1.39 | 0.522795758 | ER-negative breast cancer | Hematopoietic |
| CD8_naive_primary_(UCSF-UBC) | H3K4me3 | 4.11 | 4.90 | 0.526038425 | ER-negative breast cancer | Hematopoietic |
| Pancreatic_islets | H3K9ac | 4.74 | 6.32 | 0.549423702 | ER-negative breast cancer | Adrenal_Pancreas |
| Placenta_chorion | H3K4me1 | 3.53 | 4.60 | 0.581964584 | ER-negative breast cancer | Other |
| Anterior_caudate | H3K9ac | 2.45 | 2.78 | 0.600674967 | ER-negative breast cancer | CNS |
| CD8_primary | H3K4me3 | 2.97 | 3.92 | 0.616621336 | ER-negative breast cancer | Hematopoietic |
| CD14 | H3K27ac | 0.41 | 1.19 | 0.616719448 | ER-negative breast cancer | Hematopoietic |
| Liver_(UCSD) | H3K4me1 | 7.41 | 13.01 | 0.620820728 | ER-negative breast cancer | Liver |
| CD3_primary | H3K27ac | 1.55 | 1.14 | 0.629485052 | ER-negative breast cancer | Hematopoietic |
| Neurosphere | H3K27ac | 1.58 | 1.22 | 0.630631199 | ER-negative breast cancer | CNS |
| Duodenum_mucosa | H3K27ac | 1.83 | 1.78 | 0.63837087 | ER-negative breast cancer | GI |
| Inferior_temporal_lobe | H3K27ac | 0.57 | 0.96 | 0.6550926 | ER-negative breast cancer | CNS |
| Substantia_nigra | H3K27ac | 0.60 | 0.91 | 0.659414564 | ER-negative breast cancer | CNS |
| Hippocampus_middle | H3K9ac | 2.08 | 2.56 | 0.671018719 | ER-negative breast cancer | CNS |
| Hippocampus_middle | H3K27ac | 0.54 | 1.15 | 0.687122584 | ER-negative breast cancer | CNS |
| Cingulate_gyrus | H3K27ac | 0.62 | 0.93 | 0.687171786 | ER-negative breast cancer | CNS |
| Thymus | H3K4me1 | 2.31 | 3.43 | 0.701691773 | ER-negative breast cancer | Hematopoietic |
| Esophagus | H3K4me1 | 3.33 | 6.29 | 0.710729141 | ER-negative breast cancer | GI |
| Sigmoid_colon | H3K4me1 | 4.08 | 8.74 | 0.724655429 | ER-negative breast cancer | GI |
| CD25-_IL17+_Th17_stim | H3K27ac | 1.48 | 1.43 | 0.73794152 | ER-negative breast cancer | Hematopoietic |
| Inferior_temporal_lobe | H3K9ac | 1.86 | 2.66 | 0.745062678 | ER-negative breast cancer | CNS |
| Mid_frontal_lobe | H3K9ac | 1.96 | 3.04 | 0.752707405 | ER-negative breast cancer | CNS |
| CD8_naive_primary_(BI) | H3K4me3 | 2.08 | 3.53 | 0.758083111 | ER-negative breast cancer | Hematopoietic |
| Anterior_caudate | H3K27ac | 0.67 | 1.29 | 0.794898351 | ER-negative breast cancer | CNS |
| Mid_frontal_lobe | H3K27ac | 1.35 | 1.49 | 0.812739294 | ER-negative breast cancer | CNS |
| Peripheralblood_mononuclear_primary | H3K9ac | 1.61 | 3.29 | 0.852515842 | ER-negative breast cancer | Hematopoietic |
| Breast_myoepithelial | H3K9ac | 0.13 | 6.68 | 0.896051615 | ER-negative breast cancer | Other |
| CD25-_IL17-_Th_stim_MACS | H3K27ac | 1.16 | 1.33 | 0.901364119 | ER-negative breast cancer | Hematopoietic |
| Rectal_smooth_muscle | H3K9ac | 1.75 | 8.04 | 0.92504831 | ER-negative breast cancer | GI |
| Right_ventricle | H3K4me1 | -0.08 | 13.99 | 0.938263412 | ER-negative breast cancer | Cardiovascular |
| Angular_gyrus | H3K9ac | 1.28 | 3.94 | 0.942749639 | ER-negative breast cancer | CNS |
| Peripheralblood_mononuclear_primary | H3K4me1 | 1.23 | 4.19 | 0.955369334 | ER-negative breast cancer | Hematopoietic |
| Colon_smooth_muscle | H3K9ac | 1.09 | 4.37 | 0.983131169 | ER-negative breast cancer | GI |
| Fetal_heart | H3K4me3 | 1.08 | 6.62 | 0.990010935 | ER-negative breast cancer | Cardiovascular |
| Breast_fibroblast_primary | H3K4me3 | 1.07 | 5.73 | 0.990357866 | ER-negative breast cancer | Connective_Bone |
| Adipose_nuclei | H3K4me1 | 6.05 | 1.01 | **2.05E-06** | ER-positive breast cancer | Other |
| Breast_myoepithelial | H3K4me1 | 7.09 | 1.29 | **7.22E-06** | ER-positive breast cancer | Other |
| Rectal_mucosa | H3K9ac | 13.09 | 2.78 | **1.38E-05** | ER-positive breast cancer | GI |
| Fetal_small_intestine | H3K4me1 | 8.77 | 1.73 | **1.38E-05** | ER-positive breast cancer | GI |
| Fetal_large_intestine | H3K4me1 | 8.72 | 1.79 | **2.75E-05** | ER-positive breast cancer | GI |
| Duodenum_Mucosa | H3K4me3 | 9.66 | 2.09 | **2.98E-05** | ER-positive breast cancer | GI |
| Penis_foreskin_fibroblast_primary | H3K4me1 | 4.88 | 0.91 | **3.00E-05** | ER-positive breast cancer | Connective_Bone |
| Fetal_lung | H3K4me1 | 5.53 | 1.06 | **3.92E-05** | ER-positive breast cancer | Cardiovascular |
| Adipose_nuclei | H3K9ac | 10.20 | 2.25 | **4.16E-05** | ER-positive breast cancer | Other |
| Penis_foreskin_keratinocyte_primary | H3K4me1 | 6.36 | 1.31 | **4.57E-05** | ER-positive breast cancer | Other |
| Liver_(BI) | H3K4me1 | 5.45 | 1.15 | **5.30E-05** | ER-positive breast cancer | Liver |
| Penis_foreskin_fibroblast_primary | H3K4me3 | 6.33 | 1.28 | **6.28E-05** | ER-positive breast cancer | Connective_Bone |
| Kidney | H3K4me3 | 10.44 | 2.39 | **7.34E-05** | ER-positive breast cancer | Kidney |
| Breast_vHMEC | H3K4me1 | 7.70 | 1.73 | **7.70E-05** | ER-positive breast cancer | Other |
| Colonic_mucosa | H3K9ac | 13.04 | 3.05 | **7.80E-05** | ER-positive breast cancer | GI |
| Rectal_smooth_muscle | H3K4me3 | 14.00 | 3.34 | **0.000121576** | ER-positive breast cancer | GI |
| Stomach_mucosa | H3K4me1 | 10.09 | 2.38 | **0.000146335** | ER-positive breast cancer | GI |
| Rectal_mucosa | H3K4me3 | 12.21 | 3.00 | **0.000167472** | ER-positive breast cancer | GI |
| Colon_smooth_muscle | H3K4me3 | 12.41 | 2.97 | **0.000186277** | ER-positive breast cancer | GI |
| Duodenum_Mucosa | H3K4me1 | 7.03 | 1.63 | **0.000190875** | ER-positive breast cancer | GI |
| Fetal_large_intestine | H3K4me3 | 13.75 | 3.29 | **0.000194252** | ER-positive breast cancer | GI |
| Stomach_mucosa | H3K9ac | 14.02 | 3.55 | **0.000221769** | ER-positive breast cancer | GI |
| Fetal_lung | H3K9ac | 14.13 | 3.48 | 0.000239057 | ER-positive breast cancer | Cardiovascular |
| Liver_(BI) | H3K9ac | 10.43 | 2.61 | 0.000265096 | ER-positive breast cancer | Liver |
| Rectal_mucosa | H3K4me1 | 8.51 | 2.12 | 0.000274278 | ER-positive breast cancer | GI |
| Skeletal_muscle | H3K4me1 | 5.33 | 1.15 | 0.000333832 | ER-positive breast cancer | SkeletalMuscle |
| Stomach_smooth_muscle | H3K4me3 | 10.14 | 2.44 | 0.00034094 | ER-positive breast cancer | GI |
| Fetal_small_intestine | H3K4me3 | 13.22 | 3.31 | 0.000364321 | ER-positive breast cancer | GI |
| Colonic_mucosa | H3K4me1 | 11.42 | 2.95 | 0.000394575 | ER-positive breast cancer | GI |
| Placenta_amnion | H3K4me3 | 20.41 | 5.27 | 0.00040751 | ER-positive breast cancer | Other |
| Duodenum_Mucosa | H3K9ac | 9.73 | 2.52 | 0.000459484 | ER-positive breast cancer | GI |
| Adipose_nuclei | H3K4me3 | 7.39 | 1.79 | 0.000463348 | ER-positive breast cancer | Other |
| Esophagus | H3K4me3 | 18.25 | 4.85 | 0.000531787 | ER-positive breast cancer | GI |
| Penis_foreskin_melanocyte_primary | H3K4me1 | 4.14 | 0.87 | 0.000568891 | ER-positive breast cancer | Other |
| Small_intestine | H3K4me3 | 19.49 | 5.18 | 0.000579875 | ER-positive breast cancer | GI |
| Fetal_placenta | H3K4me1 | 10.56 | 2.70 | 0.000665175 | ER-positive breast cancer | Other |
| Kidney | H3K27ac | 7.20 | 1.80 | 0.000713614 | ER-positive breast cancer | Kidney |
| Colonic_mucosa | H3K4me3 | 14.90 | 4.10 | 0.000794699 | ER-positive breast cancer | GI |
| Stomach_smooth_muscle | H3K9ac | 10.00 | 2.70 | 0.000997715 | ER-positive breast cancer | GI |
| Liver_(UCSD) | H3K4me3 | 10.45 | 2.92 | 0.001137336 | ER-positive breast cancer | Liver |
| Fetal_adrenal | H3K4me1 | 6.85 | 1.72 | 0.001152193 | ER-positive breast cancer | Adrenal_Pancreas |
| Duodenum_smooth_muscle | H3K4me3 | 8.27 | 2.24 | 0.001172794 | ER-positive breast cancer | GI |
| Spleen | H3K4me1 | 6.41 | 1.57 | 0.001267703 | ER-positive breast cancer | Hematopoietic |
| Penis_foreskin_keratinocyte_primary | H3K4me3 | 9.38 | 2.58 | 0.001312287 | ER-positive breast cancer | Other |
| Colon_smooth_muscle | H3K4me1 | 6.75 | 1.76 | 0.001412816 | ER-positive breast cancer | GI |
| CD14_primary | H3K4me1 | 5.16 | 1.28 | 0.001587403 | ER-positive breast cancer | Hematopoietic |
| Colonic_mucosa | H3K27ac | 7.25 | 1.89 | 0.001620536 | ER-positive breast cancer | GI |
| Stomach_smooth_muscle | H3K4me1 | 8.22 | 2.20 | 0.001761452 | ER-positive breast cancer | GI |
| Fetal_stomach | H3K4me1 | 9.24 | 2.44 | 0.002038921 | ER-positive breast cancer | GI |
| Pancreas | H3K4me1 | 7.48 | 2.17 | 0.002751546 | ER-positive breast cancer | Adrenal_Pancreas |
| Liver_(BI) | H3K4me3 | 8.44 | 2.51 | 0.00289653 | ER-positive breast cancer | Liver |
| Psoas_muscle | H3K4me3 | 11.62 | 3.54 | 0.00291333 | ER-positive breast cancer | SkeletalMuscle |
| Kidney | H3K9ac | 18.17 | 5.91 | 0.003025988 | ER-positive breast cancer | Kidney |
| Penis_foreskin_melanocyte_primary | H3K4me3 | 6.92 | 1.95 | 0.003035625 | ER-positive breast cancer | Other |
| CD15_primary | H3K4me1 | 4.17 | 1.07 | 0.003238515 | ER-positive breast cancer | Hematopoietic |
| Mobilized_CD34_primary | H3K4me1 | 4.64 | 1.24 | 0.003313106 | ER-positive breast cancer | Hematopoietic |
| Rectal_mucosa | H3K27ac | 6.31 | 1.76 | 0.003534153 | ER-positive breast cancer | GI |
| Right_ventricle | H3K4me3 | 10.55 | 3.25 | 0.003538862 | ER-positive breast cancer | Cardiovascular |
| CD19_primary_(BI) | H3K4me1 | 4.94 | 1.32 | 0.003714988 | ER-positive breast cancer | Hematopoietic |
| Fetal_leg_muscle | H3K4me1 | 7.46 | 2.05 | 0.004010489 | ER-positive breast cancer | SkeletalMuscle |
| Fetal_placenta | H3K4me3 | 12.59 | 3.97 | 0.004846117 | ER-positive breast cancer | Other |
| Fetal_adrenal | H3K4me3 | 11.74 | 3.75 | 0.005008614 | ER-positive breast cancer | Adrenal_Pancreas |
| Sigmoid_colon | H3K4me3 | 16.67 | 5.50 | 0.005086772 | ER-positive breast cancer | GI |
| Breast_myoepithelial | H3K4me3 | 9.32 | 2.97 | 0.005140815 | ER-positive breast cancer | Other |
| CD34_primary | H3K4me3 | 11.44 | 3.65 | 0.005217474 | ER-positive breast cancer | Hematopoietic |
| Skeletal_muscle | H3K4me3 | 9.83 | 3.17 | 0.006038847 | ER-positive breast cancer | SkeletalMuscle |
| Adipose_nuclei | H3K27ac | 6.25 | 1.94 | 0.006806528 | ER-positive breast cancer | Other |
| Fetal_stomach | H3K4me3 | 13.23 | 4.43 | 0.006861971 | ER-positive breast cancer | GI |
| Fetal_heart | H3K9ac | 7.97 | 2.59 | 0.006988222 | ER-positive breast cancer | Cardiovascular |
| Skeletal_muscle | H3K9ac | 9.35 | 3.08 | 0.007184651 | ER-positive breast cancer | SkeletalMuscle |
| Fetal_brain | H3K9ac | 8.99 | 2.92 | 0.007265907 | ER-positive breast cancer | CNS |
| Fetal_thymus | H3K4me3 | 10.63 | 3.61 | 0.008259199 | ER-positive breast cancer | Hematopoietic |
| Duodenum_mucosa | H3K27ac | 6.31 | 2.01 | 0.009677434 | ER-positive breast cancer | GI |
| Osteoblast | H3K27ac | 3.62 | 1.04 | 0.009927963 | ER-positive breast cancer | Connective_Bone |
| Gastric | H3K4me3 | 13.16 | 4.67 | 0.010867193 | ER-positive breast cancer | GI |
| CD15_primary | H3K4me3 | 8.30 | 2.82 | 0.011012714 | ER-positive breast cancer | Hematopoietic |
| CD19_primary_(UW) | H3K4me1 | 4.37 | 1.30 | 0.011566792 | ER-positive breast cancer | Hematopoietic |
| CD14_primary | H3K4me3 | 10.76 | 3.75 | 0.011802473 | ER-positive breast cancer | Hematopoietic |
| Rectal_smooth_muscle | H3K4me1 | 11.74 | 4.14 | 0.011844173 | ER-positive breast cancer | GI |
| Mobilized_CD34_primary | H3K4me3 | 10.34 | 3.70 | 0.012307769 | ER-positive breast cancer | Hematopoietic |
| Colon_smooth_muscle | H3K9ac | 11.25 | 4.21 | 0.01266296 | ER-positive breast cancer | GI |
| CD4+_CD25-_IL17-_PMA_Ionomycin_stim_MACS_Th_sprimary | H3K4me1 | 3.20 | 0.88 | 0.013517456 | ER-positive breast cancer | Hematopoietic |
| Angular_gyrus | H3K27ac | -1.69 | 1.03 | 0.013747235 | ER-positive breast cancer | CNS |
| Mid_frontal_lobe | H3K27ac | -1.72 | 1.09 | 0.01414893 | ER-positive breast cancer | CNS |
| Inferior_temporal_lobe | H3K27ac | -1.35 | 0.90 | 0.01434339 | ER-positive breast cancer | CNS |
| CD4+_CD25-_IL17+_PMA_Ionomycin_stim_Th17_primary | H3K4me1 | 4.37 | 1.36 | 0.014495112 | ER-positive breast cancer | Hematopoietic |
| Fetal_leg_muscle | H3K4me3 | 18.78 | 7.23 | 0.01521142 | ER-positive breast cancer | SkeletalMuscle |
| CD4+_CD25-_IL17+_PMA_Ionomycin_stim_Th17_primary | H3K4me3 | 8.02 | 2.87 | 0.015348983 | ER-positive breast cancer | Hematopoietic |
| Ovary | H3K4me3 | 8.89 | 3.31 | 0.01546738 | ER-positive breast cancer | Other |
| Fetal_trunk_muscle | H3K4me3 | 19.35 | 7.48 | 0.01549371 | ER-positive breast cancer | SkeletalMuscle |
| Peripheralblood_mononuclear_primary | H3K9ac | 9.67 | 3.56 | 0.016794596 | ER-positive breast cancer | Hematopoietic |
| Breast_vHMEC | H3K4me3 | 11.08 | 4.23 | 0.017579912 | ER-positive breast cancer | Other |
| Spleen | H3K4me3 | 21.37 | 8.40 | 0.018443977 | ER-positive breast cancer | Hematopoietic |
| Left_Ventricle | H3K4me3 | 11.50 | 4.39 | 0.018708477 | ER-positive breast cancer | Cardiovascular |
| Liver | H3K27ac | 3.80 | 1.20 | 0.018734331 | ER-positive breast cancer | Liver |
| Chondrogenic_dif | H3K27ac | 5.83 | 2.01 | 0.019494383 | ER-positive breast cancer | Connective_Bone |
| CD34_primary | H3K4me1 | 6.38 | 2.34 | 0.020092897 | ER-positive breast cancer | Hematopoietic |
| Skeletal_muscle | H3K27ac | 5.07 | 1.74 | 0.021110724 | ER-positive breast cancer | SkeletalMuscle |
| Fetal_trunk_muscle | H3K4me1 | 5.68 | 1.94 | 0.022328616 | ER-positive breast cancer | SkeletalMuscle |
| Stomach_mucosa | H3K4me3 | 12.03 | 4.85 | 0.023112207 | ER-positive breast cancer | GI |
| Aorta | H3K4me3 | 9.18 | 3.65 | 0.023229686 | ER-positive breast cancer | Cardiovascular |
| Placenta_chorion | H3K4me3 | 13.63 | 5.51 | 0.023407037 | ER-positive breast cancer | Other |
| CD4+_CD25-_Th_primary | H3K4me1 | 3.58 | 1.14 | 0.025209222 | ER-positive breast cancer | Hematopoietic |
| Fetal_heart | H3K4me1 | 3.93 | 1.30 | 0.025682793 | ER-positive breast cancer | Cardiovascular |
| Right_atrium | H3K4me3 | 8.92 | 3.56 | 0.025705819 | ER-positive breast cancer | Cardiovascular |
| Fetal_thymus | H3K4me1 | 4.17 | 1.41 | 0.025981806 | ER-positive breast cancer | Hematopoietic |
| Gastric | H3K4me1 | 11.44 | 4.62 | 0.026005649 | ER-positive breast cancer | GI |
| CD4+_CD25-_IL17-_PMA_Ionomycin_stim_MACS_Th_sprimary | H3K4me3 | 6.05 | 2.26 | 0.026358929 | ER-positive breast cancer | Hematopoietic |
| Kidney | H3K4me1 | 15.57 | 6.68 | 0.02694331 | ER-positive breast cancer | Kidney |
| Duodenum_smooth_muscle | H3K4me1 | 21.21 | 9.20 | 0.027307215 | ER-positive breast cancer | GI |
| Fetal_lung | H3K4me3 | 9.43 | 3.83 | 0.027946203 | ER-positive breast cancer | Cardiovascular |
| Penis_foreskin_keratinocyte_primary | H3K9ac | 4.89 | 1.76 | 0.028175108 | ER-positive breast cancer | Other |
| Right_atrium | H3K4me1 | 15.27 | 6.43 | 0.029518491 | ER-positive breast cancer | Cardiovascular |
| Pancreas | H3K4me3 | 9.51 | 3.91 | 0.030256945 | ER-positive breast cancer | Adrenal_Pancreas |
| CD4+_CD25+_CD127-_Treg_primary | H3K4me3 | 6.77 | 2.62 | 0.030339065 | ER-positive breast cancer | Hematopoietic |
| CD4+_CD25-_CD45RA+_naive_primary | H3K4me1 | 3.30 | 1.05 | 0.031399255 | ER-positive breast cancer | Hematopoietic |
| Left_Ventricle | H3K4me1 | 5.95 | 2.27 | 0.031565421 | ER-positive breast cancer | Cardiovascular |
| CD4+_CD25+_CD127-_Treg_primary | H3K4me1 | 4.68 | 1.70 | 0.032887432 | ER-positive breast cancer | Hematopoietic |
| Duodenum_smooth_muscle | H3K27ac | 4.45 | 1.59 | 0.033565565 | ER-positive breast cancer | GI |
| CD19_primary_(BI) | H3K4me3 | 8.56 | 3.52 | 0.035362294 | ER-positive breast cancer | Hematopoietic |
| CD3_primary_(BI) | H3K4me1 | 4.41 | 1.58 | 0.035881079 | ER-positive breast cancer | Hematopoietic |
| Germinal_matrix | H3K4me3 | 8.23 | 3.44 | 0.036615929 | ER-positive breast cancer | CNS |
| Peripheralblood_mononuclear_primary | H3K4me3 | 8.48 | 3.54 | 0.038422858 | ER-positive breast cancer | Hematopoietic |
| CD4+_CD25-_CD45R0+_memory_primary | H3K4me1 | 3.81 | 1.35 | 0.039159698 | ER-positive breast cancer | Hematopoietic |
| Colon_smooth_muscle | H3K27ac | 5.38 | 2.07 | 0.0404789 | ER-positive breast cancer | GI |
| CD4+_CD25-_CD45R0+_memory_primary | H3K4me3 | 8.60 | 3.67 | 0.041113446 | ER-positive breast cancer | Hematopoietic |
| Fetal_kidney | H3K9ac | 12.28 | 5.56 | 0.043189028 | ER-positive breast cancer | Kidney |
| Lung | H3K4me3 | 10.85 | 4.84 | 0.043223559 | ER-positive breast cancer | Cardiovascular |
| Pancreatic_islets | H3K4me3 | 7.79 | 3.33 | 0.04437434 | ER-positive breast cancer | Adrenal_Pancreas |
| Peripheralblood_mononuclear_primary | H3K4me1 | 11.89 | 5.35 | 0.045847137 | ER-positive breast cancer | Hematopoietic |
| Treg_primary | H3K4me3 | 5.64 | 2.33 | 0.049556171 | ER-positive breast cancer | Hematopoietic |
| Liver_(UCSD) | H3K4me1 | 22.83 | 11.10 | 0.049888462 | ER-positive breast cancer | Liver |
| CD4_primary | H3K4me3 | 6.88 | 2.95 | 0.049914063 | ER-positive breast cancer | Hematopoietic |
| CD4_memory_primary | H3K4me3 | 6.53 | 2.78 | 0.051329066 | ER-positive breast cancer | Hematopoietic |
| CD4_memory_primary | H3K4me1 | 3.15 | 1.10 | 0.051382915 | ER-positive breast cancer | Hematopoietic |
| Lung | H3K4me1 | 8.01 | 3.59 | 0.051785472 | ER-positive breast cancer | Cardiovascular |
| CD56_primary | H3K4me3 | 8.81 | 4.00 | 0.056022148 | ER-positive breast cancer | Hematopoietic |
| Anterior_caudate | H3K9ac | 6.29 | 2.84 | 0.058084146 | ER-positive breast cancer | CNS |
| Fetal_brain | H3K4me3 | 6.34 | 2.82 | 0.058759969 | ER-positive breast cancer | CNS |
| CD19_primary_(UW) | H3K4me3 | 9.20 | 4.23 | 0.058899453 | ER-positive breast cancer | Hematopoietic |
| Breast_fibroblast_primary | H3K4me1 | 9.38 | 4.58 | 0.059012866 | ER-positive breast cancer | Connective_Bone |
| CD4+_CD25int_CD127+_Tmem_primary | H3K4me1 | 5.97 | 2.60 | 0.060200394 | ER-positive breast cancer | Hematopoietic |
| Rectal_smooth_muscle | H3K27ac | 4.09 | 1.61 | 0.061372803 | ER-positive breast cancer | GI |
| Rectal_smooth_muscle | H3K9ac | 13.24 | 6.41 | 0.061871077 | ER-positive breast cancer | GI |
| CD3_primary_(UW) | H3K4me1 | 3.58 | 1.38 | 0.064371913 | ER-positive breast cancer | Hematopoietic |
| CD4+_CD25-_Th_primary | H3K4me3 | 6.30 | 2.87 | 0.070714592 | ER-positive breast cancer | Hematopoietic |
| CD3_primary_(UW) | H3K4me3 | 7.35 | 3.49 | 0.072508296 | ER-positive breast cancer | Hematopoietic |
| CD8_memory_primary | H3K4me3 | 6.15 | 2.84 | 0.073487894 | ER-positive breast cancer | Hematopoietic |
| CD8_naive_primary_(BI) | H3K4me3 | 6.24 | 2.90 | 0.076089843 | ER-positive breast cancer | Hematopoietic |
| Esophagus | H3K4me1 | 18.94 | 10.07 | 0.076706132 | ER-positive breast cancer | GI |
| CD4+_CD25int_CD127+_Tmem_primary | H3K4me3 | 7.07 | 3.39 | 0.077545187 | ER-positive breast cancer | Hematopoietic |
| Breast_luminal_epithelial | H3K4me1 | 38.57 | 21.24 | 0.079337261 | ER-positive breast cancer | Other |
| CD3_primary_(BI) | H3K4me3 | 6.04 | 2.85 | 0.081696362 | ER-positive breast cancer | Hematopoietic |
| Cingulate_gyrus | H3K9ac | 4.30 | 1.92 | 0.082782677 | ER-positive breast cancer | CNS |
| Placenta_chorion | H3K4me1 | 11.69 | 6.16 | 0.083860105 | ER-positive breast cancer | Other |
| CD8_primary | H3K4me3 | 6.74 | 3.28 | 0.085335757 | ER-positive breast cancer | Hematopoietic |
| CD4+_CD25-_CD45RA+_naive_primary | H3K4me3 | 5.89 | 2.83 | 0.089617213 | ER-positive breast cancer | Hematopoietic |
| Stomach_smooth_muscle | H3K27ac | 3.32 | 1.39 | 0.099740547 | ER-positive breast cancer | GI |
| CD4_naive_primary | H3K4me3 | 5.52 | 2.72 | 0.100608556 | ER-positive breast cancer | Hematopoietic |
| Placenta_amnion | H3K4me1 | 26.15 | 15.12 | 0.103811789 | ER-positive breast cancer | Other |
| Substantia_nigra | H3K9ac | 5.22 | 2.66 | 0.10564438 | ER-positive breast cancer | CNS |
| Substantia_nigra | H3K4me3 | 5.27 | 2.69 | 0.105675612 | ER-positive breast cancer | CNS |
| Breast_fibroblast_primary | H3K4me3 | 9.11 | 5.03 | 0.107113414 | ER-positive breast cancer | Connective_Bone |
| Hippocampus_middle | H3K9ac | 4.24 | 2.06 | 0.111151511 | ER-positive breast cancer | CNS |
| CD8_naive_primary_(UCSF-UBC) | H3K4me3 | 8.36 | 4.60 | 0.116092045 | ER-positive breast cancer | Hematopoietic |
| CD8_memory_primary | H3K4me1 | 3.03 | 1.29 | 0.116157467 | ER-positive breast cancer | Hematopoietic |
| Breast_myoepithelial | H3K9ac | 9.07 | 5.09 | 0.117369899 | ER-positive breast cancer | Other |
| Neurosphere | H3K27ac | 3.05 | 1.29 | 0.117834078 | ER-positive breast cancer | CNS |
| Sigmoid_colon | H3K4me1 | 22.33 | 13.83 | 0.118374933 | ER-positive breast cancer | GI |
| CD8_naive_primary_(BI) | H3K4me1 | 2.96 | 1.26 | 0.119390833 | ER-positive breast cancer | Hematopoietic |
| Ovary | H3K4me1 | 10.52 | 6.03 | 0.119722732 | ER-positive breast cancer | Other |
| CD56_primary | H3K4me1 | 3.44 | 1.60 | 0.130268287 | ER-positive breast cancer | Hematopoietic |
| CD4_naive_primary | H3K4me1 | 2.91 | 1.27 | 0.135770281 | ER-positive breast cancer | Hematopoietic |
| Mobilized_CD34 | H3K27ac | 4.02 | 2.07 | 0.144372688 | ER-positive breast cancer | Hematopoietic |
| Pancreatic_islets | H3K9ac | 8.00 | 4.81 | 0.146738469 | ER-positive breast cancer | Adrenal_Pancreas |
| Pancreatic_islets | H3K27ac | 3.15 | 1.50 | 0.147166855 | ER-positive breast cancer | Adrenal_Pancreas |
| Hippocampus_middle | H3K4me1 | 2.62 | 1.15 | 0.150496815 | ER-positive breast cancer | CNS |
| Small_intestine | H3K4me1 | 30.43 | 20.92 | 0.158561382 | ER-positive breast cancer | GI |
| Anterior_caudate | H3K4me1 | 2.97 | 1.44 | 0.160916313 | ER-positive breast cancer | CNS |
| Cingulate_gyrus | H3K27ac | -0.47 | 1.04 | 0.169789274 | ER-positive breast cancer | CNS |
| Psoas_muscle | H3K4me1 | 13.43 | 9.16 | 0.174656029 | ER-positive breast cancer | SkeletalMuscle |
| CD25-_CD45RA+_naive | H3K27ac | -0.30 | 0.97 | 0.175495914 | ER-positive breast cancer | Hematopoietic |
| Hippocampus_middle | H3K4me3 | 3.54 | 1.93 | 0.181124087 | ER-positive breast cancer | CNS |
| Right_ventricle | H3K4me1 | 47.50 | 34.52 | 0.181212479 | ER-positive breast cancer | Cardiovascular |
| CD20 | H3K27ac | 3.15 | 1.68 | 0.202674914 | ER-positive breast cancer | Hematopoietic |
| Mid_frontal_lobe | H3K4me3 | 3.81 | 2.28 | 0.211290364 | ER-positive breast cancer | CNS |
| Mid_frontal_lobe | H3K9ac | 4.02 | 2.54 | 0.233302699 | ER-positive breast cancer | CNS |
| CD19 | H3K27ac | 3.05 | 1.73 | 0.23936619 | ER-positive breast cancer | Hematopoietic |
| Substantia_nigra | H3K4me1 | 2.53 | 1.36 | 0.251024134 | ER-positive breast cancer | CNS |
| CD8_naive_primary_(UCSF-UBC) | H3K9ac | 12.03 | 9.66 | 0.255467603 | ER-positive breast cancer | Hematopoietic |
| Th0 | H3K27ac | -0.04 | 0.93 | 0.258980057 | ER-positive breast cancer | Hematopoietic |
| Mid_frontal_lobe | H3K4me1 | 7.50 | 5.88 | 0.263769056 | ER-positive breast cancer | CNS |
| Cingulate_gyrus | H3K4me3 | 3.43 | 2.21 | 0.265376527 | ER-positive breast cancer | CNS |
| Inferior_temporal_lobe | H3K9ac | 3.24 | 2.05 | 0.269288632 | ER-positive breast cancer | CNS |
| Anterior_caudate | H3K4me3 | 3.20 | 2.09 | 0.284803443 | ER-positive breast cancer | CNS |
| Angular_gyrus | H3K4me3 | 4.44 | 3.32 | 0.296134666 | ER-positive breast cancer | CNS |
| CD25-_IL17+_Th17_stim | H3K27ac | 2.50 | 1.45 | 0.296676008 | ER-positive breast cancer | Hematopoietic |
| Fetal_brain | H3K4me3 | 6.86 | 5.99 | 0.324814679 | ER-positive breast cancer | CNS |
| Pancreatic_islets | H3K4me3 | 4.32 | 3.48 | 0.334268829 | ER-positive breast cancer | Adrenal_Pancreas |
| Anterior_caudate | H3K27ac | -0.19 | 1.28 | 0.360846469 | ER-positive breast cancer | CNS |
| CD8_naive_primary_(UCSF-UBC) | H3K4me1 | 2.28 | 1.43 | 0.370002629 | ER-positive breast cancer | Hematopoietic |
| Angular_gyrus | H3K9ac | 3.42 | 2.83 | 0.389334829 | ER-positive breast cancer | CNS |
| Pancreatic_islets | H3K4me1 | 9.10 | 9.85 | 0.409493743 | ER-positive breast cancer | Adrenal_Pancreas |
| Inferior_temporal_lobe | H3K4me1 | 1.84 | 1.09 | 0.433387408 | ER-positive breast cancer | CNS |
| Substantia_nigra | H3K27ac | 0.16 | 1.06 | 0.437678402 | ER-positive breast cancer | CNS |
| Pancreatic_islets | H3K4me1 | 4.35 | 4.67 | 0.470402562 | ER-positive breast cancer | Adrenal_Pancreas |
| Inferior_temporal_lobe | H3K4me3 | 2.45 | 2.06 | 0.478396759 | ER-positive breast cancer | CNS |
| Cingulate_gyrus | H3K4me1 | 1.84 | 1.21 | 0.481110291 | ER-positive breast cancer | CNS |
| Fetal_heart | H3K4me3 | 4.08 | 4.43 | 0.484624823 | ER-positive breast cancer | Cardiovascular |
| CD25int_CD127+_Tmem | H3K27ac | 2.39 | 2.08 | 0.503579021 | ER-positive breast cancer | Hematopoietic |
| CD25+_CD127-_Treg | H3K27ac | 1.79 | 1.37 | 0.562491757 | ER-positive breast cancer | Hematopoietic |
| Hippocampus_middle | H3K27ac | 0.23 | 1.40 | 0.586679878 | ER-positive breast cancer | CNS |
| Th2 | H3K27ac | 0.53 | 0.97 | 0.627965194 | ER-positive breast cancer | Hematopoietic |
| CD25-_IL17-_Th_stim_MACS | H3K27ac | 0.44 | 1.22 | 0.647922075 | ER-positive breast cancer | Hematopoietic |
| Angular_gyrus | H3K4me1 | 1.97 | 2.40 | 0.683685345 | ER-positive breast cancer | CNS |
| Fetal_brain | H3K4me1 | 0.86 | 0.68 | 0.839985893 | ER-positive breast cancer | CNS |
| CD14 | H3K27ac | 1.20 | 1.11 | 0.855736687 | ER-positive breast cancer | Hematopoietic |
| CD3_primary | H3K27ac | 0.85 | 1.19 | 0.899153253 | ER-positive breast cancer | Hematopoietic |
| Thymus | H3K4me1 | 1.29 | 2.60 | 0.910525204 | ER-positive breast cancer | Hematopoietic |
| Th1 | H3K27ac | 1.00 | 0.99 | 0.99810821 | ER-positive breast cancer | Hematopoietic |
| CD56_primary | H3K4me1 | 11.08 | 2.91 | 0.000969316 | Ever-smoking lung cancer | Hematopoietic |
| CD8_naive_primary_(UCSF-UBC) | H3K4me1 | 11.87 | 3.39 | 0.001875585 | Ever-smoking lung cancer | Hematopoietic |
| CD4+_CD25-_IL17-_PMA_Ionomycin_stim_MACS_Th_sprimary | H3K4me1 | 7.54 | 1.98 | 0.002155879 | Ever-smoking lung cancer | Hematopoietic |
| CD4_memory_primary | H3K4me1 | 7.99 | 2.30 | 0.002204689 | Ever-smoking lung cancer | Hematopoietic |
| CD4+_CD25-_Th_primary | H3K4me1 | 9.09 | 2.40 | 0.002519255 | Ever-smoking lung cancer | Hematopoietic |
| CD4+_CD25-_CD45R0+_memory_primary | H3K4me1 | 8.81 | 2.66 | 0.003592364 | Ever-smoking lung cancer | Hematopoietic |
| CD3_primary | H3K27ac | 9.43 | 3.21 | 0.0047116 | Ever-smoking lung cancer | Hematopoietic |
| CD8_memory_primary | H3K4me1 | 8.82 | 2.90 | 0.004910805 | Ever-smoking lung cancer | Hematopoietic |
| Th0 | H3K27ac | 10.06 | 3.50 | 0.005213899 | Ever-smoking lung cancer | Hematopoietic |
| CD15_primary | H3K4me1 | 7.25 | 2.47 | 0.006428955 | Ever-smoking lung cancer | Hematopoietic |
| CD3_primary_(UW) | H3K4me1 | 9.47 | 2.90 | 0.00673916 | Ever-smoking lung cancer | Hematopoietic |
| CD14_primary | H3K4me1 | 7.35 | 2.59 | 0.006917603 | Ever-smoking lung cancer | Hematopoietic |
| CD4_naive_primary | H3K4me1 | 8.53 | 2.73 | 0.007179006 | Ever-smoking lung cancer | Hematopoietic |
| CD4+_CD25+_CD127-_Treg_primary | H3K4me1 | 9.33 | 3.12 | 0.008194817 | Ever-smoking lung cancer | Hematopoietic |
| CD14 | H3K27ac | 8.52 | 3.06 | 0.009209465 | Ever-smoking lung cancer | Hematopoietic |
| CD4+_CD25-_IL17+_PMA_Ionomycin_stim_Th17_primary | H3K4me1 | 7.98 | 2.56 | 0.01049123 | Ever-smoking lung cancer | Hematopoietic |
| CD25-_IL17+_Th17_stim | H3K27ac | 11.66 | 4.04 | 0.0111747 | Ever-smoking lung cancer | Hematopoietic |
| CD4+_CD25-_CD45RA+_naive_primary | H3K4me1 | 7.47 | 2.33 | 0.011507391 | Ever-smoking lung cancer | Hematopoietic |
| Rectal_mucosa | H3K4me3 | 15.25 | 6.06 | 0.013116086 | Ever-smoking lung cancer | GI |
| Placenta_chorion | H3K4me1 | -19.55 | 8.82 | 0.013302804 | Ever-smoking lung cancer | Other |
| Treg_primary | H3K4me3 | 13.11 | 4.67 | 0.013708982 | Ever-smoking lung cancer | Hematopoietic |
| CD34_primary | H3K4me1 | 8.87 | 3.19 | 0.013948677 | Ever-smoking lung cancer | Hematopoietic |
| CD8_naive_primary_(BI) | H3K4me1 | 8.22 | 2.79 | 0.017423909 | Ever-smoking lung cancer | Hematopoietic |
| CD19_primary_(BI) | H3K4me1 | 7.40 | 2.52 | 0.017757238 | Ever-smoking lung cancer | Hematopoietic |
| Duodenum_Mucosa | H3K4me3 | 13.72 | 5.60 | 0.018413998 | Ever-smoking lung cancer | GI |
| CD3_primary_(BI) | H3K4me1 | 7.46 | 2.75 | 0.019666294 | Ever-smoking lung cancer | Hematopoietic |
| Th1 | H3K27ac | 8.72 | 3.34 | 0.020113796 | Ever-smoking lung cancer | Hematopoietic |
| Fetal_small_intestine | H3K4me3 | 23.50 | 9.43 | 0.022463029 | Ever-smoking lung cancer | GI |
| Mobilized_CD34_primary | H3K4me1 | 5.54 | 1.86 | 0.02342339 | Ever-smoking lung cancer | Hematopoietic |
| CD15_primary | H3K4me3 | 14.54 | 5.93 | 0.024850705 | Ever-smoking lung cancer | Hematopoietic |
| Fetal_thymus | H3K4me3 | 35.38 | 14.03 | 0.024966554 | Ever-smoking lung cancer | Hematopoietic |
| Fetal_large_intestine | H3K4me3 | 20.58 | 8.79 | 0.025854418 | Ever-smoking lung cancer | GI |
| Fetal_thymus | H3K4me1 | 7.53 | 2.98 | 0.030368184 | Ever-smoking lung cancer | Hematopoietic |
| Mobilized_CD34_primary | H3K4me3 | 14.11 | 5.75 | 0.034081173 | Ever-smoking lung cancer | Hematopoietic |
| CD4+_CD25+_CD127-_Treg_primary | H3K4me3 | 18.65 | 7.64 | 0.035525745 | Ever-smoking lung cancer | Hematopoietic |
| CD25+_CD127-_Treg | H3K27ac | 7.79 | 3.35 | 0.036684976 | Ever-smoking lung cancer | Hematopoietic |
| CD8_primary | H3K4me3 | 22.23 | 9.42 | 0.036845049 | Ever-smoking lung cancer | Hematopoietic |
| CD4+_CD25-_Th_primary | H3K4me3 | 21.86 | 9.24 | 0.040646451 | Ever-smoking lung cancer | Hematopoietic |
| Duodenum_smooth_muscle | H3K4me3 | 12.06 | 5.11 | 0.042127466 | Ever-smoking lung cancer | GI |
| Rectal_smooth_muscle | H3K4me3 | 18.15 | 8.64 | 0.044169908 | Ever-smoking lung cancer | GI |
| Th2 | H3K27ac | 7.72 | 3.62 | 0.047932684 | Ever-smoking lung cancer | Hematopoietic |
| CD34_primary | H3K4me3 | 22.42 | 10.42 | 0.052094621 | Ever-smoking lung cancer | Hematopoietic |
| CD19_primary_(UW) | H3K4me1 | 6.51 | 2.56 | 0.053712582 | Ever-smoking lung cancer | Hematopoietic |
| CD4_naive_primary | H3K4me3 | 18.66 | 8.34 | 0.053731276 | Ever-smoking lung cancer | Hematopoietic |
| CD25int_CD127+_Tmem | H3K27ac | 7.35 | 3.58 | 0.053795498 | Ever-smoking lung cancer | Hematopoietic |
| CD3_primary_(UW) | H3K4me3 | 19.35 | 9.02 | 0.054312783 | Ever-smoking lung cancer | Hematopoietic |
| Right_ventricle | H3K4me3 | 20.99 | 10.45 | 0.059432846 | Ever-smoking lung cancer | Cardiovascular |
| Colon_smooth_muscle | H3K4me3 | 15.82 | 8.06 | 0.060602876 | Ever-smoking lung cancer | GI |
| Peripheralblood_mononuclear_primary | H3K9ac | 19.02 | 9.28 | 0.065696434 | Ever-smoking lung cancer | Hematopoietic |
| CD4_primary | H3K4me3 | 16.97 | 8.01 | 0.065779822 | Ever-smoking lung cancer | Hematopoietic |
| CD8_naive_primary_(BI) | H3K4me3 | 19.61 | 9.44 | 0.066586844 | Ever-smoking lung cancer | Hematopoietic |
| Rectal_mucosa | H3K9ac | 10.66 | 5.44 | 0.067164939 | Ever-smoking lung cancer | GI |
| Hippocampus_middle | H3K4me3 | 12.48 | 6.31 | 0.071898993 | Ever-smoking lung cancer | CNS |
| CD4+_CD25-_CD45RA+_naive_primary | H3K4me3 | 17.22 | 8.30 | 0.073493478 | Ever-smoking lung cancer | Hematopoietic |
| Mobilized_CD34 | H3K27ac | 5.86 | 2.96 | 0.076421453 | Ever-smoking lung cancer | Hematopoietic |
| Left_Ventricle | H3K4me3 | 24.48 | 13.07 | 0.080731268 | Ever-smoking lung cancer | Cardiovascular |
| CD4+_CD25-_IL17+_PMA_Ionomycin_stim_Th17_primary | H3K4me3 | 16.18 | 8.39 | 0.082118037 | Ever-smoking lung cancer | Hematopoietic |
| Skeletal_muscle | H3K4me3 | 10.02 | 5.14 | 0.082784353 | Ever-smoking lung cancer | SkeletalMuscle |
| CD3_primary_(BI) | H3K4me3 | 15.40 | 7.56 | 0.083332328 | Ever-smoking lung cancer | Hematopoietic |
| CD19_primary_(BI) | H3K4me3 | 17.48 | 9.16 | 0.087787128 | Ever-smoking lung cancer | Hematopoietic |
| Penis_foreskin_fibroblast_primary | H3K4me3 | 5.56 | 2.81 | 0.088094889 | Ever-smoking lung cancer | Connective_Bone |
| Fetal_brain | H3K4me3 | 45.31 | 25.10 | 0.088131359 | Ever-smoking lung cancer | CNS |
| Duodenum_Mucosa | H3K9ac | 11.81 | 6.80 | 0.088309725 | Ever-smoking lung cancer | GI |
| CD4+_CD25int_CD127+_Tmem_primary | H3K4me1 | 8.63 | 4.56 | 0.09300225 | Ever-smoking lung cancer | Hematopoietic |
| Hippocampus_middle | H3K9ac | 9.36 | 5.06 | 0.093589723 | Ever-smoking lung cancer | CNS |
| Cingulate_gyrus | H3K4me3 | 11.96 | 6.41 | 0.096070386 | Ever-smoking lung cancer | CNS |
| Psoas_muscle | H3K4me1 | -19.72 | 13.07 | 0.096560059 | Ever-smoking lung cancer | SkeletalMuscle |
| CD25-_IL17-_Th_stim_MACS | H3K27ac | 6.76 | 3.43 | 0.098629207 | Ever-smoking lung cancer | Hematopoietic |
| CD56_primary | H3K4me3 | 22.26 | 12.08 | 0.098647893 | Ever-smoking lung cancer | Hematopoietic |
| Right_atrium | H3K4me3 | 18.19 | 10.31 | 0.100902242 | Ever-smoking lung cancer | Cardiovascular |
| Thymus | H3K4me1 | 21.39 | 12.30 | 0.101581174 | Ever-smoking lung cancer | Hematopoietic |
| CD8_naive_primary_(UCSF-UBC) | H3K9ac | 60.36 | 34.18 | 0.101765571 | Ever-smoking lung cancer | Hematopoietic |
| CD4+_CD25-_IL17-_PMA_Ionomycin_stim_MACS_Th_sprimary | H3K4me3 | 11.55 | 5.89 | 0.101845551 | Ever-smoking lung cancer | Hematopoietic |
| CD8_memory_primary | H3K4me3 | 14.96 | 8.10 | 0.104275048 | Ever-smoking lung cancer | Hematopoietic |
| CD25-_CD45RA+_naive | H3K27ac | 6.31 | 3.14 | 0.104495523 | Ever-smoking lung cancer | Hematopoietic |
| CD4+_CD25int_CD127+_Tmem_primary | H3K4me3 | 21.36 | 11.87 | 0.111213492 | Ever-smoking lung cancer | Hematopoietic |
| Fetal_lung | H3K4me3 | 15.64 | 9.02 | 0.11235862 | Ever-smoking lung cancer | Cardiovascular |
| Inferior_temporal_lobe | H3K4me3 | 11.18 | 6.37 | 0.11304672 | Ever-smoking lung cancer | CNS |
| Inferior_temporal_lobe | H3K9ac | 9.06 | 5.19 | 0.113733166 | Ever-smoking lung cancer | CNS |
| Fetal_brain | H3K9ac | 12.44 | 7.47 | 0.11701764 | Ever-smoking lung cancer | CNS |
| CD19_primary_(UW) | H3K4me3 | 22.25 | 13.31 | 0.12257191 | Ever-smoking lung cancer | Hematopoietic |
| Colonic_mucosa | H3K9ac | 7.79 | 4.66 | 0.122946484 | Ever-smoking lung cancer | GI |
| Fetal_kidney | H3K9ac | 32.27 | 19.69 | 0.122972744 | Ever-smoking lung cancer | Kidney |
| Rectal_smooth_muscle | H3K9ac | 34.78 | 21.60 | 0.123790367 | Ever-smoking lung cancer | GI |
| Skeletal_muscle | H3K9ac | 7.02 | 3.83 | 0.12758463 | Ever-smoking lung cancer | SkeletalMuscle |
| CD4+_CD25-_CD45R0+_memory_primary | H3K4me3 | 18.71 | 11.04 | 0.127714022 | Ever-smoking lung cancer | Hematopoietic |
| Stomach_smooth_muscle | H3K4me3 | 10.64 | 6.19 | 0.128754584 | Ever-smoking lung cancer | GI |
| Lung | H3K4me1 | -5.93 | 5.40 | 0.138875723 | Ever-smoking lung cancer | Cardiovascular |
| Fetal_adrenal | H3K4me3 | 20.06 | 12.65 | 0.144761596 | Ever-smoking lung cancer | Adrenal_Pancreas |
| Anterior_caudate | H3K4me3 | 9.60 | 5.96 | 0.145779047 | Ever-smoking lung cancer | CNS |
| Colonic_mucosa | H3K4me3 | 12.67 | 8.10 | 0.148407788 | Ever-smoking lung cancer | GI |
| Pancreatic_islets | H3K4me1 | -7.49 | 6.53 | 0.150073346 | Ever-smoking lung cancer | Adrenal_Pancreas |
| Penis_foreskin_melanocyte_primary | H3K4me3 | 8.98 | 5.50 | 0.15316459 | Ever-smoking lung cancer | Other |
| Peripheralblood_mononuclear_primary | H3K4me3 | 18.76 | 11.76 | 0.153706586 | Ever-smoking lung cancer | Hematopoietic |
| Germinal_matrix | H3K4me3 | 13.32 | 8.41 | 0.154312339 | Ever-smoking lung cancer | CNS |
| Mid_frontal_lobe | H3K4me3 | 10.18 | 6.60 | 0.156847802 | Ever-smoking lung cancer | CNS |
| Fetal_leg_muscle | H3K4me3 | 15.21 | 10.03 | 0.15774425 | Ever-smoking lung cancer | SkeletalMuscle |
| CD4_memory_primary | H3K4me3 | 13.60 | 8.50 | 0.16026414 | Ever-smoking lung cancer | Hematopoietic |
| Adipose_nuclei | H3K4me3 | 6.61 | 3.94 | 0.169116558 | Ever-smoking lung cancer | Other |
| Colon_smooth_muscle | H3K9ac | 17.86 | 12.48 | 0.171486631 | Ever-smoking lung cancer | GI |
| Angular_gyrus | H3K9ac | 11.23 | 7.51 | 0.171860706 | Ever-smoking lung cancer | CNS |
| CD14_primary | H3K4me3 | 16.59 | 11.03 | 0.174137608 | Ever-smoking lung cancer | Hematopoietic |
| Fetal_stomach | H3K4me3 | 15.54 | 10.50 | 0.176234617 | Ever-smoking lung cancer | GI |
| Substantia_nigra | H3K4me3 | 10.71 | 7.06 | 0.178840195 | Ever-smoking lung cancer | CNS |
| Penis_foreskin_keratinocyte_primary | H3K9ac | 5.29 | 3.40 | 0.185384505 | Ever-smoking lung cancer | Other |
| Skeletal_muscle | H3K27ac | 4.67 | 2.88 | 0.191775526 | Ever-smoking lung cancer | SkeletalMuscle |
| Colonic_mucosa | H3K27ac | 5.58 | 4.11 | 0.197014903 | Ever-smoking lung cancer | GI |
| Small_intestine | H3K4me3 | 27.26 | 20.06 | 0.202190972 | Ever-smoking lung cancer | GI |
| Gastric | H3K4me3 | 23.46 | 17.16 | 0.203686821 | Ever-smoking lung cancer | GI |
| Duodenum_Mucosa | H3K4me1 | 4.41 | 3.08 | 0.204749449 | Ever-smoking lung cancer | GI |
| Right_atrium | H3K4me1 | -8.34 | 8.17 | 0.205290695 | Ever-smoking lung cancer | Cardiovascular |
| Rectal_smooth_muscle | H3K27ac | 4.68 | 2.92 | 0.212495672 | Ever-smoking lung cancer | GI |
| Kidney | H3K4me1 | -8.99 | 7.95 | 0.213445114 | Ever-smoking lung cancer | Kidney |
| Cingulate_gyrus | H3K9ac | 6.84 | 4.76 | 0.215140235 | Ever-smoking lung cancer | CNS |
| Colon_smooth_muscle | H3K27ac | 4.53 | 2.84 | 0.216123232 | Ever-smoking lung cancer | GI |
| Pancreas | H3K4me1 | -2.40 | 3.05 | 0.217243608 | Ever-smoking lung cancer | Adrenal_Pancreas |
| Placenta_amnion | H3K4me3 | 19.16 | 14.53 | 0.218806293 | Ever-smoking lung cancer | Other |
| Sigmoid_colon | H3K4me3 | 25.08 | 19.78 | 0.231708261 | Ever-smoking lung cancer | GI |
| Fetal_placenta | H3K4me3 | 14.75 | 11.46 | 0.235883414 | Ever-smoking lung cancer | Other |
| CD8_naive_primary_(UCSF-UBC) | H3K4me3 | 19.74 | 15.61 | 0.243224751 | Ever-smoking lung cancer | Hematopoietic |
| Fetal_heart | H3K9ac | 6.53 | 4.78 | 0.256557626 | Ever-smoking lung cancer | Cardiovascular |
| Fetal_trunk_muscle | H3K4me3 | 13.49 | 11.01 | 0.262989431 | Ever-smoking lung cancer | SkeletalMuscle |
| Kidney | H3K4me3 | 7.76 | 6.02 | 0.263244566 | Ever-smoking lung cancer | Kidney |
| Neurosphere | H3K27ac | 3.86 | 2.55 | 0.268294884 | Ever-smoking lung cancer | CNS |
| Placenta_chorion | H3K4me3 | 13.07 | 10.79 | 0.269358146 | Ever-smoking lung cancer | Other |
| Lung | H3K4me3 | 16.72 | 14.30 | 0.270547391 | Ever-smoking lung cancer | Cardiovascular |
| Anterior_caudate | H3K27ac | 4.39 | 3.35 | 0.271536732 | Ever-smoking lung cancer | CNS |
| Stomach_mucosa | H3K9ac | 7.15 | 5.73 | 0.278339129 | Ever-smoking lung cancer | GI |
| Substantia_nigra | H3K27ac | 3.23 | 2.15 | 0.28261197 | Ever-smoking lung cancer | CNS |
| Chondrogenic_dif | H3K27ac | -1.80 | 2.86 | 0.286919543 | Ever-smoking lung cancer | Connective_Bone |
| Peripheralblood_mononuclear_primary | H3K4me1 | 11.25 | 9.48 | 0.291722243 | Ever-smoking lung cancer | Hematopoietic |
| Adipose_nuclei | H3K4me1 | 3.44 | 2.32 | 0.300322534 | Ever-smoking lung cancer | Other |
| Anterior_caudate | H3K9ac | 6.96 | 5.84 | 0.30295341 | Ever-smoking lung cancer | CNS |
| Aorta | H3K4me3 | 13.13 | 11.77 | 0.303658639 | Ever-smoking lung cancer | Cardiovascular |
| Fetal_brain | H3K4me3 | 8.51 | 7.31 | 0.31153862 | Ever-smoking lung cancer | CNS |
| Esophagus | H3K4me3 | 11.59 | 10.46 | 0.317826076 | Ever-smoking lung cancer | GI |
| Hippocampus_middle | H3K27ac | 3.92 | 3.17 | 0.319695065 | Ever-smoking lung cancer | CNS |
| Mid_frontal_lobe | H3K27ac | 4.91 | 4.37 | 0.321370325 | Ever-smoking lung cancer | CNS |
| Angular_gyrus | H3K4me1 | 6.35 | 5.65 | 0.325215336 | Ever-smoking lung cancer | CNS |
| Mid_frontal_lobe | H3K9ac | 6.80 | 6.15 | 0.326913832 | Ever-smoking lung cancer | CNS |
| Cingulate_gyrus | H3K27ac | 3.30 | 2.50 | 0.327009545 | Ever-smoking lung cancer | CNS |
| Stomach_mucosa | H3K4me3 | 16.94 | 16.12 | 0.329220312 | Ever-smoking lung cancer | GI |
| Substantia_nigra | H3K9ac | 6.21 | 5.36 | 0.331407002 | Ever-smoking lung cancer | CNS |
| Inferior_temporal_lobe | H3K27ac | 3.59 | 2.99 | 0.340741373 | Ever-smoking lung cancer | CNS |
| Angular_gyrus | H3K27ac | 3.95 | 3.39 | 0.341842622 | Ever-smoking lung cancer | CNS |
| Breast_fibroblast_primary | H3K4me3 | 18.09 | 17.95 | 0.343199136 | Ever-smoking lung cancer | Connective_Bone |
| Liver_(BI) | H3K4me3 | 6.31 | 5.52 | 0.343302522 | Ever-smoking lung cancer | Liver |
| Fetal_brain | H3K4me1 | 3.37 | 2.72 | 0.351290948 | Ever-smoking lung cancer | CNS |
| Breast_fibroblast_primary | H3K4me1 | -6.22 | 7.93 | 0.355263305 | Ever-smoking lung cancer | Connective_Bone |
| Fetal_lung | H3K4me1 | 2.87 | 2.01 | 0.357584279 | Ever-smoking lung cancer | Cardiovascular |
| Fetal_stomach | H3K4me1 | 4.15 | 3.39 | 0.358953417 | Ever-smoking lung cancer | GI |
| Psoas_muscle | H3K4me3 | 10.59 | 10.46 | 0.361003819 | Ever-smoking lung cancer | SkeletalMuscle |
| Sigmoid_colon | H3K4me1 | -15.25 | 18.37 | 0.372392288 | Ever-smoking lung cancer | GI |
| Skeletal_muscle | H3K4me1 | 2.70 | 1.86 | 0.374965291 | Ever-smoking lung cancer | SkeletalMuscle |
| Kidney | H3K27ac | -1.37 | 2.69 | 0.38461074 | Ever-smoking lung cancer | Kidney |
| Duodenum_smooth_muscle | H3K27ac | 4.53 | 4.17 | 0.388007408 | Ever-smoking lung cancer | GI |
| Small_intestine | H3K4me1 | 38.08 | 44.63 | 0.390541009 | Ever-smoking lung cancer | GI |
| Angular_gyrus | H3K4me3 | 8.92 | 9.20 | 0.394530142 | Ever-smoking lung cancer | CNS |
| CD20 | H3K27ac | 2.89 | 2.22 | 0.395679409 | Ever-smoking lung cancer | Hematopoietic |
| Ovary | H3K4me3 | 11.20 | 12.06 | 0.399037892 | Ever-smoking lung cancer | Other |
| Adipose_nuclei | H3K9ac | 5.09 | 4.94 | 0.403666779 | Ever-smoking lung cancer | Other |
| Liver_(UCSD) | H3K4me1 | -29.49 | 35.14 | 0.404259201 | Ever-smoking lung cancer | Liver |
| Breast_myoepithelial | H3K4me3 | 6.68 | 7.06 | 0.416236897 | Ever-smoking lung cancer | Other |
| Fetal_lung | H3K9ac | 7.80 | 8.66 | 0.425393377 | Ever-smoking lung cancer | Cardiovascular |
| Fetal_large_intestine | H3K4me1 | 4.20 | 4.54 | 0.427955251 | Ever-smoking lung cancer | GI |
| Liver | H3K27ac | -1.24 | 2.86 | 0.438783298 | Ever-smoking lung cancer | Liver |
| Stomach_smooth_muscle | H3K9ac | 5.94 | 6.44 | 0.44146714 | Ever-smoking lung cancer | GI |
| Colon_smooth_muscle | H3K4me1 | 3.50 | 3.33 | 0.45112265 | Ever-smoking lung cancer | GI |
| Breast_myoepithelial | H3K4me1 | -0.71 | 2.28 | 0.454095342 | Ever-smoking lung cancer | Other |
| Fetal_heart | H3K4me3 | 11.17 | 14.09 | 0.464877604 | Ever-smoking lung cancer | Cardiovascular |
| Fetal_adrenal | H3K4me1 | 3.90 | 4.03 | 0.468322059 | Ever-smoking lung cancer | Adrenal_Pancreas |
| Breast_vHMEC | H3K4me1 | -1.32 | 3.28 | 0.483739089 | Ever-smoking lung cancer | Other |
| Fetal_trunk_muscle | H3K4me1 | 2.96 | 2.76 | 0.48445661 | Ever-smoking lung cancer | SkeletalMuscle |
| Breast_luminal_epithelial | H3K4me1 | -26.36 | 38.93 | 0.484921149 | Ever-smoking lung cancer | Other |
| Pancreatic_islets | H3K27ac | 2.96 | 2.91 | 0.488598543 | Ever-smoking lung cancer | Adrenal_Pancreas |
| Stomach_smooth_muscle | H3K27ac | 2.95 | 2.88 | 0.498881996 | Ever-smoking lung cancer | GI |
| Fetal_small_intestine | H3K4me1 | 3.60 | 4.51 | 0.523674668 | Ever-smoking lung cancer | GI |
| Fetal_heart | H3K4me1 | 2.36 | 2.20 | 0.536414125 | Ever-smoking lung cancer | Cardiovascular |
| Penis_foreskin_keratinocyte_primary | H3K4me3 | 4.52 | 5.84 | 0.550366881 | Ever-smoking lung cancer | Other |
| CD19 | H3K27ac | 2.42 | 2.39 | 0.552021824 | Ever-smoking lung cancer | Hematopoietic |
| Ovary | H3K4me1 | -3.99 | 9.00 | 0.560436127 | Ever-smoking lung cancer | Other |
| Inferior_temporal_lobe | H3K4me1 | 2.32 | 2.24 | 0.56098148 | Ever-smoking lung cancer | CNS |
| Left_Ventricle | H3K4me1 | -1.43 | 5.01 | 0.56834539 | Ever-smoking lung cancer | Cardiovascular |
| Pancreatic_islets | H3K9ac | 8.51 | 13.72 | 0.581441544 | Ever-smoking lung cancer | Adrenal_Pancreas |
| Osteoblast | H3K27ac | -0.33 | 2.54 | 0.587864158 | Ever-smoking lung cancer | Connective_Bone |
| Breast_myoepithelial | H3K9ac | 11.66 | 20.29 | 0.593846997 | Ever-smoking lung cancer | Other |
| Substantia_nigra | H3K4me1 | 2.28 | 2.47 | 0.607929878 | Ever-smoking lung cancer | CNS |
| Cingulate_gyrus | H3K4me1 | 2.15 | 2.44 | 0.638875442 | Ever-smoking lung cancer | CNS |
| Spleen | H3K4me1 | 2.71 | 3.87 | 0.645509293 | Ever-smoking lung cancer | Hematopoietic |
| Duodenum_mucosa | H3K27ac | 2.98 | 5.10 | 0.676426537 | Ever-smoking lung cancer | GI |
| Gastric | H3K4me1 | 4.82 | 9.36 | 0.685277306 | Ever-smoking lung cancer | GI |
| Pancreatic_islets | H3K4me3 | -2.31 | 8.88 | 0.688698959 | Ever-smoking lung cancer | Adrenal_Pancreas |
| Rectal_mucosa | H3K4me1 | 2.05 | 2.72 | 0.698140906 | Ever-smoking lung cancer | GI |
| Liver_(UCSD) | H3K4me3 | 4.35 | 8.82 | 0.702374838 | Ever-smoking lung cancer | Liver |
| Rectal_mucosa | H3K27ac | 2.24 | 3.42 | 0.70472679 | Ever-smoking lung cancer | GI |
| Hippocampus_middle | H3K4me1 | 1.79 | 2.11 | 0.709236287 | Ever-smoking lung cancer | CNS |
| Kidney | H3K9ac | 3.44 | 7.50 | 0.743405708 | Ever-smoking lung cancer | Kidney |
| Colonic_mucosa | H3K4me1 | -0.22 | 3.78 | 0.746322159 | Ever-smoking lung cancer | GI |
| Placenta_amnion | H3K4me1 | -3.64 | 15.17 | 0.759604685 | Ever-smoking lung cancer | Other |
| Spleen | H3K4me3 | 7.58 | 22.37 | 0.762648327 | Ever-smoking lung cancer | Hematopoietic |
| Liver_(BI) | H3K4me1 | 1.59 | 2.02 | 0.76991564 | Ever-smoking lung cancer | Liver |
| Stomach_smooth_muscle | H3K4me1 | 2.26 | 4.65 | 0.778940882 | Ever-smoking lung cancer | GI |
| Adipose_nuclei | H3K27ac | 0.25 | 2.89 | 0.780098223 | Ever-smoking lung cancer | Other |
| Penis_foreskin_melanocyte_primary | H3K4me1 | 0.54 | 1.84 | 0.794957506 | Ever-smoking lung cancer | Other |
| Anterior_caudate | H3K4me1 | 1.58 | 2.39 | 0.806745651 | Ever-smoking lung cancer | CNS |
| Pancreatic_islets | H3K4me3 | -1.01 | 8.62 | 0.808445017 | Ever-smoking lung cancer | Adrenal_Pancreas |
| Pancreatic_islets | H3K4me1 | 3.28 | 9.58 | 0.810488089 | Ever-smoking lung cancer | Adrenal_Pancreas |
| Right_ventricle | H3K4me1 | 10.98 | 45.12 | 0.81684492 | Ever-smoking lung cancer | Cardiovascular |
| Pancreas | H3K4me3 | 3.16 | 10.91 | 0.842284298 | Ever-smoking lung cancer | Adrenal_Pancreas |
| Stomach_mucosa | H3K4me1 | 0.42 | 3.12 | 0.851016337 | Ever-smoking lung cancer | GI |
| Mid_frontal_lobe | H3K4me1 | 3.29 | 15.09 | 0.875949122 | Ever-smoking lung cancer | CNS |
| Fetal_placenta | H3K4me1 | 0.49 | 3.51 | 0.885759257 | Ever-smoking lung cancer | Other |
| Penis_foreskin_fibroblast_primary | H3K4me1 | 1.20 | 1.61 | 0.898898766 | Ever-smoking lung cancer | Connective_Bone |
| Breast_vHMEC | H3K4me3 | 2.29 | 11.24 | 0.904188031 | Ever-smoking lung cancer | Other |
| Rectal_smooth_muscle | H3K4me1 | 1.74 | 6.86 | 0.914052371 | Ever-smoking lung cancer | GI |
| Liver_(BI) | H3K9ac | 1.61 | 6.02 | 0.919252759 | Ever-smoking lung cancer | Liver |
| Duodenum_smooth_muscle | H3K4me1 | 1.98 | 12.65 | 0.936857936 | Ever-smoking lung cancer | GI |
| Esophagus | H3K4me1 | 1.39 | 9.51 | 0.967450917 | Ever-smoking lung cancer | GI |
| Fetal_leg_muscle | H3K4me1 | 1.10 | 2.96 | 0.970588335 | Ever-smoking lung cancer | SkeletalMuscle |
| Penis_foreskin_keratinocyte_primary | H3K4me1 | 1.06 | 2.56 | 0.982091073 | Ever-smoking lung cancer | Other |
| CD14 | H3K27ac | 8.56 | 2.10 | 0.000696419 | Lung adenocarcinoma | Hematopoietic |
| CD15_primary | H3K4me1 | 7.20 | 2.29 | 0.006542077 | Lung adenocarcinoma | Hematopoietic |
| CD56_primary | H3K4me1 | 7.53 | 2.27 | 0.006832337 | Lung adenocarcinoma | Hematopoietic |
| CD14_primary | H3K4me1 | 7.14 | 2.33 | 0.006861181 | Lung adenocarcinoma | Hematopoietic |
| CD4_memory_primary | H3K4me1 | 6.47 | 2.01 | 0.01104447 | Lung adenocarcinoma | Hematopoietic |
| CD4+_CD25-_CD45R0+_memory_primary | H3K4me1 | 7.40 | 2.40 | 0.011277054 | Lung adenocarcinoma | Hematopoietic |
| CD3_primary_(BI) | H3K4me1 | 7.11 | 2.31 | 0.011669208 | Lung adenocarcinoma | Hematopoietic |
| CD19_primary_(BI) | H3K4me1 | 7.33 | 2.38 | 0.012810412 | Lung adenocarcinoma | Hematopoietic |
| CD8_naive_primary_(UCSF-UBC) | H3K4me1 | 8.77 | 2.87 | 0.013055292 | Lung adenocarcinoma | Hematopoietic |
| CD4+_CD25+_CD127-_Treg_primary | H3K4me1 | 7.92 | 2.70 | 0.013403054 | Lung adenocarcinoma | Hematopoietic |
| CD4+_CD25-_IL17-_PMA_Ionomycin_stim_MACS_Th_sprimary | H3K4me1 | 5.88 | 1.79 | 0.013951188 | Lung adenocarcinoma | Hematopoietic |
| CD8_memory_primary | H3K4me1 | 6.79 | 2.33 | 0.015891676 | Lung adenocarcinoma | Hematopoietic |
| CD3_primary | H3K27ac | 6.82 | 2.57 | 0.018630679 | Lung adenocarcinoma | Hematopoietic |
| CD4+_CD25-_Th_primary | H3K4me1 | 6.64 | 2.17 | 0.019260548 | Lung adenocarcinoma | Hematopoietic |
| CD4_naive_primary | H3K4me1 | 6.69 | 2.32 | 0.019840413 | Lung adenocarcinoma | Hematopoietic |
| CD4+_CD25-_IL17+_PMA_Ionomycin_stim_Th17_primary | H3K4me1 | 7.03 | 2.46 | 0.022535671 | Lung adenocarcinoma | Hematopoietic |
| CD25-_IL17+_Th17_stim | H3K27ac | 10.90 | 4.01 | 0.024501246 | Lung adenocarcinoma | Hematopoietic |
| CD4+_CD25int_CD127+_Tmem_primary | H3K4me1 | 10.31 | 4.31 | 0.02872057 | Lung adenocarcinoma | Hematopoietic |
| CD8_naive_primary_(BI) | H3K4me1 | 6.58 | 2.41 | 0.032050862 | Lung adenocarcinoma | Hematopoietic |
| CD3_primary_(UW) | H3K4me1 | 6.31 | 2.31 | 0.034259614 | Lung adenocarcinoma | Hematopoietic |
| CD15_primary | H3K4me3 | 15.38 | 6.77 | 0.038017973 | Lung adenocarcinoma | Hematopoietic |
| CD56_primary | H3K4me3 | 25.27 | 11.05 | 0.038439143 | Lung adenocarcinoma | Hematopoietic |
| Right_ventricle | H3K4me1 | 63.74 | 28.93 | 0.040572277 | Lung adenocarcinoma | Cardiovascular |
| CD4+_CD25-_CD45RA+_naive_primary | H3K4me1 | 5.55 | 2.08 | 0.045254535 | Lung adenocarcinoma | Hematopoietic |
| CD19_primary_(UW) | H3K4me1 | 6.06 | 2.34 | 0.048452996 | Lung adenocarcinoma | Hematopoietic |
| Treg_primary | H3K4me3 | 9.36 | 4.10 | 0.053883257 | Lung adenocarcinoma | Hematopoietic |
| Peripheralblood_mononuclear_primary | H3K9ac | 16.00 | 7.67 | 0.062036477 | Lung adenocarcinoma | Hematopoietic |
| CD4+_CD25-_IL17+_PMA_Ionomycin_stim_Th17_primary | H3K4me3 | 15.69 | 7.61 | 0.066034651 | Lung adenocarcinoma | Hematopoietic |
| CD25-_IL17-_Th_stim_MACS | H3K27ac | 6.55 | 2.93 | 0.069053738 | Lung adenocarcinoma | Hematopoietic |
| Osteoblast | H3K27ac | 5.09 | 2.28 | 0.07421595 | Lung adenocarcinoma | Connective_Bone |
| Liver_(BI) | H3K4me1 | 3.93 | 1.65 | 0.076877522 | Lung adenocarcinoma | Liver |
| CD25+_CD127-_Treg | H3K27ac | 5.09 | 2.31 | 0.077064152 | Lung adenocarcinoma | Hematopoietic |
| Th2 | H3K27ac | 7.46 | 3.66 | 0.079247992 | Lung adenocarcinoma | Hematopoietic |
| Th1 | H3K27ac | 6.20 | 2.80 | 0.081225383 | Lung adenocarcinoma | Hematopoietic |
| Mobilized_CD34_primary | H3K4me3 | 10.42 | 5.06 | 0.082065379 | Lung adenocarcinoma | Hematopoietic |
| Mobilized_CD34_primary | H3K4me1 | 4.17 | 1.70 | 0.086583585 | Lung adenocarcinoma | Hematopoietic |
| Th0 | H3K27ac | 6.36 | 3.07 | 0.087527203 | Lung adenocarcinoma | Hematopoietic |
| CD19_primary_(BI) | H3K4me3 | 17.87 | 9.69 | 0.094864835 | Lung adenocarcinoma | Hematopoietic |
| CD8_primary | H3K4me3 | 15.87 | 8.54 | 0.096600189 | Lung adenocarcinoma | Hematopoietic |
| CD34_primary | H3K4me1 | 5.98 | 3.03 | 0.116130452 | Lung adenocarcinoma | Hematopoietic |
| CD25int_CD127+_Tmem | H3K27ac | 5.05 | 2.70 | 0.117503772 | Lung adenocarcinoma | Hematopoietic |
| CD34_primary | H3K4me3 | 17.51 | 10.13 | 0.121599022 | Lung adenocarcinoma | Hematopoietic |
| CD4+_CD25+_CD127-_Treg_primary | H3K4me3 | 12.25 | 6.96 | 0.124011085 | Lung adenocarcinoma | Hematopoietic |
| CD4+_CD25-_IL17-_PMA_Ionomycin_stim_MACS_Th_sprimary | H3K4me3 | 9.94 | 5.53 | 0.126272625 | Lung adenocarcinoma | Hematopoietic |
| Peripheralblood_mononuclear_primary | H3K4me3 | 17.80 | 10.65 | 0.130340116 | Lung adenocarcinoma | Hematopoietic |
| Mobilized_CD34 | H3K27ac | 4.62 | 2.40 | 0.137108377 | Lung adenocarcinoma | Hematopoietic |
| CD8_memory_primary | H3K4me3 | 11.60 | 6.97 | 0.142637215 | Lung adenocarcinoma | Hematopoietic |
| Duodenum_Mucosa | H3K4me3 | 7.73 | 4.72 | 0.146578039 | Lung adenocarcinoma | GI |
| Peripheralblood_mononuclear_primary | H3K4me1 | 13.95 | 8.73 | 0.149268221 | Lung adenocarcinoma | Hematopoietic |
| Fetal_lung | H3K4me1 | 3.85 | 1.92 | 0.158203826 | Lung adenocarcinoma | Cardiovascular |
| CD8_naive_primary_(BI) | H3K4me3 | 12.48 | 7.90 | 0.161080999 | Lung adenocarcinoma | Hematopoietic |
| Penis_foreskin_fibroblast_primary | H3K4me3 | 4.19 | 2.33 | 0.164622478 | Lung adenocarcinoma | Connective_Bone |
| Fetal_large_intestine | H3K4me3 | 12.06 | 7.98 | 0.176235588 | Lung adenocarcinoma | GI |
| CD4+_CD25-_Th_primary | H3K4me3 | 12.84 | 8.47 | 0.177552885 | Lung adenocarcinoma | Hematopoietic |
| Rectal_smooth_muscle | H3K4me3 | 10.94 | 7.29 | 0.180087617 | Lung adenocarcinoma | GI |
| Liver_(UCSD) | H3K4me1 | 36.67 | 31.40 | 0.185609018 | Lung adenocarcinoma | Liver |
| Colonic_mucosa | H3K27ac | 5.28 | 3.68 | 0.188636387 | Lung adenocarcinoma | GI |
| CD4+_CD25-_CD45RA+_naive_primary | H3K4me3 | 10.82 | 7.40 | 0.196314561 | Lung adenocarcinoma | Hematopoietic |
| CD19 | H3K27ac | 3.71 | 2.09 | 0.198562481 | Lung adenocarcinoma | Hematopoietic |
| CD4_naive_primary | H3K4me3 | 10.68 | 7.33 | 0.19960187 | Lung adenocarcinoma | Hematopoietic |
| Penis_foreskin_keratinocyte_primary | H3K9ac | 4.25 | 2.59 | 0.200840413 | Lung adenocarcinoma | Other |
| Rectal_mucosa | H3K9ac | 7.84 | 5.41 | 0.201798521 | Lung adenocarcinoma | GI |
| CD3_primary_(BI) | H3K4me3 | 10.54 | 7.39 | 0.204944909 | Lung adenocarcinoma | Hematopoietic |
| CD3_primary_(UW) | H3K4me3 | 11.80 | 8.32 | 0.206169461 | Lung adenocarcinoma | Hematopoietic |
| Rectal_mucosa | H3K4me1 | 4.41 | 2.78 | 0.208039418 | Lung adenocarcinoma | GI |
| Esophagus | H3K4me1 | 11.12 | 8.65 | 0.210494226 | Lung adenocarcinoma | GI |
| Skeletal_muscle | H3K9ac | 5.19 | 3.33 | 0.217379291 | Lung adenocarcinoma | SkeletalMuscle |
| Adipose_nuclei | H3K4me1 | 3.71 | 2.11 | 0.221607457 | Lung adenocarcinoma | Other |
| Adipose_nuclei | H3K27ac | 3.87 | 2.21 | 0.221629378 | Lung adenocarcinoma | Other |
| Gastric | H3K4me1 | 7.98 | 6.00 | 0.23541774 | Lung adenocarcinoma | GI |
| Fetal_heart | H3K9ac | 6.11 | 4.20 | 0.236433158 | Lung adenocarcinoma | Cardiovascular |
| CD19_primary_(UW) | H3K4me3 | 14.96 | 11.69 | 0.237218382 | Lung adenocarcinoma | Hematopoietic |
| Skeletal_muscle | H3K4me1 | 3.10 | 1.73 | 0.242839343 | Lung adenocarcinoma | SkeletalMuscle |
| Liver | H3K27ac | 3.81 | 2.63 | 0.243333103 | Lung adenocarcinoma | Liver |
| CD4_primary | H3K4me3 | 9.89 | 7.43 | 0.245449665 | Lung adenocarcinoma | Hematopoietic |
| Mid_frontal_lobe | H3K9ac | -4.26 | 4.42 | 0.248755122 | Lung adenocarcinoma | CNS |
| Colon_smooth_muscle | H3K27ac | 3.65 | 2.17 | 0.249201242 | Lung adenocarcinoma | GI |
| Right_ventricle | H3K4me3 | 12.38 | 9.91 | 0.25070091 | Lung adenocarcinoma | Cardiovascular |
| Placenta_chorion | H3K4me1 | -6.70 | 6.52 | 0.253704346 | Lung adenocarcinoma | Other |
| Rectal_mucosa | H3K4me3 | 7.27 | 5.54 | 0.257367283 | Lung adenocarcinoma | GI |
| CD4+_CD25-_CD45R0+_memory_primary | H3K4me3 | 12.35 | 9.75 | 0.257960389 | Lung adenocarcinoma | Hematopoietic |
| Adipose_nuclei | H3K9ac | 6.48 | 4.74 | 0.258015998 | Lung adenocarcinoma | Other |
| Liver_(BI) | H3K9ac | 6.97 | 5.50 | 0.274035608 | Lung adenocarcinoma | Liver |
| CD20 | H3K27ac | 3.06 | 1.86 | 0.276056712 | Lung adenocarcinoma | Hematopoietic |
| Skeletal_muscle | H3K27ac | 3.66 | 2.50 | 0.281743475 | Lung adenocarcinoma | SkeletalMuscle |
| CD14_primary | H3K4me3 | 11.80 | 9.89 | 0.286234412 | Lung adenocarcinoma | Hematopoietic |
| Colonic_mucosa | H3K9ac | 5.73 | 4.55 | 0.289769167 | Lung adenocarcinoma | GI |
| Colon_smooth_muscle | H3K4me1 | 3.97 | 2.78 | 0.29791223 | Lung adenocarcinoma | GI |
| CD4+_CD25int_CD127+_Tmem_primary | H3K4me3 | 12.38 | 10.92 | 0.303624778 | Lung adenocarcinoma | Hematopoietic |
| CD8_naive_primary_(UCSF-UBC) | H3K4me3 | 15.14 | 13.92 | 0.316566015 | Lung adenocarcinoma | Hematopoietic |
| Stomach_mucosa | H3K4me1 | 3.63 | 2.88 | 0.326349678 | Lung adenocarcinoma | GI |
| Penis_foreskin_keratinocyte_primary | H3K4me1 | 3.44 | 2.46 | 0.32684815 | Lung adenocarcinoma | Other |
| Duodenum_Mucosa | H3K4me1 | 3.13 | 2.32 | 0.332264665 | Lung adenocarcinoma | GI |
| Rectal_smooth_muscle | H3K27ac | 3.11 | 2.11 | 0.338067537 | Lung adenocarcinoma | GI |
| CD4_memory_primary | H3K4me3 | 8.13 | 7.49 | 0.345485296 | Lung adenocarcinoma | Hematopoietic |
| Colonic_mucosa | H3K4me3 | 8.32 | 7.83 | 0.345616719 | Lung adenocarcinoma | GI |
| Chondrogenic_dif | H3K27ac | 3.77 | 2.91 | 0.348473095 | Lung adenocarcinoma | Connective_Bone |
| Fetal_heart | H3K4me1 | 2.95 | 2.04 | 0.352838002 | Lung adenocarcinoma | Cardiovascular |
| CD25-_CD45RA+_naive | H3K27ac | 2.95 | 2.20 | 0.378244294 | Lung adenocarcinoma | Hematopoietic |
| Fetal_large_intestine | H3K4me1 | 3.90 | 3.62 | 0.38031077 | Lung adenocarcinoma | GI |
| Fetal_stomach | H3K4me3 | 9.53 | 9.69 | 0.38504375 | Lung adenocarcinoma | GI |
| Substantia_nigra | H3K27ac | 2.65 | 1.98 | 0.393626595 | Lung adenocarcinoma | CNS |
| Breast_myoepithelial | H3K4me3 | 6.57 | 6.59 | 0.393985939 | Lung adenocarcinoma | Other |
| Left_Ventricle | H3K4me3 | 11.16 | 12.08 | 0.394608314 | Lung adenocarcinoma | Cardiovascular |
| Anterior_caudate | H3K27ac | 3.00 | 2.50 | 0.39525478 | Lung adenocarcinoma | CNS |
| Penis_foreskin_keratinocyte_primary | H3K4me3 | 5.24 | 5.14 | 0.411150816 | Lung adenocarcinoma | Other |
| Placenta_chorion | H3K4me3 | 8.64 | 9.48 | 0.415232734 | Lung adenocarcinoma | Other |
| Breast_myoepithelial | H3K9ac | 15.68 | 18.55 | 0.425023656 | Lung adenocarcinoma | Other |
| Mid_frontal_lobe | H3K4me1 | -7.76 | 10.84 | 0.432588774 | Lung adenocarcinoma | CNS |
| Stomach_smooth_muscle | H3K27ac | 2.74 | 2.15 | 0.43312044 | Lung adenocarcinoma | GI |
| Thymus | H3K4me1 | 6.98 | 7.58 | 0.437402061 | Lung adenocarcinoma | Hematopoietic |
| Fetal_small_intestine | H3K4me3 | 7.75 | 8.64 | 0.437834312 | Lung adenocarcinoma | GI |
| Angular_gyrus | H3K9ac | -3.60 | 6.01 | 0.438563031 | Lung adenocarcinoma | CNS |
| Ovary | H3K4me1 | -4.25 | 7.35 | 0.445238999 | Lung adenocarcinoma | Other |
| Fetal_thymus | H3K4me1 | 2.63 | 2.11 | 0.451667308 | Lung adenocarcinoma | Hematopoietic |
| Rectal_mucosa | H3K27ac | 3.39 | 3.36 | 0.452818216 | Lung adenocarcinoma | GI |
| Skeletal_muscle | H3K4me3 | 4.35 | 4.51 | 0.456852392 | Lung adenocarcinoma | SkeletalMuscle |
| Fetal_adrenal | H3K4me3 | 9.55 | 11.68 | 0.459611688 | Lung adenocarcinoma | Adrenal_Pancreas |
| Lung | H3K4me1 | 3.83 | 3.86 | 0.466282459 | Lung adenocarcinoma | Cardiovascular |
| Right_atrium | H3K4me3 | 8.26 | 10.08 | 0.466748615 | Lung adenocarcinoma | Cardiovascular |
| Duodenum_Mucosa | H3K9ac | 4.67 | 5.19 | 0.476065403 | Lung adenocarcinoma | GI |
| Germinal_matrix | H3K4me3 | -4.28 | 8.06 | 0.4852365 | Lung adenocarcinoma | CNS |
| Kidney | H3K4me3 | 4.59 | 5.25 | 0.492229162 | Lung adenocarcinoma | Kidney |
| Duodenum_smooth_muscle | H3K4me3 | 4.19 | 4.72 | 0.492491822 | Lung adenocarcinoma | GI |
| Liver_(UCSD) | H3K4me3 | 6.84 | 8.59 | 0.494849194 | Lung adenocarcinoma | Liver |
| Fetal_lung | H3K4me3 | 6.85 | 8.79 | 0.501841892 | Lung adenocarcinoma | Cardiovascular |
| Fetal_leg_muscle | H3K4me3 | 6.98 | 9.29 | 0.518783594 | Lung adenocarcinoma | SkeletalMuscle |
| Pancreatic_islets | H3K9ac | -6.83 | 12.96 | 0.52019711 | Lung adenocarcinoma | Adrenal_Pancreas |
| Fetal_thymus | H3K4me3 | 8.99 | 12.92 | 0.525711044 | Lung adenocarcinoma | Hematopoietic |
| Duodenum_smooth_muscle | H3K27ac | 2.89 | 2.98 | 0.530200092 | Lung adenocarcinoma | GI |
| Psoas_muscle | H3K4me1 | -5.48 | 10.40 | 0.540602647 | Lung adenocarcinoma | SkeletalMuscle |
| CD8_naive_primary_(UCSF-UBC) | H3K9ac | 21.51 | 34.43 | 0.542640737 | Lung adenocarcinoma | Hematopoietic |
| Stomach_smooth_muscle | H3K4me3 | 4.26 | 5.40 | 0.543741113 | Lung adenocarcinoma | GI |
| Angular_gyrus | H3K27ac | -0.41 | 2.31 | 0.548576552 | Lung adenocarcinoma | CNS |
| Stomach_mucosa | H3K9ac | 4.47 | 6.05 | 0.566398774 | Lung adenocarcinoma | GI |
| Colon_smooth_muscle | H3K9ac | -4.97 | 11.11 | 0.566989068 | Lung adenocarcinoma | GI |
| Pancreas | H3K4me1 | 2.42 | 2.53 | 0.572752621 | Lung adenocarcinoma | Adrenal_Pancreas |
| Adipose_nuclei | H3K4me3 | 3.03 | 3.69 | 0.574015601 | Lung adenocarcinoma | Other |
| Mid_frontal_lobe | H3K27ac | -0.72 | 3.08 | 0.57632914 | Lung adenocarcinoma | CNS |
| Right_atrium | H3K4me1 | -2.47 | 6.47 | 0.577196826 | Lung adenocarcinoma | Cardiovascular |
| Cingulate_gyrus | H3K4me3 | 4.15 | 5.71 | 0.579870917 | Lung adenocarcinoma | CNS |
| Left_Ventricle | H3K4me1 | 2.89 | 3.72 | 0.60336024 | Lung adenocarcinoma | Cardiovascular |
| Rectal_smooth_muscle | H3K4me1 | -1.38 | 4.72 | 0.604074864 | Lung adenocarcinoma | GI |
| Esophagus | H3K4me3 | 6.02 | 9.77 | 0.60483807 | Lung adenocarcinoma | GI |
| Kidney | H3K9ac | 4.45 | 6.98 | 0.61864064 | Lung adenocarcinoma | Kidney |
| Cingulate_gyrus | H3K4me1 | 1.98 | 1.96 | 0.618959505 | Lung adenocarcinoma | CNS |
| Anterior_caudate | H3K4me3 | -1.54 | 5.21 | 0.620930095 | Lung adenocarcinoma | CNS |
| Fetal_brain | H3K4me3 | -9.34 | 24.43 | 0.628366015 | Lung adenocarcinoma | CNS |
| Angular_gyrus | H3K4me3 | -2.93 | 8.37 | 0.632904098 | Lung adenocarcinoma | CNS |
| Gastric | H3K4me3 | 8.98 | 17.43 | 0.636956557 | Lung adenocarcinoma | GI |
| Fetal_placenta | H3K4me3 | 5.78 | 10.30 | 0.637527675 | Lung adenocarcinoma | Other |
| Fetal_brain | H3K4me3 | -2.12 | 6.83 | 0.638657388 | Lung adenocarcinoma | CNS |
| Substantia_nigra | H3K4me1 | 1.88 | 2.02 | 0.660611944 | Lung adenocarcinoma | CNS |
| Penis_foreskin_fibroblast_primary | H3K4me1 | 1.64 | 1.47 | 0.665109258 | Lung adenocarcinoma | Connective_Bone |
| Placenta_amnion | H3K4me3 | 6.62 | 13.20 | 0.665651923 | Lung adenocarcinoma | Other |
| Breast_vHMEC | H3K4me3 | 5.64 | 11.09 | 0.672528796 | Lung adenocarcinoma | Other |
| Hippocampus_middle | H3K9ac | 2.63 | 4.04 | 0.684839087 | Lung adenocarcinoma | CNS |
| Colon_smooth_muscle | H3K4me3 | 3.57 | 6.67 | 0.694827009 | Lung adenocarcinoma | GI |
| Fetal_trunk_muscle | H3K4me1 | 1.94 | 2.43 | 0.69524418 | Lung adenocarcinoma | SkeletalMuscle |
| Breast_fibroblast_primary | H3K4me1 | 3.97 | 7.65 | 0.697068184 | Lung adenocarcinoma | Connective_Bone |
| Fetal_trunk_muscle | H3K4me3 | 4.90 | 10.17 | 0.697098192 | Lung adenocarcinoma | SkeletalMuscle |
| Angular_gyrus | H3K4me1 | 2.60 | 4.35 | 0.704139133 | Lung adenocarcinoma | CNS |
| Small_intestine | H3K4me3 | 7.73 | 18.44 | 0.70769201 | Lung adenocarcinoma | GI |
| Fetal_kidney | H3K9ac | 8.31 | 20.38 | 0.708716807 | Lung adenocarcinoma | Kidney |
| Inferior_temporal_lobe | H3K27ac | 0.23 | 2.09 | 0.711088401 | Lung adenocarcinoma | CNS |
| Spleen | H3K4me1 | 2.13 | 3.20 | 0.711888672 | Lung adenocarcinoma | Hematopoietic |
| Pancreatic_islets | H3K4me3 | -1.90 | 8.42 | 0.722636882 | Lung adenocarcinoma | Adrenal_Pancreas |
| Inferior_temporal_lobe | H3K4me3 | 2.97 | 5.61 | 0.723722109 | Lung adenocarcinoma | CNS |
| Lung | H3K4me3 | 5.57 | 13.36 | 0.726653241 | Lung adenocarcinoma | Cardiovascular |
| Liver_(BI) | H3K4me3 | 2.76 | 5.19 | 0.728510044 | Lung adenocarcinoma | Liver |
| Fetal_small_intestine | H3K4me1 | 2.09 | 3.36 | 0.730100966 | Lung adenocarcinoma | GI |
| Fetal_placenta | H3K4me1 | 0.02 | 2.85 | 0.732205856 | Lung adenocarcinoma | Other |
| Fetal_stomach | H3K4me1 | 0.01 | 3.12 | 0.738762033 | Lung adenocarcinoma | GI |
| Pancreatic_islets | H3K4me1 | -1.51 | 7.50 | 0.740360307 | Lung adenocarcinoma | Adrenal_Pancreas |
| Fetal_lung | H3K9ac | 3.88 | 9.02 | 0.74249612 | Lung adenocarcinoma | Cardiovascular |
| Fetal_brain | H3K9ac | -0.85 | 6.39 | 0.765096442 | Lung adenocarcinoma | CNS |
| Sigmoid_colon | H3K4me3 | 6.10 | 18.31 | 0.771111267 | Lung adenocarcinoma | GI |
| Breast_fibroblast_primary | H3K4me3 | 5.70 | 16.95 | 0.773802324 | Lung adenocarcinoma | Connective_Bone |
| Breast_vHMEC | H3K4me1 | 2.05 | 3.70 | 0.775584757 | Lung adenocarcinoma | Other |
| Inferior_temporal_lobe | H3K9ac | 2.11 | 4.24 | 0.791663495 | Lung adenocarcinoma | CNS |
| Duodenum_smooth_muscle | H3K4me1 | -1.41 | 9.78 | 0.805739871 | Lung adenocarcinoma | GI |
| Spleen | H3K4me3 | 5.67 | 20.22 | 0.809332317 | Lung adenocarcinoma | Hematopoietic |
| Anterior_caudate | H3K4me1 | 1.52 | 2.16 | 0.809907876 | Lung adenocarcinoma | CNS |
| Placenta_amnion | H3K4me1 | -2.00 | 12.68 | 0.810547255 | Lung adenocarcinoma | Other |
| Aorta | H3K4me3 | -1.83 | 12.44 | 0.81128129 | Lung adenocarcinoma | Cardiovascular |
| Stomach_smooth_muscle | H3K4me1 | 1.84 | 3.67 | 0.811563562 | Lung adenocarcinoma | GI |
| Sigmoid_colon | H3K4me1 | -2.76 | 15.67 | 0.812851265 | Lung adenocarcinoma | GI |
| Fetal_adrenal | H3K4me1 | 0.28 | 3.35 | 0.814122817 | Lung adenocarcinoma | Adrenal_Pancreas |
| Stomach_smooth_muscle | H3K9ac | 2.24 | 5.77 | 0.826093892 | Lung adenocarcinoma | GI |
| Psoas_muscle | H3K4me3 | 3.06 | 10.32 | 0.838290317 | Lung adenocarcinoma | SkeletalMuscle |
| Ovary | H3K4me3 | -1.22 | 11.70 | 0.839097115 | Lung adenocarcinoma | Other |
| Small_intestine | H3K4me1 | -4.88 | 29.47 | 0.843228455 | Lung adenocarcinoma | GI |
| Kidney | H3K27ac | 1.44 | 2.32 | 0.846003142 | Lung adenocarcinoma | Kidney |
| Penis_foreskin_melanocyte_primary | H3K4me3 | 1.86 | 4.68 | 0.851934755 | Lung adenocarcinoma | Other |
| Anterior_caudate | H3K9ac | 0.10 | 4.87 | 0.853083599 | Lung adenocarcinoma | CNS |
| Rectal_smooth_muscle | H3K9ac | 4.35 | 19.31 | 0.857737571 | Lung adenocarcinoma | GI |
| Pancreas | H3K4me3 | -0.72 | 10.37 | 0.863427333 | Lung adenocarcinoma | Adrenal_Pancreas |
| Stomach_mucosa | H3K4me3 | 3.44 | 15.26 | 0.867204132 | Lung adenocarcinoma | GI |
| Hippocampus_middle | H3K27ac | 0.58 | 2.54 | 0.867632242 | Lung adenocarcinoma | CNS |
| Colonic_mucosa | H3K4me1 | 1.49 | 3.82 | 0.895412936 | Lung adenocarcinoma | GI |
| Duodenum_mucosa | H3K27ac | 0.47 | 4.22 | 0.89700503 | Lung adenocarcinoma | GI |
| Substantia_nigra | H3K4me3 | 0.20 | 6.94 | 0.906072901 | Lung adenocarcinoma | CNS |
| Pancreatic_islets | H3K4me1 | 1.68 | 5.89 | 0.906195101 | Lung adenocarcinoma | Adrenal_Pancreas |
| Pancreatic_islets | H3K4me3 | 0.08 | 8.36 | 0.910127631 | Lung adenocarcinoma | Adrenal_Pancreas |
| Fetal_heart | H3K4me3 | -0.40 | 13.33 | 0.911652122 | Lung adenocarcinoma | Cardiovascular |
| Mid_frontal_lobe | H3K4me3 | 0.39 | 5.58 | 0.9120988 | Lung adenocarcinoma | CNS |
| Pancreatic_islets | H3K27ac | 1.23 | 2.09 | 0.91284315 | Lung adenocarcinoma | Adrenal_Pancreas |
| Breast_myoepithelial | H3K4me1 | 1.16 | 1.68 | 0.923960578 | Lung adenocarcinoma | Other |
| Penis_foreskin_melanocyte_primary | H3K4me1 | 0.87 | 1.51 | 0.929015473 | Lung adenocarcinoma | Other |
| Substantia_nigra | H3K9ac | 1.35 | 4.22 | 0.933255747 | Lung adenocarcinoma | CNS |
| Fetal_leg_muscle | H3K4me1 | 0.81 | 2.46 | 0.935464204 | Lung adenocarcinoma | SkeletalMuscle |
| Fetal_brain | H3K4me1 | 0.88 | 1.63 | 0.939979724 | Lung adenocarcinoma | CNS |
| Cingulate_gyrus | H3K27ac | 1.14 | 1.98 | 0.943840226 | Lung adenocarcinoma | CNS |
| Cingulate_gyrus | H3K9ac | 1.25 | 3.60 | 0.944810493 | Lung adenocarcinoma | CNS |
| Neurosphere | H3K27ac | 1.13 | 2.14 | 0.949969701 | Lung adenocarcinoma | CNS |
| Hippocampus_middle | H3K4me1 | 0.91 | 1.80 | 0.96067718 | Lung adenocarcinoma | CNS |
| Kidney | H3K4me1 | 0.77 | 5.83 | 0.969292796 | Lung adenocarcinoma | Kidney |
| Inferior_temporal_lobe | H3K4me1 | 1.06 | 1.76 | 0.972111057 | Lung adenocarcinoma | CNS |
| Breast_luminal_epithelial | H3K4me1 | 1.93 | 28.67 | 0.972481347 | Lung adenocarcinoma | Other |
| Hippocampus_middle | H3K4me3 | 0.90 | 5.32 | 0.984417697 | Lung adenocarcinoma | CNS |
| Right_ventricle | H3K4me3 | 29.01 | 11.21 | 0.009086903 | Lung squamous cell carcinoma | Cardiovascular |
| Hippocampus_middle | H3K4me3 | 20.52 | 8.04 | 0.01052347 | Lung squamous cell carcinoma | CNS |
| Fetal_small_intestine | H3K4me3 | 27.51 | 11.01 | 0.010550499 | Lung squamous cell carcinoma | GI |
| Fetal_trunk_muscle | H3K4me1 | 8.45 | 2.84 | 0.014338839 | Lung squamous cell carcinoma | SkeletalMuscle |
| Germinal_matrix | H3K4me3 | 26.29 | 10.94 | 0.015563011 | Lung squamous cell carcinoma | CNS |
| Substantia_nigra | H3K4me3 | 24.92 | 10.30 | 0.015912423 | Lung squamous cell carcinoma | CNS |
| Fetal_placenta | H3K4me3 | 33.12 | 14.23 | 0.016174023 | Lung squamous cell carcinoma | Other |
| Fetal_thymus | H3K4me3 | 34.00 | 12.91 | 0.016693496 | Lung squamous cell carcinoma | Hematopoietic |
| Rectal_mucosa | H3K4me3 | 19.52 | 8.80 | 0.018201541 | Lung squamous cell carcinoma | GI |
| Fetal_brain | H3K4me3 | 68.34 | 28.14 | 0.018782885 | Lung squamous cell carcinoma | CNS |
| Colonic_mucosa | H3K4me3 | 23.01 | 10.33 | 0.020334932 | Lung squamous cell carcinoma | GI |
| Penis_foreskin_melanocyte_primary | H3K4me3 | 14.73 | 6.27 | 0.02101633 | Lung squamous cell carcinoma | Other |
| Fetal_leg_muscle | H3K4me1 | 7.96 | 2.84 | 0.021289628 | Lung squamous cell carcinoma | SkeletalMuscle |
| Stomach_mucosa | H3K9ac | 20.80 | 9.54 | 0.023114491 | Lung squamous cell carcinoma | GI |
| Cingulate_gyrus | H3K4me3 | 18.91 | 7.99 | 0.02357441 | Lung squamous cell carcinoma | CNS |
| Fetal_large_intestine | H3K4me3 | 21.67 | 10.02 | 0.026877791 | Lung squamous cell carcinoma | GI |
| Esophagus | H3K4me3 | 28.83 | 13.11 | 0.026927093 | Lung squamous cell carcinoma | GI |
| Duodenum_Mucosa | H3K4me3 | 15.91 | 7.17 | 0.028418228 | Lung squamous cell carcinoma | GI |
| Duodenum_Mucosa | H3K4me1 | 9.34 | 4.42 | 0.029152058 | Lung squamous cell carcinoma | GI |
| CD56_primary | H3K4me3 | 29.80 | 13.21 | 0.029664461 | Lung squamous cell carcinoma | Hematopoietic |
| Left_Ventricle | H3K4me3 | 29.89 | 13.55 | 0.031279987 | Lung squamous cell carcinoma | Cardiovascular |
| Placenta_amnion | H3K4me3 | 38.14 | 17.52 | 0.031464409 | Lung squamous cell carcinoma | Other |
| Placenta_chorion | H3K4me1 | -23.09 | 11.39 | 0.031940395 | Lung squamous cell carcinoma | Other |
| Fetal_adrenal | H3K4me3 | 27.62 | 12.28 | 0.033007743 | Lung squamous cell carcinoma | Adrenal_Pancreas |
| Stomach_mucosa | H3K4me3 | 40.68 | 18.50 | 0.033256605 | Lung squamous cell carcinoma | GI |
| Aorta | H3K4me3 | 30.52 | 14.28 | 0.033665676 | Lung squamous cell carcinoma | Cardiovascular |
| Anterior_caudate | H3K27ac | 9.35 | 4.28 | 0.034552773 | Lung squamous cell carcinoma | CNS |
| Rectal_smooth_muscle | H3K9ac | 50.57 | 23.37 | 0.034718607 | Lung squamous cell carcinoma | GI |
| Placenta_chorion | H3K4me3 | 28.21 | 13.34 | 0.034940387 | Lung squamous cell carcinoma | Other |
| Fetal_leg_muscle | H3K4me3 | 23.89 | 11.57 | 0.041198489 | Lung squamous cell carcinoma | SkeletalMuscle |
| Lung | H3K4me3 | 38.98 | 18.71 | 0.041996023 | Lung squamous cell carcinoma | Cardiovascular |
| CD19_primary_(UW) | H3K4me3 | 27.94 | 12.91 | 0.042084917 | Lung squamous cell carcinoma | Hematopoietic |
| Inferior_temporal_lobe | H3K9ac | 15.54 | 7.62 | 0.043106077 | Lung squamous cell carcinoma | CNS |
| Duodenum_Mucosa | H3K9ac | 15.56 | 8.00 | 0.046210653 | Lung squamous cell carcinoma | GI |
| Small_intestine | H3K4me3 | 41.60 | 20.09 | 0.046541373 | Lung squamous cell carcinoma | GI |
| Pancreas | H3K4me3 | 26.54 | 13.08 | 0.047774488 | Lung squamous cell carcinoma | Adrenal_Pancreas |
| Fetal_trunk_muscle | H3K4me3 | 26.13 | 12.72 | 0.049804704 | Lung squamous cell carcinoma | SkeletalMuscle |
| Fetal_brain | H3K4me1 | 5.64 | 2.57 | 0.05043631 | Lung squamous cell carcinoma | CNS |
| Skeletal_muscle | H3K4me1 | 5.57 | 2.35 | 0.050523772 | Lung squamous cell carcinoma | SkeletalMuscle |
| Inferior_temporal_lobe | H3K4me3 | 16.71 | 8.29 | 0.050810633 | Lung squamous cell carcinoma | CNS |
| Gastric | H3K4me3 | 38.94 | 19.22 | 0.054723738 | Lung squamous cell carcinoma | GI |
| Sigmoid_colon | H3K4me3 | 39.57 | 19.70 | 0.05533999 | Lung squamous cell carcinoma | GI |
| Angular_gyrus | H3K4me3 | 22.40 | 11.43 | 0.055483426 | Lung squamous cell carcinoma | CNS |
| Angular_gyrus | H3K9ac | 20.08 | 10.33 | 0.058694315 | Lung squamous cell carcinoma | CNS |
| Rectal_smooth_muscle | H3K4me3 | 17.14 | 8.92 | 0.058988869 | Lung squamous cell carcinoma | GI |
| Colonic_mucosa | H3K9ac | 12.37 | 6.45 | 0.05995821 | Lung squamous cell carcinoma | GI |
| Fetal_kidney | H3K9ac | 39.67 | 19.58 | 0.060879 | Lung squamous cell carcinoma | Kidney |
| CD8_primary | H3K4me3 | 21.17 | 10.44 | 0.061997791 | Lung squamous cell carcinoma | Hematopoietic |
| CD4+_CD25-_IL17+_PMA_Ionomycin_stim_Th17_primary | H3K4me3 | 17.89 | 9.20 | 0.064484028 | Lung squamous cell carcinoma | Hematopoietic |
| Fetal_stomach | H3K4me3 | 22.49 | 11.90 | 0.065163679 | Lung squamous cell carcinoma | GI |
| Mid_frontal_lobe | H3K4me3 | 15.48 | 8.31 | 0.067150086 | Lung squamous cell carcinoma | CNS |
| Rectal_mucosa | H3K9ac | 14.17 | 7.64 | 0.068924358 | Lung squamous cell carcinoma | GI |
| CD4+_CD25-_Th_primary | H3K4me3 | 20.36 | 10.27 | 0.070946587 | Lung squamous cell carcinoma | Hematopoietic |
| Right_atrium | H3K4me3 | 20.89 | 11.36 | 0.072277737 | Lung squamous cell carcinoma | Cardiovascular |
| CD4+_CD25+_CD127-_Treg_primary | H3K4me3 | 17.33 | 8.97 | 0.077488659 | Lung squamous cell carcinoma | Hematopoietic |
| Skeletal_muscle | H3K9ac | 9.05 | 4.63 | 0.080103608 | Lung squamous cell carcinoma | SkeletalMuscle |
| CD3_primary | H3K27ac | 7.03 | 3.75 | 0.080799508 | Lung squamous cell carcinoma | Hematopoietic |
| CD4+_CD25-_CD45R0+_memory_primary | H3K4me3 | 21.64 | 11.87 | 0.084807455 | Lung squamous cell carcinoma | Hematopoietic |
| Colonic_mucosa | H3K27ac | 9.60 | 5.61 | 0.086088681 | Lung squamous cell carcinoma | GI |
| Duodenum_smooth_muscle | H3K4me3 | 11.72 | 6.11 | 0.087586274 | Lung squamous cell carcinoma | GI |
| Fetal_heart | H3K9ac | 9.69 | 5.08 | 0.087889557 | Lung squamous cell carcinoma | Cardiovascular |
| Anterior_caudate | H3K4me3 | 13.83 | 7.68 | 0.088860891 | Lung squamous cell carcinoma | CNS |
| Rectal_mucosa | H3K4me1 | 8.04 | 4.43 | 0.089151995 | Lung squamous cell carcinoma | GI |
| Thymus | H3K4me1 | 20.90 | 11.81 | 0.090619134 | Lung squamous cell carcinoma | Hematopoietic |
| CD4+_CD25int_CD127+_Tmem_primary | H3K4me3 | 23.13 | 12.46 | 0.09149206 | Lung squamous cell carcinoma | Hematopoietic |
| Colon_smooth_muscle | H3K4me3 | 14.78 | 8.11 | 0.092093645 | Lung squamous cell carcinoma | GI |
| Substantia_nigra | H3K9ac | 12.29 | 6.88 | 0.092723174 | Lung squamous cell carcinoma | CNS |
| CD34_primary | H3K4me3 | 19.43 | 10.62 | 0.094722572 | Lung squamous cell carcinoma | Hematopoietic |
| CD25-_IL17+_Th17_stim | H3K27ac | 8.95 | 5.07 | 0.094798465 | Lung squamous cell carcinoma | Hematopoietic |
| Treg_primary | H3K4me3 | 10.63 | 5.85 | 0.095891195 | Lung squamous cell carcinoma | Hematopoietic |
| CD8_naive_primary_(UCSF-UBC) | H3K9ac | 57.27 | 32.73 | 0.096346677 | Lung squamous cell carcinoma | Hematopoietic |
| CD8_naive_primary_(BI) | H3K4me3 | 19.03 | 10.70 | 0.097583071 | Lung squamous cell carcinoma | Hematopoietic |
| Mobilized_CD34_primary | H3K4me1 | 4.96 | 2.41 | 0.098394232 | Lung squamous cell carcinoma | Hematopoietic |
| Fetal_stomach | H3K4me1 | 6.99 | 3.47 | 0.099256338 | Lung squamous cell carcinoma | GI |
| Hippocampus_middle | H3K27ac | 7.84 | 4.48 | 0.099562288 | Lung squamous cell carcinoma | CNS |
| CD3_primary_(UW) | H3K4me3 | 18.34 | 10.49 | 0.09979339 | Lung squamous cell carcinoma | Hematopoietic |
| CD4_naive_primary | H3K4me3 | 16.94 | 9.43 | 0.100707857 | Lung squamous cell carcinoma | Hematopoietic |
| Skeletal_muscle | H3K4me3 | 10.82 | 6.11 | 0.103979445 | Lung squamous cell carcinoma | SkeletalMuscle |
| Colon_smooth_muscle | H3K9ac | 21.41 | 12.97 | 0.104535656 | Lung squamous cell carcinoma | GI |
| Hippocampus_middle | H3K9ac | 12.01 | 7.01 | 0.106034773 | Lung squamous cell carcinoma | CNS |
| CD4+_CD25-_CD45RA+_naive_primary | H3K4me3 | 17.03 | 9.61 | 0.106075388 | Lung squamous cell carcinoma | Hematopoietic |
| Fetal_thymus | H3K4me1 | 6.03 | 3.18 | 0.106375294 | Lung squamous cell carcinoma | Hematopoietic |
| CD8_memory_primary | H3K4me3 | 16.50 | 9.78 | 0.108977642 | Lung squamous cell carcinoma | Hematopoietic |
| Peripheralblood_mononuclear_primary | H3K4me3 | 21.28 | 12.19 | 0.109349248 | Lung squamous cell carcinoma | Hematopoietic |
| Adipose_nuclei | H3K4me3 | 8.24 | 4.44 | 0.11077829 | Lung squamous cell carcinoma | Other |
| Ovary | H3K4me3 | 20.33 | 11.87 | 0.111048785 | Lung squamous cell carcinoma | Other |
| Substantia_nigra | H3K4me1 | 5.58 | 2.90 | 0.113471851 | Lung squamous cell carcinoma | CNS |
| Stomach_smooth_muscle | H3K4me1 | 8.70 | 4.76 | 0.114382831 | Lung squamous cell carcinoma | GI |
| Rectal_smooth_muscle | H3K27ac | 7.01 | 3.77 | 0.115615329 | Lung squamous cell carcinoma | GI |
| CD19_primary_(BI) | H3K4me1 | 5.63 | 3.06 | 0.119236762 | Lung squamous cell carcinoma | Hematopoietic |
| Mobilized_CD34_primary | H3K4me3 | 11.24 | 6.49 | 0.121339638 | Lung squamous cell carcinoma | Hematopoietic |
| CD8_naive_primary_(UCSF-UBC) | H3K4me3 | 25.42 | 15.46 | 0.124194518 | Lung squamous cell carcinoma | Hematopoietic |
| Fetal_large_intestine | H3K4me1 | 7.98 | 5.15 | 0.127760342 | Lung squamous cell carcinoma | GI |
| Inferior_temporal_lobe | H3K27ac | 6.08 | 3.63 | 0.131876642 | Lung squamous cell carcinoma | CNS |
| Fetal_brain | H3K4me3 | 14.27 | 8.99 | 0.132170217 | Lung squamous cell carcinoma | CNS |
| CD4_primary | H3K4me3 | 15.26 | 9.19 | 0.134323284 | Lung squamous cell carcinoma | Hematopoietic |
| Stomach_smooth_muscle | H3K4me3 | 10.21 | 6.20 | 0.134773493 | Lung squamous cell carcinoma | GI |
| Osteoblast | H3K27ac | -3.24 | 3.03 | 0.135328434 | Lung squamous cell carcinoma | Connective_Bone |
| CD34_primary | H3K4me1 | 7.29 | 4.35 | 0.137752875 | Lung squamous cell carcinoma | Hematopoietic |
| Colon_smooth_muscle | H3K4me1 | 6.69 | 3.90 | 0.142947435 | Lung squamous cell carcinoma | GI |
| CD56_primary | H3K4me1 | 6.26 | 3.55 | 0.14655077 | Lung squamous cell carcinoma | Hematopoietic |
| CD4_memory_primary | H3K4me3 | 14.98 | 9.44 | 0.146840326 | Lung squamous cell carcinoma | Hematopoietic |
| CD14_primary | H3K4me1 | 4.87 | 2.90 | 0.150146462 | Lung squamous cell carcinoma | Hematopoietic |
| Cingulate_gyrus | H3K9ac | 10.44 | 6.68 | 0.154041908 | Lung squamous cell carcinoma | CNS |
| Substantia_nigra | H3K27ac | 5.35 | 3.16 | 0.154742686 | Lung squamous cell carcinoma | CNS |
| CD19_primary_(UW) | H3K4me1 | 5.41 | 3.13 | 0.155941172 | Lung squamous cell carcinoma | Hematopoietic |
| Kidney | H3K9ac | 13.11 | 8.71 | 0.158159577 | Lung squamous cell carcinoma | Kidney |
| Stomach_smooth_muscle | H3K9ac | 10.88 | 7.22 | 0.160193167 | Lung squamous cell carcinoma | GI |
| Liver_(BI) | H3K4me3 | 9.25 | 5.80 | 0.170045539 | Lung squamous cell carcinoma | Liver |
| Peripheralblood_mononuclear_primary | H3K9ac | 14.73 | 9.91 | 0.171505012 | Lung squamous cell carcinoma | Hematopoietic |
| Colon_smooth_muscle | H3K27ac | 5.99 | 3.67 | 0.174564647 | Lung squamous cell carcinoma | GI |
| Penis_foreskin_melanocyte_primary | H3K4me1 | -2.13 | 2.41 | 0.175383784 | Lung squamous cell carcinoma | Other |
| Inferior_temporal_lobe | H3K4me1 | 4.68 | 2.72 | 0.178688895 | Lung squamous cell carcinoma | CNS |
| Angular_gyrus | H3K27ac | 5.93 | 3.91 | 0.179754437 | Lung squamous cell carcinoma | CNS |
| Penis_foreskin_keratinocyte_primary | H3K4me3 | 9.28 | 6.33 | 0.192020294 | Lung squamous cell carcinoma | Other |
| CD3_primary_(UW) | H3K4me1 | 5.60 | 3.53 | 0.192853824 | Lung squamous cell carcinoma | Hematopoietic |
| CD4+_CD25-_IL17-_PMA_Ionomycin_stim_MACS_Th_sprimary | H3K4me3 | 9.58 | 6.43 | 0.196588636 | Lung squamous cell carcinoma | Hematopoietic |
| Cingulate_gyrus | H3K27ac | 5.20 | 3.39 | 0.199773417 | Lung squamous cell carcinoma | CNS |
| Hippocampus_middle | H3K4me1 | 4.29 | 2.57 | 0.203629596 | Lung squamous cell carcinoma | CNS |
| CD4+_CD25-_Th_primary | H3K4me1 | 4.67 | 2.92 | 0.203630655 | Lung squamous cell carcinoma | Hematopoietic |
| CD3_primary_(BI) | H3K4me3 | 11.39 | 7.96 | 0.208659388 | Lung squamous cell carcinoma | Hematopoietic |
| Duodenum_mucosa | H3K27ac | 8.01 | 6.14 | 0.212433417 | Lung squamous cell carcinoma | GI |
| CD4+_CD25-_IL17-_PMA_Ionomycin_stim_MACS_Th_sprimary | H3K4me1 | 3.98 | 2.46 | 0.213870107 | Lung squamous cell carcinoma | Hematopoietic |
| CD15_primary | H3K4me3 | 9.09 | 6.58 | 0.219882403 | Lung squamous cell carcinoma | Hematopoietic |
| Th0 | H3K27ac | 5.16 | 3.51 | 0.222117051 | Lung squamous cell carcinoma | Hematopoietic |
| CD4+_CD25-_CD45R0+_memory_primary | H3K4me1 | 4.94 | 3.36 | 0.222342548 | Lung squamous cell carcinoma | Hematopoietic |
| Colonic_mucosa | H3K4me1 | 7.76 | 5.85 | 0.227768872 | Lung squamous cell carcinoma | GI |
| Rectal_mucosa | H3K27ac | 6.98 | 5.35 | 0.228266128 | Lung squamous cell carcinoma | GI |
| Kidney | H3K4me3 | 9.79 | 7.34 | 0.228796979 | Lung squamous cell carcinoma | Kidney |
| Anterior_caudate | H3K4me1 | 4.84 | 3.24 | 0.232526891 | Lung squamous cell carcinoma | CNS |
| CD4+_CD25-_IL17+_PMA_Ionomycin_stim_Th17_primary | H3K4me1 | 4.57 | 3.09 | 0.236186431 | Lung squamous cell carcinoma | Hematopoietic |
| Fetal_heart | H3K4me3 | 17.44 | 13.71 | 0.237016735 | Lung squamous cell carcinoma | Cardiovascular |
| CD25+_CD127-_Treg | H3K27ac | 5.59 | 4.08 | 0.2386166 | Lung squamous cell carcinoma | Hematopoietic |
| Fetal_small_intestine | H3K4me1 | 6.45 | 5.08 | 0.238833253 | Lung squamous cell carcinoma | GI |
| CD15_primary | H3K4me1 | 4.04 | 2.70 | 0.241796136 | Lung squamous cell carcinoma | Hematopoietic |
| Breast_myoepithelial | H3K9ac | 26.82 | 21.79 | 0.245521324 | Lung squamous cell carcinoma | Other |
| Spleen | H3K4me3 | 30.25 | 25.23 | 0.253271561 | Lung squamous cell carcinoma | Hematopoietic |
| Pancreas | H3K4me1 | 5.72 | 4.16 | 0.260563931 | Lung squamous cell carcinoma | Adrenal_Pancreas |
| Fetal_brain | H3K9ac | 10.10 | 8.16 | 0.264805343 | Lung squamous cell carcinoma | CNS |
| Adipose_nuclei | H3K4me1 | 3.80 | 2.46 | 0.265350411 | Lung squamous cell carcinoma | Other |
| CD4+_CD25+_CD127-_Treg_primary | H3K4me1 | 5.36 | 4.04 | 0.270069358 | Lung squamous cell carcinoma | Hematopoietic |
| Fetal_lung | H3K4me3 | 12.05 | 9.87 | 0.270493839 | Lung squamous cell carcinoma | Cardiovascular |
| Duodenum_smooth_muscle | H3K27ac | 6.78 | 5.52 | 0.272842708 | Lung squamous cell carcinoma | GI |
| CD8_naive_primary_(UCSF-UBC) | H3K4me1 | 5.48 | 4.17 | 0.275878658 | Lung squamous cell carcinoma | Hematopoietic |
| CD4_memory_primary | H3K4me1 | 4.04 | 2.90 | 0.280300872 | Lung squamous cell carcinoma | Hematopoietic |
| Breast_fibroblast_primary | H3K4me3 | 21.25 | 18.26 | 0.281296296 | Lung squamous cell carcinoma | Connective_Bone |
| Anterior_caudate | H3K9ac | 9.18 | 7.70 | 0.281456374 | Lung squamous cell carcinoma | CNS |
| CD14_primary | H3K4me3 | 13.23 | 11.40 | 0.286159655 | Lung squamous cell carcinoma | Hematopoietic |
| Th1 | H3K27ac | 4.71 | 3.57 | 0.293575643 | Lung squamous cell carcinoma | Hematopoietic |
| Right_atrium | H3K4me1 | 10.65 | 9.48 | 0.297763488 | Lung squamous cell carcinoma | Cardiovascular |
| Breast_vHMEC | H3K4me3 | 14.91 | 13.39 | 0.302284039 | Lung squamous cell carcinoma | Other |
| Liver_(UCSD) | H3K4me1 | -34.21 | 35.01 | 0.306407503 | Lung squamous cell carcinoma | Liver |
| Liver_(BI) | H3K4me1 | 3.79 | 2.79 | 0.307873909 | Lung squamous cell carcinoma | Liver |
| CD19_primary_(BI) | H3K4me3 | 10.51 | 9.28 | 0.3081852 | Lung squamous cell carcinoma | Hematopoietic |
| Mid_frontal_lobe | H3K27ac | 5.47 | 4.59 | 0.308658222 | Lung squamous cell carcinoma | CNS |
| Pancreatic_islets | H3K4me3 | 11.34 | 10.12 | 0.310106978 | Lung squamous cell carcinoma | Adrenal_Pancreas |
| Cingulate_gyrus | H3K4me1 | 3.88 | 2.85 | 0.316152413 | Lung squamous cell carcinoma | CNS |
| Pancreatic_islets | H3K9ac | 15.82 | 14.58 | 0.317869066 | Lung squamous cell carcinoma | Adrenal_Pancreas |
| Sigmoid_colon | H3K4me1 | -18.18 | 19.39 | 0.321977876 | Lung squamous cell carcinoma | GI |
| Mid_frontal_lobe | H3K9ac | 8.57 | 8.02 | 0.3338083 | Lung squamous cell carcinoma | CNS |
| Stomach_mucosa | H3K4me1 | 5.03 | 4.51 | 0.334512955 | Lung squamous cell carcinoma | GI |
| Stomach_smooth_muscle | H3K27ac | 4.23 | 3.53 | 0.356953704 | Lung squamous cell carcinoma | GI |
| Breast_myoepithelial | H3K4me3 | 8.26 | 8.15 | 0.366347732 | Lung squamous cell carcinoma | Other |
| Adipose_nuclei | H3K9ac | 6.06 | 5.59 | 0.370238689 | Lung squamous cell carcinoma | Other |
| Fetal_heart | H3K4me1 | 3.25 | 2.58 | 0.378144405 | Lung squamous cell carcinoma | Cardiovascular |
| Angular_gyrus | H3K4me1 | 6.18 | 6.09 | 0.381289367 | Lung squamous cell carcinoma | CNS |
| Ovary | H3K4me1 | -8.21 | 11.23 | 0.382972561 | Lung squamous cell carcinoma | Other |
| CD19 | H3K27ac | 3.83 | 3.38 | 0.383306097 | Lung squamous cell carcinoma | Hematopoietic |
| Gastric | H3K4me1 | 8.58 | 8.56 | 0.385031483 | Lung squamous cell carcinoma | GI |
| Adipose_nuclei | H3K27ac | -1.85 | 3.63 | 0.395354414 | Lung squamous cell carcinoma | Other |
| Mobilized_CD34 | H3K27ac | 4.14 | 3.75 | 0.397547179 | Lung squamous cell carcinoma | Hematopoietic |
| Kidney | H3K27ac | 4.11 | 3.85 | 0.401499378 | Lung squamous cell carcinoma | Kidney |
| Breast_vHMEC | H3K4me1 | -2.44 | 4.13 | 0.407277424 | Lung squamous cell carcinoma | Other |
| Liver_(UCSD) | H3K4me3 | 8.48 | 8.96 | 0.408349662 | Lung squamous cell carcinoma | Liver |
| Chondrogenic_dif | H3K27ac | -1.86 | 3.60 | 0.413181262 | Lung squamous cell carcinoma | Connective_Bone |
| CD25-_IL17-_Th_stim_MACS | H3K27ac | 4.05 | 3.93 | 0.42821702 | Lung squamous cell carcinoma | Hematopoietic |
| CD25-_CD45RA+_naive | H3K27ac | 3.84 | 3.72 | 0.428648147 | Lung squamous cell carcinoma | Hematopoietic |
| CD14 | H3K27ac | 3.38 | 3.18 | 0.443347297 | Lung squamous cell carcinoma | Hematopoietic |
| Pancreatic_islets | H3K4me1 | 9.83 | 11.87 | 0.450229735 | Lung squamous cell carcinoma | Adrenal_Pancreas |
| Fetal_lung | H3K9ac | 7.55 | 8.77 | 0.456759499 | Lung squamous cell carcinoma | Cardiovascular |
| CD8_memory_primary | H3K4me1 | 3.57 | 3.56 | 0.463173994 | Lung squamous cell carcinoma | Hematopoietic |
| CD20 | H3K27ac | 3.22 | 3.21 | 0.475084831 | Lung squamous cell carcinoma | Hematopoietic |
| CD4+_CD25-_CD45RA+_naive_primary | H3K4me1 | 3.06 | 2.93 | 0.48136857 | Lung squamous cell carcinoma | Hematopoietic |
| Penis_foreskin_fibroblast_primary | H3K4me3 | 3.08 | 3.06 | 0.492874035 | Lung squamous cell carcinoma | Connective_Bone |
| Psoas_muscle | H3K4me3 | 8.95 | 11.71 | 0.50123027 | Lung squamous cell carcinoma | SkeletalMuscle |
| Skeletal_muscle | H3K27ac | 3.34 | 3.54 | 0.504954699 | Lung squamous cell carcinoma | SkeletalMuscle |
| CD4_naive_primary | H3K4me1 | 3.21 | 3.37 | 0.506885793 | Lung squamous cell carcinoma | Hematopoietic |
| Breast_luminal_epithelial | H3K4me1 | -24.45 | 39.12 | 0.514411995 | Lung squamous cell carcinoma | Other |
| Pancreatic_islets | H3K27ac | 2.88 | 3.25 | 0.553909792 | Lung squamous cell carcinoma | Adrenal_Pancreas |
| Psoas_muscle | H3K4me1 | -8.28 | 16.56 | 0.574632301 | Lung squamous cell carcinoma | SkeletalMuscle |
| CD8_naive_primary_(BI) | H3K4me1 | 2.86 | 3.40 | 0.581946437 | Lung squamous cell carcinoma | Hematopoietic |
| Lung | H3K4me1 | -1.95 | 5.51 | 0.587778835 | Lung squamous cell carcinoma | Cardiovascular |
| Peripheralblood_mononuclear_primary | H3K4me1 | 7.77 | 12.66 | 0.591780344 | Lung squamous cell carcinoma | Hematopoietic |
| Spleen | H3K4me1 | 3.00 | 3.79 | 0.594491146 | Lung squamous cell carcinoma | Hematopoietic |
| CD4+_CD25int_CD127+_Tmem_primary | H3K4me1 | 4.10 | 5.92 | 0.597820351 | Lung squamous cell carcinoma | Hematopoietic |
| Liver_(BI) | H3K9ac | 4.61 | 7.08 | 0.610536862 | Lung squamous cell carcinoma | Liver |
| Rectal_smooth_muscle | H3K4me1 | 5.27 | 8.54 | 0.616677577 | Lung squamous cell carcinoma | GI |
| Penis_foreskin_keratinocyte_primary | H3K9ac | 2.70 | 3.44 | 0.623224283 | Lung squamous cell carcinoma | Other |
| Kidney | H3K4me1 | 5.16 | 8.91 | 0.639009786 | Lung squamous cell carcinoma | Kidney |
| Th2 | H3K27ac | 2.59 | 3.47 | 0.642450225 | Lung squamous cell carcinoma | Hematopoietic |
| Pancreatic_islets | H3K4me3 | 4.99 | 10.88 | 0.714112451 | Lung squamous cell carcinoma | Adrenal_Pancreas |
| Penis_foreskin_fibroblast_primary | H3K4me1 | 0.33 | 1.96 | 0.730322146 | Lung squamous cell carcinoma | Connective_Bone |
| Fetal_placenta | H3K4me1 | 2.56 | 4.58 | 0.732011571 | Lung squamous cell carcinoma | Other |
| Breast_myoepithelial | H3K4me1 | 2.01 | 3.01 | 0.732531179 | Lung squamous cell carcinoma | Other |
| CD3_primary_(BI) | H3K4me1 | 2.07 | 3.35 | 0.749066204 | Lung squamous cell carcinoma | Hematopoietic |
| Penis_foreskin_keratinocyte_primary | H3K4me1 | 0.18 | 2.67 | 0.753988439 | Lung squamous cell carcinoma | Other |
| Placenta_amnion | H3K4me1 | 6.34 | 18.07 | 0.765235416 | Lung squamous cell carcinoma | Other |
| Mid_frontal_lobe | H3K4me1 | -3.55 | 16.06 | 0.775050731 | Lung squamous cell carcinoma | CNS |
| Small_intestine | H3K4me1 | 12.23 | 45.39 | 0.803519409 | Lung squamous cell carcinoma | GI |
| Liver | H3K27ac | 0.15 | 3.91 | 0.825406157 | Lung squamous cell carcinoma | Liver |
| Left_Ventricle | H3K4me1 | 2.07 | 4.91 | 0.825801332 | Lung squamous cell carcinoma | Cardiovascular |
| Fetal_adrenal | H3K4me1 | 1.70 | 3.56 | 0.841648385 | Lung squamous cell carcinoma | Adrenal_Pancreas |
| Breast_fibroblast_primary | H3K4me1 | -0.75 | 9.16 | 0.84834687 | Lung squamous cell carcinoma | Connective_Bone |
| Esophagus | H3K4me1 | -0.61 | 12.54 | 0.89778229 | Lung squamous cell carcinoma | GI |
| Pancreatic_islets | H3K4me1 | 0.14 | 8.66 | 0.920684101 | Lung squamous cell carcinoma | Adrenal_Pancreas |
| CD25int_CD127+_Tmem | H3K27ac | 1.35 | 3.96 | 0.927767652 | Lung squamous cell carcinoma | Hematopoietic |
| Duodenum_smooth_muscle | H3K4me1 | -0.22 | 15.62 | 0.936957465 | Lung squamous cell carcinoma | GI |
| Right_ventricle | H3K4me1 | 3.78 | 38.99 | 0.939672646 | Lung squamous cell carcinoma | Cardiovascular |
| Fetal_lung | H3K4me1 | 1.14 | 2.55 | 0.955670333 | Lung squamous cell carcinoma | Cardiovascular |
| Neurosphere | H3K27ac | 0.82 | 3.48 | 0.959693796 | Lung squamous cell carcinoma | CNS |
| Kidney | H3K27ac | 11.70 | 4.04 | 0.007835001 | Serous ovarian cancer | Kidney |
| Sigmoid_colon | H3K4me1 | -35.45 | 18.40 | 0.008693782 | Serous ovarian cancer | GI |
| Right_ventricle | H3K4me1 | -61.61 | 22.74 | 0.009713031 | Serous ovarian cancer | Cardiovascular |
| Liver | H3K27ac | 6.47 | 2.57 | 0.010286406 | Serous ovarian cancer | Liver |
| Pancreas | H3K4me1 | 8.66 | 3.30 | 0.01344651 | Serous ovarian cancer | Adrenal_Pancreas |
| Breast_vHMEC | H3K4me1 | -6.28 | 3.71 | 0.019185717 | Serous ovarian cancer | Other |
| Right_atrium | H3K4me1 | -15.75 | 6.63 | 0.02229775 | Serous ovarian cancer | Cardiovascular |
| Liver_(BI) | H3K4me1 | 4.93 | 1.70 | 0.033446486 | Serous ovarian cancer | Liver |
| Neurosphere | H3K27ac | 5.49 | 2.43 | 0.036530783 | Serous ovarian cancer | CNS |
| Duodenum_mucosa | H3K27ac | 10.41 | 4.85 | 0.040297465 | Serous ovarian cancer | GI |
| Duodenum_Mucosa | H3K4me3 | 14.22 | 5.37 | 0.043901944 | Serous ovarian cancer | GI |
| Fetal_small_intestine | H3K4me3 | 26.68 | 10.71 | 0.048175916 | Serous ovarian cancer | GI |
| Placenta_chorion | H3K4me1 | -13.34 | 6.78 | 0.049589472 | Serous ovarian cancer | Other |
| Anterior_caudate | H3K27ac | 6.78 | 3.16 | 0.052773803 | Serous ovarian cancer | CNS |
| Liver_(BI) | H3K9ac | 12.32 | 5.19 | 0.060670799 | Serous ovarian cancer | Liver |
| Kidney | H3K4me3 | 25.41 | 11.34 | 0.061206848 | Serous ovarian cancer | Kidney |
| Rectal_smooth_muscle | H3K27ac | 5.67 | 2.38 | 0.076142695 | Serous ovarian cancer | GI |
| Liver_(BI) | H3K4me3 | 8.88 | 3.89 | 0.081057891 | Serous ovarian cancer | Liver |
| Colon_smooth_muscle | H3K4me3 | 20.78 | 9.57 | 0.083770977 | Serous ovarian cancer | GI |
| Ovary | H3K4me3 | 26.90 | 16.22 | 0.084180784 | Serous ovarian cancer | Other |
| Fetal_large_intestine | H3K4me3 | 23.12 | 11.16 | 0.09159577 | Serous ovarian cancer | GI |
| Pancreatic_islets | H3K27ac | 4.65 | 2.30 | 0.100312169 | Serous ovarian cancer | Adrenal_Pancreas |
| Rectal_mucosa | H3K4me3 | 20.01 | 10.01 | 0.102655774 | Serous ovarian cancer | GI |
| Breast_myoepithelial | H3K9ac | -24.92 | 16.78 | 0.104812998 | Serous ovarian cancer | Other |
| Breast_myoepithelial | H3K4me1 | -1.93 | 2.11 | 0.106366307 | Serous ovarian cancer | Other |
| CD25-_CD45RA+_naive | H3K27ac | 4.63 | 2.45 | 0.112242834 | Serous ovarian cancer | Hematopoietic |
| CD25-_IL17-_Th_stim_MACS | H3K27ac | 6.22 | 3.02 | 0.11440384 | Serous ovarian cancer | Hematopoietic |
| Fetal_small_intestine | H3K4me1 | 4.82 | 2.35 | 0.12068908 | Serous ovarian cancer | GI |
| Fetal_stomach | H3K4me3 | 25.70 | 13.95 | 0.121047403 | Serous ovarian cancer | GI |
| CD4+_CD25-_IL17-_PMA_Ionomycin_stim_MACS_Th_sprimary | H3K4me1 | 4.81 | 2.22 | 0.127179785 | Serous ovarian cancer | Hematopoietic |
| Colon_smooth_muscle | H3K27ac | 5.25 | 2.64 | 0.132551669 | Serous ovarian cancer | GI |
| Liver_(UCSD) | H3K4me1 | 51.95 | 40.65 | 0.136834065 | Serous ovarian cancer | Liver |
| Fetal_kidney | H3K9ac | 58.75 | 35.75 | 0.13912205 | Serous ovarian cancer | Kidney |
| Colonic_mucosa | H3K4me3 | 23.44 | 13.45 | 0.145854076 | Serous ovarian cancer | GI |
| CD4_memory_primary | H3K4me1 | 5.06 | 2.52 | 0.149644116 | Serous ovarian cancer | Hematopoietic |
| Lung | H3K4me1 | -5.28 | 3.97 | 0.151000466 | Serous ovarian cancer | Cardiovascular |
| Fetal_large_intestine | H3K4me1 | 4.75 | 2.56 | 0.154750409 | Serous ovarian cancer | GI |
| Liver_(UCSD) | H3K4me3 | 13.16 | 8.09 | 0.162237986 | Serous ovarian cancer | Liver |
| Duodenum_smooth_muscle | H3K4me3 | 8.70 | 4.88 | 0.165458244 | Serous ovarian cancer | GI |
| Colonic_mucosa | H3K27ac | 7.27 | 4.37 | 0.166049989 | Serous ovarian cancer | GI |
| Skeletal_muscle | H3K27ac | 4.39 | 2.51 | 0.176234267 | Serous ovarian cancer | SkeletalMuscle |
| Small_intestine | H3K4me3 | 33.19 | 21.92 | 0.180787501 | Serous ovarian cancer | GI |
| Kidney | H3K9ac | 28.02 | 18.79 | 0.183334105 | Serous ovarian cancer | Kidney |
| CD4+_CD25-_CD45RA+_naive_primary | H3K4me1 | 4.76 | 2.62 | 0.187135627 | Serous ovarian cancer | Hematopoietic |
| CD3_primary_(UW) | H3K4me1 | 4.78 | 2.65 | 0.187876109 | Serous ovarian cancer | Hematopoietic |
| CD4+_CD25-_CD45R0+_memory_primary | H3K4me1 | 5.29 | 2.98 | 0.188241679 | Serous ovarian cancer | Hematopoietic |
| Colon_smooth_muscle | H3K4me1 | 4.63 | 2.59 | 0.189583482 | Serous ovarian cancer | GI |
| Breast_myoepithelial | H3K4me3 | -6.91 | 7.31 | 0.190981873 | Serous ovarian cancer | Other |
| CD4+_CD25-_Th_primary | H3K4me1 | 4.91 | 2.79 | 0.196415957 | Serous ovarian cancer | Hematopoietic |
| Thymus | H3K4me1 | -5.38 | 4.87 | 0.200010183 | Serous ovarian cancer | Hematopoietic |
| Sigmoid_colon | H3K4me3 | 27.57 | 19.11 | 0.202600194 | Serous ovarian cancer | GI |
| Mobilized_CD34 | H3K27ac | -2.08 | 2.71 | 0.224776415 | Serous ovarian cancer | Hematopoietic |
| Rectal_smooth_muscle | H3K4me3 | 19.78 | 14.57 | 0.227044378 | Serous ovarian cancer | GI |
| CD4+_CD25-_IL17+_PMA_Ionomycin_stim_Th17_primary | H3K4me1 | 4.71 | 2.85 | 0.228307211 | Serous ovarian cancer | Hematopoietic |
| CD8_memory_primary | H3K4me1 | 4.78 | 2.95 | 0.23061118 | Serous ovarian cancer | Hematopoietic |
| Penis_foreskin_melanocyte_primary | H3K4me3 | 9.23 | 6.48 | 0.230834469 | Serous ovarian cancer | Other |
| CD4_naive_primary | H3K4me1 | 4.45 | 2.77 | 0.242519587 | Serous ovarian cancer | Hematopoietic |
| CD3_primary_(BI) | H3K4me1 | 4.42 | 2.81 | 0.254378617 | Serous ovarian cancer | Hematopoietic |
| Stomach_mucosa | H3K9ac | 11.27 | 8.29 | 0.254518762 | Serous ovarian cancer | GI |
| Fetal_lung | H3K4me3 | 15.19 | 11.55 | 0.255065293 | Serous ovarian cancer | Cardiovascular |
| Pancreatic_islets | H3K4me1 | 7.78 | 6.19 | 0.257824497 | Serous ovarian cancer | Adrenal_Pancreas |
| CD3_primary | H3K27ac | 3.55 | 2.21 | 0.258336249 | Serous ovarian cancer | Hematopoietic |
| Esophagus | H3K4me1 | -9.00 | 8.43 | 0.260715044 | Serous ovarian cancer | GI |
| Hippocampus_middle | H3K27ac | 4.29 | 2.87 | 0.260841904 | Serous ovarian cancer | CNS |
| CD4+_CD25+_CD127-_Treg_primary | H3K4me1 | 5.87 | 4.15 | 0.268360464 | Serous ovarian cancer | Hematopoietic |
| Fetal_brain | H3K4me3 | -16.63 | 20.25 | 0.270974297 | Serous ovarian cancer | CNS |
| CD8_naive_primary_(BI) | H3K4me1 | 4.12 | 2.70 | 0.271875027 | Serous ovarian cancer | Hematopoietic |
| Fetal_leg_muscle | H3K4me3 | 13.42 | 10.94 | 0.280914567 | Serous ovarian cancer | SkeletalMuscle |
| Fetal_brain | H3K4me3 | -5.39 | 6.32 | 0.283883474 | Serous ovarian cancer | CNS |
| CD4+_CD25-_IL17-_PMA_Ionomycin_stim_MACS_Th_sprimary | H3K4me3 | 6.68 | 4.90 | 0.286625692 | Serous ovarian cancer | Hematopoietic |
| Psoas_muscle | H3K4me3 | 13.04 | 10.89 | 0.291600905 | Serous ovarian cancer | SkeletalMuscle |
| Fetal_placenta | H3K4me3 | 12.96 | 10.76 | 0.302093788 | Serous ovarian cancer | Other |
| CD4+_CD25int_CD127+_Tmem_primary | H3K4me1 | 6.32 | 4.87 | 0.302366664 | Serous ovarian cancer | Hematopoietic |
| Germinal_matrix | H3K4me3 | -6.03 | 7.49 | 0.306507798 | Serous ovarian cancer | CNS |
| Rectal_mucosa | H3K9ac | 10.82 | 9.34 | 0.309416994 | Serous ovarian cancer | GI |
| Mid_frontal_lobe | H3K9ac | -4.26 | 5.84 | 0.314155271 | Serous ovarian cancer | CNS |
| Pancreas | H3K4me3 | 12.98 | 11.31 | 0.316551762 | Serous ovarian cancer | Adrenal_Pancreas |
| Mid_frontal_lobe | H3K4me3 | -4.26 | 5.65 | 0.323463851 | Serous ovarian cancer | CNS |
| CD3_primary_(BI) | H3K4me3 | 7.87 | 6.77 | 0.332047868 | Serous ovarian cancer | Hematopoietic |
| CD25+_CD127-_Treg | H3K27ac | 3.48 | 2.53 | 0.338044573 | Serous ovarian cancer | Hematopoietic |
| Rectal_mucosa | H3K27ac | 4.82 | 3.94 | 0.34442968 | Serous ovarian cancer | GI |
| Angular_gyrus | H3K27ac | 3.48 | 2.68 | 0.352974184 | Serous ovarian cancer | CNS |
| Fetal_trunk_muscle | H3K4me3 | 12.63 | 12.03 | 0.353747013 | Serous ovarian cancer | SkeletalMuscle |
| Placenta_chorion | H3K4me3 | 9.27 | 8.46 | 0.353907091 | Serous ovarian cancer | Other |
| Pancreatic_islets | H3K4me3 | 9.46 | 8.84 | 0.357317535 | Serous ovarian cancer | Adrenal_Pancreas |
| Fetal_lung | H3K4me1 | 2.81 | 1.88 | 0.358260894 | Serous ovarian cancer | Cardiovascular |
| Angular_gyrus | H3K4me3 | -5.68 | 7.91 | 0.361823471 | Serous ovarian cancer | CNS |
| Th1 | H3K27ac | 3.95 | 3.36 | 0.36247618 | Serous ovarian cancer | Hematopoietic |
| CD8_naive_primary_(UCSF-UBC) | H3K4me1 | 4.21 | 3.48 | 0.363187135 | Serous ovarian cancer | Hematopoietic |
| CD4+_CD25-_CD45RA+_naive_primary | H3K4me3 | 8.23 | 7.70 | 0.364364658 | Serous ovarian cancer | Hematopoietic |
| CD4_naive_primary | H3K4me3 | 7.50 | 7.00 | 0.37639774 | Serous ovarian cancer | Hematopoietic |
| Substantia_nigra | H3K27ac | 3.07 | 2.35 | 0.379557666 | Serous ovarian cancer | CNS |
| Adipose_nuclei | H3K4me3 | 4.33 | 3.62 | 0.382865146 | Serous ovarian cancer | Other |
| Th0 | H3K27ac | 3.98 | 3.61 | 0.383773968 | Serous ovarian cancer | Hematopoietic |
| Treg_primary | H3K4me3 | 6.06 | 5.64 | 0.384154566 | Serous ovarian cancer | Hematopoietic |
| Kidney | H3K4me1 | 6.98 | 7.47 | 0.386529521 | Serous ovarian cancer | Kidney |
| Duodenum_Mucosa | H3K4me1 | 2.82 | 2.13 | 0.39609484 | Serous ovarian cancer | GI |
| Fetal_adrenal | H3K4me1 | -1.10 | 2.72 | 0.404583314 | Serous ovarian cancer | Adrenal_Pancreas |
| Placenta_amnion | H3K4me3 | 11.54 | 12.20 | 0.407960098 | Serous ovarian cancer | Other |
| Penis_foreskin_melanocyte_primary | H3K4me1 | 2.80 | 2.13 | 0.409381752 | Serous ovarian cancer | Other |
| CD34_primary | H3K4me1 | -2.07 | 4.69 | 0.41244296 | Serous ovarian cancer | Hematopoietic |
| CD4+_CD25-_Th_primary | H3K4me3 | 7.09 | 7.25 | 0.418057944 | Serous ovarian cancer | Hematopoietic |
| Adipose_nuclei | H3K9ac | -2.35 | 5.24 | 0.418639837 | Serous ovarian cancer | Other |
| Fetal_brain | H3K9ac | -4.81 | 8.97 | 0.423886471 | Serous ovarian cancer | CNS |
| CD19_primary_(UW) | H3K4me1 | 2.55 | 1.92 | 0.429164333 | Serous ovarian cancer | Hematopoietic |
| Fetal_adrenal | H3K4me3 | 11.48 | 13.65 | 0.451510981 | Serous ovarian cancer | Adrenal_Pancreas |
| Rectal_mucosa | H3K4me1 | 3.95 | 3.92 | 0.453084054 | Serous ovarian cancer | GI |
| CD14 | H3K27ac | -0.33 | 1.72 | 0.456579991 | Serous ovarian cancer | Hematopoietic |
| Mid_frontal_lobe | H3K4me1 | 11.28 | 13.97 | 0.457867541 | Serous ovarian cancer | CNS |
| Angular_gyrus | H3K4me1 | 5.04 | 5.37 | 0.459627953 | Serous ovarian cancer | CNS |
| CD4+_CD25int_CD127+_Tmem_primary | H3K4me3 | 8.46 | 10.07 | 0.460306603 | Serous ovarian cancer | Hematopoietic |
| Left_Ventricle | H3K4me1 | -1.40 | 3.15 | 0.461113547 | Serous ovarian cancer | Cardiovascular |
| CD8_naive_primary_(BI) | H3K4me3 | 6.89 | 7.90 | 0.462176698 | Serous ovarian cancer | Hematopoietic |
| Cingulate_gyrus | H3K27ac | 2.78 | 2.43 | 0.463214127 | Serous ovarian cancer | CNS |
| Skeletal_muscle | H3K4me3 | 4.58 | 4.73 | 0.464643199 | Serous ovarian cancer | SkeletalMuscle |
| Ovary | H3K4me1 | 7.24 | 9.14 | 0.468108652 | Serous ovarian cancer | Other |
| Duodenum_smooth_muscle | H3K27ac | 3.86 | 3.92 | 0.470392561 | Serous ovarian cancer | GI |
| Colonic_mucosa | H3K9ac | 6.66 | 7.94 | 0.471108939 | Serous ovarian cancer | GI |
| Substantia_nigra | H3K4me1 | 2.64 | 2.29 | 0.480534327 | Serous ovarian cancer | CNS |
| Stomach_mucosa | H3K4me1 | 3.06 | 2.92 | 0.486228871 | Serous ovarian cancer | GI |
| Inferior_temporal_lobe | H3K27ac | 2.67 | 2.39 | 0.48653007 | Serous ovarian cancer | CNS |
| CD19_primary_(BI) | H3K4me1 | 2.37 | 2.05 | 0.511104134 | Serous ovarian cancer | Hematopoietic |
| CD8_memory_primary | H3K4me3 | 6.19 | 7.93 | 0.51414595 | Serous ovarian cancer | Hematopoietic |
| Pancreatic_islets | H3K4me1 | 6.30 | 8.32 | 0.521376615 | Serous ovarian cancer | Adrenal_Pancreas |
| Fetal_trunk_muscle | H3K4me1 | 2.34 | 2.14 | 0.532055273 | Serous ovarian cancer | SkeletalMuscle |
| CD15_primary | H3K4me1 | -0.18 | 1.92 | 0.533023558 | Serous ovarian cancer | Hematopoietic |
| Stomach_smooth_muscle | H3K4me1 | -1.10 | 3.74 | 0.535184384 | Serous ovarian cancer | GI |
| Cingulate_gyrus | H3K4me3 | -2.14 | 5.45 | 0.550556696 | Serous ovarian cancer | CNS |
| Pancreatic_islets | H3K9ac | -7.22 | 14.35 | 0.555145352 | Serous ovarian cancer | Adrenal_Pancreas |
| Lung | H3K4me3 | 8.52 | 12.81 | 0.556119862 | Serous ovarian cancer | Cardiovascular |
| Esophagus | H3K4me3 | 7.66 | 11.18 | 0.556282759 | Serous ovarian cancer | GI |
| Stomach_smooth_muscle | H3K9ac | -3.24 | 8.96 | 0.56723547 | Serous ovarian cancer | GI |
| Breast_vHMEC | H3K4me3 | -5.36 | 13.41 | 0.569860812 | Serous ovarian cancer | Other |
| Left_Ventricle | H3K4me3 | -4.31 | 10.30 | 0.57657089 | Serous ovarian cancer | Cardiovascular |
| Mid_frontal_lobe | H3K27ac | 2.63 | 2.97 | 0.581893683 | Serous ovarian cancer | CNS |
| Right_atrium | H3K4me3 | 6.10 | 9.29 | 0.58416907 | Serous ovarian cancer | Cardiovascular |
| CD4+_CD25+_CD127-_Treg_primary | H3K4me3 | 5.02 | 7.54 | 0.585987187 | Serous ovarian cancer | Hematopoietic |
| Fetal_heart | H3K9ac | -1.10 | 4.07 | 0.590039939 | Serous ovarian cancer | Cardiovascular |
| Cingulate_gyrus | H3K9ac | -1.34 | 4.88 | 0.599386899 | Serous ovarian cancer | CNS |
| Angular_gyrus | H3K9ac | -2.67 | 7.95 | 0.6028517 | Serous ovarian cancer | CNS |
| CD8_naive_primary_(UCSF-UBC) | H3K4me3 | -5.96 | 16.59 | 0.607835991 | Serous ovarian cancer | Hematopoietic |
| CD19_primary_(UW) | H3K4me3 | 6.19 | 10.59 | 0.617532993 | Serous ovarian cancer | Hematopoietic |
| Spleen | H3K4me3 | 9.43 | 16.68 | 0.620064466 | Serous ovarian cancer | Hematopoietic |
| Peripheralblood_mononuclear_primary | H3K9ac | -3.15 | 10.34 | 0.621211444 | Serous ovarian cancer | Hematopoietic |
| Th2 | H3K27ac | 3.24 | 4.66 | 0.626537527 | Serous ovarian cancer | Hematopoietic |
| Penis_foreskin_keratinocyte_primary | H3K4me1 | 0.01 | 2.10 | 0.634230628 | Serous ovarian cancer | Other |
| Breast_fibroblast_primary | H3K4me3 | 10.39 | 20.76 | 0.638941969 | Serous ovarian cancer | Connective_Bone |
| CD56_primary | H3K4me1 | 2.20 | 2.60 | 0.642159254 | Serous ovarian cancer | Hematopoietic |
| Fetal_thymus | H3K4me1 | 0.06 | 2.32 | 0.65597339 | Serous ovarian cancer | Hematopoietic |
| Colon_smooth_muscle | H3K9ac | 8.92 | 18.82 | 0.656694041 | Serous ovarian cancer | GI |
| Peripheralblood_mononuclear_primary | H3K4me3 | 5.47 | 10.61 | 0.661066233 | Serous ovarian cancer | Hematopoietic |
| Fetal_lung | H3K9ac | 5.57 | 11.06 | 0.663582926 | Serous ovarian cancer | Cardiovascular |
| CD4+_CD25-_CD45R0+_memory_primary | H3K4me3 | 5.72 | 11.47 | 0.664525884 | Serous ovarian cancer | Hematopoietic |
| Inferior_temporal_lobe | H3K4me1 | 1.99 | 2.33 | 0.668003743 | Serous ovarian cancer | CNS |
| Inferior_temporal_lobe | H3K4me3 | -1.14 | 5.14 | 0.673946035 | Serous ovarian cancer | CNS |
| Fetal_heart | H3K4me3 | -3.37 | 11.58 | 0.684800389 | Serous ovarian cancer | Cardiovascular |
| Placenta_amnion | H3K4me1 | 6.01 | 12.61 | 0.688517043 | Serous ovarian cancer | Other |
| Penis_foreskin_keratinocyte_primary | H3K4me3 | 3.20 | 5.55 | 0.695221428 | Serous ovarian cancer | Other |
| Duodenum_Mucosa | H3K9ac | 3.82 | 7.79 | 0.703886146 | Serous ovarian cancer | GI |
| CD4_memory_primary | H3K4me3 | 4.15 | 8.81 | 0.704177644 | Serous ovarian cancer | Hematopoietic |
| Hippocampus_middle | H3K4me1 | 1.81 | 2.13 | 0.704325265 | Serous ovarian cancer | CNS |
| Hippocampus_middle | H3K9ac | -0.78 | 5.10 | 0.706616711 | Serous ovarian cancer | CNS |
| Penis_foreskin_keratinocyte_primary | H3K9ac | -0.23 | 3.37 | 0.707368809 | Serous ovarian cancer | Other |
| Substantia_nigra | H3K4me3 | 3.08 | 5.88 | 0.722889867 | Serous ovarian cancer | CNS |
| Anterior_caudate | H3K4me1 | 1.86 | 2.46 | 0.725786303 | Serous ovarian cancer | CNS |
| Mobilized_CD34_primary | H3K4me1 | 1.74 | 2.22 | 0.726241469 | Serous ovarian cancer | Hematopoietic |
| Psoas_muscle | H3K4me1 | 5.20 | 12.22 | 0.730540684 | Serous ovarian cancer | SkeletalMuscle |
| Gastric | H3K4me1 | 3.09 | 6.74 | 0.733530012 | Serous ovarian cancer | GI |
| Fetal_heart | H3K4me1 | 1.61 | 1.88 | 0.738824047 | Serous ovarian cancer | Cardiovascular |
| CD25-_IL17+_Th17_stim | H3K27ac | 2.54 | 4.71 | 0.740580555 | Serous ovarian cancer | Hematopoietic |
| Stomach_smooth_muscle | H3K4me3 | 3.15 | 6.79 | 0.743082713 | Serous ovarian cancer | GI |
| CD15_primary | H3K4me3 | -0.59 | 5.72 | 0.770351753 | Serous ovarian cancer | Hematopoietic |
| Cingulate_gyrus | H3K4me1 | 1.71 | 2.48 | 0.770551732 | Serous ovarian cancer | CNS |
| CD34_primary | H3K4me3 | 4.13 | 11.41 | 0.771569173 | Serous ovarian cancer | Hematopoietic |
| Fetal_brain | H3K4me1 | 0.53 | 1.68 | 0.774348866 | Serous ovarian cancer | CNS |
| Penis_foreskin_fibroblast_primary | H3K4me1 | 1.45 | 1.59 | 0.777188872 | Serous ovarian cancer | Connective_Bone |
| CD8_naive_primary_(UCSF-UBC) | H3K9ac | -7.52 | 36.50 | 0.778878139 | Serous ovarian cancer | Hematopoietic |
| Skeletal_muscle | H3K9ac | 0.02 | 4.03 | 0.788813471 | Serous ovarian cancer | SkeletalMuscle |
| CD14_primary | H3K4me1 | 1.53 | 2.01 | 0.791186305 | Serous ovarian cancer | Hematopoietic |
| Right_ventricle | H3K4me3 | 3.46 | 9.93 | 0.79722318 | Serous ovarian cancer | Cardiovascular |
| CD4+_CD25-_IL17+_PMA_Ionomycin_stim_Th17_primary | H3K4me3 | 2.90 | 8.00 | 0.800302657 | Serous ovarian cancer | Hematopoietic |
| Rectal_smooth_muscle | H3K4me1 | 2.70 | 6.91 | 0.804136984 | Serous ovarian cancer | GI |
| Anterior_caudate | H3K4me3 | -0.25 | 5.10 | 0.804777185 | Serous ovarian cancer | CNS |
| Adipose_nuclei | H3K27ac | 0.54 | 2.01 | 0.818030645 | Serous ovarian cancer | Other |
| Adipose_nuclei | H3K4me1 | 1.40 | 1.88 | 0.825313848 | Serous ovarian cancer | Other |
| Stomach_smooth_muscle | H3K27ac | 0.51 | 2.28 | 0.825652403 | Serous ovarian cancer | GI |
| CD8_primary | H3K4me3 | -0.86 | 10.15 | 0.831013776 | Serous ovarian cancer | Hematopoietic |
| CD19_primary_(BI) | H3K4me3 | 2.45 | 7.83 | 0.846562716 | Serous ovarian cancer | Hematopoietic |
| CD4_primary | H3K4me3 | 2.38 | 8.07 | 0.853540021 | Serous ovarian cancer | Hematopoietic |
| Fetal_placenta | H3K4me1 | 0.28 | 4.06 | 0.856564519 | Serous ovarian cancer | Other |
| Rectal_smooth_muscle | H3K9ac | 4.27 | 19.89 | 0.862143817 | Serous ovarian cancer | GI |
| CD19 | H3K27ac | 1.33 | 2.18 | 0.872385044 | Serous ovarian cancer | Hematopoietic |
| Peripheralblood_mononuclear_primary | H3K4me1 | 2.69 | 11.98 | 0.875655423 | Serous ovarian cancer | Hematopoietic |
| CD25int_CD127+_Tmem | H3K27ac | 1.41 | 2.93 | 0.883969555 | Serous ovarian cancer | Hematopoietic |
| Breast_luminal_epithelial | H3K4me1 | -2.49 | 24.21 | 0.884672724 | Serous ovarian cancer | Other |
| Hippocampus_middle | H3K4me3 | 1.74 | 5.18 | 0.885685486 | Serous ovarian cancer | CNS |
| CD14_primary | H3K4me3 | -0.55 | 12.61 | 0.889041796 | Serous ovarian cancer | Hematopoietic |
| Spleen | H3K4me1 | 0.69 | 2.36 | 0.894418495 | Serous ovarian cancer | Hematopoietic |
| Pancreatic_islets | H3K4me3 | 2.08 | 8.69 | 0.899508507 | Serous ovarian cancer | Adrenal_Pancreas |
| Penis_foreskin_fibroblast_primary | H3K4me3 | 0.65 | 3.20 | 0.909153275 | Serous ovarian cancer | Connective_Bone |
| CD3_primary_(UW) | H3K4me3 | 1.81 | 9.14 | 0.922762421 | Serous ovarian cancer | Hematopoietic |
| Substantia_nigra | H3K9ac | 1.49 | 5.31 | 0.924971214 | Serous ovarian cancer | CNS |
| Colonic_mucosa | H3K4me1 | 0.61 | 4.58 | 0.928124983 | Serous ovarian cancer | GI |
| Breast_fibroblast_primary | H3K4me1 | 0.45 | 6.21 | 0.928856807 | Serous ovarian cancer | Connective_Bone |
| CD56_primary | H3K4me3 | 1.84 | 13.54 | 0.943854138 | Serous ovarian cancer | Hematopoietic |
| Fetal_leg_muscle | H3K4me1 | 1.15 | 2.29 | 0.947403809 | Serous ovarian cancer | SkeletalMuscle |
| Aorta | H3K4me3 | 1.68 | 11.02 | 0.948050764 | Serous ovarian cancer | Cardiovascular |
| Duodenum_smooth_muscle | H3K4me1 | 0.14 | 14.81 | 0.948952297 | Serous ovarian cancer | GI |
| Inferior_temporal_lobe | H3K9ac | 0.76 | 5.46 | 0.963161728 | Serous ovarian cancer | CNS |
| Small_intestine | H3K4me1 | 2.29 | 32.81 | 0.968502341 | Serous ovarian cancer | GI |
| Skeletal_muscle | H3K4me1 | 1.05 | 1.86 | 0.975422165 | Serous ovarian cancer | SkeletalMuscle |
| Mobilized_CD34_primary | H3K4me3 | 0.82 | 6.83 | 0.976733396 | Serous ovarian cancer | Hematopoietic |
| CD20 | H3K27ac | 1.06 | 2.05 | 0.976841849 | Serous ovarian cancer | Hematopoietic |
| Osteoblast | H3K27ac | 1.05 | 2.25 | 0.981461299 | Serous ovarian cancer | Connective_Bone |
| Chondrogenic_dif | H3K27ac | 0.95 | 2.26 | 0.983011947 | Serous ovarian cancer | Connective_Bone |
| Fetal_stomach | H3K4me1 | 1.05 | 2.97 | 0.985152704 | Serous ovarian cancer | GI |
| Stomach_mucosa | H3K4me3 | 1.26 | 15.32 | 0.985577033 | Serous ovarian cancer | GI |
| Gastric | H3K4me3 | 1.23 | 13.50 | 0.986112656 | Serous ovarian cancer | GI |
| Anterior_caudate | H3K9ac | 1.06 | 6.42 | 0.992665689 | Serous ovarian cancer | CNS |
| Fetal_thymus | H3K4me3 | 0.96 | 8.70 | 0.995826274 | Serous ovarian cancer | Hematopoietic |
| Bold font: P < 0.05/220; GI: gastrointestinal; CNS: central nervous system. When the same cell type in the same histone mark from more than one institution was used, the name of institution is given in parentheses. | | | | | | |
