# Supplementary material for: Shared heritability and functional enrichment across six solid cancers
Source: Nat Commun. 2019 Jan 25;10:431. doi: 10.1038/s41467-018-08054-4 (PMC6347624; doi:10.1038/s41467-018-08054-4)
Supplement: Supplementary file 8 — Reporting Summary [file 41467_2018_8054_MOESM8_ESM.pdf]

nature research

Corresponding author(s): Sara Lindstroem, Xia Jiang, Peter Kraft

Reporting Summary

Nature Research wishes to improve the reproducibility of the work that we publish. This form provides structure for consistency and transparency in reporting. For further information on Nature Research policies, see Authors & Referees and the Editorial Policy Checklist.

Statistical parameters

When statistical analyses are reported, confirm that the following items are present in the relevant location (e.g. figure legend, table legend, main text, or Methods section).

n/a	Confirmed	
☐	☒	The exact sample size (*n*) for each experimental group/condition, given as a discrete number and unit of measurement
☒	☐	An indication of whether measurements were taken from distinct samples or whether the same sample was measured repeatedly
☐	☒	The statistical test(s) used AND whether they are one- or two-sided<br>*Only common tests should be described solely by name; describe more complex techniques in the Methods section.*
☒	☐	A description of all covariates tested
☐	☒	A description of any assumptions or corrections, such as tests of normality and adjustment for multiple comparisons
☐	☒	A full description of the statistics including central tendency (e.g. means) or other basic estimates (e.g. regression coefficient) AND variation (e.g. standard deviation) or associated estimates of uncertainty (e.g. confidence intervals)
☐	☒	For null hypothesis testing, the test statistic (e.g. *F*, *t*, *r*) with confidence intervals, effect sizes, degrees of freedom and *P* value noted<br>*Give P values as exact values whenever suitable.*
☒	☐	For Bayesian analysis, information on the choice of priors and Markov chain Monte Carlo settings
☒	☐	For hierarchical and complex designs, identification of the appropriate level for tests and full reporting of outcomes
☒	☐	Estimates of effect sizes (e.g. Cohen's *d*, Pearson's *r*), indicating how they were calculated
☐	☒	Clearly defined error bars<br>*State explicitly what error bars represent (e.g. SD, SE, CI)*

Our web collection on statistics for biologists may be useful.

Software and code

Policy information about availability of computer code

Data collection	N.A.
Data analysis	LD score regression (LDSC) v1.0.0, https://github.com/bulik/ldsc<br>gwas-pw version 0.21, https://github.com/joepickrell/gwas-pw

For manuscripts utilizing custom algorithms or software that are central to the research but not yet described in published literature, software must be made available to editors/reviewers upon request. We strongly encourage code deposition in a community repository (e.g. GitHub). See the Nature Research guidelines for submitting code & software for further information.

Data

Policy information about availability of data

All manuscripts must include a data availability statement. This statement should provide the following information, where applicable:

- Accession codes, unique identifiers, or web links for publicly available datasets
- A list of figures that have associated raw data
- A description of any restrictions on data availability

The datasets generated during and/or analyzed during the current study are available from the authors on request.
Breast cancer: Summary results for all variants are available at http://bcac.ccge.medschl.cam.ac.uk/. Requests for further data should be made through the Data

Access Coordination Committee (http://bcac.ccge.medschl.cam.ac.uk/).
Ovarian cancer: Summary results are available from the Ovarian Cancer Association Consortium (OCAC) (http://ocac.ccge.medschl.cam.ac.uk/). Requests for further data can be made to the Data Access Coordination Committee (http://cimba.ccge.medschl.cam.ac.uk/).
Prostate cancer: Summary results are publicly available at the PRACTICAL website (http://practical.icr.ac.uk/blog/).
Lung cancer: Genotype data for lung cancer are available at the database of Genotypes and Phenotypes (dbGaP) under accession phs001273.v1.p1. Readers interested in obtaining a copy of the original data can do so by completing the proposal request form at http://oncoarray.dartmouth.edu/
Head / neck cancer: Genotype data for the oral and pharyngeal OncoArray study have been deposited at the database of Genotypes and Phenotypes (dbGaP) under accession phs001202.v1.p1.
Colorectal cancer: Genotype data have been deposited at the database of Genotypes and Phenotypes (dbGaP) under accession number phs001415.v1.p1 and phs001078.v1.p1.

Field-specific reporting

Please select the best fit for your research. If you are not sure, read the appropriate sections before making your selection.

☒ Life sciences ☐ Behavioural & social sciences ☐ Ecological, evolutionary & environmental sciences

For a reference copy of the document with all sections, see nature.com/authors/policies/ReportingSummary-flat.pdf

Life sciences study design

All studies must disclose on these points even when the disclosure is negative.

Sample size	We used summary statistics from six cancer GWASs based on a total of 597,534 participants of European ancestry. Cancer-specific sample sizes were: breast cancer: 122,977 cases / 105,974 controls; colorectal cancer: 36,948 / 30,864; head/neck cancer (oral and oropharyngeal cancers): 5,452 / 5,984; lung cancer: 29,266 / 56,450; ovarian cancer: 22,406 / 40,941; prostate cancer: 79,166 / 61,106.
Data exclusions	We included autosomal SNPs with a minor allele frequency (MAF) larger than 1% and present in HapMap3 (NSNPs = ~1 million) because those SNPs are usually well imputed in most studies (note that excluding sex chromosomes could reduce the overall heritability estimates).
Replication	NA
Randomization	NA
Blinding	The investigators were blinded during the data collection and analysis stage.

Reporting for specific materials, systems and methods

Materials & experimental systems

n/a	Involved in the study
☒	☐ Unique biological materials
☒	☐ Antibodies
☒	☐ Eukaryotic cell lines
☒	☐ Palaeontology
☒	☐ Animals and other organisms
☐	☒ Human research participants

Methods

n/a	Involved in the study
☒	☐ ChIP-seq
☒	☐ Flow cytometry
☒	☐ MRI-based neuroimaging

Human research participants

Policy information about studies involving human research participants

Population characteristics	Individual of European populations; both men and women.
Recruitment	We did not recruit any individuals, we used the summary-level GWAS data generated based on European ancestry populations.
