# Supplementary material for: Shared heritability and functional enrichment across six solid cancers
Source: Nat Commun. 2019 Jan 25;10:431. doi: 10.1038/s41467-018-08054-4 (PMC6347624; doi:10.1038/s41467-018-08054-4)
Supplement: Supplementary file 9 — Peer Review Files [file 41467_2018_8054_MOESM9_ESM.pdf]

Reviewers' comments:

Reviewer #1 (Remarks to the Author):

Using summary statistics from GWAS, the study quantified the genetic correlations among cancers, their subtypes, as well as with other non-cancer traits. They also assessed the proportion of cancer heritability attributable to specific functional categories to identify functional elements that are enriched for SNP-heritability. Overall the study is well conducted. I have a few comments for the authors to consider.
1. The study can be viewed as a cross-sectional study on the genetic levels and has limited ability for inferring causality (ie, the shared roots of genetic causes). Thus, it is unclear what information the study adds to what we have already known about the shared risk factors among many cancers (eg, smoking for lung and head and neck cancer) and the causal relationship b/t certain traits and cancer (eg, obesity and colorectal cancer). What is even more concerning is that some of the well-known relationships is not supported by the current study (eg, reproductive factors and breast cancer), raising questions about the study methodology (eg, reliance on the GWAS findings and lack of consideration of rare loci). Also, as acknowledged by the authors, even the observed genetic correlations between traits are subject to confounding by other common risk factors.
2. The first part of the results on heritability estimates is very interesting. It is surprising to see that common GWAS loci can almost entirely explain the classical heritability of head/neck cancer and explain 30-40% of heritability for other cancers. This does not seem to be consistent with the literature about the limited heritability that GWAS loci explains for most cancers, and deserves some mention in the abstract and detailed discussion. If the current study finding is correct, however, we would not expect much contradiction with the Mendelian randomization studies about the relationship b/t risk factors and common cancers since the MR studies are completely based on the identified GWAS loci.
3. The selection criteria for non-cancer traits are unclear. While most of the traits can be considered as cancer risk factors, others are more symptoms-related (eg, lung function for lung cancer, psychiatric factors). On the other hand, other well-established cancer risk factors are not considered, such as infection. A more structured, hypothesis-driven selection process may be considered to clarify the aims of the investigation and facilitate downstream inference.

Reviewer #2 (Remarks to the Author):

This study presents the largest analysis of genetic correlations among different solid cancers. While this type of study is not novel, the very large increase in sample size has enabled detection of a large number of shared genetic effects that will potentially lead to important advances in this field. The study relies primarily on LD score regression that has some advantages over other approaches to examine GWAS signal correlations. In addition, interesting local genetic correlations as well as analyses of enrichment of cancer heritability due to epigenetic and other putative functional elements are valuable additions. Overall, the study the presentation is balanced and well written.

Some suggestive clarifications.
1. Methods – Some additional details concerning QA should be included within the methods and not simply referenced. These include indicating briefly the computational algorithm for imputing, quality of imputed SNPs (r2 or other criteria), and any other data cleaning (HW exclusion etc.). Also, it unclear whether these studies used a final common SNP set (number of SNPs should be indicated) among the solid cancers and between these cancers and other non-cancer traits. If not, the final SNP number should be included for each component. If a common set of SNPs was not used some discussion about how this would affect the analyses should be considered.
a. It is not clear to me whether the LDSR can distinguish between opposite effects (protective vs. risk) when comparing phenotypes. This might be worth a comment.

2. It would be useful to provide in a supplemental Table the number of regions (+/- 500 kb) for each cancer that reach the 5x10-8 threshold (p values) and some measure of effect size. This would be of value in providing additional context for the contribution of known loci to the h2g calculations (e.g. differences between head and neck vs. lung cancer).
3. It would be interesting to see whether combinations of identified GWAS loci/regions (5x10-8 +/- 500 kb) in different solid cancers could explain a higher proportion of the GWAS calculated h2g. For example, can the lung cancer 5x10-8 GWAS loci explain a proportion of the head and neck GWAS calculated h2. Perhaps excluding these regions would be more informative in assessing whether how much of the overlap is due mostly to large numbers of unidentified GWAS loci (i.e. how is the genetic sharing affected if the 5x10-8 regions are excluded?).
4. Some comment concerning whether population structure differences between studies of different European populations (i.e. potential for differences in population groups between different cancers) and how this could affect LDSC.
5. Some empiric assessment of the sensitivity of the LD regression analysis to sample size or more importantly, study origin, affect the analyses. For example, do smaller sets of squamous lung cancer derived from different studies show approach sharing of 1.0. Do these smaller squamous lung cancer sets show similar rg with adenocarcinoma.
6. Last sentence in abstract is a bit strong – would suggest "...... suggests that solid tumors arising across tissues in part share a common germline genetic basis."

Reviewer #1:

Using summary statistics from GWAS, the study quantified the genetic correlations among cancers, their subtypes, as well as with other non-cancer traits. They also assessed the proportion of cancer heritability attributable to specific functional categories to identify functional elements that are enriched for SNP-heritability. Overall the study is well conducted. I have a few comments for the authors to consider.

1. The study can be viewed as a cross-sectional study on the genetic levels and has limited ability for inferring causality (eg, the shared roots of genetic causes). Thus, it is unclear what information the study adds to what we have already known about the shared risk factors among many cancers (eg, smoking for lung and head and neck cancer) and the causal relationship b/t certain traits and cancer (eg, obesity and colorectal cancer). What is even more concerning is that some of the well-known relationships is not supported by the current study (eg, reproductive factors and breast cancer), raising questions about the study methodology (eg, reliance on the GWAS findings and lack of consideration of rare loci). Also, as acknowledged by the authors, even the observed genetic correlations between traits are subject to confounding by other common risk factors.

Response: We thank the reviewer for raising these potential concerns. The main purpose of our manuscript is to understand the magnitude of genetic correlation among cancers, which may have important implications for the biology and future study design (e.g., would a pan-cancer GWAS make sense?). We note that comprehensive Mendelian randomization analyses between non-cancer traits and the included cancers are beyond the scope of our current study, and are topics of ongoing research projects of other groups within the same network.

In this study, we explored the relationship between potential risk factors and cancer using two different approaches. We first conducted genome-wide genetic correlation analyses which take all common SNPs in the genome into account, regardless of their statistical significance. In addition, we assessed the directional genetic correlations between potential risk factors and cancers, which could also provide causal interpretations (see Figure 4 in the original manuscript). Such analyses are based on genome-wide significant loci only and are unfortunately not feasible for traits with limited number of GWAS-identified SNPs such as smoking (only two loci have been identified).

In our directional genetic correlation analysis, we *did* find that SNPs associated with age at natural menopause showed correlated effect estimates with breast cancer but the reverse was not true; previous Mendelian randomization analyses have also identified a causal link between reproductive factors (e.g., age at menarche) and breast cancer, whereas we did not observe a significant genetic correlation using SNPs all over the genome. The lack of genome-wide correlation could be due to the sparse nature of the correlation, and the sample size.

We thus argued in our discussion that "…… *It is possible that a relatively small overlap in strongly associated SNPs can result in significant MR results despite low evidence of an overall genetic correlation. ……*"

2. The first part of the results on heritability estimates is very interesting. It is surprising to see that common GWAS loci can almost entirely explain the classical heritability of head/neck cancer and explain 30-40% of heritability for other cancers. This does not seem to be consistent with the literature about the limited heritability that GWAS loci explains for most cancers, and deserves some mention in the abstract and detailed discussion. If the current study finding is correct, however, we would not expect much contradiction with the Mendelian randomization studies about the relationship b/t risk factors and common cancers since the MR studies are completely based on the identified GWAS loci.

Response: We thank the reviewer for this opportunity to further clarify our results. If we define the classical twin studies or familial studies narrow sense heritability as h^2, heritability explained by common SNPs (SNPs across the whole genome) or SNP-heritability as h_g^2, and heritability explained by known GWAS loci (significant

GWAS SNPs) as h^2_{GWAS}, indeed, the classical heritability can be explained almost entirely (head/neck cancer) or 30-40% (other cancers) by using all SNPs across the genome (h^2_g), but not the GWAS-identified significant loci (h^2_{GWAS}).

These numbers are consistent across multiple common traits. For example, the classical heritability (h^2) of rheumatoid arthritis is ~0.5 and genome-wide SNPs can explain almost half of this heritability (h^2_g, 0.2 out of 0.5, 40%), whereas genome-wide significant SNPs could only explain 12% (h^2_{GWAS}, 0.06 out of 0.5). Table 1 below was cited from Visscher *et al.* (PMID 22243964), where for most of the traits, all GWAS SNPs explain a majority (30-60%) of the classical heritability.

We note that head /neck cancer is a special case because of estimate variability. In our current study, the sample size of head/neck is the smallest among all cancers (N=5,452 cases and 5,984 controls), and its SNP-heritability on the liability scale varies between 5-14% (point estimate 9%). Similarly, the largest available twin study of head/neck cancer, to which we compared our SNP-heritability, is based on only 196 monozygotic and 367 dizygotic twins, and the heritability varies between 0-60% (point estimate 9%). Comparing the point estimates, it seems that the classical heritability can be explained almost entirely for head/neck cancer, which may be influenced by limited sample size and power. We have explicitly mentioned the uncertainty of point estimates as a limitation in our discussion.

We note that Mendelian randomization analysis to explore if risk factor A is associated with cancer B will be based on the SNPs found to be genome-wide significantly associated with risk factor A and not with cancer B. Thus, the SNPs that are genome-wide significantly associated with cancer B are not considered in Mendelian randomization (unless they have shown genome-wide significance for both risk factor A and cancer B). Therefore, it is possible that even though common SNPs can explain a high proportion of the heritability of cancer, the individual GWAS SNPs for risk factor A, may only explain a very small part of the observed SNP-heritability for cancer.

Table 1. Population Variation Explained by GWAS for a Selected Number of Complex Traits

Trait or Disease	h^2 Pedigree Studies	h^2 GWAS Hits[a]	h^2 All GWAS SNPs[b]
Type 1 diabetes	0.9[98]	0.6[99,c]	0.3[12]
Type 2 diabetes	0.3–0.6[100]	0.05-0.10[34]	
Obesity (BMI)	0.4–0.6[101,102]	0.01-0.02[36]	0.2[14]
Crohn's disease	0.6–0.8[103]	0.1[11]	0.4[12]
Ulcerative colitis	0.5[103]	0.05[12]	
Multiple sclerosis	0.3–0.8[104]	0.1[45]	
Ankylosing spondylitis	>0.90[105]	0.2[106]	
Rheumatoid arthritis	0.6[107]		
Schizophrenia	0.7–0.8[108]	0.01[79]	0.3[109]
Bipolar disorder	0.6–0.7[108]	0.02[79]	0.4[12]
Breast cancer	0.3[110]	0.08[111]	
Von Willebrand factor	0.66–0.75[112,113]	0.13[114]	0.25[14]
Height	0.8[115,116]	0.1[13]	0.5[13,14]
Bone mineral density	0.6-0.8[117]	0.05[118]	
QT interval	0.37–0.60[119,120]	0.07[121]	0.2[14]
HDL cholesterol	0.5[122]	0.1[57]	
Platelet count	0.8[123]	0.05–0.1[58]	

3. The selection criteria for non-cancer traits are unclear. While most of the traits can be considered as cancer risk factors, others are more symptoms-related (eg, lung function for lung cancer, psychiatric factors). On the other hand, other well-established cancer risk factors are not considered, such as infection. A more structured, hypothesis-driven selection process may be considered to clarify the aims of the investigation and facilitate downstream inference.

Response: We thank the reviewer for raising this point. The purpose of the genetic correlation analyses between non-cancer traits and cancer was two-fold. First, we wanted to quantify the genetic correlation between established risk factors (such as smoking, obesity), but we also wanted to conduct exploratory analyses to discover novel relationships. Our non-cancer traits were selected based on data availability and is mostly hypothesis free. We collected GWAS summary statistics from UK Biobank as well as other publically available GWAS summary results. Among those traits, we calculated trait-specific SNP-heritability and restricted our analysis only to traits with a heritable component (z-score > 7). This quality control procedure may preclude some of the traits (e.g., with a small sample size, or no signs of heritability) from being included in our study.

We have explicitly mentioned this as a limitation in our discussion, it reads, "…… *We were not able to consider all cancer risk factors when selecting non-cancer traits, since some of the well-established risk factors such as infection were either not available, showed no evidence of heritability or were not based on adequate sample sizes for robust analyses. ……"*

Reviewer #2:

This study presents the largest analysis of genetic correlations among different solid cancers. While this type of study is not novel, the very large increase in sample size has enabled detection of a large number of shared

genetic effects that will potentially lead to important advances in this field. The study relies primarily on LD score regression that has some advantages over other approaches to examine GWAS signal correlations. In addition, interesting local genetic correlations as well as analyses of enrichment of cancer heritability due to epigenetic and other putative functional elements are valuable additions. Overall, the study the presentation is balanced and well written.

Thank you.

Some suggestive clarifications.

1. Methods – Some additional details concerning QA should be included within the methods and not simply referenced. These include indicating briefly the computational algorithm for imputing, quality of imputed SNPs (r^2 or other criteria), and any other data cleaning (HW exclusion etc.). Also, it unclear whether these studies used a final common SNP set (number of SNPs should be indicated) among the solid cancers and between these cancers and other non-cancer traits. If not, the final SNP number should be included for each component. If a common set of SNPs was not used some discussion about how this would affect the analyses should be considered.

a. It is not clear to me whether the LDSR can distinguish between opposite effects (protective vs. risk) when comparing phenotypes. This might be worth a comment.

Response: Following the reviewer's suggestion, we have summarized the additional details of quality control in Supplement 1, including the computational algorithm for imputation, the reference panel, quality of imputed SNPs, and data cleaning strategy. This table has also been included in our manuscript. For more details, we encourage the reader to look into the original GWAS paper of each cancer (Michailidou *et al.*, PMID 29059683; McKay *et al.*, PMID 28604730; Lesseur *et al.*, PMID 27749845; Schmit *et al.*, PMID 29917119; Phelan *et al.*, PMID 28346442; Schumacher *et al.*, PMID 29892016).

For all our SNP-heritability and genetic correlation analysis, we used a final common SNP set. We have further clarified this in our Methods, "…… *We included autosomal SNPs with a minor allele frequency (MAF) larger than 1% and present in HapMap3 because those SNPs are usually well imputed in most studies (N_{SNPs} = ~1 million). ……*".

LDSC can distinguish between concordant effects—where the same allele is associated with an increase in both traits being compared—and opposite effects—where the same allele is associated with an increase in one trait but a decrease in the other. LDSC calculates the correlation in regression coefficients for the two traits across all SNPs. This genetic correlation ranges between –1 to 1, so LDSC can distinguish opposite effects (positive or negative genetic correlation).

In a previous paper published by Bulik-Sullivan *et al.* (PMID 26414676), several negative genetic correlations have been observed between traits such as anorexia nervosa and obesity, height and coronary artery disease, college attendance and Alzheimer's disease, smoking and college attendance, and are consistent with epidemiological reports. We have also identified, in our current manuscript, negative genetic correlations of educational attainment with multiple cancers.

This is also one of the reasons why it's possible to observe multiple regions that show local genetic correlation between two traits even though the overall genome-wide genetic correlation is minimal (e.g., lung and prostate cancer, see Figure 2 in the original manuscript). Because negative and positive significant local genetic correlations between two traits will cancel out in the overall genetic correlation and LDSC is sensitive to this.

Supplement1. Quality control and imputation procedures of each cancer.

Cancer type	Imputation algorithm	Imputation reference	Included SNPs for	Other data cleaning strategy

		panel	association	
**Breast**	Part of the data used SHAPEIT for phasing and IMPUTEv2 for imputation; Part of the data used MACH and Minimac for imputation.	The October 2014 (version 3) release of the 1000 Genomes Project dataset	Imputation r-square > 0.30	SNPs with a call rate <95% in any consortium, SNPs not in Hardy-Weinberg equilibrium ($P<10^{-7}$ in controls or $P <10^{-12}$ in cases) and SNPs with concordance <98% among duplicate sample pairs were excluded. For the imputation, SNPs with a MAF <1% and a call rate <98% in any consortium, SNPs that could not be linked to the 1000 Genomes Project reference or differed significantly in frequency from the 1000 Genomes Project dataset were additionally excluded. A further 1,128 SNPs where the cluster plot was judged to be not ideal on visual inspection were excluded.
**Colorectal**	SHAPEIT for phasing and IMPUTE2v2 for imputation.	The October 2014 (version 3) release of the 1000 Genomes Project dataset	Imputation info≥0.7, certainty ≥0.9, concordance ≥0.9 for directly measured markers as well as a MAF filter of ≥0.01	Standard QC filters were applied. A first round of filtering excluded samples with <80% call rate, then variants with <80% call rate. Next, samples with <95% call rate were excluded as well as those marked for removal from various QC checks such as replicate concordance (within and across platforms), unexpected replicate search (within and across platforms), genotyped vs. reported sex concordance, plate mix-ups, and removal due to lack of consent. Markers were then excluded based on the following criteria: 1) <95% call rate; 2) duplicate error rate >1% (only in matching reps with call rate >=99%) or heterozygote duplicate error rate >5% and >2 het mismatches, and 3) SNPs with duplicate probes.
**Head and neck**	SHAPEIT for phasing, and Minimac3 for imputation	The Haplotype Reference Consortium panel	Imputation r-square > 0.30	An initial filtering step on the complete dataset excluded samples with genotyping rate <80% and SNPs with call rate <80%. During the individuals QC, samples with unsolved genetic and reported sex discrepancies and individuals with outlying autosomic heterozygosity rate were removed. Identity-by-descent (IBD) analysis performed on the LD-pruned dataset identified 103 expected experimental duplicate-pairs (IBD > 0.9), from these the sample with lower genotyping rate were excluded. Additionally, 44 unexpected relative pairs (IBD > 0.3) were identified and excluded. SNPs with deviation of Hardy-Weinberg Equilibrium in controls ($P < 1x10^{-7}$) were further excluded.
**Lung**	SHAPEIT for phasing and IMPUTE2v2 for imputation.	The October 2014 (version 3) release of the 1000 Genomes Project dataset	Imputation r-square > 0.30 and info > 0.40	Standard quality control procedures were used to exclude underperforming individuals (DNAs) and genotyping assays (judged by success rate, genotype distributions deviated from that expected by Hardy Weinberg equilibrium). Samples were subjected to genotype calling rate and individual calling rate check. 1,708 individuals were removed for call rate less than 95%, and 16,149 SNPs with call rates of less than 95% were removed. After filtering, there were 517,482 SNPs available for analysis. We applied the standard OncoArray consortium filter for removing SNPs if they showed departure from Hardy-Weinberg equilibrium in the controls (P-value $<1 \times 10^{-7}$) or cases (P-value $<1 \times 10^{-12}$).
**Ovarian**	SHAPEIT for phasing and IMPUTE2v2 for imputation.	The October 2014 (version 3) release of the 1000 Genomes Project dataset	Imputation r-square > 0.30 and MAF > 0.01.	Samples were excluded if they had a genotyping call rate <95%, if they had excessively low or high heterozygosity, if they were not female or if they were duplicates. Duplicates and close relatives were identified using in-house software that calculates a concordance matrix for all individuals. SNP quality control was carried out according to the OncoArray QC Guidelines. Only SNPs that passed quality control for all consortia were used for imputation. SNPs with a call rate <95%, SNPs deviating from Hardy-Weinberg equilibrium ($P < 1 \times 10^{-7}$ in controls or unrelated samples in CIMBA and $P < 1 \times 10^{-12}$ in cases) and SNPs with concordance <98% among duplicate pairs were excluded.
**Prostate**	SHAPEIT for phasing and IMPUTE2v2 for imputation.	The October 2014 (version 3) release of the 1000 Genomes Project dataset	Imputation r-square > 0.30	Variants likely to have problematic clusters were selected for manual inspection on the basis of the following criteria: call rate <99%, MAF <0.001, poor Illumina intensity and clustering metrics, deviation from the MAF observed in the 1KGP. SNPs with a call rate <95% by study, not in Hardy-Weinberg equilibrium ($P < 10^{-7}$ in controls or $P < 10^{-12}$ in cases) or with concordance <98% among duplicate pairs were further excluded.

2. It would be useful to provide in a supplemental Table the number of regions (+/- 500 kb) for each cancer that reach the 5×10^{-8} threshold (p-values) and some measure of effect size. This would be of value in providing

additional context for the contribution of known loci to the h^2_g calculations (e.g. differences between head and neck vs. lung cancer).

Response: We agree with the reviewer, and have listed the specific regions for each cancer that reached the 5×10^{-8} threshold (p-values), its chromosome, start and end positions (+/– 500 kb), and the most significant SNP (referred to as the "best SNP") of that region in the Supplement 2 below. This table has also been included in our manuscript.

Supplement2. The number of regions (+/- 500 kb) for each cancer that reach the $5x10^{-8}$ threshold (p-values) in each cancer and the best SNP in the region.

Chromosome	Region start	Region end	Best SNP	Position	Z-score	Combined P-value
**Breast cancer**						
1	10011589	11138604	rs2506889	10596022	-9.32	1.71E-20
1	18299285	19311668	rs2992756	18807339	8.00	1.58E-15
1	40880440	42640601	rs4233486	41380440	5.77	6.60E-09
1	46100917	47103348	rs1707302	46600917	-5.62	2.46E-08
1	50329879	51360499	rs145905639	50829879	-5.53	2.92E-08
1	87656923	88929024	rs17426269	88156923	5.64	1.70E-08
1	113673410	114951386	rs7513707	114445880	6.77	1.33E-11
1	117695233	118758703	rs7529522	118230221	-6.37	1.54E-10
1	119758970	121977667	rs11249433	121280613	-15.20	1.34E-52
1	145088184	146213071	rs36107432	145604791	-6.40	1.93E-10
1	149392872	150495265	rs11205303	149906413	-7.77	1.05E-14
1	154643768	156171678	rs4971059	155148781	6.66	3.22E-11
1	200937832	201937832	rs35383942	201437832	7.25	3.68E-13
1	203266395	204447358	rs59867004	203801249	-6.46	1.19E-10
1	216700580	217722804	rs11117758	217220574	-5.92	3.10E-09
1	241523898	242547847	rs72755295	242034263	-7.69	1.46E-14
2	9594526	10646757	rs113577745	10135681	-6.27	3.33E-10
2	18768232	19927429	rs11684853	19310918	-7.19	6.13E-13
2	24577856	25973311	rs6725517	25129473	7.00	2.25E-12
2	120576438	121764471	rs4848599	121239360	-9.31	1.35E-20
2	171869881	173474566	rs2016394	172972971	-6.82	6.78E-12
2	173698854	174719118	rs2010610	174210908	-6.29	3.75E-10
2	201614624	202867589	rs3769821	202123430	-8.74	2.04E-18
2	217223569	219239300	rs4442975	217920769	-20.89	2.57E-96
2	226711914	227743909	rs12479355	227226952	5.61	2.13E-08
3	4228008	5296807	rs6787391	4728574	8.81	1.03E-18
3	26531928	28149948	rs7626742	27268398	16.90	2.90E-64
3	30167425	31189421	rs17838698	30684907	7.67	1.67E-14
3	46246022	47407435	rs56387622	46888198	8.95	4.64E-19
3	63328780	64520447	rs3821902	63941697	-7.02	2.29E-12
3	71017527	72044614	rs6805189	71532113	5.50	3.21E-08
3	86537543	87537543	rs13066793	87037543	6.12	9.71E-10
3	98875151	100342140	rs9833888	99723580	6.26	4.70E-10
3	140533481	141836708	rs7650602	141147414	-7.75	5.60E-15
3	171770437	172794031	rs58058861	172285237	6.41	1.72E-10
4	38263103	39394380	rs6815814	38816338	-7.25	4.27E-13
4	83864808	84956142	rs9284657	84419143	-5.87	3.58E-09
4	88740476	89746214	rs10022462	89243818	6.06	1.30E-09
4	105563987	106856761	rs62331150	106069013	6.81	1.15E-11

4	126343504	127343504	rs77528541	126843504	-6.11	9.35E-10
4	175318396	176425281	rs7697216	175828036	-10.70	1.27E-26
5	-185065	1800070	rs2853669	1295349	9.42	3.98E-21
5	15695004	16785704	rs4702131	16233619	6.87	6.88E-12
5	32067732	33081186	rs12519859	32581186	5.92	4.35E-09
5	43645931	46903779	rs10941679	44706498	-18.21	3.40E-73
5	49141645	50760139	rs27279	50238519	-6.57	3.86E-11
5	55412533	56829225	rs62355901	56053535	-21.18	3.14E-99
5	57684061	58895679	rs1498608	58343067	-6.28	2.87E-10
5	80615839	82110680	rs4081859	81466669	7.21	6.67E-13
5	90153868	91290451	rs332529	90789470	-6.86	8.63E-12
5	110614379	111717786	rs6882649	111217786	5.92	2.57E-09
5	131875335	132944509	rs56083805	132442263	5.82	6.20E-09
5	157564697	158946223	rs11135046	158230013	-11.58	2.52E-31
5	169041551	170091487	rs4562056	169591487	6.21	4.00E-10
6	13138243	14245518	rs418053	13713366	-7.52	8.52E-14
6	15899557	16899557	rs3819405	16399557	-5.67	1.25E-08
6	20036748	21206418	rs2223621	20621238	6.38	2.47E-10
6	25675866	29856687	rs34546498	26961280	-6.13	7.40E-10
6	80572063	82887139	rs9361840	82254932	-7.53	5.00E-14
6	129828639	130903515	rs6569648	130349119	7.05	1.75E-12
6	151316054	152974790	rs60954078	151955914	-15.60	4.07E-55
7	21440960	22440960	rs7971	21940960	5.58	1.98E-08
7	27856889	28856889	rs17156577	28356889	-5.91	3.74E-09
7	90917816	92529302	rs3753107	91629151	-6.60	3.60E-11
7	93559899	94804344	rs17268829	94113799	-7.29	3.51E-13
7	101000996	102068195	rs71559437	101552440	-6.93	4.28E-12
7	130127014	131190824	rs61729633	130668912	7.20	4.43E-13
7	139437791	140458544	rs11977670	139942304	8.29	9.76E-17
7	143548902	144639419	rs62485509	144048902	-6.90	3.96E-12
8	28917238	30030479	rs9693444	29509616	9.62	1.34E-21
8	36158914	37359186	rs4286946	36849946	9.64	5.51E-22
8	75668870	77189287	rs72658071	76305785	-11.09	1.09E-28
8	101975114	102983100	rs514192	102478959	5.92	3.95E-09
8	105821126	106872180	rs12546444	106358620	6.50	7.22E-11
8	116667843	117709548	rs13267382	117209548	6.74	1.19E-11
8	124059709	125257661	rs58847541	124610166	7.21	4.33E-13
8	127773489	129724888	rs10096351	128372172	-17.05	4.97E-65
9	21449527	22603183	rs1985742	21961227	-9.23	4.12E-20
9	109797639	111573347	rs630965	110885479	15.50	1.51E-54
9	118655568	119988626	rs1895062	119313486	7.78	8.60E-15
9	128883199	129896434	rs10760444	129396434	-5.82	5.98E-09
9	135641870	136655000	rs507666	136149399	5.58	2.73E-08
10	8576366	9628818	rs67801543	9108324	6.02	1.79E-09
10	21189036	23415712	rs7072776	22032942	9.12	1.25E-19
10	63389801	65352335	rs10995201	64299890	15.15	6.79E-52
10	80310343	81392739	rs1268974	80852378	12.62	1.38E-36
10	114232882	115764973	rs12250948	115128491	7.82	5.94E-15
10	122591543	124306607	rs34032268	123341525	-37.49	0.00E+00
11	280827	1325110	rs6597981	803017	-7.05	1.34E-12
11	1371813	2539274	rs1973765	1898664	12.98	4.78E-38
11	64993112	66183531	rs3903072	65583066	-7.02	1.59E-12

11	68422043	70009669	rs78540526	69331418	24.42	2.34E-132
11	128952507	129976625	rs11822830	129461016	-7.70	1.25E-14
12	13880438	14922475	rs12422552	14413931	7.91	2.22E-15
12	27517188	29774538	rs7297051	28174817	-16.47	1.73E-60
12	84498818	85509562	rs10862899	85004551	5.48	3.91E-08
12	95521033	96538543	rs17356907	96027759	13.16	8.43E-40
12	114602482	116338648	rs2464264	115835798	-13.11	8.46E-40
12	120332146	121336293	rs1167362	120836293	-5.60	2.24E-08
13	32339990	33339990	rs56404467	32839990	6.54	6.31E-11
13	73306982	74467507	rs6562760	73957681	-6.10	1.10E-09
14	36518022	37779425	rs7149262	37136545	-9.53	1.82E-21
14	67862510	69572051	rs11624333	68979835	13.68	2.11E-42
14	91242924	92490948	rs941764	91841069	-7.09	8.68E-13
14	92569980	93618229	rs117068593	93118229	-7.08	1.30E-12
14	104713978	105717921	rs4983544	105213978	-5.60	2.42E-08
15	90960302	92061182	rs77554484	91509215	-8.07	9.41E-16
16	51982782	53180827	rs4784227	52599188	30.34	1.84E-202
16	53297908	55183802	rs62048402	53803223	-9.86	4.09E-23
16	55879792	56920987	rs2432539	56420987	5.56	3.32E-08
16	80141031	81176117	rs7500067	80648296	-10.79	1.73E-27
16	86532855	87591139	rs4496150	87085237	-5.76	7.85E-09
17	28664023	29771319	rs7223535	29211667	-6.27	3.10E-10
17	40244470	41336389	rs72826962	40836389	5.86	4.90E-09
17	42971489	45365603	rs118045117	44252468	-7.21	5.01E-13
17	52474643	53771918	rs2787486	53209774	11.12	4.63E-29
17	77268654	78304936	rs8082452	77771548	6.43	9.71E-11
18	23656018	25125756	rs170801	24500899	-8.47	1.99E-17
18	29405293	30540417	rs117618124	29977689	6.94	4.42E-12
18	41871256	43419925	rs9954058	42411803	-7.32	2.13E-13
19	12658277	14456663	rs78269692	13158277	-6.01	1.89E-09
19	16890291	17907695	rs56069439	17393925	5.81	6.73E-09
19	18010767	20157632	rs8105994	18593553	11.08	2.22E-28
19	43781492	44932840	rs1685191	44283232	-9.16	2.87E-20
19	45679043	46683586	rs11672660	46180184	5.88	4.06E-09
20	5448227	6448227	rs16991615	5948227	6.05	1.45E-09
20	48439076	49477740	rs6122906	48945911	-6.36	1.87E-10
21	15839172	17132322	rs2403907	16574455	-11.85	1.47E-32
22	27695386	30610546	rs132289	29551872	10.36	4.75E-25
22	38005356	39858037	rs4820318	38570313	-7.45	6.08E-14
22	39969249	42538786	rs5995875	40960692	12.32	4.74E-35
22	45774072	46783297	rs28512361	46283297	5.61	2.03E-08
**Colorectal cancer**						
1	182581194	183582825	rs6669796	183082825	-5.53	2.78E-08
1	221589108	222719753	rs114008224	222141545	-6.30	3.08E-10
2	218584082	219684301	rs12053514	219167965	-5.92	3.23E-09
3	40406460	41510253	rs35401364	40923718	-7.27	3.89E-13
4	94443383	95449438	rs1370821	94943383	5.50	3.99E-08
5	792983	1796486	rs2735940	1296486	-7.26	3.13E-13
5	39719972	40785970	rs1445011	40280202	-7.46	7.79E-14
5	133967220	135022977	rs4976270	134467220	-6.48	1.08E-10
6	35028378	36028378	rs6906359	35528378	-5.52	3.43E-08
6	55157261	56237971	rs62404968	55714314	-6.11	8.57E-10

8	117099247	118305397	rs16892766	117630683	-10.12	3.94E-24
8	127907190	128955694	rs6983267	128413305	-10.69	7.74E-27
10	8188998	9243313	rs1537603	8734295	-7.49	8.16E-14
10	52145248	53146093	rs10994860	52645424	-5.51	3.46E-08
10	80319132	81319132	rs704017	80819132	-5.59	1.96E-08
10	100843317	101847038	rs35564340	101344263	5.91	3.64E-09
11	61049025	62097972	rs1535	61597972	5.50	4.15E-08
11	73776167	75258059	rs193143010	74656658	-6.96	3.59E-12
11	110619694	111681130	rs3087967	111156836	9.17	5.07E-20
12	3868607	4900808	rs12818766	4376091	6.88	6.74E-12
12	50141572	51721127	rs4307773	51144432	7.26	3.37E-13
12	111333788	113184221	rs653178	112007756	6.38	1.86E-10
12	115375881	116436753	rs7315438	115891403	6.25	4.38E-10
13	33521943	34594345	rs10161980	34093518	5.86	4.66E-09
14	53910919	54919110	rs35107139	54419106	-7.69	1.84E-14
14	58683198	59711557	rs17094971	59183198	6.27	3.82E-10
15	32494756	33543455	rs2293582	33010412	8.85	7.21E-19
16	85838288	86840448	rs2696839	86340448	-5.62	2.02E-08
17	310559	1312534	rs6598833	811968	5.69	1.12E-08
18	45948805	46969962	rs11874392	46453156	11.54	6.13E-31
19	33004997	34024919	rs8112217	33518718	6.78	1.20E-11
20	5815656	7206493	rs6117251	6406440	-7.57	3.68E-14
20	32412050	33720070	rs2295444	33173883	-5.94	3.31E-09
20	48478609	49563830	rs1810502	49057488	-5.74	1.02E-08
20	60390808	61486019	rs1741640	60932414	-9.03	1.89E-19
**Lung cancer**						
1	77467507	79123626	rs71658797	77967507	6.63	3.25E-11
3	8717383	9717383	rs446975	9217383	-5.86	4.68E-09
5	775857	1864439	rs380286	1320247	-11.88	1.51E-32
6	25184606	33283086	rs116822326	31434111	8.91	5.29E-19
6	166869897	167912048	rs239935	167411788	5.69	1.29E-08
8	26844719	27844719	rs11780471	27344719	-5.64	1.69E-08
11	117608331	118628455	rs1629083	118126576	5.69	1.25E-08
12	498819	1572696	rs7953330	998819	-6.88	6.10E-12
13	32468550	33739130	rs11571833	32972626	8.09	6.12E-16
15	46990614	48077451	rs66759488	47577451	5.55	2.83E-08
15	48830854	49876624	rs77468143	49376624	-6.11	1.00E-09
15	78211803	79715568	rs55781567	78857986	21.58	3.08E-103
19	40833284	41870338	rs56113850	41353107	-8.91	5.02E-19
**Head and neck cancer**						
4	99739319	100762242	rs1229984	100239319	-7.15	8.32E-13
6	31979729	33180122	rs3828805	32636120	7.45	9.62E-14
10	125657446	126657446	rs201982221	126157446	5.74	9.50E-09
**Ovarian cancer**						
2	176487112	177572189	rs6755777	177043226	-7.55	4.31E-14
3	155838528	157202477	rs62274042	156435952	13.04	7.04E-39
5	779790	1785974	rs4449583	1284135	6.88	5.93E-12
8	82159306	83161120	rs78740005	82659306	6.13	8.53E-10
8	128826499	130090818	rs73375000	129561866	-8.09	5.81E-16
9	16336724	17482414	rs62543619	16914716	-12.52	5.97E-36
9	135649711	136655000	rs635634	136155000	6.04	1.54E-09
10	21306832	22771669	rs7084454	21821274	6.29	3.13E-10

15	91032869	92032869	rs6496746	91532869	-5.49	3.97E-08
17	42960181	45365603	rs111960572	43563349	7.46	8.89E-14
17	45469808	47055268	rs7217120	46484755	7.48	7.20E-14
19	16837555	17962094	rs4808075	17390291	8.56	1.09E-17
**Prostate cancer**						
1	87695672	88727120	rs12139208	88213014	5.59	2.58E-08
1	149748767	151474162	rs1811698	150772613	-7.55	5.33E-14
1	153260104	156190186	rs56103503	154980351	7.95	1.81E-15
1	203528177	205107227	rs4245739	204518842	10.15	3.17E-24
1	205153056	206262406	rs823121	205724302	-5.90	3.38E-09
2	8097123	9098444	rs62106670	8597123	5.82	7.11E-09
2	9591952	11362188	rs1990613	10781975	8.56	1.59E-17
2	20378105	21429067	rs9306894	20878105	-9.48	1.92E-21
2	42955654	44354377	rs7591218	43637998	9.88	2.96E-23
2	62210273	64950722	rs58235267	63277843	-14.87	1.11E-49
2	66138815	67220444	rs74702681	66652885	6.01	1.96E-09
2	85192999	86390758	rs2028900	85767735	-10.25	6.67E-25
2	111359287	112415946	rs11691517	111893096	6.98	3.51E-12
2	172710952	174734547	rs28485589	173303031	13.87	1.11E-43
2	201624502	202630308	rs6754084	202124997	-5.49	3.53E-08
2	237851347	238949107	rs11891348	238440449	-6.98	3.98E-12
2	241254433	242943157	rs77482050	242139600	-9.77	1.51E-22
3	86466639	87974275	rs17023964	87185759	-14.32	3.08E-46
3	106458845	107465492	rs1283104	106962521	-5.73	8.81E-09
3	112446985	113809549	rs12629813	113284149	-10.64	1.98E-26
3	127210138	128784711	rs11707297	127933203	-11.51	5.80E-31
3	140606063	141650026	rs6763927	141140366	-5.74	8.47E-09
3	151492162	152715437	rs182314334	152004202	6.62	4.06E-11
3	168593100	170660493	rs78416326	170074517	-17.28	5.60E-67
4	73199144	75034437	rs17804499	74442349	-8.03	9.15E-16
4	94909802	96097814	rs12510147	95521863	-10.33	4.34E-25
4	105434084	106928563	rs10007915	106065308	14.84	8.27E-50
5	728166	2399523	rs2242652	1280028	-15.22	3.46E-52
5	43791404	44892142	rs1482680	44392142	-5.89	3.58E-09
5	133328356	134363352	rs10793821	133836209	6.59	5.43E-11
5	172455855	173459030	rs9686557	172959030	-6.03	1.94E-09
5	177468915	178468915	rs4976790	177968915	5.80	6.73E-09
6	10671163	11727328	rs2018336	11217897	7.07	1.91E-12
6	29218220	33526185	rs114489703	31301771	7.95	1.39E-15
6	34049699	35332661	rs9469899	34793124	5.83	5.27E-09
6	41009901	42091125	rs10947980	41525739	-9.37	8.78E-21
6	43194598	44195371	rs4711748	43694598	5.54	3.36E-08
6	75995882	76995882	rs9443189	76495882	5.47	4.68E-08
6	108777908	109999259	rs6941125	109287209	6.34	1.99E-10
6	116593340	117786939	rs339351	117200434	-9.69	2.90E-22
6	152858706	153950489	rs6557265	153433402	9.63	1.04E-21
6	159571652	161865436	rs140793115	160606525	12.84	6.48E-38
7	19939334	21564647	rs12155172	20994491	9.84	9.12E-23
7	26704732	28538082	rs10486567	27976563	-14.20	2.04E-45
7	40282572	41400398	rs17621345	40875192	7.53	6.72E-14
7	46937072	48014360	rs4724578	47482829	-7.05	1.60E-12
7	97134876	98595655	rs4727386	97688440	13.19	1.17E-39

8	22882594	24048805	rs11135766	23533623	-16.14	5.09E-59
8	25391729	26434684	rs11135910	25892142	7.11	9.19E-13
8	42973748	43973748	rs8175525	43473748	7.29	3.19E-13
8	127235049	129170924	rs11986220	128531689	29.10	1.10E-187
9	18051961	19598265	rs1048169	19055965	-7.52	6.53E-14
9	21541998	22541998	rs17694493	22041998	-6.37	2.26E-10
9	33303408	34549779	rs10122495	34049779	-5.69	1.34E-08
9	109642648	110807810	rs77334358	110256979	-7.83	5.17E-15
9	132049740	133097840	rs1182	132576060	6.12	1.10E-09
9	139673894	140673894	rs118073815	140173894	6.75	1.39E-11
10	334057	1452816	rs34487581	897201	7.11	9.34E-13
10	45003571	46648326	rs7075427	46104943	7.75	1.02E-14
10	47034989	48171874	rs6602880	47546323	-8.52	2.10E-17
10	50955870	52095095	rs10993994	51549496	25.94	2.29E-147
10	89692003	90695149	rs1935581	90195149	-5.82	6.55E-09
10	103721854	105032828	rs12570611	104421679	-8.22	2.54E-16
10	114211755	115212154	rs7094871	114712154	-5.44	4.84E-08
10	122251994	123554018	rs1004934	122796182	7.06	1.39E-12
10	126082160	127237997	rs11245446	126650696	-7.00	2.05E-12
11	1007512	2805799	rs10840603	2233797	17.40	4.38E-68
11	7047587	8047587	rs61890184	7547587	5.79	6.56E-09
11	61408440	62429242	rs2277283	61908440	-6.27	3.03E-10
11	66372320	67576064	rs12785906	66951966	5.79	7.82E-09
11	68311777	69963679	rs12795301	68992285	20.96	3.07E-98
11	75625116	76767477	rs17749618	76251818	7.16	7.48E-13
11	101892380	102907191	rs12285347	102396607	9.09	1.17E-19
11	107643456	108857137	rs1800057	108143456	-5.77	8.15E-09
11	113048935	114317286	rs11214775	113807181	-7.89	3.93E-15
11	133766372	134766372	rs878987	134266372	-5.46	4.77E-08
12	12371099	13371099	rs2066827	12871099	6.00	2.31E-09
12	13905600	14923294	rs10845938	14416918	-7.15	9.80E-13
12	47863253	50211553	rs10875943	49676010	-7.91	2.35E-15
12	52727384	53882160	rs73110464	53312612	13.65	1.11E-42
12	64484142	65581229	rs7968403	65012824	6.93	3.38E-12
12	89625089	90727779	rs35644221	90227779	-6.86	8.14E-12
12	114110142	115190513	rs10774740	114666202	-9.04	1.63E-19
12	132551210	133636860	rs7295014	133067989	-6.14	9.50E-10
13	73201852	74642058	rs7996468	73714290	-6.92	4.37E-12
14	22805649	23805649	rs1004030	23305649	5.63	1.55E-08
14	36587647	37641989	rs11629412	37138294	6.99	2.34E-12
14	52663421	53970916	rs62003551	53424320	8.59	8.46E-18
14	68526379	69634264	rs767127	69134264	-6.48	1.00E-10
14	70176661	71596344	rs11158871	71091142	-5.84	5.60E-09
15	40377322	41469222	rs4924487	40922915	5.71	1.32E-08
15	55885868	56906361	rs33984059	56385868	5.72	1.10E-08
15	66087581	67339282	rs80326387	66705043	6.23	4.64E-10
16	57151924	58193055	rs11863709	57654576	-6.73	1.78E-11
16	81662812	82683403	rs8052913	82166181	-5.65	1.71E-08
16	89440386	90440386	rs13332673	89940386	5.70	1.18E-08
17	-64104	1185640	rs684232	618965	-10.15	4.34E-24
17	7071752	8424746	rs28441558	7803118	-8.28	1.02E-16
17	29580257	30604598	rs142444269	30098749	-6.28	3.19E-10

17	35541031	36719586	rs11263763	36103565	25.41	3.06E-141
17	45458009	48013127	rs117576373	46820676	10.41	2.03E-25
17	55956120	56987636	rs2680708	56456120	-5.63	1.58E-08
17	68550570	69743542	rs9911515	69115358	20.48	6.47E-93
18	51269949	52272473	rs8093601	51772473	5.57	2.31E-08
18	52730859	54015052	rs28607662	53230859	-5.57	2.85E-08
18	56241676	57246356	rs12956892	56746315	5.79	7.68E-09
18	72535513	73543846	rs10460109	73036165	5.51	3.48E-08
18	76234830	77302013	rs9959454	76770820	9.69	5.35E-22
19	16680358	17728554	rs11666569	17214073	-5.73	8.17E-09
19	31667803	32668343	rs118005503	32167803	-5.78	7.31E-09
19	38047277	39377997	rs12610267	38744733	12.23	4.87E-34
19	41475688	43200947	rs74738513	41985931	-10.17	1.64E-24
19	50835049	51918257	rs62113212	51360840	-19.04	4.26E-81
20	48997045	50091337	rs7274624	49563100	-7.12	9.90E-13
20	51917890	52971030	rs6068688	52456926	-8.10	4.36E-16
20	60501851	61517081	rs2427347	61017081	-5.56	2.76E-08
20	61698236	62931824	rs1058319	62374389	-10.17	1.92E-24
21	42271554	43415988	rs145013758	42897136	7.11	1.15E-12
22	19249525	20258399	rs1978060	19749525	-6.84	8.54E-12
22	28388939	29388939	rs9625483	28888939	5.58	2.43E-08
22	39902817	41442021	rs6001723	40428706	-7.36	2.23E-13
22	42679613	44069608	rs5759167	43500212	-17.79	5.55E-71

3. It would be interesting to see whether combinations of identified GWAS loci/regions (5×10^{-8} +/– 500 kb) in different solid cancers could explain a higher proportion of the GWAS calculated h^2_g. For example, can the lung cancer 5×10^{-8} GWAS loci explain a proportion of the head and neck GWAS calculated h^2? Perhaps excluding these regions would be more informative in assessing whether how much of the overlap is due mostly to large numbers of unidentified GWAS loci (i.e. how is the genetic sharing affected if the 5×10^{-8} regions are excluded?).

Response: We thank the reviewer for raising this interesting point. We have performed two additional analyses to address this question. In results shown in Supplement 3, we present the heritability of each cancer using 1) all SNPs, 2) SNPs after removing identified GWAS loci from the cancer under study (results also shown in the original Supplementary Table 1), and 3) SNPs after removing identified GWAS loci for each of the other 5 cancers.

For most of the cancers, the GWAS significant loci for that particular cancer explain the most of its heritability. For some cancers, however, significant GWAS loci of other cancers also explain a non-trivial part of its heritability. For example, the significant breast cancer GWAS loci explained 10%, 15% and 22% heritability of colorectal, ovarian and prostate cancer, respectively; the significant colorectal cancer GWAS loci explained 11% heritability of prostate cancer; the significant lung cancer GWAS loci explained 10% heritability of head/neck cancer; and the significant prostate cancer GWAS loci explained 11% and 15% heritability of breast and ovarian cancer, respectively. These findings are consistent with the main genetic correlation results and reflect the shared genetic basis between cancers. We have described these results explicitly in the manuscript as well as added a supplementary table.

Supplement3. Estimates of SNP-heritability on the liability scale based on HapMap3 SNPs using LD score regression for each cancer, remove GWAS significant hits.

cancer type	All SNPs	Remove GWAS significant hits +/- 500 kb of	Heritability after removing GWAS significant hits +/- 500 kb of different cancers

		the same cancer													
		h^2	Prop. exp	Breast cancer	Prop. exp	Colorectal cancer	Prop. exp	Head/ neck cancer	Prop. exp	Lung cancer	Prop. exp	Ovarian cancer	Prop. exp	Prostate cancer	Prop. exp
**Breast**	0.14 (0.012)	0.077 (0.0043)	0.45	NA	NA	0.138 (0.011)	0.01	0.14 (0.012)	0.00	0.14 (0.011)	0.00	0.138 (0.012)	0.01	0.125 (0.009)	**0.11**
**Colorectal**	0.090 (0.0089)	0.072 (0.0074)	0.20	0.081 (0.0089)	**0.10**	NA	NA	0.089 (0.0088)	0.01	0.089 (0.0094)	0.01	0.089 (0.009)	0.01	0.087 (0.0094)	0.03
**Head/neck**	0.097 (0.023)	0.097 (0.023)	0.00	0.094 (0.022)	0.03	0.095 (0.022)	0.02	NA	NA	0.087 (0.021)	**0.10**	0.096 (0.022)	0.01	0.094 (0.023)	0.03
**Lung**	0.075 (0.011)	0.056 (0.0058)	0.25	0.068 (0.011)	0.09	0.074 (0.012)	0.01	0.074 (0.011)	0.01	NA	NA	0.074 (0.011)	0.01	0.070 (0.011)	0.07
**Ovarian**	0.033 (0.0065)	0.025 (0.0051)	0.24	0.028 (0.0064)	**0.15**	0.033 (0.0062)	0.00	0.032 (0.0065)	0.03	0.033 (0.0065)	0.00	NA	NA	0.028 (0.0061)	**0.15**
**Prostate**	0.18 (0.021)	0.083 (0.0058)	0.54	0.14 (0.013)	**0.22**	0.16 (0.013)	**0.11**	0.18 (0.022)	0.00	0.18 (0.012)	0.00	0.18 (0.021)	0.00	NA	NA

In Supplement 4, we calculated the cross-cancer genetic correlation based on data after excluding the GWAS significant regions of each cancer. The estimates were mostly consistent with the results calculated based on all SNPs. We compared the two sets of genetic correlations in a scatter plot (before *vs.* after removing GWAS significant regions), and got a Spearman's correlation coefficient of 0.97, which may indicate that the genetic sharing could be affected to some small extent if the 5×10^{-8} regions are excluded. We have added a sentence to the manuscript describing these results.

Supplement4. Estimates of cross-cancer genetic correlation based on HapMap3 SNPs using LD score regression for each cancer and its subsets, top hits with p-values < $5x10^{-8}$ +/- 500kb region were excluded.

**cancer type**	**Breast cancer**	ER-positive	ER-negative	**Colorectal cancer**	**Head and neck cancer**	**Lung cancer**	Adenocarcinoma	Ever smoking	Squamous cell	**Ovarian cancer**	Serous invasive	**Prostate cancer**	Advanced stage
**Breast cancer**	1	**0.97 (0.006), <1.0E-200**	**0.74 (0.03), 1.64E-142**	**0.15 (0.05), 0.003**	0.006 (0.07), 0.92	**0.23 (0.048), 0.00000091**	**0.26 (0.058), 0.000009**	**0.30 (0.07), 0.0000066**	**0.24 (0.07), 0.0003**	**0.34 (0.08), 0.00002**	**0.28 (0.08), 0.0005**	**0.12 (0.045), 0.008**	0.06 (0.052), 0.28
ER-positive		1	**0.6 (0.04), 4.8E-45**	**0.17 (0.06), 0.004**	-0.044 (0.084), 0.60	**0.15 (0.049), 0.0027**	**0.18 (0.059), 0.002**	**0.18 (0.062), 0.003**	**0.17 (0.074), 0.025**	**0.28 (0.08), 0.0005**	**0.22 (0.08), 0.007**	**0.12 (0.048), 0.010**	0.067 (0.057), 0.24
ER-negative			1	**0.16 (0.065), 0.016**	**0.17 (0.093), 0.07**	**0.35 (0.062), 0.00000002**	**0.29 (0.072), 0.000039**	**0.38 (0.081), 0.0000035**	**0.41 (0.094), 0.00001**	**0.32 (0.10), 0.002**	**0.27 (0.10), 0.0084**	0.01 (0.06), 0.08	0.01 (0.07), 0.91
**Colorectal cancer**				1	-0.12 (0.11), 0.30	**0.32 (0.07), 0.0000035**	**0.35 (0.084), 0.000029**	**0.29 (0.085), 0.00053**	**0.27 (0.1), 0.0072**	-0.069 (0.11), 0.54	-0.04 (0.11), 0.68	**0.17 (0.059), 0.005**	0.105 (0.082), 0.20
**head and neck cancer**					1	**0.60 (0.12), 0.00000035**	**0.54 (0.14), 0.00019**	**0.63 (0.14), 0.0000096**	**0.62 (0.15), 0.00005**	0.089 (0.17), 0.59	0.24 (0.15), 0.11	0.16 (0.10), 0.11	**0.26 (0.13), 0.038**
**Lung cancer**						1	**0.80 (0.035), 8.1E-120**	**1.06 (0.034), 7.8E-207**	**0.94 (0.055), 1.2E-65**	**0.24 (0.1), 0.016**	**0.27 (0.099), 0.0066**	0.11 (0.061), 0.079	**0.17 (0.075), 0.023**
Adenocarcinoma							1	**0.89 (0.054), 5.7E-61**	**0.56 (0.11), 0.0000015**	0.11 (0.12), 0.36	0.14 (0.12), 0.23	**0.18 (0.073), 0.015**	0.15 (0.093), 0.10
Ever smoking								1	**0.92 (0.073), 1.2E-36**	0.23 (0.13), 0.072	0.24 (0.13), 0.064	0.069 (0.073), 0.35	0.087 (0.095), 0.36
Squamous carcinoma									1	0.14 (0.14), 0.29	0.24 (0.14), 0.08	0.0056 (0.078), 0.94	0.13 (0.099), 0.20
**Ovarian cancer**										1	**0.96 (0.026), 3.0E-307**	-0.034 (0.084), 0.68	0.0083 (0.104), 0.94
Serous invasive											1	0.011 (0.082), 0.89	-0.034 (0.094), 0.72
**Prostate cancer**												1	**0.85 (0.036),**

Advanced stage		**6.7E-127**<br>1

The genetic correlations among cancer pairs, in the brackets were standard errors, followed by p-values. Bold red font: results withstood multiple corrections (Bonferroni correction, P < 0.05/78 = 0.00064); black bold font: results with nominal significance (P < 0.05).

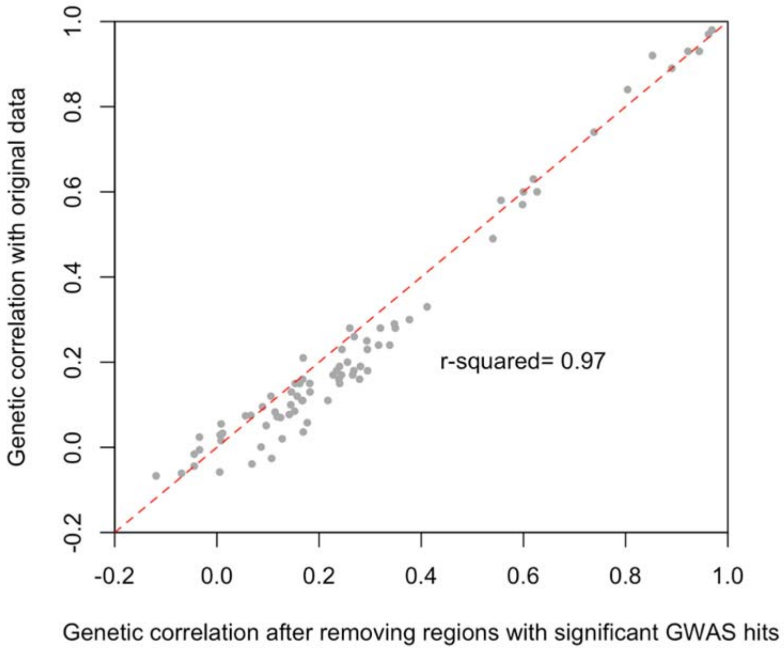

4. Some comment concerning whether population structure differences between studies of different European populations (i.e. potential for differences in population groups between different cancers) and how this could affect LDSC.

Response: Intra-European differences as a source of bias in LDSC has been examined by a previous work of Bulik-Sullivan *et al.* (PMID 25642630). To explore the stability of LD Score across European-ancestry populations, the authors estimated LD Scores using each of the 1000 Genomes Project EUR subpopulations separately (Utah residents with Northern and Western European ancestry (CEU), British in England and Scotland (GBR), Toscani in Italia (TSI) and Finnish in Finland (FIN)). The LD Scores from all four subpopulations were highly correlated, but mean LD Score increased with latitude, consistent with the observation that southern European populations have gone through less severe bottlenecks than northern European populations.

The authors evaluated the impact of these differences on the behavior of the LD Score regression analysis and found that the EUR reference panel was adequate for studies in outbred populations of predominantly northern European ancestry, such as European-American or UK populations. Therefore, 1000 Genomes Project European ancestry reference panel LD Scores are a good approximation to in-sample LD Scores. For genetic correlation analyses, the LD score in the estimating equation is the cross product of the linkage disequilibrium correlations from the two GWAS samples (this reduces to the sum of squared correlations when both GWAS are identical, i.e. when calculating heritability for a single trait). Again, considering that our studies are predominantly of European-ancestry subjects (our data were based on GWAS meta-analysis from multiple individual GWAS across European ancestry populations from Europe, Australia and the US), the 1KGP reference LD scores should suffice. We believe that any population structure across cancers will have minimal effect on our results. We have added a paragraph to the discussion about this.

5. Some empiric assessment of the sensitivity of the LD regression analysis to sample size or more importantly, study origin, affect the analyses. For example, do smaller sets of squamous lung cancer derived from different studies show approach sharing of 1.0. Do these smaller squamous lung cancer sets show similar r_g with adenocarcinoma.

Response: We thank the reviewer for raising this interesting point; however, it is beyond the scope of our current analysis as we don't have access to the individual-level data. Based on our response to the previous question, we don't think the study origin would affect the genetic correlation estimates.

Due to logistical issues, we could only get a smaller set of ER-negative breast cancer derived from different studies. As shown in the table below, we extracted a smaller set (BCAC) from the overall study (BCAC+CIMBA). The genetic correlation between BCAC+CIMBA (N=133295) vs. BCAC (N=122032) ER-negative breast cancer is 1.00 (0.007). These two sets of data presented very similar genetic correlation with other cancers and subtypes.

Supplement5. The genetic correlation between ER-negative breast cancer and other cancers, overall data vs. a subset of data.

Cancer	ER-negative breast cancer (BCAC + CIMBA)	ER-negative breast cancer (BCAC)
**Breast cancer**	0.74 (0.025)	0.75 (0.023)
ER-positive	0.60 (0.032)	0.61 (0.031)
**Colorectal cancer**	0.12 (0.059)	0.13 (0.057)
**head and neck cancer**	0.21 (0.086)	0.17 (0.086)
**Lung cancer**	0.29 (0.059)	0.27 (0.056)
Adenocarcinoma	0.23 (0.063)	0.21 (0.064)
Ever smoking	0.30 (0.074)	0.29 (0.071)
Squamous carcinoma	0.33 (0.075)	0.32 (0.077)
**Ovarian cancer**	0.24 (0.089)	0.27 (0.090)
Serous invasive	0.17 (0.080)	0.22 (0.076)
**Prostate cancer**	0.05 (0.041)	0.05 (0.042)
Advanced stage	0.016 (0.058)	0.01 (0.060)

6. Last sentence in abstract is a bit strong – would suggest "…… suggests that solid tumors arising across tissues in part share a common germline genetic basis."

Response: We thank the reviewer for this edit. We have now modified the last sentence as the reviewer suggested, it reads, "Our comprehensive analysis of cross-cancer heritability suggests that solid tumors arising across tissues *in part* share a common germline genetic basis."

REVIEWERS' COMMENTS:

Reviewer #1 (Remarks to the Author):

The authors have addressed my comments. I do not have any further comments.

Reviewer #2 (Remarks to the Author):

The authors have addressed the concerns and suggestions provided in the initial review and the manuscript is substantially enhanced.

Reviewer #1 (Remarks to the Author):

The authors have addressed my comments. I do not have any further comments.

Response: Thank you. We appreciate this positive feedback.

Reviewer #2 (Remarks to the Author):

The authors have addressed the concerns and suggestions provided in the initial review and the manuscript is substantially enhanced.

Response: Thank you. We appreciate this positive feedback.
